# Supplementary material for: Barriers and enablers to blood culture sampling in Indonesia, Thailand and Viet Nam: a Theoretical Domains Framework-based survey
Source: BMJ Open. 2024 Feb 19;14(2):e075526. doi: 10.1136/bmjopen-2023-075526 (PMC10882306; doi:10.1136/bmjopen-2023-075526)
Supplement: Supplementary data [file bmjopen-2023-075526supp001.pdf]

Appendices: Barriers and enablers to blood culture sampling in Indonesia, Thailand and Vietnam: a Theoretical Domains Framework (TDF)-based survey

Appendix S1: Supplementary Text

Supplementary Methods

The healthcare systems in SEA are highly diverse.[1] In 2020, Indonesia (GDP per capita: 3,869.6 US$) and Vietnam (GDP per capita: 2,785.7 US$) were a lower-middle-income country and Thailand (GDP per capita:7,186.9 US$) was an upper-middle-income country in SEA.[2] Indonesia has a decentralised public healthcare system, in which provincial or district-level governments have the authority over most public hospitals, and a substantial private health sector. To achieve the goal of universal healthcare coverage (UHC), in 2014 the Government introduced national health insurance (Jaminan Kesehatan Nasional), which had reached 84% of the population by 2021. Thailand achieved the status of UHC in 2002 in terms of insurance entitlement, when the gross national income per capita was 1,900 US$.[3] It is shown that UHC in Thailand can improve quality of care without undermining the efficiency and equity of the policy.[4] Vietnam has implemented social health insurance (SHI) since 1992, and SHI had a role as a financial mechanism towards achieving UHC,[5] which had reached 82% of the population in 2018. The benefit package of universal SHI in Vietnam is considered generous, particularly regarding the drugs subsidized.[5] However, out-of-pocket payments are still high.[5, 6] In 2019, percentages of out-of-pocket expenditure among all health expenditure were 35%, 9% and 43% in Indonesia, Thailand and Vietnam, respectively.[7]

Analysis

We explored the agreement between two themes of the TDF domain reinforcement. The degree of agreement between responses to the questions for barriers/enablers was estimated using the Kappa index. This describes the level of association, both positive and negative, beyond that caused by chance, as follows: 0.00–0.20, slight; 0.21–0.40, fair; 0.41–0.60, moderate; 0.61–0.80, substantial; 0.81–1.00, high.

Suntornsut P, *et al. BMJ Open* 2024; 14:e075526. doi: 10.1136/bmjopen-2023-075526

Additional analysis

We explored whether the answers of respondents who completed the survey were different from the answers of respondents who did not complete the survey. We compared the answers to the case scenario between those who completed the questionnaire and those who answered the case scenario (Question 1-3 in the questionnaire) but did not complete the questionnaire. Logistic regression model with random effects for countries was used for the analysis.

Supplementary Results

Additional results and the content themes in the domains that were identified as key domains are described in further detail in the sections below.

TDF-Reinforcement

Theme: Consequences that encourage BC sampling. Some respondents (23.7%, 294/1,243) answered that there are either positive social (e.g. praise) or positive material (e.g. a positive score) consequences if they order a BC when it is recommended. Those respondents were less likely to answer with "definitely take BC" in the case scenario (OR 0.53; 95%CI 0.37-0.74, p<0.001). We explored and found that respondents who answered that there are positive consequences that encourage BC sampling when recommended also answered that there are negative consequences that discourage BC sampling when recommended with moderate agreement beyond that expected by chance (Kappa value 0.46, p<0.001).

We also evaluated whether they are negative consequences if practitioners do not order a BC when it is recommended. Some respondents (37.7%, 464/1,230) answered that there are either negative social (e.g. verbal reprimand) or negative material (e.g. a negative score) consequences if they do not order a BC when it is recommended. Those respondents were not associated with answering with "definitely order BC" in the case scenario (p=0.42).

TDF-Emotion

Theme: Fear or anxiety of healthcare providers and *Fear or anxiety of patients or family of patients.* Some respondents (7.1%, 93/1,308) stated that there are emotional factors associated

with ordering BC. Those include fear or anxiety related to pain, needles, blood-borne diseases, high volume of blood being drawn, anaemia, etc. Those respondents were not associated with answering "definitely take BC" in the case scenario (p=0.82). Numerous quotes on this theme as a barrier were noted (Appendix S5).

TDF-Optimism

Theme: Optimism about the BC sampling and the laboratory. Most (80.5%, 1,034/1,285) respondents answered that they are strongly optimistic or optimistic that a BC will be sampled and processed in the laboratory appropriately if they order a BC. Respondents who were strongly optimistic or optimistic about the laboratory were more likely to answer with "definitely take BC" in the case scenario (OR 1.78, 95%CI 1.29-2.46, p<0.001). Most of the Thai respondents (88.3%, 263/298) are optimistic about the BC sampling and the laboratory, while 82.4% (400/487) of Indonesian respondents and 74.2% (368/496) of Vietnam respondents are (p<0.001).

TDF-Skills

Theme: Skills in drawing blood for BC. Among respondents whom were tasked to draw blood from patients for BC in their hospitals, 44.1% (143/324) answered that their skill of drawing blood from patients for BC is very good or good, 44.8% (145/324) fair, and 11.1% (36/324) poor or very poor. Respondents who answered that they have very good or good skill in drawing blood for BC was more likely to answer with "definitely take BC" in the case scenario (OR 1.74; 95%CI 1.02-2.07, p=0.04).

TDF-Memory, attention and decision processes

Theme: Patients who are already on antibiotics or have anemia. Some respondents (10.2%, 131/1,287) stated that they will definite or likely not order BC when patients are already on antibiotics even if BC is recommended. A quarter of Thai respondents (26.6%, 81/304) answered that they were very likely to still order BC, while 14.4% (72/501) of Vietnamese respondents and 3.2% (16/503) did (p<0.001, Appendix S6). Those respondents were not associated with answering with "definitely take BC" in the case scenario (p=0.13).

Suntornsut P, *et al. BMJ Open* 2024; 14:e075526. doi: 10.1136/bmjopen-2023-075526

Some respondents (22.3%, 280/1,258) answered that they will definite or likely not order BC when patients have anemia even if BC is recommended. Those respondents were not associated with answering with "definitely take BC" in the case scenario (p=0.55).

Theme: Clinical presentations for deciding to order BC. Among respondents who responded that they know of local guidelines, some stated that patients with no clinical improvement after receiving empirical antibiotics (36.2%, 274/756), presenting with fever of unknown origin (30.6%, 231/756), suspected of hospital-acquired infection (30.8%, 233/756), presenting with chronic fever (28.6%, 216/756) or suspected of infection caused by antimicrobial-resistant organisms (28.6%, 216/756) are their additional reasons to order BC.

TDF-Belief about capabilities

Theme: Belief in their own capability to draw blood. Most respondents (73.9%, 244/358) answered that they are strongly confident or confident that they can draw BC successfully. Those respondents were not associated with answering with "definitely take BC" in the case scenario (p=0.36). Most respondents (74.8%, 246/329) also answered that they are strongly confident or confident that they can draw BC appropriately using aseptic technique. Those respondents were not associated with answering with "definitely take BC" in the case scenario (p=0.11).

Theme: Belief in capability of those who are tasked to draw blood. Most respondents (88.5%, 1,151/1,300) answered that they are strongly confident or confident that those who are tasked to draw BC can draw BC successfully. Those respondents were not associated with answering with "definitely take BC" in the case scenario (p=0.13). Most respondents (76.7%, 996/1,298) also answered that they are strongly confident or confident that those who are tasked to draw BC can draw BC appropriately using aseptic technique. Those respondents were not associated with answering with "definitely take BC" in the case scenario (p=0.23).

Additional analysis

We explored whether there was any evidence showing a difference between respondents who completed and did not complete the survey. Of 2,095 respondents who agreed to participate the online survey, 1,308 (62.4%) completed the questionnaire, 256 (12.2%) answered the question

about the case scenario (Question 1-3) but did not complete the questionnaire, and 531 (25.3%) did not answer up to the question about the case scenario. The proportion of patients who answered that they would definitely take BC for the case scenario was not different between those who completed the questionnaire (52.1%; 682/1,308) and those who answered the question about the case scenario but did not complete the questionnaire (51.2%; 131/256) (p=0.08).

Appendix S2. Theoretical Domains Framework: Definitions and examples

TDF domain and definition	Examples related to blood culture (BC) sampling
**TDF-1 Knowledge:** awareness of the existence of something	In the context of this study, knowledge of the condition/scientific rationale could relate to their knowledge of:<br>• when and whom BC should be sampled<br>• local and international guidelines for BC sampling<br><br>Knowledge may be both correct and incorrect
**TDF-2 Skills:** ability or proficiency acquired through practice	In the context of this study, skills/competence include skill of participant to draw blood for BC sample collection.<br><br>Skills may be both present and absent
**TDF-3 Social professional role and identity:** a coherent set of behaviours and displayed personal qualities of an individual in a social or work setting	In the context of this study, professional role may relate to the extent that healthcare professionals feel that ordering or initiating an order for BC are part of their professional role or their job description.<br><br>Personal identity may relate to how a participant views their role of<br>• ordering or initiating an order for BC<br>• drawing blood for BC
**TDF-4 Beliefs about capabilities:** acceptance of the truth/reality about or validity of an ability, talent or facility that a person can put to constructive use	In the context of this study, beliefs about capabilities relates to the judgments on medical doctor/final-year medical student's ability to:<br>• draw blood successfully<br>• draw blood appropriately<br><br>As BC may be ordered by respondents but collected by other professionals, beliefs about capabilities also include their judgments on the ability of persons who are tasked to draw blood<br><br>"Successfully" means obtaining blood, and "Appropriately" means that general guidelines for BC specimen collection such as aseptic technique are followed.
**TDF-5 Optimism:** confidence that things will happen for the best or that desired goals will be attained	In the context of this study, optimism related to their judgment regarding that a BC will be sampled and processed in the laboratory appropriately if they order a BC.

TDF domain and definition	Examples related to blood culture (BC) sampling
	This includes optimism and pessimism.
**TDF-6 Beliefs about consequences***:* acceptance of the truth/reality about or validity of outcomes of a behaviour in a given situation	In the context of this study, beliefs about their judgments on:<br>• the purpose, value, and effectiveness of BC<br>• negative/positive outcomes of BC
**TDF-7 Reinforcement:** increasing the probability of a response by arranging a dependent relationship, or contingency, between the response and a given stimulus	In the context of this study, reinforcements relate to their judgments on:<br>• receiving an incentive or reward (these can be social [e.g. praise] or material [e.g. a positive score]) for ordering a BC when recommended<br>• receiving any negative consequences (these can be social [e.g. verbal reprimand or that you/doctors are at risk of being scrutinized] or material [e.g. a negative score]) for not ordering BC when recommended<br><br>As feedbacks could discourage the behavior, reinforcement also include judgements on:<br>• receiving any negative consequences for ordering BC when recommended
**TDF-8 Intentions:** conscious decision to perform a behaviour or a resolve to act in a certain way	In the context of this study, intentions relate to the statements on their intention to order BC.
**TDF-9 Goals:** mental representation of outcomes or end states that an individual wants to achieve	In the context of this study, goals relate to the statements on:<br>• the goals they wish to collect BC prior to giving empirical antibiotics<br>• competing goals (goals that might conflict with BC collection; e.g. giving empirical antibiotics)
**TDF-10 Memory, attention and decision processes:** ability to retain information, focus selectively on aspects of the environment and choose between two or more alternatives	In the context of this study, memory, attention and decision processes relate the statements on how they decide whether to order or not order BC
**TDF-11 Environmental context and resources:** any circumstances of a person's situation or environment that discourages or encourages the development of skills and abilities, independence, social competence, and adaptive behaviour	In the context of this study, environmental context and resources relates to their perceptions of the:<br>• Availability of consumables such as bottles, needles, syringes, blood collection set, etc.<br>• Availability of microbiology laboratories<br>• Financial resources, whether patients have to pay out-of-pocket<br>• Cost-effectiveness of BC

TDF domain and definition	Examples related to blood culture (BC) sampling
**TDF-12 Social influences:** interpersonal processes that can cause an individual to change their thoughts, feeling or behaviours.	In the context of this study, social influences relate to the statements expressing the influence of others on attending BC. Including:<br>• norms<br>• influences from nurses, other medical doctors, consultants, head of department, executive of the hospitals, patients and family of patients<br><br>"Norms" mean usual practice that are typical of or accepted within their hospital.
**TDF-13 Emotion:** a complex reaction pattern, involving experiential, behavioural and physiological elements, by which the individual attempts to deal with a personally significant matter or event	In the context of this study, emotions relate to the statements of expressing their emotional reaction/state relating to order and sample for BC<br><br>Any logical reasons or social influence which are stated as "fear of" are categorized as "Memory, attention and decision processes" or "Social influence" as appropriate.
**TDF-14 Behavioural regulation:** anything aimed at managing or changing objectively observed or measured actions	In the context of this study, behavioural regulation relates to the statements about managements or steps taken to<br>• order BC<br>• adopt local/national/international guidelines for BC sampling

Appendix S3. TDF-based questionnaire

Online research participant information sheet and electronic consent form

You are invited to participate in a web-based online survey on **"Barriers and facilitators to ordering blood culture samples in Indonesia, Thailand and Viet Nam".** This is a research project being conducted under the collaboration between Eijkman Oxford Clinical Research Unit (EOCRU), **Indonesia,** and Mahidol Oxford Tropical Medicine Research Unit (MORU), Faculty of Tropical Medicine, Mahidol University, **Thailand**, Oxford University Clinical Research Unit (OUCRU), **Viet Nam,** Centre for Behaviour Change, University College London, **United Kingdom**.

PROPOSE: This study aim to identify barrier and facilitators to the adoption of blood culture sampling recommendations in Indonesia, Thailand and Viet Nam

PARTICIPATION: The participants include 1,500 medical doctors and final-year medical students in Indonesia, Thailand and Viet Nam (500 participants per country). The survey is voluntary. You may refuse to take part in the research or exit the survey at any time without penalty. You are free to decline to answer any particular question you do not wish to answer for any reason.

PROCEDURE: You may have received an invitation from clinical directors, head of final-year medical student, or head of recently graduated medical doctors to do this online survey. You may also receive two email reminders about the invitation. We also ask final-year medical students and medical doctors in those hospitals to share the invitation to the survey to any final-year medical students and medical doctors in the country using their networks such as Facebook, Line and WhatsApp application.

In this survey, we will ask whether you know of any local and international guidelines on when to perform blood culture sampling, whether you would perform blood culture sampling for the constructed case scenario, and why you do or do not perform blood culture sampling. It should take approximately 30 – 40 minutes to complete.

All study data will be entered on a Qualtrics. The participants will be identified by a unique study specific number and/or code in any database. We will ask for your email account or telephone number in order to provide you an electronic gift. You may refuse to providing your email account or telephone number and to receiving an electronic gift. The name and any other identifying detail will NOT be included in any study data electronic file.

BENEFITS: You will receive no direct benefits from participating in this research study. However, your responses may help us learn more about what are barriers and facilitators of doctors to order and collect blood culture samples per local, national or international recommendations in different countries. The questionnaire focuses only on when and why blood culture is sampled. Participants will receive a gift or cash (about $4 USD in value) for completing the questionnaire. Participants could receive the gift electronically if email account or telephone number is provided.

RISKS: There is the risk that you may find some of the questions to be sensitive, and that some questions may cause emotional discomfort. Nonetheless, the possible risks or discomforts of the study are minimal. If you feel uncomfortable or distressed at any time during this survey, you should feel free to terminate participation. You are free to decline to answer any particular question you do not wish to answer for any reason. The study team does not expect any risks for participants beyond the minimal risks described above regarding confidentiality surrounding sensitive comments that might arise when participating in the qualitative interviews.

WITHDRAWAL: The survey is voluntary. You can withdraw from the study without penalty at any time and you are free to decline to answer any particular question you do not wish to answer for any reason with no obligation to give the reason for withdrawal.

CONFIDENTIALITY: Although we will collect your identifying information such as your medical license number (student identification no if you are a medical student), email address and telephone number, your identifying information are needed for compensation and your identifying information will be known only to the researchers performing this study or to specific groups for auditing purposes (if requested). These groups are government institutions or organisations authorised to conduct audits such as the ethics committee. Only summary results will be published and anonymous information will be put in open-access scientific database. No one will be able to identify you or your answers, and no one will know whether you participated in the study.

ETHICAL: The study protocol, informed consent form, participant information sheet and any proposed advertising material will be submitted to OxTREC, the ethics Committee of the Faculty of Tropical Medicine, Mahidol University, Thailand and (FTMEC), and local ethics committees for written approval.

CONTACT: If you have questions at any time about the study or the procedures, you may contact Dr Ralalicia Limato (rlimato@eocru.org) in Indonesia, Pornpan Suntornsut (pornpan@tropmedres.ac) in Thailand, and Dr Vu Thi Lan Huong (huongvtl@oucru.org) in Viet Nam.

DATA PROTECTION: The University of Oxford is responsible for ensuring the safe and proper use of any personal information you provide, solely for research purposes.

DATA SHARING: Data collected for this study will be de-identified and may be shared with other groups of researchers in accordance with the current MORU Data Sharing Policy. All applications will be carefully reviewed by the MORU Data Access Committee before granting any approvals to access data. All researchers accessing the data need to adhere to a set of terms and conditions that aim to protect the interests of research participants and other relevant stakeholders.

INTERNET AND DEVICE REQUIREMENT: This online questionnaire requires good internet connection and relatively up-to-date devices. Mobile devices with small screens may not show the questions clearly. If your devices are relatively out-of-date or with small screens, we recommend you to use a desktop computer at a place with good internet connection. If you have a problem with the online questionnaire, you may ask for the word file (.doc) or the paper questionnaire by contacting Dr Ralalicia Limato (rlimato@eocru.org) in Indonesia, Pornpan Suntornsut (pornpan@tropmedres.ac) in Thailand, and Dr Vu Thi Lan Huong (huongvtl@oucru.org) in Viet Nam.

ELECTRONIC CONSENT: Please select your choice below. You may print a copy of this consent form for your records. Clicking on the "Agree" button indicates that I agree to participate in the research study. I have read the above information and I am participating voluntarily.

○ Agree
○ Disagree

EXPLANATION: The questionnaire may contain ○ for radio button (can take only one answer) ☐ for multiple choices (can take more than one answer)) and open text answer as well. Please indicate your level of opinion and mark in the button or box of your answer.

Q1-1. **At which type of hospital are you currently working?** If you are currently working at more than one hospital, select where you are currently spending most time. (please select the most relevant answer)

○ Government hospital (including National hospital, Provincial hospital, District hospital)
○ Private hospital
○ University hospital
○ I do not want to answer

10

○ Other: ……………
Q1-2. **What is your Medical license number or student ID number?** This is to confirm that you are a medical doctor or a final-year medical student in Indonesia, Thailand or Viet Nam. If you are not a medical doctor or a final-year medical student in Indonesia, Thailand or Viet Nam, you should not participate in this questionnaire. Your identifying information will be known only to the researchers. No one will be able to identify you or your answers, and no one will know whether you participated in the study.<br>………………………………………………………………….
Q1-3. **As an introduction to the topic blood culture sampling, we present a case scenario to you. We would like to know if you consider taking blood culture samples in your everyday clinical practice and your current hospital setting.**<br><br>**If you are currently working at more than one hospital, please consider the hospital you are spending most time as your current hospital setting.**<br><br>**Case scenario.** "A 72-year-old woman who was brought to the emergency department of your hospital by her daughter when she noticed the patient was more confused than her baseline and was found to have a high fever and fast breathing. She had an auscultatory finding compatible with pneumonia. It is decided that this patient will be admitted to your hospital."<br><br>If you have an authority to take a blood culture, would you take blood culture sample(s) in this case on admission?<br><br>○ Definitely (>95-100% of the time)<br>○ Likely (75-95% of the time)<br>○ Maybe (25-74% of the time)<br>○ Unlikely (5-24% of the time)<br>○ Rarely (ranging from never <5% of the time)<br>○ I do not know<br>○ I do not want to answer
Q1-4. Do you know of any recommendation(s) or guideline(s) for blood culture sampling being used in your hospital?<br><br>○ Yes<br>○ No, my hospital does not use any recommendations or guidelines for blood culture sampling (go to Q1-8)<br>○ I do not know if my hospital uses any recommendations or guidelines. (go to Q1-8)<br>○ I do not want to answer (go to Q1-8)
(Page break)
Q1-5. **Based on your understanding**, do any following statement(s) represent the recommendation(s) or guideline(s) for blood culture sampling being used in your hospital? (you can select more than one answer)<br><br>☐ Recommend blood culture sampling in all patients presenting with SIRS (Systemic inflammatory Response Syndrome [SIRS] is defined as having at least two of the following criteria: fever or hypothermia, tachycardia, tachypnea, and leukocytosis or leucopenia)<br>☐ Recommend blood culture sampling in all patients presenting with sepsis ('sepsis' here is defined as an acute change in total Sequential Organ Failure Assessment [SOFA] score ≥2 points consequent to the infection based on the most recent definition of sepsis [Sepsis-3 criteria])<br>☐ Recommend blood culture sampling in all patients presenting with septic shock<br>☐ Recommend blood culture sampling in all patients starting parenteral antibiotic treatment<br>☐ Recommend blood culture sampling in all patients with no clinical improvement after receiving empirical antibiotics

☐ Recommend blood culture sampling in all patients presenting with infection and having underlying diseases
☐ Recommend blood culture sampling in all patients with chronic fever
☐ Recommend blood culture sampling in all patients with fever of unknown origins
☐ Recommend blood culture sampling in all patients suspected of infections caused by atypical organisms
☐ Recommend blood culture sampling in all patients suspected of infections caused by antimicrobial-resistant organisms
☐ Recommend blood culture sampling in all patients suspected of infections caused by multiple-drug-resistant organisms
☐ Recommend blood culture sampling in all patients suspected of hospital-acquired infections
☐ I do not know
☐ I do not want to answer
☐ Other: ……………

Due to many factors, there are times that doctors can not follow the recommendation(s) or guideline(s).

Q1-6. In your current hospital setting, how often do you plan to follow the recommendation(s) or guideline(s) for blood culture sampling being used in your hospital?

○ All the time (>95-100% of the cases)
○ Often (75-95% of the cases)
○ Moderately (25-74% of the cases)
○ Occasionally (5-24% of the cases)
○ Rarely (ranging from never to <5% of the cases)
○ I do not know
○ I do not want to answer

Q1-7. Apart from the recommendation(s) or guideline(s) being used at your hospital (as you answered in the previous question), do you have **any additional reasons** for deciding to do blood culture sampling? (you can select more than one answers that are applicable to your current hospital setting)

☐ No. All reasons are stated in the recommendation(s) or guideline(s) being used in my hospital.
☐ Patients presenting with chills
☐ Patients presenting with sepsis
☐ Patients presenting with septic shock
☐ Patients starting parenteral antibiotic treatment
☐ Patients with no clinical improvement after receiving empirical antibiotics
☐ Patients presenting with infection and having underlying diseases
☐ Patients presenting with chronic fever
☐ Patients presenting with fever of unknown origin
☐ Patients suspected of infections caused by atypical organisms
☐ Patients suspected of infections caused by antimicrobial-resistant organisms
☐ Patients suspected of infections caused by multiple-drug-resistant organisms
☐ Patients suspected of hospital-acquired infections
☐ Laboratory results showing leukocytosis
☐ Laboratory results showing neutropenia
☐ Laboratory results showing left shift in blood count (i.e. showing immature white blood cells)
☐ Laboratory results showing CRP increase
☐ Laboratory results showing procalcitonin increase
☐ Patients can afford the cost of blood culture
☐ Patients have a health scheme or insurance that covers the cost of blood culture

☐ Patients are likely to have a final diagnosis that includes the cost of blood culture in the package of fee for service
☐ I do not know
☐ I do not want to answer
☐ Other: ……………

(Skip to Q1-9 after this question)

(Page break)

Q1-8. In your current hospital setting, what are the **reasons** for deciding to do blood culture sampling? (you can select more than one answer that are applicable for your current hospital setting)

☐ Patients presenting with chills
☐ Patients presenting with sepsis
☐ Patients presenting with septic shock
☐ Patients presenting with infection and having underlying diseases
☐ Patients starting parenteral antibiotic treatment
☐ Patients with no clinical improvement after receiving empirical antibiotics
☐ Patients presenting with infection and having underlying diseases
☐ Patients presenting with chronic fever
☐ Patients presenting with fever of unknown origin
☐ Patients suspected of infections caused by atypical organisms
☐ Patients suspected of infections caused by antimicrobial-resistant organisms
☐ Patients suspected of infections caused by multiple-drug-resistant organisms
☐ Patients suspected of hospital-acquired infections
☐ Laboratory results showing leukocytosis
☐ Laboratory results showing neutropenia
☐ Laboratory results showing left shift in blood count
☐ Laboratory results showing CRP increase
☐ Laboratory results showing procalcitonin increase
☐ Patients can afford the cost of blood culture
☐ Patients have a health scheme or insurance that covers the cost of blood culture
☐ Patients are likely to have a final diagnosis that includes the cost of blood culture in the package of fee for service
☐ I do not know
☐ I do not want to answer
☐ Other: ……………

(Page break)

Q1-9. Are you aware of any international recommendation(s) or guideline(s) for blood culture sampling? Examples of international recommendations are surviving sepsis campaign (SSC), the diagnostic stewardship of the World Health Organization (WHO), The Infectious Diseases Society of America (IDSA) and The National Institute for Health and Care Excellence (NICE)

○ Yes
○ No (go to Q2-1)
○ I do not want to answer (go to Q2-1)

Q1-10. **Based on your understanding**, can any following statement(s) represent international recommendation(s) for blood culture sampling (you can select more than one answers)

☐ Recommend collecting blood culture in all patients presenting with sepsis
☐ Recommend collecting blood culture in all patients starting parenteral antibiotic treatment
☐ I do not know
☐ I do not want to answer
☐ Other:…………………….

(Page break)

We would like to understand your current job and how doctors in different positions are involved in ordering and collecting blood culture in your current hospital setting.

Q2-1. First, please state your current job. (please select the most relevant answer)
○ Medical doctor – working in an executive or administrative position (not doing clinical work)
○ Medical doctor – working as a consultant (defined as a doctor with a clinical specialty/subspecialty degree)
○ Medical doctor – working as a physician (defined as a doctor without a clinical specialty/subspecialty degree and not under any postgraduate clinical training)
○ Medical doctor – working as a resident/registra/fellow (defined as a doctor who is currently under any postgraduate clinical training)
○ Intern (defined as a recent medical school graduate who is in the first year of post-graduate on-the-job training)
○ Final-year medical student
○ Other:……………

Final-year medical students (and interns) in some countries or some settings can **initiate an order** for a blood culture under authority of residents, consultants or other medical doctors. The order may be supervised, signed or co-signed by residents, consultants or other medical doctors later.

Q2-2. In your current hospital setting, which types of professionals/staff **can order** a blood culture. **"Order"** means initiating an order either verbally or in writing. (you can select more than one answers)

☐ Medical doctors – working in executive or administrative positions (not doing clinical work)
☐ Medical doctors – working as consultants (defined as a doctor with a clinical specialty/subspecialty degree)
☐ Medical doctors – working as physicians (defined as a doctor without a clinical specialty/subspecialty degree and not under any postgraduate clinical training)
☐ Medical doctors – working as residents/registras/fellows (defined as a doctor who is currently under any postgraduate clinical training)
☐ Interns (defined as recent medical school graduates who are in the first year of post-graduate on-the-job training)
☐ Final-year medical students
☐ I do not want to answer
☐ Other:……………

Q2-3. Do you know when and which patients should receive an **order** for a blood culture in your hospital?

○ Definitely (>95-100% of the case)
○ Likely (75-95% of the case)
○ Uncertain (25-74% of the case)
○ Unlikely (5-24% of the case)
○ Rarely (ranging from never to <5% of the case)
○ I do not know

○ I do not want to answer
Q2-4. If you **can order** for a blood culture as per your current job description or position, do you think that it is an appropriate part of your current job (as per your job description or position) to **order** a blood culture?<br><br>○ Very appropriate<br>○ Appropriate<br>○ Uncertain<br>○ Inappropriate<br>○ Very inappropriate<br>○ I cannot order blood culture. It is not part of my job (Go to Q2-5).<br>○ I do not know<br>○ I do not want to answer<br><br>(Skip to Q2-6 after this question, except answering "I cannot order blood culture. It is not part of my job")
(Page break)
Q2-5. As you **cannot order** for a blood culture as per your current job description or position, do you think that it would be an appropriate part of your current job (as per your job description or position) to **order** a blood culture?<br><br>○ Very appropriate<br>○ Appropriate<br>○ Uncertain<br>○ Inappropriate<br>○ Very inappropriate<br>○ I do not know<br>○ I do not want to answer
(Page break)
Q2-6. In your current hospital setting, which **types of professionals** are tasked to **draw blood** from patients for blood culture. (you can select more than one answers)<br><br>☐ Medical doctors – working in executive or administrative positions (not doing clinical work)<br>☐ Medical doctors – working as consultants (defined as a doctor with a clinical specialty/subspecialty degree)<br>☐ Medical doctors – working as physicians (defined as a doctor without a clinical specialty/subspecialty degree and not under any postgraduate clinical training)<br>☐ Medical doctors – working as residents/registras/fellows (defined as a doctor who is currently under any postgraduate clinical training)<br>☐ Interns (defined as recent medical school graduates who are in the first year of post-graduate on-the-job training)☐ Interns<br>☐ Final-year medical students<br>☐ Registered nurses<br>☐ Microbiology laboratory team<br>☐ Specialized blood draw team<br>☐ I do not want to answer<br>☐ Other:……………
Q2-7. Do you think that it is an appropriate part of your job (as per your job description or position) to **draw blood**?

○ Very appropriate<br>○ Appropriate<br>○ Uncertain<br>○ Inappropriate<br>○ Very inappropriate<br>○ It is not part of my job to draw blood from patients for blood culture (go to Q2-11)<br>○ I do not know<br>○ I do not want to answer
(Page break)
Q2-8. How skilled are you in **drawing blood**?<br>○ Very good skill<br>○ Good skill<br>○ Fair skill<br>○ Poor skill<br>○ Very poor skill<br>○ I do not know<br>○ I do not want to answer
**Having confidence is different from having skills. Due to many factors, there are times that blood could not be drawn even though we are skilled.**<br><br>Q2-9. If you have to draw blood yourself, are you confident that **you can draw blood successfully**? "Successfully" means obtaining blood.<br>○ Strongly confident<br>○ Confident<br>○ Uncertain<br>○ Doubtful<br>○ Strongly doubtful<br>○ It is not part of my job to draw blood from patients for blood culture<br>○ I do not know<br>○ I do not want to answer
Q2-10. Are you confident that **you can draw blood appropriately**? "Appropriately" means that general recommendations for blood culture specimen collection such as aseptic technique are followed.<br>○ Strongly confident<br>○ Confident<br>○ Uncertain<br>○ Doubtful<br>○ Strongly doubtful<br>○ It is not part of my job to draw blood from patients for blood culture<br>○ I do not know<br>○ I do not want to answer
(Page break)
Q2-11. Are you confident that **others (who are tasked to draw blood in your hospital) can draw blood successfully**?<br>○ Strongly confident<br>○ Confident<br>○ Uncertain

○ Doubtful
○ Strongly doubtful
○ I do not know
○ I do not want to answer
○ I do not want to answer

Q2-12. Are you confident that **others (who are tasked to draw blood in your hospital) can draw blood appropriately**? "Appropriately" means that general recommendations for blood culture specimen collection such as aseptic technique are followed.

○ Strongly confident
○ Confident
○ Uncertain
○ Doubtful
○ Strongly doubtful
○ I do not know
○ I do not want to answer

Q2-13. In your current hospital setting, how **optimistic** are you that a blood culture will be sampled and processed in the laboratory appropriately if you order a blood culture? "Optimistic" means the confidence that things will happen for the best or that desired goals will be attained.

○ Strongly optimistic
○ Optimistic
○ Neither optimistic nor pessimistic
○ Pessimistic
○ Strongly pessimistic
○ I do not know
○ I do not want to answer

(Page break)

Many advantages and disadvantages of blood culture have been mentioned in surveys in different countries. This advantages and disadvantages could differ between settings.

Please answer of all following question to the best of your ability. Please a check mark "v " in the appropriate answer for each question.

Q3-1. Do you agree or disagree about the following potential advantages of blood culture, making blood culture helpful in your current hospital setting?	Strongly agree	Agree	Uncertain	Disagree	Strongly disagree	I do not know	I do not want to answer
• Blood culture is helpful in clinical decisions.							
• Blood culture is helpful to rule in an infection.							
• Blood culture is helpful to rule out an infection.							
• Blood culture is helpful in detecting antimicrobial-resistant bacterial infections.							
• Blood culture is helpful in adjusting antibiotics.							
• Blood culture can reduce overuse of antibiotics.							
• Blood culture can reduce length of hospital stay.							

• Blood culture can reduce patient mortality.							
• Accumulative results of blood culture (i.e. antimicrobial-resistance surveillance report) are helpful in understanding epidemiology of antimicrobial-resistant bacterial infections.							

Q3-2. Additional comments why blood culture is helpful in your current hospital setting (Note: limit to 2,000 characters)
…………………………………………………………………

Please answer of all following question to the best of your ability. Please a check mark "√ " in the appropriate answer for each question.

Q3-3. Do you agree or disagree about the following disadvantages of blood culture, **making blood culture unnecessary in your current hospital setting?**	Strongly agree	Agree	Uncertain	Disagree	Strongly disagree	I do not know	I do not want to answer
• Blood culture is unnecessary because antibiotic therapy can be determined based on clinical presentations.							
• The therapeutic consequence of blood culture sampling is questionable.							
• The scientific basis of the guideline on blood culture is questionable							
• Blood culture is unnecessary because results are often delayed.							
• Blood culture is unnecessary because results are often not interpretable.							
• Blood culture is unnecessary because results are often negative or no growth.							
• Blood culture is unnecessary because cultures are often contaminated.							
• Blood culture is unnecessary because results often do not agree with clinical signs.							
• Blood culture is unnecessary because a contaminated result often leads to wrong therapeutic approaches.							
• Blood culture is unnecessary because it is too expensive.							
• Blood culture is not benefiting the patients.							
• It is not too late to collect blood culture later, particularly if patients do not improve after receiving empirical antibiotic treatment.							
• Quality of laboratory is questionable.							
• Levels of local antibiotic resistance are low.							

Q3-4. Additional comments why blood culture is not helpful in your current hospital setting (Note: limit to 2,000 characters)
…………………………………………………………………

(Page break)

In different settings, other tasks may be considered more urgent than collecting blood culture samples.

Q3-5. In your current hospital setting, how often do you obtain blood culture **prior to administration of empirical antibiotics** in patients presenting with **sepsis**? '('sepsis' here is defined as an acute change in total Sequential Organ Failure Assessment [SOFA] score ≥2 points consequent to the infection based on the most recent definition of sepsis [Sepsis-3 criteria])

○ All the time (>95-100% of the time)

○ Often (75-95% of the time)
○ Moderately (25-74% of the time)
○ Occasionally (5-24% of the time)
○ Rarely (ranging from never to <5% of the time)
○ I do not know
○ I do not want to answer

Q3-6. In your current hospital setting, how often do you obtain blood culture **prior to administration of empirical antibiotics** in patients presenting with **septic shock**?
○ All the time (>95-100% of the time)
○ Often (75-95% of the time)
○ Moderately (25-74% of the time)
○ Occasionally (5-24% of the time)
○ Rarely (ranging from never to <5% of the time) ○ Rarely (ranging from never to <5% of the time)
○ I do not know
○ I do not want to answer

Even if blood culture is recommended, doctors may decide not to order blood culture in some situations.

Please answer of all following question to the best of your ability. Please a check mark "√ " in the appropriate answer for each question.

Q3-7. Would you still order blood culture in the following situation?	Definitely not order	Likely not order	Maybe not order	Likely to still order	Very likely to still order	I do not Know	I do not want to answer
• Patients are already on antibiotics.							
• Patients have anemia.							
• Blood should be used for other laboratory tests.							
• There are no local guidelines/recommendations for blood culture sampling							
• Patients do not meet certain conditions for a blood culture following the local guidelines							
• Patients do not have a health scheme or insurance that covers the cost of blood culture							
• Microbiology laboratory in your hospital is not available							

Q3-8. Additional comments why you do not order blood culture regarding situations mentioned above (Note: limit to 2,000 characters)
……………………………………………………………….

(Page break)

Resources are commonly limited in many settings worldwide.

Q4-1. In your hospital, how often could you (or doctors in your hospital) **not order blood culture** because consumables (such as blood culture bottles, needles, syringes, blood collection set, etc.) are **not available**?

○ All the time (>95-100% of the time)
○ Often (75-95% of the time)
○ Moderately (25-74% of the time)

○ Occasionally (5-24% of the time)
○ Rarely (ranging from never to <5% of the time)
○ I do not know
○ I do not want to answer

Q4-2. In your hospital, how often could you (or doctors in your hospital) **not order blood culture** because the microbiology laboratory is **not available** or not functioning?

○ All the time (>95-100% of the time)
○ Often (75-95% of the time)
○ Moderately (25-74% of the time)
○ Occasionally (5-24% of the time)
○ Rarely (ranging from never to <5% of the time)
○ I do not know
○ I do not want to answer

Q4-3. In your hospital, how often do patients have to pay for blood culture using their own money (i.e. out of pocket)?

○ All the time (>95-100% of the patients)
○ Often (75-95% of the patients)
○ Moderately (25-74% of the patients)
○ Occasionally (5-24% of the patients)
○ Rarely (ranging from never to <5% of the patients)
○ I do not know I do not know
○ I do not want to answer

Q4-4. Regardless of who pays for the cost of blood culture, would you say that the benefits of blood culture outweigh the cost?

○ Very likely
○ Likely
○ Uncertain
○ Unlikely
○ Very unlikely
○ I do not know
○ I do not want to answer

(Page break)

Positive and negative consequences could encourage us to follow guidelines.

Q5-1. Are there **any positive consequences, incentives or rewards** (these can be social [e.g. praise] or material [e.g. a positive score]) if you or doctors in your hospital **order a blood culture when recommended**? (you can select more than one answer)

☐ No
☐ Yes- social
☐ Yes- material
☐ Yes- both social and material
☐ I do not know
☐ I do not want to answer

Suntornsut P, *et al. BMJ Open* 2024; 14:e075526. doi: 10.1136/bmjopen-2023-075526

☐ Other: ……………

Q5-2. Are there **any negative consequences** to you or doctors (these can be social [e.g. verbal reprimand or that you/doctors are at risk of being scrutinized] or material [e.g. a negative score]) if you or doctors in your hospital **do not order a blood culture when recommended**? (you can select more than one answer)

☐ No
☐ Yes- social
☐ Yes- material
☐ Yes- both social and material
☐ I do not know
☐ I do not want to answer
☐ Other: ……………

Sometimes there are feedbacks that could discourage us to follow guidelines. This could be due to many reasons based on local context.

Q5-3. Are there **any negative consequences** to you or doctors (these can be social [e.g. verbal reprimand or any pressure from your supervisors/executives of your hospital as the hospital (may) have to pay for the (extra) cost of blood culture] or material [e.g. a negative score, that you/doctors are at risk of having to spend extra time and effort to reimburse the cost of blood culture from any health scheme or insurance, or that you/doctors are at risk of having to pay for the (extra) cost of blood culture yourselves]), if you or doctors in your hospital **order blood culture when recommended**? (you can select more than one answer)

☐ No
☐ Yes- social
☐ Yes- material
☐ Yes- both social and material
☐ I do not know
☐ I do not want to answer
☐ Other: ……………

Q5-4. Additional comments about feedbacks (including encouragement, punishments or any positive and negative consequences) on blood culture sampling in your hospital setting. Also, please provide more comments about whether any consequences you would recommend to implement in your hospital to support blood culture ordering.

…………………………………………………………………

(Page break)

Q5-5. In your hospital, are there **any training, lectures, classes or meetings** that provide you knowledge about local/national/international guidelines for blood culture sampling? (you can select more than one answers)

☐ No
☐ Yes, infrequently (less than once a year)
☐ Yes, occasionally (at least once a year)
☐ Yes, regularly (more than once a year)
☐ I do not know
☐ I do not want to answer

☐ Other: ……………

Q5-6. In your hospital, are there **any procedures** that support you or doctors in your hospital to order or regulate ordering of blood culture per local/national/international guidelines? (you can select more than one answers)

☐ No
☐ Yes, there is a poster (and blood culture is mentioned)
☐ Yes, there is a standard order form for patients presenting with sepsis (and blood culture is already written in the order form)
☐ Yes, there is a computer system to remind ordering blood culture
☐ Yes, there is a case review (e.g. grand round; morning ward round, clinical meetings, etc and blood culture is often mentioned)
☐ Yes, there is a stewardship programme and reviewing blood culture is included in the programme (e.g. post-prescription review and stewardship round, etc.)
☐ Yes, there is a local hospital guideline (e.g. standard operating procedure [SOP])
☐ I do not know
☐ I do not want to answer
☐ Other: ……………

(Page break)

Due to different personal beliefs, norms and limitations, blood culture sampling is encouraged or discouraged by peers and co-workers in different settings.

Q6-1. **To what extent do you or doctors in your hospital order blood culture sampling because you are following local norms? "**Norms" mean usual practice that are typical of or accepted within your hospital.

○ All the time (>95-100% of the time)
○ Often (75-95% of the time)
○ Moderately (25-74% of the time)
○ Occasionally (5-24% of the time)
○ Rarely (ranging from never to <5% of the time)
○ I do not know
○ I do not want to answer

Please answer of all following question to the best of your ability. Please a check mark "√ " in the appropriate answer for each question.

Q6-2. **Do following people have any positive or negative influence on you or doctors in your hospital to order blood culture?** Positive influence could mean facilitate, support or encourage blood culture sampling. Negative influence could mean hinder or discourage blood culture sampling.	Very positive influence	Positive influence	Neither positive nor negative influence	Negative influence	Very negative influence	I do not know	I do not want to answer
• Nurses							
• Final-year medical students							
• Interns							
• Residents (any postgraduate clinical training)							

• Doctors (defined as a doctor without a specialty/subspecialty degree and not under any postgraduate clinical training)							
• Consultants (defined as a doctor with a clinical specialty/subspecialty degree)							
• Head of the Department							
• Executives of the hospital							
• Patients							
• Family of patients							

Q6-3. Additional comments about social influence on blood culture sampling
…………………………………………………………………

Q6-4. Apart from your logical considerations, do you think that **any emotional factors** of anyone are involved in ordering and sampling for blood culture (including patients and family of patients) could influence whether blood culture is ordered or sampled? (for example: fear of blood, fear of needle, fear of blood transmitted diseases, etc)

○ No
○ Other: ……………

Q6-5. Additional comments about emotional factors (from anyone who are involved in ordering and sampling for blood culture; including patients and family of patients) on blood culture sampling
…………………………………………………………………

(Page break)

Finally, we have some questions about yourself

Q7-1. Which country do you currently work in?

○ Thailand
○ Vietnam
○ Indonesia
○ I do not want to answer

Province of your current hospital:……………………………………… (Dropdown list for each country)

Q7-2. Are you female or male?

○ Female
○ Male
○ Other
○ I do not want to answer

Q7-3. What is the number of beds in your hospital? (Please use the official number, and please estimate if you are uncertain.)<br><br>○ < 200<br>○ 201 - 400<br>○ 401 - 600<br>○ 601 - 1,000<br>○ 1,001 - 2,000<br>○ > 2,000<br>○ I do not know<br>○ I do not want to answer
Q7-4. In which department are you **currently working**? If your role (such as medical students) moves from one department to another department over time, please state the current department you are working in.<br>(you can select more than one answers; for example both internal medicine and infectious disease devision)<br><br>☐ Internal Medicine<br>☐ Pediatrics<br>☐ Infection disease division/department<br>☐ Surgery<br>☐ Orthopaedics<br>☐ Obstetrics / Gynaecology<br>☐ Emergency department<br>☐ Intensive care unit<br>☐ I do not want to answer<br>☐ Other: ……………
(Page break)
Q7-5. Do you want to be contacted for further studies?<br><br>○ Yes<br>○ No
Q7-6. Do you want to be informed the results of this study?<br><br>○ Yes<br>○ No
Q7-7. Your email address (If you want to be contacted via email address. Please leave it blank, if you do not want to be contact via email address)<br>……………………………………………………………….
Q7-8. Your phone number (if you want to be contacted via phone. Please leave it blank, if you do not want to be contact via phone)<br>………………………………………………………………..
Please note that a gift or cash (about $4 in value) for completing the survey is to be provided to you. Participants could receive the gift electronically if email account or telephone number is provided.

Please make sure that you click "submit" on the next page to complete the questionnaire. Otherwise, all answers that you made and your information for compensation will not be submitted to us via the system.
(Page break)
We are grateful for your participation. Thank you very much.

Appendix S4. Survey results

Questions	Indonesia (n=503)	Thailand (n=304)	Viet Nam (n=501)	P value
**Type of hospitals (Q1-1)**				
Government hospital	340 (67.6%)	209 (68.8%)	431 (86.0%)	<0.001
Private hospital	113 (22.5%)	15 (4.9%)	17 (3.4%)	
University hospital	26 (5.2%)	76 (25.0%)	29 (5.8%)	
Other[1]	19 (3.8%)	2 (0.7%)	22 (4.4%)	
I do not want to answer	5 (1.0%)	2 (0.7%)	2 (0.4%)	
**Case-study: Would you take BC sample from a hypothetical sepsis case? (Q1-3)**				
Definitely (>95-100% of the time)	157 (31.2%)	273 (89.8%)	252 (50.3%)	<0.001
Likely (75-95% of the time)	138 (27.4%)	23 (7.6%)	149 (29.7%)	
Maybe (25-74% of the time)	116 (23.1%)	5 (1.6%)	70 (14.0%)	
Unlikely (5-24% of the time)	44 (8.7%)	2 (0.7%)	19 (3.8%)	
Rarely (ranging from never <5% of the time)	46 (9.1%)	1 (0.3%)	9 (1.8%)	
I do not know	1 (0.2%)	0 (0%)	1 (0.2%)	
I do not want to answer	1 (0.2%)	0 (0%)	1 (0.2%)	
**Knowledge (TDF-1): Do you know of any guideline(s) or guideline(s) used in my hospital (Q1-4)?**				
Yes	240 (47.7%)	169 (55.6%)	347 (69.3%)	<0.001
No, my hospital does not have any	68 (13.5%)	33 (10.9%)	49 (9.8%)	
No, I do not know if my hospital uses any	183 (36.4%)	98 (32.2%)	95 (19.0%)	
I do not want to answer	12 (2.4%)	4 (1.3%)	10 (2.0%)	
**Knowledge (TDF-1): known local guideline among those who answered that they know of local guideline (Q1-5)**				
All patients presenting with SIRS	155/240 (64.6%)	147/169 (87.0%)	218/347 (62.8%)	<0.001
All patients presenting with sepsis	183/240 (76.2%)	138/169 (81.7%)	291/347 (83.9%)	0.07
All patients presenting with septic shock	147/240 (61.3%)	131/169 (77.5%)	270/347 (77.8%)	<0.001
All patients starting parenteral antibiotic treatment	92/240 (38.3%)	92/169 (54.4%)	73/347 (21.0%)	<0.001
All patients with no clinical improvement after receiving empirical antibiotics	141/240 (58.7%)	99/169 (58.6%)	160/347 (46.1%)	0.003
All patients presenting with infection and having underlying diseases	76/240 (31.7%)	61/169 (36.1%)	94/347 (27.1%)	0.10
All patients with chronic fever	97/240 (40.4%)	87/169 (51.5%)	208/347 (59.9%)	<0.001
All patients with fever of unknown origins	114/240 (47.5%)	100/169 (59.2%)	185/347 (53.3%)	0.06
All patients suspected of infections caused by atypical organisms	97/240 (40.4%)	74/169 (43.8%)	94/347 (27.1%)	<0.001
All patients suspected of infections caused by antimicrobial-resistant organisms	131/240 (54.6%)	96/169 (56.8%)	168/347 (48.4%)	0.14
All patients suspected of infections caused by multiple-drug-resistant organisms	136/240 (56.7%)	103/169 (60.9%)	194/347 (55.9%)	0.54
All patients suspected of hospital-acquired infections	116/240 (48.3%)	99/169 (58.6%)	184/347 (53.0%)	0.12
**Intention (TDF-8): How often do you plan to follow the local guideline among those who answered that they know of local guideline (Q1-6)?**				
All the time (>95-100% of the cases)	70/240 (29.2%)	76/169 (45.0%)	88/347 (25.4%)	<0.001

Questions	Indonesia (n=503)	Thailand (n=304)	Viet Nam (n=501)	P value
Often (75-95% of the cases)	102/240 (42.5%)	81/169 (47.9%)	195/347 (56.2%)	
Moderately (25-74% of the cases)	33/240 (13.8%)	11/169 (6.5%)	49/347 (14.1%)	
Occasionally (5-24% of the cases)	16/240 (6.7%)	0/169 (0%)	11/347 (3.2%)	
Rarely (ranging from never <5% of the cases)	11/240 (4.6%)	1/169 (0.6%)	2/347 (0.6%)	
I do not know	7/240 (2.9%)	0/169 (0%)	2/347 (0.6%)	
I do not want to answer	1/240 (0.4%)	0/169 (0%)	0/347 (0%)	
**Memory, attention and decision processes (TDF-10): any additional reasons for deciding to do BC among those who answered that they know of local guideline (Q1-7)?**				
No additional reasons	77/240 (32.1%)	35/169 (20.7%)	110/347 (31.7%)	0.02
Patients presenting with chills	15/240 (6.3%)	39/169 (23.1%)	23/347 (6.6%)	<0.001
Patients presenting with sepsis	102/240 (42.5%)	101/169 (59.8%)	113/347 (32.6%)	<0.001
Patients presenting with septic shock	86/240 (35.8%)	96/169 (56.8%)	139/347 (40.1%)	<0.001
Patients starting parenteral antibiotic treatment	48/240 (20.0%)	59/169 (34.9%)	35/347 (10.1%)	<0.001
Patient with no clinical improvement after receiving empirical antibiotics	102/240 (42.5%)	75/169 (44.4%)	97/347 (28.0%)	<0.001
Patients with infection and having underlying diseases	42/240 (17.5%)	36/169 (21.3%)	56/347 (16.1%)	0.35
Patients presenting with chronic fever	54/240 (22.5%)	55/169 (32.5%)	107/347 (30.8%)	0.04
Patients presenting with fever of unknown origin	72/240 (30.0%)	63/169 (37.3%)	96/347 (27.7%)	0.08
Patients suspected of infections caused by atypical organisms	52/240 (21.7%)	46/169 (27.2%)	48/347 (13.8%)	0.001
Patients suspected of infections caused by antimicrobial-resistant organisms	77/240 (32.1%)	53/169 (31.4%)	86/347 (24.8%)	0.10
Patients suspected of infections caused by multiple-drug-resistant organisms	82/240 (34.2%)	63/169 (37.3%)	92/347 (26.5%)	0.03
Patients suspected of hospital-acquired infections	77/240 (32.1%)	59/169 (34.9%)	97/347 (28.0%)	0.24
Laboratory results showing leukocytosis	29/240 (12.1%)	42/169 (24.9%)	25/347 (7.2%)	<0.001
Laboratory results showing neutropenia	36/240 (15.0%)	54/169 (32.0%)	28/347 (8.1%)	<0.001
Laboratory results showing left shift in blood count	31/240 (12.9%)	26/169 (15.4%)	14/347 (4.0%)	<0.001
Laboratory results showing CRP increase	37/240 (15.4%)	22/169 (13.0%)	42/347 (12.1%)	0.51
Laboratory results showing procalcitonin increase	55/240 (22.9%)	22/169 (13.0%)	94/347 (27.1%)	0.002
Patients can afford the cost of BC	25/240 (10.4%)	9/169 (5.3%)	32/347 (9.2%)	0.18
Patients have a health scheme or insurance that covers the cost of BC	24/240 (10.0%)	8/169 (4.7%)	26/347 (7.5%)	0.14
Patients are likely to have a final diagnosis that includes the cost of BC in the package of fee for service	18/240 (7.5%)	0/169 (0%)	25/347 (7.2%)	0.001
**Memory, attention and decision processes (TDF-10): any reasons for deciding to do BC among those who did not answer that they know of local guideline (Q1-8)?**				
Patients presenting with chills	20/263 (7.6%)	49/135 (36.3%)	29/154 (18.8%)	<0.001
Patients presenting with sepsis	188/263 (71.5%)	132/135 (97.8%)	109/154 (70.8%)	<0.001
Patients presenting with septic shock	165/263 (62.7%)	128/135 (94.8%)	135/154 (87.7%)	<0.001
Patients starting parenteral antibiotic treatment	48/263 (18.3%)	95/135 (70.4%)	26/154 (16.9%)	<0.001

Suntornsut P, et al. BMJ Open 2024; 14:e075526. doi: 10.1136/bmjopen-2023-075526

Questions	Indonesia (n=503)	Thailand (n=304)	Viet Nam (n=501)	P value
Patient with no clinical improvement after receiving empirical antibiotics	188/263 (71.5%)	119/135 (88.1%)	84/154 (54.5%)	<0.001
Patients with infection and having underlying diseases	85/263 (32.3%)	79/135 (58.5%)	52/154 (33.8%)	<0.001
Patients presenting with chronic fever	91/263 (34.6%)	89/135 (65.9%)	108/154 (70.1%)	<0.001
Patients presenting with fever of unknown origin	138/263 (52.5%)	110/135 (81.5%)	100/154 (64.9%)	<0.001
Patients suspected of infections caused by atypical organisms	123/263 (46.8%)	81/135 (60.0%)	55/154 (35.7%)	<0.001
Patients suspected of infections caused by antimicrobial-resistant organisms	177/263 (67.3%)	108/135 (80.0%)	85/154 (55.2%)	<0.001
Patients suspected of infections caused by multiple-drug-resistant organisms	183/263 (69.6%)	113/135 (83.7%)	85/354 (24.0%)	<0.001
Patients suspected of hospital-acquired infections	136/263 (51.7%)	107/135 (79.3%)	78/154 (50.6%)	<0.001
Laboratory results showing leukocytosis	41/263 (15.6%)	52/135 (38.5%)	15/154 (9.7%)	<0.001
Laboratory results showing neutropenia	34/263 (12.9%)	59/135 (43.7%)	18/154 (11.7%)	<0.001
Laboratory results showing left shift in blood count	47/263 (17.9%)	47/135 (34.8%)	16/154 (10.4%)	<0.001
Laboratory results showing CRP increase	59/263 (22.4%)	23/135 (17.0%)	26/154 (16.9%)	0.27
Laboratory results showing procalcitonin increase	73/263 (27.8%)	28/135 (20.7%)	53/154 (34.4%)	0.04
Patients can afford the cost of BC	81/263 (30.8%)	18/135 (13.3%)	32/154 (20.8%)	<0.001
Patients have a health scheme or insurance that covers the cost of BC	88/263 (33.5%)	19/135 (14.1%)	31/154 (20.1%)	<0.001
Patients are likely to have a final diagnosis that includes the cost of BC in the package of fee for service	51/263 (19.4%)	0/135 (0%)	30/154 (19.5%)	<0.001
**Knowledge (TDF-1): Do you know of any international guideline(s) or guideline(s) (Q1-9)?**				
Yes	229 (45.5%)	142 (46.7%)	225 (44.9%)	<0.001
No	263 (52.3%)	156 (51.3%)	233 (46.5%)	
I do not want to answer	11 (2.2%)	6 (2.0%)	43 (8.6%)	
**Knowledge (TDF-1): known international guideline or guideline among those who answered that they know of any international guideline(s) or guideline(s) (Q1-10)**				
BC sampling in all patients presenting with sepsis	220/229 (96.1%)	138/142 (97.2%)	208/225 (92.4%)	0.08
BC sampling in all patients starting parenteral antibiotic treatment	125/229 (54.6%)	87/142 (61.3%)	147/225 (65.3%)	<0.001
**Professional role (Q2-1): Current job**				
Medical doctor – an executive level	13 (2.6%)	5 (1.6%)	17 (3.4%)	<0.001
Medical doctor – a consultant level	74 (14.7%)	75 (24.7%)	198 (39.5%)	
Medical doctor – a general physician level	124 (24.7%)	38 (12.5%)	112 (22.4%)	
Medical doctor – a resident/registra/fellow level	168 (33.4%)	63 (20.7%)	101 (20.2%)	
Intern – recent medical school graduate	33 (6.6%)	35 (11.5%)	14 (2.8%)	
Final-year medical student	91 (18.1%)	88 (28.9%)	59 (11.8%)	
**Professional role (Q2-2): Which types of professionals/staff can order or initiate an order for a BC?**				

Questions	Indonesia (n=503)	Thailand (n=304)	Viet Nam (n=501)	P value
Medical doctor – an executive level	61 (12.1%)	163 (53.6%)	59 (11.8%)	<0.001
Medical doctor – a consultant level	431 (85.7%)	250 (82.2%)	439 (87.6%)	0.11
Medical doctor – a general physician level	265 (52.7%)	240 (78.9%)	347 (69.3%)	<0.001
Medical doctor – a resident (postgrad training) level	268 (53.3%)	242 (79.6%)	317 (63.3%)	<0.001
Intern – a recent medical school graduate level	83 (16.5%)	231 (76.0%)	118 (23.6%)	<0.001
Final-year medical student	11 (2.2%)	87 (28.6%)	3 (0.6%)	<0.001
I do not want to answer	3 (0.6%)	1 (0.3%)	11 (2.2%)	0.03
Other	0 (0%)	0 (0%)	0 (0%)	>0.99
**Knowledge (TDF-1): Do you know when and which patients should receive an order for a BC in your hospital (Q2-3)?**				
Definitely (>95-100% of the case)	65 (12.9%)	106 (34.9%)	72 (14.4%)	<0.001
Likely (75-95% of the case)	200 (39.8%)	168 (55.3%)	245 (48.9%)	
Uncertain (25-74% of the case)	148 (29.4%)	28 (9.2%)	128 (25.5%)	
Unlikely (5-24% of the case)	59 (11.7%)	0 (0%)	31 (6.2%)	
Rarely (ranging from never <5% of the case)	19 (3.8%)	0 (0%)	6 (1.2%)	
I do not know	10 (2.0%)	1 (0.3%)	8 (1.6%)	
I do not want to answer	2 (0.4%)	1 (0.3%)	11 (2.2%)	
**Social professional role and identity (TDF-3): Is it an appropriate part of your current job to order BC (Q2-4)?**				
Very appropriate	119 (23.7%)	103 (33.9%)	110 (22.0%)	<0.001
Appropriate	232 (46.1%)	166 (54.6%)	290 (57.9%)	
Uncertain	62 (12.3%)	20 (6.6%)	48 (9.6%)	
Inappropriate	21 (4.2%)	2 (0.7%)	12 (2.4%)	
Very inappropriate	2 (0.4%)	0 (0%)	0 (0%)	
I do not know	10 (2.0%)	0 (0%)	0 (0%)	
I do not want to answer	2 (0.4%)	0 (0%)	19 (3.8%)	
I cannot order BC. It is not part of my job	55 (10.9%)	13 (4.3%)	22 (4.4%)	
**Social professional role and identity (TDF-3): Would it be an appropriate part of your current job to order BC among those who answered that they cannot order for a BC (Q2-5)?**				
Very appropriate	4/55 (7.3%)	0/13 (0%)	0/22 (0%)	0.009
Appropriate	19/55 (34.5%)	8/13 (61.5%)	4/22 (18.2%)	
Uncertain	10/55 (18.2%)	4/13 (30.8%)	2/22 (9.1%)	
Inappropriate	15/55 (27.3%)	1/13 (7.7%)	8/22 (36.4%)	
Very inappropriate	3/55 (5.5%)	0/13 (0%)	2/22 (9.1%)	
I do not know	4/55 (7.3%)	0/13 (0%)	2/22 (9.1%)	
I do not want to answer	0/55 (0%)	0/13 (0%)	4/22 (18.2%)	
**Professional role (Q2-6): Which types of professionals/staff are tasked to draw blood from patients for BC?**				
Medical doctor – executive level	12 (2.4%)	44 (14.5%)	23 (4.6%)	<0.001
Medical doctor – a consultant level	60 (11.9%)	90 (29.6%)	152 (30.3%)	0.11
Medical doctor – a general physician level	72 (14.3%)	105 (34.5%)	129 (25.7%)	<0.001
Medical doctor – a resident level	96 (19.1%)	122 (40.1%)	113 (22.6%)	<0.001
Intern – recent medical school graduate	39 (7.8%)	105 (34.5%)	85 (17.0%)	<0.001
Final-year medical student	27 (5.4%)	99 (32.6%)	25 (5.0%)	<0.001
Registered nurses	342 (68.0%)	215 (70.7%)	392 (78.2%)	0.001

Questions	Indonesia (n=503)	Thailand (n=304)	Viet Nam (n=501)	P value
Microbiology laboratory team	227 (45.1%)	91 (29.9%)	151 (30.1%)	<0.001
Specialized blood draw team	197 (39.2%)	91 (29.9%)	69 (13.8%)	<0.001
I do not want to answer	3 (0.6%)	0 (0%)	2 (0.4%)	0.41
**Social professional role and identity (TDF-3): Is it an appropriate part of your current job to draw blood (Q2-7)?**				
Very appropriate	34 (6.8%)	36 (11.8%)	49 (9.8%)	0.01
Appropriate	179 (35.6%)	102 (33.6%)	179 (35.7%)	
Uncertain	109 (21.7%)	52 (17.1%)	68 (13.6%)	
Inappropriate	89 (17.7%)	46 (15.1%)	85 (17.0%)	
Very inappropriate	7 (1.4%)	6 (2.0%)	3 (0.6%)	
I do not know	8 (1.6%)	4 (1.3%)	4 (0.8%)	
I do not want to answer	4 (0.8%)	1 (0.3%)	4 (0.8%)	
It is not part of my job to draw blood	73 (14.5%)	57 (18.8%)	109 (21.8%)	
**Skill (TDF-2): How skilled are you in drawing blood excluding those whose jobs did not include drawing blood (Q2-8)?**				
Very good skill	18/430 (4.2%)	12/247 (4.9%)	32/392 (8.2%)	<0.001
Good skill	138/430 (32.1%)	46/247 (18.6%)	112/392 (28.6%)	
Fair skill	202/430 (47.0%)	118/247 (47.8%)	196/392 (50.0%)	
Poor skill	20/430 (4.7%)	52/247 (21.1%)	33/392 (8.4%)	
Very poor skill	4/430 (0.9%)	16/247 (6.5%)	1/392 (0.3%)	
I do not know	39/430 (9.1%)	3/247 (1.2%)	11/392 (2.8%)	
I do not want to answer	9/430 (2.1%)	0/247 (0%)	7/392 (1.8%)	
**Beliefs about capabilities (TDF-4): How confident that you can draw blood successfully excluding those whose jobs did not include drawing blood (Q2-9)?**				
Strongly confident	32/430 (7.4%)	20/247 (8.1%)	42/392 (10.7%)	<0.001
Confident	271/430 (63.0%)	93/247 (37.7%)	231/392 (58.9%)	
Uncertain	74/430 (17.2%)	81/247 (32.8%)	90/392 (23.0%)	
Doubtful	42/430 (9.8%)	34/247 (13.8%)	22/392 (5.6%)	
Strongly doubtful	2/430 (0.5%)	19/247 (7.7%)	6/392 (1.5%)	
I do not know	4/430 (0.9%)	0/247 (0%)	0/392 (0%)	
I do not want to answer	5/430 (1.2%)	0/247 (0%)	1/392 (0.3%)	
**Beliefs about capabilities (TDF-4): How confident that you can draw blood appropriately excluding those whose jobs did not include drawing blood (Q2-10)?**				
Strongly confident	28/430 (6.5%)	30/247 (12.1%)	37/392 (9.4%)	<0.001
Confident	262/430 (60.9%)	109/247 (44.1%)	222/392 (56.6%)	
Uncertain	86/430 (20.0%)	61/247 (24.7%)	109/392 (27.8%)	
Doubtful	44/430 (10.2%)	33/247 (13.4%)	17/392 (4.3%)	
Strongly doubtful	3/430 (0.7%)	11/247 (4.5%)	2/392 (0.5%)	
I do not know	3/430 (0.7%)	1/247 (0.4%)	1/392 (0.3%)	
I do not want to answer	4/430 (0.9%)	2/247 (0.8%)	4/392 (1.0%)	
**Beliefs about capabilities (TDF-4): Are you confident that others can draw blood successfully (Q2-11)?**				
Strongly confident	99 (19.7%)	106 (34.9%)	71 (14.2%)	<0.001
Confident	366 (72.8%)	176 (57.9%)	333 (66.5%)	
Uncertain	17 (3.4%)	14 (4.6%)	88 (17.6%)	

Questions	Indonesia (n=503)	Thailand (n=304)	Viet Nam (n=501)	P value
Doubtful	16 (3.2%)	7 (2.3%)	6 (1.2%)	
Strongly doubtful	0 (0%)	0 (0%)	1 (0.2%)	
I do not know	2 (0.4%)	1 (0.3%)	1 (0.2%)	
I do not want to answer	3 (0.6%)	0 (0%)	1 (0.2%)	
**Beliefs about capabilities (TDF-4): Are you confident that others can draw blood appropriately (Q2-12)?**				
Strongly confident	86 (17.1%)	66 (21.7%)	45 (9.0%)	<0.001
Confident	342 (68.0%)	184 (60.5%)	273 (54.5%)	
Uncertain	42 (8.3%)	45 (14.8%)	170 (33.9%)	
Doubtful	26 (5.2%)	6 (2.0%)	8 (1.6%)	
Strongly doubtful	1 (0.2%)	2 (0.7%)	2 (0.4%)	
I do not know	4 (0.8%)	1 (0.3%)	1 (0.2%)	
I do not want to answer	2 (0.4%)	0 (0%)	2 (0.4%)	
**Optimism (TDF-5): how optimistic are you that a BC will be sampled and processed in the laboratory appropriately (Q2-13)?**				
Strongly optimistic	70 (13.9%)	38 (12.5%)	31 (6.2%)	<0.001
Optimistic	332 (66.0%)	225 (74.0%)	338 (67.5%)	
Neither optimistic nor pessimistic	74 (14.7%)	31 (10.2%)	124 (24.8%)	
Pessimistic	8 (1.6%)	4 (1.3%)	4 (0.8%)	
Strongly pessimistic	5 (1.0%)	0 (0%)	1 (0.2%)	
I do not know	10 (2.0%)	5 (1.6%)	2 (0.4%)	
I do not want to answer	4 (0.8%)	1 (0.3%)	1 (0.2%)	
**Beliefs about consequence (TDF-6): BC is helpful in clinical decisions (Q3-1-1).**				
Strongly agree	204 (40.6%)	153 (50.3%)	194 (38.7%)	<0.001
Agree	279 (55.5%)	144 (47.4%)	246 (49.1%)	
Uncertain	13 (2.6%)	6 (2.0%)	47 (9.4%)	
Disagree	4 (0.8%)	1 (0.3%)	11 (2.2%)	
Strongly disagree	0 (0%)	0 (0%)	1 (0.2%)	
I do not know	2 (0.4%)	0 (0%)	0 (0%)	
I do not want to answer	1 (0.2%)	0 (0%)	2 (0.4%)	
**Beliefs about consequence (TDF-6): BC is helpful to rule in an infection (Q3-1-2).**				
Strongly agree	192 (38.2%)	123 (40.5%)	162 (32.3%)	<0.001
Agree	276 (54.9%)	159 (52.3%)	260 (51.9%)	
Uncertain	14 (2.8%)	10 (3.3%)	51 (10.2%)	
Disagree	18 (3.6%)	7 (2.3%)	24 (4.8%)	
Strongly disagree	0 (0%)	1 (0.3%)	2 (0.4%)	
I do not know	2 (0.4%)	4 (1.3%)	0 (0%)	
I do not want to answer	1 (0.2%)	0 (0%)	2 (0.4%)	
**Beliefs about consequence (TDF-6): BC is helpful to rule out an infection (Q3-1-3).**				
Strongly agree	137 (27.2%)	72 (23.7%)	59 (11.8%)	<0.001
Agree	258 (51.3%)	97 (31.9%)	163 (32.5%)	
Uncertain	44 (8.7%)	32 (10.5%)	126 (25.1%)	
Disagree	56 (11.1%)	79 (26.0%)	127 (25.3%)	
Strongly disagree	5 (1.0%)	22 (7.2%)	23 (4.6%)	
I do not know	2 (0.4%)	2 (0.7%)	0 (0%)	
I do not want to answer	1 (0.2%)	0 (0%)	3 (0.6%)	

Questions	Indonesia (n=503)	Thailand (n=304)	Viet Nam (n=501)	P value
**Beliefs about consequence (TDF-6): BC is helpful in detecting AMR infections (Q3-1-4).**				
Strongly agree	267 (53.1%)	147 (48.4%)	154 (30.7%)	<0.001
Agree	219 (43.5%)	140 (46.1%)	272 (54.3%)	
Uncertain	10 (2.0%)	11 (3.6%)	51 (10.2%)	
Disagree	4 (0.8%)	4 (1.3%)	18 (3.6%)	
Strongly disagree	0 (0%)	1 (0.3%)	4 (0.8%)	
I do not know	2 (0.4%)	1 (0.3%)	1 (0.2%)	
I do not want to answer	1 (0.2%)	0 (0%)	1 (0.2%)	
**Beliefs about consequence (TDF-6): BC is helpful in adjusting antibiotics (Q3-1-5).**				
Strongly agree	285 (56.7%)	172 (56.6%)	177 (35.3%)	<0.001
Agree	206 (41.0%)	128 (42.1%)	256 (51.1%)	
Uncertain	9 (1.8%)	2 (0.7%)	40 (8.0%)	
Disagree	0 (0%)	1 (0.3%)	21 (4.2%)	
Strongly disagree	1 (0.2%)	1 (0.3%)	3 (0.6%)	
I do not know	1 (0.2%)	0 (0%)	0 (0%)	
I do not want to answer	1 (0.2%)	0 (0%)	4 (0.8%)	
**Beliefs about consequence (TDF-6): BC can reduce overuse of antibiotics (Q3-1-6).**				
Strongly agree	241 (47.9%)	142 (46.7%)	157 (31.3%)	<0.001
Agree	220 (43.7%)	131 (43.1%)	249 (49.7%)	
Uncertain	30 (6.0%)	19 (6.3%)	59 (11.8%)	
Disagree	9 (1.8%)	11 (3.6%)	30 (6.0%)	
Strongly disagree	1 (0.2%)	1 (0.3%)	4 (0.8%)	
I do not know	1 (0.2%)	0 (0%)	0 (0%)	
I do not want to answer	1 (0.2%)	0 (0%)	2 (0.4%)	
**Beliefs about consequence (TDF-6): BC can reduce length of hospital stay (Q3-1-7).**				
Strongly agree	167 (33.2%)	101 (33.2%)	106 (21.2%)	<0.001
Agree	215 (42.7%)	122 (40.1%)	227 (45.3%)	
Uncertain	97 (19.3%)	54 (17.8%)	124 (24.8%)	
Disagree	18 (3.6%)	23 (7.6%)	39 (7.8%)	
Strongly disagree	0 (0%)	2 (0.7%)	3 (0.6%)	
I do not know	4 (0.8%)	1 (0.3%)	0 (0%)	
I do not want to answer	2 (0.4%)	1 (0.3%)	2 (0.4%)	
**Beliefs about consequence (TDF-6): BC can reduce patient mortality (Q3-1-8).**				
Strongly agree	178 (35.4%)	120 (39.5%)	124 (24.8%)	<0.001
Agree	228 (45.3%)	135 (44.4%)	242 (48.3%)	
Uncertain	79 (15.7%)	38 (12.5%)	98 (19.6%)	
Disagree	12 (2.4%)	8 (2.6%)	31 (6.2%)	
Strongly disagree	1 (0.2%)	0 (0%)	3 (0.6%)	
I do not know	4 (0.8%)	3 (1.0%)	1 (0.2%)	
I do not want to answer	1 (0.2%)	0 (0%)	2 (0.4%)	
**Beliefs about consequence (TDF-6): Accumulative results of BC are helpful in understanding epidemiology of AMR bacterial infections (Q3-1-9).**				
Strongly agree	237 (47.1%)	144 (47.4%)	193 (38.5%)	0.003
Agree	247 (49.1%)	141 (46.4%)	266 (53.1%)	
Uncertain	13 (2.6%)	16 (5.3%)	32 (6.4%)	

Questions	Indonesia (n=503)	Thailand (n=304)	Viet Nam (n=501)	P value
Disagree	0 (0%)	1 (0.3%)	7 (1.4%)	
Strongly disagree	1 (0.2%)	0 (0%)	1 (0.2%)	
I do not know	4 (0.8%)	2 (0.7%)	0 (0%)	
I do not want to answer	1 (0.2%)	0 (0%)	2 (0.4%)	
**Beliefs about consequence (TDF-6): BC is unnecessary because antibiotic therapy can be determined based on clinical presentations (Q3-3-1).**				
Strongly agree	13 (2.6%)	7 (2.3%)	18 (3.6%)	<0.001
Agree	89 (17.7%)	48 (15.8%)	53 (10.6%)	
Uncertain	154 (30.6%)	48 (15.8%)	113 (22.6%)	
Disagree	199 (39.6%)	146 (48.0%)	264 (52.7%)	
Strongly disagree	42 (8.3%)	54 (17.8%)	53 (10.6%)	
I do not know	6 (1.2%)	1 (0.3%)	0 (0%)	
I do not want to answer	0 (0%)	0 (0%)	0 (0%)	
**Beliefs about consequence (TDF-6): The therapeutic consequence of BC sampling is questionable (Q3-3-2).**				
Strongly agree	12 (2.4%)	25 (8.2%)	16 (3.2%)	<0.001
Agree	82 (16.3%)	58 (19.1%)	45 (9.0%)	
Uncertain	167 (33.2%)	60 (19.7%)	123 (24.6%)	
Disagree	191 (38.0%)	116 (38.2%)	275 (54.9%)	
Strongly disagree	34 (6.8%)	39 (12.8%)	34 (6.8%)	
I do not know	17 (3.4%)	5 (1.6%)	2 (0.4%)	
I do not want to answer	0 (0%)	1 (0.3%)	6 (1.2%)	
**Beliefs about consequence (TDF-6): The scientific basis of the guideline on BC is questionable (Q3-3-3).**				
Strongly agree	9 (1.8%)	16 (5.3%)	15 (3.0%)	<0.001
Agree	45 (8.9%)	63 (20.7%)	43 (8.6%)	
Uncertain	106 (21.1%)	58 (19.1%)	141 (28.1%)	
Disagree	248 (49.3%)	120 (39.5%)	254 (50.7%)	
Strongly disagree	79 (15.7%)	39 (12.8%)	41 (8.2%)	
I do not know	15 (3.0%)	7 (2.3%)	4 (0.8%)	
I do not want to answer	1 (0.2%)	1 (0.3%)	3 (0.6%)	
**Beliefs about consequence (TDF-6): BC is unnecessary because results are often delayed (Q3-3-4).**				
Strongly agree	15 (3.0%)	8 (2.6%)	15 (3.0%)	<0.001
Agree	113 (22.5%)	31 (10.2%)	38 (7.6%)	
Uncertain	119 (23.7%)	23 (7.6%)	82 (16.4%)	
Disagree	212 (42.1%)	161 (53.0%)	303 (60.5%)	
Strongly disagree	36 (7.2%)	80 (26.3%)	62 (12.4%)	
I do not know	8 (1.6%)	0 (0%)	0 (0%)	
I do not want to answer	0 (0%)	1 (0.3%)	1 (0.2%)	
**Beliefs about consequence (TDF-6): BC is unnecessary because results are often not interpretable (Q3-3-5).**				
Strongly agree	7 (1.4%)	4 (1.3%)	11 (2.2%)	<0.001
Agree	46 (9.1%)	18 (5.9%)	26 (5.2%)	
Uncertain	120 (23.9%)	18 (5.9%)	70 (14.0%)	
Disagree	275 (54.7%)	166 (54.6%)	326 (65.1%)	

**Questions**	**Indonesia (n=503)**	**Thailand (n=304)**	**Viet Nam (n=501)**	**P value**
Strongly disagree	47 (9.3%)	97 (31.9%)	67 (13.4%)	
I do not know	7 (1.4%)	1 (0.3%)	0 (0%)	
I do not want to answer	1 (0.2%)	0 (0%)	1 (0.2%)	
**Beliefs about consequence (TDF-6): BC is unnecessary because results are often negative or no growth (Q3-3-6).**				
Strongly agree	9 (1.8%)	6 (2.0%)	11 (2.2%)	<0.001
Agree	57 (11.3%)	26 (8.6%)	39 (7.8%)	
Uncertain	114 (22.7%)	37 (12.2%)	83 (16.6%)	
Disagree	261 (51.9%)	149 (49.0%)	312 (62.3%)	
Strongly disagree	51 (10.1%)	85 (28.0%)	55 (11.0%)	
I do not know	10 (2.0%)	1 (0.3%)	0 (0%)	
I do not want to answer	1 (0.2%)	0 (0%)	1 (0.2%)	
**Beliefs about consequence (TDF-6): BC is unnecessary because cultures are often contaminated (Q3-3-7).**				
Strongly agree	8 (1.6%)	6 (2.0%)	10 (2.0%)	<0.001
Agree	65 (12.9%)	23 (7.6%)	31 (6.2%)	
Uncertain	166 (33.0%)	44 (14.5%)	105 (21.0%)	
Disagree	212 (42.1%)	153 (50.3%)	290 (57.9%)	
Strongly disagree	39 (7.8%)	77 (25.3%)	59 (11.8%)	
I do not know	12 (2.4%)	0 (0%)	1 (0.2%)	
I do not want to answer	1 (0.2%)	1 (0.3%)	5 (1.0%)	
**Beliefs about consequence (TDF-6): BC is unnecessary because results often do not agree with clinical signs (Q3-3-8).**				
Strongly agree	8 (1.6%)	5 (1.6%)	13 (2.6%)	<0.001
Agree	46 (9.1%)	22 (7.2%)	21 (4.2%)	
Uncertain	147 (29.2%)	36 (11.8%)	84 (16.8%)	
Disagree	249 (49.5%)	158 (52.0%)	325 (64.9%)	
Strongly disagree	43 (8.5%)	83 (27.3%)	49 (9.8%)	
I do not know	10 (2.0%)	0 (0%)	0 (0%)	
I do not want to answer	0 (0%)	0 (0%)	9 (1.8%)	
**Beliefs about consequence (TDF-6): BC is unnecessary because a contaminated result often leads to wrong therapeutic approaches (Q3-3-9).**				
Strongly agree	10 (2.0%)	7 (2.3%)	14 (2.8%)	<0.001
Agree	85 (16.9%)	23 (7.6%)	38 (7.6%)	
Uncertain	128 (25.4%)	42 (13.8%)	116 (23.2%)	
Disagree	229 (45.5%)	148 (48.7%)	277 (55.3%)	
Strongly disagree	41 (8.2%)	83 (27.3%)	42 (8.4%)	
I do not know	9 (1.8%)	1 (0.3%)	3 (0.6%)	
I do not want to answer	1 (0.2%)	0 (0%)	11 (2.2%)	
**Environmental context and resources (TDF-11): BC is unnecessary because it is too expensive (Q3-3-10).**				
Strongly agree	25 (5.0%)	6 (2.0%)	12 (2.4%)	<0.001
Agree	83 (16.5%)	19 (6.3%)	24 (4.8%)	
Uncertain	114 (22.7%)	37 (12.2%)	79 (15.8%)	
Disagree	227 (45.1%)	133 (43.8%)	310 (61.9%)	
Strongly disagree	39 (7.8%)	103 (33.9%)	64 (12.8%)	

Questions	Indonesia (n=503)	Thailand (n=304)	Viet Nam (n=501)	P value
I do not know	12 (2.4%)	5 (1.6%)	2 (0.4%)	
I do not want to answer	3 (0.6%)	1 (0.3%)	10 (2.0%)	
**Beliefs about consequence (TDF-6): BC is not benefiting the patients (Q3-3-11).**				
Strongly agree	5 (1.0%)	5 (1.6%)	10 (2.0%)	<0.001
Agree	19 (3.8%)	17 (5.6%)	20 (4.0%)	
Uncertain	88 (17.5%)	13 (4.3%)	46 (9.2%)	
Disagree	290 (57.7%)	139 (45.7%)	302 (60.3%)	
Strongly disagree	92 (18.3%)	130 (42.8%)	121 (24.2%)	
I do not know	8 (1.6%)	0 (0%)	0 (0%)	
I do not want to answer	1 (0.2%)	0 (0%)	2 (0.4%)	
**Beliefs about consequence (TDF-6): It is not too late to collect BC later, particularly if patients do not improve after receiving empirical antibiotic treatment (Q3-3-12).**				
Strongly agree	23 (4.6%)	48 (15.8%)	15 (3.0%)	<0.001
Agree	116 (23.1%)	114 (37.5%)	107 (21.4%)	
Uncertain	95 (18.9%)	32 (10.5%)	89 (17.8%)	
Disagree	208 (41.4%)	65 (21.4%)	226 (45.1%)	
Strongly disagree	49 (9.7%)	45 (14.8%)	61 (12.2%)	
I do not know	11 (2.2%)	0 (0%)	3 (0.6%)	
I do not want to answer	1 (0.2%)	0 (0%)	0 (0%)	
**Beliefs about consequence (TDF-6): Quality of laboratory is questionable (Q3-3-13).**				
Strongly agree	15 (3.0%)	11 (3.6%)	9 (1.8%)	<0.001
Agree	77 (15.3%)	27 (8.9%)	55 (11.0%)	
Uncertain	147 (29.2%)	81 (26.6%)	148 (29.5%)	
Disagree	196 (39.0%)	114 (37.5%)	239 (47.7%)	
Strongly disagree	48 (9.5%)	62 (20.4%)	40 (8.0%)	
I do not know	18 (3.6%)	8 (2.6%)	5 (1.0%)	
I do not want to answer	2 (0.4%)	1 (0.3%)	5 (1.0%)	
**Beliefs about consequence (TDF-6): Levels of local antibiotic resistance are low (Q3-3-14).**				
Strongly agree	5 (1.0%)	4 (1.3%)	8 (1.6%)	<0.001
Agree	45 (8.9%)	22 (7.2%)	42 (8.4%)	
Uncertain	120 (23.9%)	63 (20.7%)	111 (22.2%)	
Disagree	225 (44.7%)	130 (42.8%)	268 (53.5%)	
Strongly disagree	87 (17.3%)	77 (25.3%)	68 (13.6%)	
I do not know	21 (4.2%)	7 (2.3%)	3 (0.6%)	
I do not want to answer	0 (0%)	1 (0.3%)	1 (0.2%)	
**Goals (TDF-9): How often do you obtain BC prior to administration of empirical antibiotics in patients presenting with sepsis (Q3-5)?**				
All the time (>95-100% of the time)	95 (18.9%)	158 (52.0%)	150 (29.9%)	<0.001
Often (75-95% of the time)	156 (31.0%)	116 (38.2%)	230 (45.9%)	
Moderately (25-74% of the time)	85 (16.9%)	21 (6.9%)	64 (12.8%)	
Occasionally (5-24% of the time)	45 (8.9%)	5 (1.6%)	12 (2.4%)	
Rarely (ranging from never <5% of the time)	82 (16.3%)	0 (0%)	19 (3.8%)	
I do not know	34 (6.8%)	4 (1.3%)	11 (2.2%)	
I do not want to answer	6 (1.2%)	0 (0%)	15 (3.0%)	

Questions	Indonesia (n=503)	Thailand (n=304)	Viet Nam (n=501)	P value
**Goals (TDF-9): How often do you obtain BC prior to administration of empirical antibiotics in patients presenting with septic shock (Q3-6)?**				
All the time (>95-100% of the time)	90 (17.9%)	234 (77.0%)	218 (43.5%)	<0.001
Often (75-95% of the time)	160 (31.8%)	59 (19.4%)	175 (34.9%)	
Moderately (25-74% of the time)	76 (15.1%)	6 (2.0%)	48 (9.6%)	
Occasionally (5-24% of the time)	48 (9.5%)	0 (0%)	18 (3.6%)	
Rarely (ranging from never <5% of the time)	84 (16.7%)	0 (0%)	20 (4.0%)	
I do not know	40 (8.0%)	3 (1.0%)	9 (1.8%)	
I do not want to answer	5 (1.0%)	2 (0.7%)	13 (2.6%)	
**Memory, attention and decision processes (TDF-10): Would you still order BC if patients are already on antibiotics (Q3-7-1)?**				
Definitely not order	11 (2.2%)	14 (4.6%)	6 (1.2%)	<0.001
Likely not order	19 (3.8%)	53 (17.4%)	28 (5.6%)	
Maybe not order	295 (58.6%)	38 (12.5%)	85 (17.0%)	
Likely to still order	143 (28.4%)	116 (38.2%)	308 (61.5%)	
Very likely to still order	18 (3.6%)	81 (26.6%)	72 (14.4%)	
I do not know	16 (3.2%)	2 (0.7%)	1 (0.2%)	
I do not want to answer	1 (0.2%)	0 (0%)	1 (0.2%)	
**Memory, attention and decision processes (TDF-10): Would you still order BC if patients have anemia (Q3-7-2)?**				
Definitely not order	16 (3.2%)	84 (27.6%)	24 (4.8%)	<0.001
Likely not order	59 (11.7%)	64 (21.1%)	33 (6.6%)	
Maybe not order	255 (50.7%)	52 (17.1%)	58 (11.6%)	
Likely to still order	124 (24.7%)	52 (17.1%)	257 (51.3%)	
Very likely to still order	20 (4.0%)	45 (14.8%)	115 (23.0%)	
I do not know	28 (5.6%)	5 (1.6%)	2 (0.4%)	
I do not want to answer	1 (0.2%)	2 (0.7%)	12 (2.4%)	
**Memory, attention and decision processes (TDF-10): Would you still order BC if blood should be used for other laboratory tests (Q3-7-3)?**				
Definitely not order	7 (1.4%)	57 (18.8%)	59 (11.8%)	<0.001
Likely not order	43 (8.5%)	57 (18.8%)	64 (12.8%)	
Maybe not order	228 (45.3%)	75 (24.7%)	117 (23.4%)	
Likely to still order	158 (31.4%)	63 (20.7%)	172 (34.3%)	
Very likely to still order	20 (4.0%)	40 (13.2%)	60 (12.0%)	
I do not know	41 (8.2%)	12 (3.9%)	21 (4.2%)	
I do not want to answer	6 (1.2%)	0 (0%)	8 (1.6%)	
**Memory, attention and decision processes (TDF-10): Would you still order BC if there are no local guidelines/guidelines for BC sampling (Q3-7-4)?**				
Definitely not order	11 (2.2%)	42 (13.8%)	42 (8.4%)	<0.001
Likely not order	41 (8.2%)	43 (14.1%)	66 (13.2%)	
Maybe not order	241 (47.9%)	95 (31.3%)	136 (27.1%)	
Likely to still order	152 (30.2%)	66 (21.7%)	174 (34.7%)	
Very likely to still order	19 (3.8%)	33 (10.9%)	41 (8.2%)	
I do not know	32 (6.4%)	24 (7.9%)	35 (7.0%)	
I do not want to answer	7 (1.4%)	1 (0.3%)	7 (1.4%)	

Questions	Indonesia (n=503)	Thailand (n=304)	Viet Nam (n=501)	P value
**Memory, attention and decision processes (TDF-10): Would you still order BC if patients do not meet certain conditions for a BC following the local guidelines (Q3-7-5)?**				
Definitely not order	28 (5.6%)	39 (12.8%)	54 (10.8%)	<0.001
Likely not order	131 (26.0%)	80 (26.3%)	93 (18.6%)	
Maybe not order	250 (49.7%)	93 (30.6%)	177 (35.3%)	
Likely to still order	58 (11.5%)	54 (17.8%)	121 (24.2%)	
Very likely to still order	11 (2.2%)	22 (7.2%)	44 (8.8%)	
I do not know	23 (4.6%)	15 (4.9%)	8 (1.6%)	
I do not want to answer	2 (0.4%)	1 (0.3%)	4 (0.8%)	
**Memory, attention and decision processes (TDF-10): Would you still order BC if patients do not have a health scheme or insurance that covers the cost of BC (Q3-7-6)?**				
Definitely not order	39 (7.8%)	7 (2.3%)	21 (4.2%)	<0.001
Likely not order	56 (11.1%)	33 (10.9%)	43 (8.6%)	
Maybe not order	306 (60.8%)	95 (31.3%)	101 (20.2%)	
Likely to still order	68 (13.5%)	87 (28.6%)	265 (52.9%)	
Very likely to still order	6 (1.2%)	63 (20.7%)	61 (12.2%)	
I do not know	23 (4.6%)	14 (4.6%)	5 (1.0%)	
I do not want to answer	5 (1.0%)	5 (1.6%)	5 (1.0%)	
**Memory, attention and decision processes (TDF-10): Would you still order BC if microbiology laboratory in your hospital is not available (Q3-7-7)?**				
Definitely not order	53 (10.5%)	21 (6.9%)	97 (19.4%)	<0.001
Likely not order	114 (22.7%)	53 (17.4%)	101 (20.2%)	
Maybe not order	229 (45.5%)	77 (25.3%)	120 (24.0%)	
Likely to still order	74 (14.7%)	79 (26.0%)	109 (21.8%)	
Very likely to still order	10 (2.0%)	54 (17.8%)	36 (7.2%)	
I do not know	19 (3.8%)	12 (3.9%)	30 (6.0%)	
I do not want to answer	4 (0.8%)	8 (2.6%)	8 (1.6%)	
**Environmental context and resources (TDF-11): How often could you not order BC because consumables are not available (Q4-1)?**				
All the time (>95-100% of the time)	24 (4.8%)	12 (3.9%)	19 (3.8%)	<0.001
Often (75-95% of the time)	61 (12.1%)	15 (4.9%)	19 (3.8%)	
Moderately (25-74% of the time)	52 (10.3%)	11 (3.6%)	56 (11.2%)	
Occasionally (5-24% of the time)	86 (17.1%)	15 (4.9%)	51 (10.2%)	
Rarely (ranging from never <5% of the time)	219 (43.5%)	232 (76.3%)	309 (61.7%)	
I do not know	53 (10.5%)	18 (5.9%)	25 (5.0%)	
I do not want to answer	8 (1.6%)	1 (0.3%)	22 (4.4%)	
**Environmental context and resources (TDF-11): How often could you not order BC because the microbiology laboratory is not available or not functioning (Q4-2)?**				
All the time (>95-100% of the time)	34 (6.8%)	9 (3.0%)	15 (3.0%)	<0.001
Often (75-95% of the time)	58 (11.5%)	13 (4.3%)	28 (5.6%)	
Moderately (25-74% of the time)	48 (9.5%)	9 (3.0%)	37 (7.4%)	
Occasionally (5-24% of the time)	78 (15.5%)	14 (4.6%)	27 (5.4%)	
Rarely (ranging from never <5% of the time)	224 (44.5%)	238 (78.3%)	342 (68.3%)	

Questions	Indonesia (n=503)	Thailand (n=304)	Viet Nam (n=501)	P value
I do not know	56 (11.1%)	21 (6.9%)	28 (5.6%)	
I do not want to answer	5 (1.0%)	0 (0%)	24 (4.8%)	
**Environmental context and resources (TDF-11): How often do patients have to pay for BC using their own money (i.e. out of pocket) (Q4-3)?**				
All the time (>95-100% of the time)	26 (5.2%)	11 (3.6%)	6 (1.2%)	<0.001
Often (75-95% of the time)	52 (10.3%)	17 (5.6%)	28 (5.6%)	
Moderately (25-74% of the time)	50 (9.9%)	19 (6.3%)	67 (13.4%)	
Occasionally (5-24% of the time)	69 (13.7%)	48 (15.8%)	134 (26.7%)	
Rarely (ranging from never <5% of the time)	138 (27.4%)	135 (44.4%)	173 (34.5%)	
I do not know	163 (32.4%)	73 (24.0%)	72 (14.4%)	
I do not want to answer	5 (1.0%)	1 (0.3%)	21 (4.2%)	
**Environmental context and resources (TDF-11): Would you say that the benefits of BC outweigh the cost (Q4-4)?**				
Very likely	101 (20.1%)	135 (44.4%)	184 (36.7%)	<0.001
Likely	210 (41.7%)	97 (31.9%)	223 (44.5%)	
Uncertain	93 (18.5%)	37 (12.2%)	34 (6.8%)	
Unlikely	45 (8.9%)	10 (3.3%)	16 (3.2%)	
Very unlikely	3 (0.6%)	13 (4.3%)	17 (3.4%)	
I do not know	49 (9.7%)	12 (3.9%)	17 (3.4%)	
I do not want to answer	2 (0.4%)	0 (0%)	10 (2.0%)	
**Reinforcement (TDF-7): Are there any positive consequences if you order a BC when recommended (Q5-1)?**				
No	283 (56.3%)	187 (61.5%)	206 (41.1%)	<0.001
Yes, social	31 (6.2%)	37 (12.2%)	59 (11.8%)	
Yes, material	4 (0.8%)	2 (0.7%)	8 (1.6%)	
Yes, both social and material	33 (6.6%)	18 (5.9%)	103 (20.6%)	
I do not know	143 (28.4%)	58 (19.1%)	75 (15.0%)	
I do not want to answer	8 (1.6%)	1 (0.3%)	45 (9.0%)	
Other	1 (0.2%)	1 (0.3%)	5 (1.0%)	
**Reinforcement (TDF-7): Are there any negative consequences if you do not order a BC when recommended (Q5-2)?**				
No	248 (49.3%)	101 (33.2%)	134 (26.7%)	<0.001
Yes, social	65 (12.9%)	115 (37.8%)	100 (20.0%)	
Yes, material	8 (1.6%)	4 (1.3%)	13 (2.6%)	
Yes, both social and material	27 (5.4%)	22 (7.2%)	111 (22.2%)	
I do not know	142 (28.2%)	60 (19.7%)	83 (16.6%)	
I do not want to answer	12 (2.4%)	2 (0.7%)	55 (11.0%)	
Other	1 (0.2%)	0 (0%)	5 (1.0%)	
**Reinforcement (TDF-7): Are there any negative consequences if you order a BC when recommended (Q5-3)?**				
No	251 (49.9%)	162 (53.3%)	210 (41.9%)	<0.001
Yes, social	47 (9.3%)	43 (14.1%)	31 (6.2%)	
Yes, material	10 (2.0%)	3 (1.0%)	31 (6.2%)	
Yes, both social and material	30 (6.0%)	14 (4.6%)	91 (18.2%)	
I do not know	150 (29.8%)	78 (25.7%)	83 (16.6%)	
I do not want to answer	14 (2.8%)	4 (1.3%)	53 (10.6%)	

Questions	Indonesia (n=503)	Thailand (n=304)	Viet Nam (n=501)	P value
Other	1 (0.2%)	0 (0%)	2 (0.4%)	
**Behaviour regulation (TDF-14): Any training, lectures, classes or meetings that provide you knowledge about local/national/international guidelines for BC sampling (Q5-5)?**				
No	153 (30.4%)	64 (21.1%)	52 (10.4%)	<0.001
Yes, infrequent (less than once a year)	90 (17.9%)	87 (28.6%)	111 (22.2%)	
Yes, occasionally (at least once a year)	109 (21.7%)	84 (27.6%)	196 (39.1%)	
Yes, regularly	53 (10.5%)	22 (7.2%)	61 (12.2%)	
I do not know	91 (18.1%)	46 (15.1%)	74 (14.8%)	
I do not want to answer	5 (1.0%)	1 (0.3%)	6 (1.2%)	
Other	2 (0.4%)	0 (0%)	1 (0.2%)	
**Behaviour regulation (TDF-14): any procedures that support you or doctors to order or regulate ordering of BC per local/national/international guidelines (Q5-6)?**				
No	129 (25.7%)	71 (23.4%)	76 (15.2%)	<0.001
Poster	57 (11.3%)	40 (13.2%)	66 (13.2%)	0.62
Standard order form	120 (23.9%)	90 (29.6%)	107 (21.4%)	0.03
Computer system to remind ordering BC	25 (5.0%)	14 (4.6%)	74 (14.8%)	<0.001
case review (e.g. grand round; morning ward round, clinical meetings, and BC is often mentioned)	76 (15.1%)	86 (28.3%)	164 (32.7%)	<0.001
Stewardship programme and reviewing BC is included in the programme	61 (12.1%)	25 (8.2%)	121 (24.2%)	<0.001
Local hospital guideline (e.g. standard operating procedure [SOP])	113 (22.5%)	77 (25.3%)	162 (32.3%)	0.002
I do not know	107 (21.3%)	49 (16.1%)	66 (13.2%)	0.003
I do not want to answer	9 (1.8%)	2 (0.7%)	15 (3.0%)	0.07
**Social influence (TDF-12): To what extent do you order BC because you are following local norms (Q6-1)?**				
All the time (>95-100% of the time)	50 (9.9%)	67 (22.0%)	64 (12.8%)	<0.001
Often (75-95% of the time)	130 (25.8%)	166 (54.6%)	174 (34.7%)	
Moderately (25-74% of the time)	84 (16.7%)	41 (13.5%)	144 (28.7%)	
Occasionally (5-24% of the time)	67 (13.3%)	15 (4.9%)	40 (8.0%)	
Rarely (ranging from never <5% of the time)	80 (15.9%)	8 (2.6%)	40 (8.0%)	
I do not know	87 (17.3%)	7 (2.3%)	25 (5.0%)	
I do not want to answer	5 (1.0%)	0 (0%)	14 (2.8%)	
**Social influence (TDF-12): Influence from nurses (Q6-2-1)? Positive influence could mean facilitate, support or encourage BC sampling. Negative influence could mean hinder or discourage BC sampling.**				
Very positive influence	46 (9.1%)	29 (9.5%)	60 (12.0%)	<0.001
Positive influence	230 (45.7%)	103 (33.9%)	154 (30.7%)	
Neither positive nor negative influence	162 (32.2%)	122 (40.1%)	228 (45.5%)	
Negative influence	15 (3.0%)	26 (8.6%)	25 (5.0%)	
Very negative influence	1 (0.2%)	1 (0.3%)	0 (0%)	
I do not know	45 (8.9%)	19 (6.3%)	30 (6.0%)	
I do not want to answer	4 (0.8%)	4 (1.3%)	4 (0.8%)	

**Questions**	**Indonesia (n=503)**	**Thailand (n=304)**	**Viet Nam (n=501)**	**P value**
**Social influence (TDF-12): Influence from final-year medical students (Q6-2-2)?**				
Very positive influence	29 (5.8%)	22 (7.2%)	30 (6.0%)	0.004
Positive influence	155 (30.8%)	87 (28.6%)	104 (20.8%)	
Neither positive nor negative influence	249 (49.5%)	157 (51.6%)	315 (62.9%)	
Negative influence	4 (0.8%)	3 (1.0%)	6 (1.2%)	
Very negative influence	1 (0.2%)	1 (0.3%)	0 (0%)	
I do not know	60 (11.9%)	27 (8.9%)	42 (8.4%)	
I do not want to answer	5 (1.0%)	7 (2.3%)	4 (0.8%)	
**Social influence (TDF-12): Influence from Interns (Q6-2-3)?**				
Very positive influence	31 (6.2%)	41 (13.5%)	33 (6.6%)	<0.001
Positive influence	182 (36.2%)	134 (44.1%)	170 (33.9%)	
Neither positive nor negative influence	205 (40.8%)	96 (31.6%)	251 (50.1%)	
Negative influence	5 (1.0%)	4 (1.3%)	3 (0.6%)	
Very negative influence	1 (0.2%)	0 (0%)	1 (0.2%)	
I do not know	70 (13.9%)	24 (7.9%)	38 (7.6%)	
I do not want to answer	9 (1.8%)	5 (1.6%)	5 (1.0%)	
**Social influence (TDF-12): Influence from residents (Q6-2-4)?**				
Very positive influence	64 (12.7%)	73 (24.0%)	79 (15.8%)	<0.001
Positive influence	270 (53.7%)	138 (45.4%)	219 (43.7%)	
Neither positive nor negative influence	109 (21.7%)	63 (20.7%)	161 (32.1%)	
Negative influence	2 (0.4%)	3 (1.0%)	1 (0.2%)	
Very negative influence	0 (0%)	0 (0%)	1 (0.2%)	
I do not know	51 (10.1%)	23 (7.6%)	37 (7.4%)	
I do not want to answer	7 (1.4%)	4 (1.3%)	3 (0.6%)	
**Social influence (TDF-12): Influence from doctors (Q6-2-5)?**				
Very positive influence	82 (16.3%)	62 (20.4%)	67 (13.4%)	<0.001
Positive influence	293 (58.3%)	125 (41.1%)	216 (43.1%)	
Neither positive nor negative influence	90 (17.9%)	85 (28.0%)	188 (37.5%)	
Negative influence	6 (1.2%)	3 (1.0%)	3 (0.6%)	
Very negative influence	0 (0%)	3 (1.0%)	1 (0.2%)	
I do not know	29 (5.8%)	23 (7.6%)	15 (3.0%)	
I do not want to answer	3 (0.6%)	3 (1.0%)	11 (2.2%)	
**Social influence (TDF-12): Influence from consultants (Q6-2-6)?**				
Very positive influence	172 (34.2%)	117 (38.5%)	109 (21.8%)	<0.001
Positive influence	255 (50.7%)	125 (41.1%)	261 (52.1%)	
Neither positive nor negative influence	38 (7.6%)	41 (13.5%)	113 (22.6%)	
Negative influence	5 (1.0%)	4 (1.3%)	4 (0.8%)	
Very negative influence	1 (0.2%)	2 (0.7%)	0 (0%)	
I do not know	26 (5.2%)	11 (3.6%)	13 (2.6%)	
I do not want to answer	6 (1.2%)	4 (1.3%)	1 (0.2%)	
**Social influence (TDF-12): Influence from head of department (Q6-2-7)?**				
Very positive influence	81 (16.1%)	51 (16.8%)	135 (26.9%)	<0.001
Positive influence	254 (50.5%)	89 (29.3%)	252 (50.3%)	
Neither positive nor negative influence	104 (20.7%)	119 (39.1%)	95 (19.0%)	
Negative influence	10 (2.0%)	6 (2.0%)	6 (1.2%)	
Very negative influence	0 (0%)	1 (0.3%)	0 (0%)	

Questions	Indonesia (n=503)	Thailand (n=304)	Viet Nam (n=501)	P value
I do not know	48 (9.5%)	34 (11.2%)	11 (2.2%)	
I do not want to answer	6 (1.2%)	4 (1.3%)	2 (0.4%)	
**Social influence (TDF-12): Influence from executive or administrative level of the hospital (Q6-2-8)?**				
Very positive influence	55 (10.9%)	35 (11.5%)	101 (20.2%)	<0.001
Positive influence	188 (37.4%)	67 (22.0%)	216 (43.1%)	
Neither positive nor negative influence	169 (33.6%)	145 (47.7%)	154 (30.7%)	
Negative influence	21 (4.2%)	8 (2.6%)	7 (1.4%)	
Very negative influence	8 (1.6%)	2 (0.7%)	1 (0.2%)	
I do not know	57 (11.3%)	42 (13.8%)	19 (3.8%)	
I do not want to answer	5 (1.0%)	5 (1.6%)	3 (0.6%)	
**Social influence (TDF-12): Influence from patients (Q6-2-9)?**				
Very positive influence	43 (8.5%)	44 (14.5%)	57 (11.4%)	<0.001
Positive influence	197 (39.2%)	74 (24.3%)	148 (29.5%)	
Neither positive nor negative influence	197 (39.2%)	141 (46.4%)	250 (49.9%)	
Negative influence	18 (3.6%)	14 (4.6%)	21 (4.2%)	
Very negative influence	1 (0.2%)	1 (0.3%)	1 (0.2%)	
I do not know	44 (8.7%)	26 (8.6%)	20 (4.0%)	
I do not want to answer	3 (0.6%)	4 (1.3%)	4 (0.8%)	
**Social influence (TDF-12): Influence from family of patients (Q6-2-10)?**				
Very positive influence	32 (6.4%)	21 (6.9%)	34 (6.8%)	<0.001
Positive influence	171 (34.0%)	40 (13.2%)	119 (23.8%)	
Neither positive nor negative influence	221 (43.9%)	186 (61.2%)	282 (56.3%)	
Negative influence	23 (4.6%)	20 (6.6%)	39 (7.8%)	
Very negative influence	3 (0.6%)	2 (0.7%)	2 (0.4%)	
I do not know	50 (9.9%)	30 (9.9%)	19 (3.8%)	
I do not want to answer	3 (0.6%)	5 (1.6%)	6 (1.2%)	
**Emotions (TDF-13): Any emotional factors (Q6-4)?**				
Yes	51 (10.1%)	10 (3.3%)	32 (6.4%)	0.001
**Gender (Q7-2)**				
Female	263 (52.3%)	195 (64.1%)	222 (44.3%)	<0.001
Male	236 (46.9%)	106 (34.9%)	263 (52.5%)	
Other	1 (0.2%)	0 (0%)	0 (0%)	
I do not want to answer	3 (0.6%)	3 (1.0%)	16 (3.2%)	
**Hospital bed size (Q7-3)**				
<200	99 (19.7%)	35 (11.5%)	24 (4.8%)	<0.001
201-400	107 (21.3%)	46 (15.1%)	29 (5.8%)	
401-600	72 (14.3%)	39 (12.8%)	62 (12.4%)	
601-1,000	66 (13.1%)	45 (14.8%)	144 (28.7%)	
1,001-2,000	39 (7.8%)	82 (27.0%)	125 (25.0%)	
> 2,000	27 (5.4%)	30 (9.9%)	74 (14.8%)	
I do not know	89 (17.7%)	27 (8.9%)	35 (7.0%)	
I do not want to answer	4 (0.8%)	0 (0%)	8 (1.6%)	
**Department (Q7-4)**				
Internal medicine	149 (29.6%)	155 (51.0%)	146 (29.1%)	<0.001
Pediatrics	65 (12.9%)	43 (14.1%)	45 (9.0%)	0.05
Infection disease division/department	12 (2.4%)	5 (1.6%)	56 (11.2%)	<0.001
Surgery	21 (4.2%)	45 (14.8%)	81 (16.2%)	<0.001

**Questions**	**Indonesia (n=503)**	**Thailand (n=304)**	**Viet Nam (n=501)**	**P value**
Orthopaedics	6 (1.2%)	18 (5.9%)	14 (2.8%)	0.001
Obstetrics / Gynaecology	20 (4.0%)	29 (9.5%)	7 (1.4%)	<0.001
Emergency department	112 (22.3%)	34 (11.2%)	29 (5.8%)	<0.001
Intensive care unit	45 (8.9%)	13 (4.3%)	51 (10.2%)	0.01
I do not want to answer	24 (4.8%)	25 (8.2%)	52 (10.4%)	0.004
Other	137 (27.2%)	29 (9.5%)	58 (11.6%)	<0.001

Gray color represents questions that were asked to subsets of participants. [1] Included primary health care, clinic, retired and answers as role of doctors (including residents, interns and medical students).

Appendix S5: Sample quotes

TDF domains	Themes	Sample quotes
Goal	Priority of BC	• "If other urgent examinations are to be required, BC could be delayed." (Vietnamese respondent [barrier]).<br>• "Early blood cultures should be encouraged for patients presenting with infection before antibiotics are given" (Vietnamese respondent [enabler])<br>• "BC should be performed, although the results are often negative. We can't wait for patients not responding to empirical antibiotics before starting BC. It could lead to a prolonged hospital stay" (Indonesian respondent [enabler])
Social professional role and identity	Level of doctors who can order or initiate an order for BC	• "Medical students can order BC; however, medical students must have a signature of a supervising medical doctor together all the time." (Thai respondent [enabler])<br>• "Medical doctors in charge hold the decisions of ordering BC. However, residents (medical doctors who are currently under postgraduate clinical doctors) could report (to medical doctors in charge) which patients need BC." (Indonesian respondent [barrier])
	Perception about their role to order or initiate an order for BC.	
	Perception about their role to draw blood for BC	
Belief about consequences	Perception that BC is helpful	• "(BC is helpful because) immediate use of BC and prior to giving antibiotics can inform whether a patient has bacteraemia or not, what organism is the cause, and which antibiotic would be appropriate." (Thai respondent [enabler])<br>• "(BC is helpful because) BC shortens the time to find the agent and shortens the treatment time for the patient" (Vietnamese respondent)<br>• "(BC is helpful because) BC can reduce irrational antibiotic prescriptions." (Indonesian respondent)
	Perception that BC is unnecessary	• "(BC is unnecessary because) BC often requires a long time to generate the results. Hence, the patient's condition has improved with empirical antibiotics when BC results are generated." (Indonesian respondent [barrier])<br>• "(BC is unnecessary because) laboratory often causes contamination, making the result irrelevant to clinical signs." (Thai respondent [barrier])<br>• "(BC is unnecessary because) most patients have self-medication with antibiotics at home, so BC often yields undesirable results." (Vietnamese respondent [barrier])<br>• "(BC is unnecessary because) time to return results is slow and most of them do not find pathogenic bacteria." (Vietnamese respondent [barrier])

**TDF domains**	**Themes**	**Sample quotes**
		• "BCs are not useful when the focal point of the infection is clear and the patient responds well to treatment." (Vietnamese respondent [barrier])
Intention	Intention to follow guidelines	• A guideline on BC examination should be written in detail, reviewed multiple times, monitored and followed with the appropriate rewards and punishment. (Vietnamese respondent [enabler]
Knowledge	Awareness of guidelines	
	Training	• "I have not learnt about the local recommendation for BC sampling in my university hospital." (Indonesian respondent [barrier]).<br>• "BC has not been highlighted in the clinics when I have Bed Side Teaching, Case Review, Tutorials, etc. It is recommended to do as ideal as is written in the literature." (Indonesian respondent [barrier])
Social influence	Norms of BC sampling	• "Social factors could influence diagnosis and therapy." (Indonesian respondent [barrier/enabler])
	Influences from healthcare workers, patients and family of patients	• "The patient's families often have a strong influence on patients. They often decide not to provide consent to BC." (Indonesian respondent [barrier])<br>• "Negative influence in the order of BC is cost. Supervisor or the executives (of the hospitals) gave an order to control the cost." (Thai respondent [barrier])<br>• "The patient's relatives are not satisfied with the cost of (BC) testing." (Vietnamese respondent [barrier]).<br>• "Because people do not understand, when ordering BC, they often complain." (Vietnamese respondent [barrier])<br>• "Some patients think that physicians and other healthcare workers only perform BC examinations for money." (Indonesian respondent [barrier]).<br>• "Sometimes, when the blood puncture fails on the first try, patients and their families refuse to have more blood drawn." (Indonesian respondent [barrier]).
Reinforcement	Consequences that discourage BC sampling	• "Warnings are given due to the costly examination, especially for patients insured with the Healthcare and Social Security Agency." (Indonesian respondent [barrier])<br>• "Sometimes, the cost of BC cannot be reimbursed, and the doctor has to pay." (Vietnamese respondent [barrier])<br>• "Occasionally, the insurance assessment agency often asks questions, requires explanations and can make it difficult to limit the order of BC for patients." (Vietnamese respondent [barrier])
	Consequences that encourage BC sampling	• "The consequences are usually minimal. The hospital prioritizes the clinical improvement and satisfaction of the patients and their families instead of conducting according to the guidelines or minimizing antibiotic resistance." (Vietnamese respondent [barrier])<br>• "If the patient dies without BC testing, it will be questioned in the death case report." (Indonesian respondent [enabler])<br>• "If (we) do not follow the recommendation for (BC) diagnostic tests, there will be a verbal reprimand in order

TDF domains	Themes	Sample quotes
		to make sure that the care is up to the standard." (Thai respondent [enabler])<br>• "There are no incentives, rewards or penalties." (Vietnamese respondent [lack of enabler])<br>• "The case of septic shock without a BC will be reprimanded." (Vietnamese respondent [enabler])
Behavioural regulation	Regulations on cost reimbursement	• "National insurance coverage and hospital regulation could inhibit BC examination." (Indonesian respondent [barrier])<br>• "The insurance often disapproves of BC examination. It is only approved when patients are admitted to the ICU or HCU [High Care Unit]." (Indonesian respondent [barrier])<br>• "It is affected by the insurance. Healthcare and Social Security Agency in Indonesia only covers septic patients around two million rupiahs/patient [about 138 US$], it is not sufficient to cover the resources required, including BC examinations." (Indonesian respondent [barrier].<br>• "Some hospitals allow only three laboratory tests; therefore, (doctors) must select laboratory tests for patients." (Thai respondent [barrier])<br>• "When the final diagnosis does not match, (the cost of BC) will not be paid by Health Insurance." (Vietnamese respondent [barrier])<br>• "Medical professionals often object to BC due to tiredness [disheartened feeling] and the consequence of reduced reimbursement." (Vietnamese respondent [barrier])<br>• "It is difficult (to order BC) because there are restrictions from the financial coverage on the Healthcare and Social Security Agency." (Indonesian respondent)
	Procedures to support or regulate doctors to order BC	•
Environmental context and resources	Perceived cost-effectiveness of BC	• "BC is still not cost-effective for my hospital" (Indonesian respondent [barrier]).<br>• "BC is not cost-effective" (Vietnamese respondent [barrier])
	Availability of microbiology laboratories, transport modalities, resources and consumables	• "Hospitals that do not have a microbiology laboratory cannot obtain culture results. If you still want to take BC, you have to send it to another hospital, it will cost the patient more" (Vietnamese respondent [barrier])
	Out-of-pocket	• "BC is essential, but it costs a lot (Indonesia Rp 750.000,00 [about 52US$]), and many patients could not afford it." (Indonesian respondent [barrier])<br>• "Patients usually refuse BC due to the cost." (Indonesian respondent [barrier])
Emotion	Fear or anxiety of healthcare providers	• "In some patients with blood-borne infectious diseases, doctors are afraid to draw blood." (Vietnamese respondent [barrier])<br>• "Nurses are afraid to draw a lot of blood." (Vietnamese respondent [barrier])

**TDF domains**	**Themes**	**Sample quotes**
	Fear or anxiety of patients or families of patients	• "Patient and their families are afraid of contracting blood-transmitted diseases." (Indonesian respondent [barrier])<br>• "Patient are afraid to be drawn a lot of blood." (Vietnamese respondent [barrier])<br>• "Fear of pain. Fear of needle" (Thai respondent [barrier]<br>• "Anxiety, panic or uncooperative attitude." (Vietnamese respondent [barrier])<br>• "Patients are afraid that taking a lot of blood will cause anemia." (Vietnamese respondent [barrier])
Optimism	Confidence that BC will be appropriately sampled and processed in the laboratory	
Skill	Skill in drawing blood for BC	
Memory, attention and decision processes	Patients who are already on antibiotics or have anemia	"In patients who have already received antibiotics, BC is not meaningful." (Vietnamese respondent [barrier]
	Clinical presentations for deciding to order BC	"Patients who are receiving palliative-care may not be tested for BC, even though there are criteria for it" (Thai respondent [barrier])
Beliefs about capabilities	Belief in their own capability to draw blood	
	Belief in capability of those who are tasked to draw blood	

Suntornsut P, *et al. BMJ Open* 2024; 14:e075526. doi: 10.1136/bmjopen-2023-075526

Appendix S6. Associations between barriers/enablers and the responses that they would definitely take BC in the case scenario

Barriers or enablers	Indonesia[1] (n=503)	Thailand[1] (n=304)	Viet Nam[1] (n=501)	Odds ratio[2]	P value
**TDF domain: Goals**					
**How often do you obtain BC prior to receiving empirical antibiotic in patients presenting with sepsis?**					
All the time / Often (>75-100% of the time)	45.4% (113/249)	91.6% (251/274)	58.6% (222/379)	4.25 (3.04-5.94)	<0.001
Moderately / Occasionally / Rarely (0-74% of the time)	15.6% (33/212)	73.1% (19/26)	22.1% (21/95)	1.0	
**How often do you obtain BC prior to receiving empirical antibiotic in patients presenting with septic shock?**					
All the time / Often (>75-100% of the time)	44.8% (111/248)	90.1% (264/293)	56.4% (221/392)	3.71 (2.61-5.27)	<0.001
Moderately / Occasionally / Rarely (0-74% of the time)	15.4% (32/208)	83.3% (5/6)	25.6% (22/86)	1.0	
**TDF domain: Social professional role and identity**					
**Current job**					
Medical doctor – an executive level	15.4% (2/13)	60.0% (2/3)	35.3% (6/17)	0.20 (0.09-0.47)	<0.001
Medical doctor – a consultant level	34.4% (25/73)	90.7% (68/75)	49.2% (97/197)	0.48 (0.33-0.69)	
Medical doctor – a general physician level	10.5% (13/124)	81.6% (31/38)	46.0% (51/111)	0.27 (0.18-0.40)	
Medical doctor – a resident/registra/fellow level	48.8% (82/168)	93.7% (59/63)	68.3% (69/101)	1.0	
Intern – recent medical school graduate	12.1% (4/33)	88.6% (31/35)	35.7% (5/14)	0.26 (0.14-0.49)	
Final-year medical student	34.4% (31/90)	92.1% (81/88)	40.7% (24/59)	0.50 (0.33-0.76)	
**Perception about their role to order or initiate an order for BC**					
Very appropriate / Appropriate	45.5% (120/264)	91.2% (250/274)	61.2% (195/317)	3.36 (2.50-4.51)	<0.001
Uncertain / Inappropriate / Very inappropriate	16% (36/225)	78.6% (22/28)	33.3% (55/165)	1.0	
**Perception about their role to draw blood for BC[3]**					
Very appropriate / Appropriate	38.0% (27/71)	87.8% (65/74)	52.4% (54/103)	1.94 (1.04-3.64)	0.04
Uncertain / Inappropriate / Very inappropriate	28.6% (4/14)	94.8% (55/58)	25.6% (10/39)	1.0	
**TDF domain: Belief about consequences**					
**BC is helpful in clinical decision**					
Strongly agree / Agree	31.5% (152/482)	89.9% (267/297)	54.1% (237/438)	2.96 (1.71-5.12)	<0.001
Uncertain / Disagree / Strongly disagree	23.5% (4/17)	85.7% (6/7)	23.7% (14/59)	1.0	
**BC is helpful to rule in an infection**					
Strongly agree / Agree	31.9% (149/467)	90.1% (254/282)	52.4% (220/420)	1.58 (1.04-2.39)	0.03
Uncertain / Disagree / Strongly disagree	21.9% (7/32)	100% (18/18)	40.3% (31/77)	1.0	
**BC is helpful to rule out an infection**					
Strongly agree / Agree	31.2% (123/394)	88.2% (149/169)	47.7% (105/220)	0.91 (0.69-1.19)	0.49
Uncertain / Disagree / Strongly disagree	31.4% (33/105)	91.7% (122/133)	52.9% (146/276)	1.0	

Barriers or enablers	Indonesia[1] (n=503)	Thailand[1] (n=304)	Viet Nam[1] (n=501)	Odds ratio[2]	P value
**BC is helpful to detecting AMR bacterial infections**					
Strongly agree / Agree	31.3% (152/485)	89.2% (256/287)	51.2% (217/424)	1.26 (0.80-1.98)	0.32
Uncertain / Disagree / Strongly disagree	28.6% (4/14)	100% (16/16)	45.2% (33/73)	1.0	
**BC is helpful in adjusting antibiotics**					
Strongly agree / Agree	31.0% (152/490)	89.7% (269/300)	52.2% (225/431)	1.50 (0.90-2.50)	0.12
Uncertain / Disagree / Strongly disagree	44.4% (4/9)	100% (4/4)	39.1% (25/64)	1.0	
**BC can reduce overuse of antibiotics**					
Strongly agree / Agree	30.7% (141/460)	89.0% (243/273)	52.2% (211/404)	1.08 (0.74-1.58)	0.68
Uncertain / Disagree / Strongly disagree	38.5% (15/39)	97% (30/31)	42.0% (40/93)	1.0	
**BC can reduce length of hospital stay**					
Strongly agree / Agree	31.5% (120/381)	91.5% (204/223)	55.3% (183/331)	1.53 (1.14-2.04)	0.004
Uncertain / Disagree / Strongly disagree	29.6% (34/115)	86.1% (68/79)	41.0% (68/166)	1.0	
**BC can reduce patient mortality**					
Strongly agree / Agree	32.8% (133/405)	89.0% (227/255)	55.0% (200/364)	1.61 (1.18-2.20)	0.003
Uncertain / Disagree / Strongly disagree	23.9% (22/92)	95.7% (44/46)	38.6% (51/132)	1.0	
**Accumulative results of BC are helpful in understanding epidemiology of AMR bacterial infections**					
Strongly agree / Agree	31.5% (152/483)	90.5% (258/285)	52.5% (240/457)	2.89 (1.60-5.19)	<0.001
Uncertain / Disagree / Strongly disagree	21.4% (3/14)	76.5% (13/17)	25% (10/40)	1.0	
**BC is unnecessary because antibiotic therapy can be determined based on clinical presentation**					
Strongly agree / Agree	20.8% (21/101)	83.6% (46/44)	33.8% (24/71)	0.51 (0.36-0.73)	<0.001
Uncertain / Disagree / Strongly disagree	33.9% (134/395)	91.1% (226/248)	53.3% (228/428)	1.0	
**The therapeutic consequence of BC is questionable**					
Strongly agree / Agree	32.3% (30/93)	88.0% (73/83)	41.0% (25/61)	0.84 (0.59-1.19)	0.32
Uncertain / Disagree / Strongly disagree	30.6% (120/392)	91.2% (196/215)	51.9% (223/430)	1.0	
**The scientific basis of the guideline on BC is questionable**					
Strongly agree / Agree	32.0% (17/53)	87.3% (69/79)	32.8% (19/58)	0.66 (0.45-0.98)	0.04
Uncertain / Disagree / Strongly disagree	30.4% (132/433)	91.2% (198/217)	53.2% (231/434)	1.0	
**BC is unnecessary because results are often delayed**					
Strongly agree / Agree	18.9% (24/127)	82.1% (32/39)	30.2% (16/53)	0.48 (0.33-0.69)	<0.001
Uncertain / Disagree / Strongly disagree	35.2% (129/367)	90.9% (240/264)	53.0% (236/445)	1.0	
**BC is unnecessary because results are often not interpretable**					
Strongly agree / Agree	25.0% (13/52)	77.3% (17/22)	29.7% (11/37)	0.54 (0.34-0.87)	0.01
Uncertain / Disagree / Strongly disagree	31.7% (140/442)	90.8% (255/281)	52.3% (241/461)	1.0	
**BC is unnecessary because results are often negative or no growth**					

Barriers or enablers	Indonesia[1] (n=503)	Thailand[1] (n=304)	Viet Nam[1] (n=501)	Odds ratio[2]	P value
Strongly agree / Agree	30.8% (20/65)	81.3% (26/32)	28.0% (14/50)	0.58 (0.39-0.88)	0.01
Uncertain / Disagree / Strongly disagree	30.8% (131/426)	91.1% (247/271)	53.1% (238/448)	1.0	
**BC is unnecessary because cultures are often contaminated**					
Strongly agree / Agree	26.3% (19/72)	79.3% (23/29)	34.2% (14/41)	0.64 (0.42-0.98)	0.04
Uncertain / Disagree / Strongly disagree	31.9% (133/417)	90.9% (249/274)	52.2% (236/452)	1.0	
**BC is unnecessary because results often do not agree with clinical signs**					
Strongly agree / Agree	34.0% (18/53)	88.9% (24/27)	23.5% (8/34)	0.77 (0.48-1.22)	0.27
Uncertain / Disagree / Strongly disagree	30.8% (135/439)	89.9% (249/277)	52.9% (241/456)	1.0	
**BC is unnecessary because it is too expensive**					
Strongly agree / Agree	25.5% (24/94)	80.0% (24/30)	32.7% (17/52)	0.62 (0.42-0.92)	0.02
Uncertain / Disagree / Strongly disagree	32.4% (129/398)	91.2% (249/273)	52.9% (229/443)	1.0	
**BC is not benefiting the patients**					
Strongly agree / Agree	14.0% (15/107)	84.0% (21/25)	19.4% (7/36)	0.37 (0.24-0.57)	<0.001
Uncertain / Disagree / Strongly disagree	35.8% (136/380)	90.1% (246/273)	53.0% (239/451)	1.0	
**BC is unnecessary because a contaminated result often leads to wrong therapeutic approaches**					
Strongly agree / Agree	30.4% (7/23)	86.4% (19/22)	20.0% (6/30)	0.53 (0.30-0.95)	0.03
Uncertain / Disagree / Strongly disagree	31.5% (148/470)	90.1% (254/282)	52.5% (245/467)	1.0	
**It is not too late to collect BC later, particularly if patients do not improve after receiving empirical antibiotic treatment**					
Strongly agree / Agree	13.8% (19/138)	88.3% (143/162)	31.2% (38/122)	0.37 (0.27-0.51)	<0.001
Uncertain / Disagree / Strongly disagree	38.1% (134/352)	91.6% (130/142)	57.2% (214/373)	1.0	
**Quality of laboratory is questionable**					
Strongly agree / Agree	24.2% (22/91)	84.2% (32/38)	26.6% (17/64)	0.48 (0.33-0.70)	<0.001
Uncertain / Disagree / Strongly disagree	32.7% (128/391)	90.3% (232/257)	54.1% (230/435)	1.0	
**Levels of local antibiotic resistance are low**					
Strongly agree / Agree	34.7% (17/49)	76.9% (20/26)	32.0% (16/50)	0.64 (0.41-0.98)	0.04
Uncertain / Disagree / Strongly disagree	31.3% (135/432)	91.1% (246/270)	52.8% (235/445)	1.0	
**TDF domain: Intention**					
**Intention to follow local guidelines[3]**					
All the time / Often (>75-100% of the cases)	51.7% (89/172)	90.5% (142/157)	64.7% (183/283)	2.92 (1.88-4.53)	<0.001
Moderately / Occasionally / Rarely (0-74% of the cases)	18.6% (11/59)	100% (12/12)	37.7% (23/61)	1.0	
**TDF domain: Knowledge**					
**Awareness of local guidelines**					
Yes	42.7% (102/239)	91.1% (154/169)	59.5% (206/346)	2.55 (1.93-3.38)	<0.001
No	21.1% (53/251)	89.3% (117/131)	29.4% (42/143)	1.0	

Barriers or enablers	Indonesia[1] (n=503)	Thailand[1] (n=304)	Viet Nam[1] (n=501)	Odds ratio[2]	P value
**Awareness of international guidelines**					
Yes	38.9% (138/226)	90.8% (128/141)	65.9% (147/223)	1.97 (1.50-2.57)	<0.001
No	25.4% (67/264)	89.9% (143/159)	38.0% (101/266)	1.0	
**Any training, lectures, classes or meetings that provide knowledge about guidelines for BC sampling**					
Available	36.2% (92/254)	92.2% (178/193)	53.5% (197/368)	1.68 (1.18-2.38)	0.004
Not available	21.7% (33/152)	82.8% (53/64)	46.2% (24/52)	1.0	
**TDF domain: Social influences**					
**To what extent do you order BC in your hospital because you are following local norms? [4]**					
All the time / Often (>75-100% of the time)	45.3% (81/179)	90.1% (210/233)	61.3% (146/238)	2.20 (1.67-2.90)	<0.001
Moderately / Occasionally / Rarely (0-74% of the time)	22.2% (51/230)	90.6% (58/64)	41.3% (92/223)	1.0	
**TDF domain: Reinforcement**					
**Positive consequences if doctors order a BC when it is recommended**					
Yes	29.9% (20/67)	86.0% (49/57)	42.4% (72/170)	0.53 (0.37-0.74)	<0.001
No	32.0% (136/425)	90.6% (222/245)	57.4% (160/279)	1.0	
**Negative consequences if doctors do not order a BC when it is recommended**					
Yes	39.4% (39/99)	90.1% (127/141)	50.0% (112/224)	0.87 (0.63-1.21)	0.42
No	30.1% (117/389)	89.4% (144/161)	55.6% (120/216)	1.0	
**Negative consequences if doctors order a BC when it is recommended**					
Yes	29.2% (19/65)	86.0% (49/57)	41.4% (67/162)	0.48 (0.34-0.67)	<0.001
No	32.3% (136/421)	90.5% (220/243)	60.1% (170/283)	1.0	
**TDF domain: Behavioural regulation**					
**Considering whether "patients have a health scheme or insurance that covers the cost of BC" as a reason for deciding to do BC sampling [5]**					
Yes	27.7% (31/112)	92.6% (25/27)	38.6% (22/57)	0.82 (0.57-1.18)	0.29
No	32.4% (126/389)	89.5% (248/277)	52.0% (230/442)	1.0	
**Considering whether "Patients are likely to have a final diagnosis that includes the cost of BC in the package of fee for service" as a reason for deciding to do BC sampling [5]**					
Yes	33.8% (24/69)	96.4% (27/28)	41.8% (23/55)	1.04 (0.70-1.54)	0.85
No	30.8% (133/432)	89.1% (246/276)	51.6% (229/444)	1.0	

Suntornsut P, *et al. BMJ Open* 2024; 14:e075526. doi: 10.1136/bmjopen-2023-075526

Barriers or enablers	Indonesia[1] (n=503)	Thailand[1] (n=304)	Viet Nam[1] (n=501)	Odds ratio[2]	P value
**Procedures that support doctors to order or regulate ordering of BC**					
No	44.7% (34/76)	88.7% (63/71)	24.2% (31/128)	1.0	0.006
Poster (and BC is mentioned)	36.8% 921/57)	92.5% (37/40)	51.5% (34/66)	1.13 (0.76-1.69)	
Standard order form for patients with sepsis (with BC written)	32.5% (39/120)	92.2% (83/90)	46.7% (50/107)	0.82 (0.59-1.14)	
Computer system to remind ordering BC	36.0% (9/25)	92.9% (13/14)	45% (33/73)	0.72 (0.48-1.15)	
case reviews (e.g. grand round; with BC often mentioned)	44.7% (34/76)	90.7% (78/86)	57.3% (94/164)	1.38 (0.94-2.00)	
Stewardship programmes (including BC)	49.2% (30/61)	92.0% (23/25)	58.7% (71/121)	1.33 (0.87-2.03)	
Local hospital guideline (e.g. standard operating procedure)	37.2% (42/113)	94.8% (73/77)	58.6% (95/162)	1.45 (1.06-1.99)	
**TDF domain: Environmental context and resources**					
**Do the benefits of BC outweigh the cost?**					
Very likely / likely	35.3% (109/309)	91.0% (211/232)	53.1% (216/407)	1.63 (1.17-2.26)	0.004
Uncertain / Unlikely / Very unlikely	22.0% (31/141)	86.7% (52/60)	42.3% (29/67)	1.0	
**How often are consumables for BC not available?**					
All the time / Often (>75-100% of the time)	31.3% (26/83)	88.9% (24/27)	34.2% (13/38)	0.81 (0.53-1.22)	0.32
Moderately / Occasionally / Rarely (0-74% of the time)	31.9% (114/357)	89.5% (231/258)	53.5% (222/415)	1.0	
**How often are laboratories not available or not functioning?**					
All the time / Often (>75-100% of the time)	28.9% (26/90)	90.9% (2/22)	48.8% (21/43)	0.94 (0.63-1.41)	0.78
Moderately / Occasionally / Rarely (0-74% of the time)	32.6% (114/350)	89.3% (233/261)	53.3% (216/405)	1.0	
**How often do patients have to pay for BC using their own money?**					
All the time / Often (>75-100% of the time)	22.4% (17/76)	92.7% (26/28)	47.1% (16/34)	0.79 (0.51-1.22)	0.29
Moderately / Occasionally / Rarely (0-74% of the time)	36.2% (93/257)	88.1% (178/202)	55.8% (208/373)	1.0	
**Considering whether "patients can afford the cost of BC" as a reason for deciding to do BC sampling**					
Yes	31.1% (33/106)	92.6% (25/27)	46.9% (30/64)	1.12 (0.79-1.61)	0.53
No	31.4% (124/395)	89.5% (248/277)	51.0% (222/435)	1.0	
**TDF domain: Emotion**					
**Any emotional factors**					
Yes	25.5% (13/51)	80% (8/10)	65.6% (21/32)	1.06 (0.65-1.71)	0.82
No	32.0% (144/450)	90.1% (265/294)	49.5% (231/467)	1.0	
**TDF domain: Optimism**					
**Optimistic that a BC will be sampled and processed in the laboratory appropriately**					
Strongly optimistic / Optimistic	33.3% (133/400)	90.5% (238/263)	54.4% (200/368)	1.78 (1.29-2.46)	<0.001
Neither / Pessimistic / Strongly pessimistic	20.7% (18/87)	88.6% (31/35)	39.8% (51/128)	1.0	
**TDF domain: Skills**					

Suntornsut P, *et al. BMJ Open* 2024; 14:e075526. doi: 10.1136/bmjopen-2023-075526

Barriers or enablers	Indonesia[1] (n=503)	Thailand[1] (n=304)	Viet Nam[1] (n=501)	Odds ratio[2]	P value
**How skilled are you in drawing blood? [6]**					
Very good / Good	38.5% (15/39)	88.2% (30/34)	57.1% (40/70)	1.74 (1.02-2.97)	0.04
Fair / Poor / Very poor	31.8% (14/44)	93.1% (81/87)	35.1% (20/57)	1.0	
**TDF domain: Memory, attention and decision processes**					
**Even when BC is recommended, would you still order BC if patients are already on antibiotics**					
Definitely not order / likely not order	20.0% (6/30)	86.6% (58/67)	41.2% (14/34)	0.69 (0.42-1.11)	0.13
Maybe not order/ likely to still order / very likely to still order	31.2% (142/455)	90.6% (213/235)	51.3% (238/464)	1.0	
**Even when BC is recommended, would you still order BC if patients have anemia**					
Definitely not order / likely not order	21.3% (16/75)	91.9% (136/148)	47.4% (27/57)	0.89 (0.62-1.28)	0.55
Maybe not order/ likely to still order / very likely to still order	32.2% (128/398)	87.3% (130/149)	51.3% (220/429)	1.0	
**TDF domain: Beliefs about capabilities**					
**Are you confident that you can draw blood successfully? [6,7]**					
Strongly confident / Confident	34.7% (25/72)	89.1% (57/64)	51.9% (56/108)	1.39 (0.69-2.79)	0.36
Uncertain / Doubtful / Strongly doubtful	36.4% (4/11)	94.7% (54/57)	22.2% (4/18)	1.0	
**Are you confident that you can draw blood appropriately? [6,7]**					
Strongly confident / Confident	34.8% (24/69)	89.7% (70/78)	54.6% (54/99)	1.67 (0.88-3.17)	0.11
Uncertain / Doubtful / Strongly doubtful	35.7% (5/14)	95.2% (40/42)	22.2% (6/27)	1.0	
**Are you confident that others (who are tasked to draw blood in your hospital) can draw blood successfully? [7]**					
Strongly confident / Confident	30.7% (142/463)	90.1% (254/282)	52.5% (212/404)	1.35 (0.91-2.00)	0.13
Uncertain / Doubtful / Strongly doubtful	33.3% (11/33)	85.7% (18/21)	43.0% (40/93)	1.0	
**Are you confident that others (who are tasked to draw blood in your hospital) can draw blood appropriately? [7]**					
Strongly confident / Confident	31.0% (132/426)	89.6% (224/250)	52.8% (168/318)	1.20 (0.89-1.62)	0.23
Uncertain / Doubtful / Strongly doubtful	31.9% (22/69)	90.6% (48/53)	46.6% (83/178)	1.0	

[1] Percentage of participants who answered with "definitely take BC" in the case scenario are presented. For each question, participants who answered 'I do not know' or 'I do not want to answer' were excluded. [2] Estimated by using logistic regression models with random effects for countries, for types of hospital nested in the same country, and for professional roles nested in the same types of hospital. [3] Among those who answered that they know of local guidelines. [4] "Norms" means usual practice that are typical of or accepted within your hospital. [5] Included answers in Q1-7 (which were asked to those who answered that they knew of local guideline) and Q1-8 (which were asked to those who answered that they did not know of local guideline) (Appendix S3). [6] Among those who answered that their professional roles are tasked of drawing blood for BC. [7] "Successfully" means obtaining blood; "Appropriately" means that general recommendations for BC specimen collection such as aseptic technique are followed.

Appendix S7. Links between COM-B components (Capability, Opportunity, motivation and behaviour components), and suggested intervention types and policy options.

Links between COM-B components and intervention types*

Intervention types	COM-B components					
	Capability		Opportunity		Motivation	
	Psychological	Physical	Social	Physical	Reflective	Automatic
**Education**		X			X	
**Persuasion**					X	X
**Incentivisation**					X	X
**Coerction**					X	X
**Training**	X	X				
**Restriction**			X	X		
**Environmental restructuring**			X	X		X
**Modelling**						X
**Enablement**	X	X	X	X		X

* as previously published.[8]

Links between intervention types and policy categories*

Intervention types	Policy categories						
	Communi-cation/ Marketing	Guidelines	Fiscal	Regulation	Legislation	Environ-mental/ Social planning	Service provision
**Education**	X	X		X	X		X
**Persuasion**	X	X		X	X		X
**Incentivisation**	X	X	X	X	X		X
**Coerction**	X	X	X	X	X		X
**Training**		X	X	X	X		X
**Restriction**		X		X	X		
**Environmental restructuring**		X	X	X	X	X	
**Modelling**	X						X
**Enablement**		X	X	X	X	X	X

* as previously published.[8]

Suntornsut P, *et al. BMJ Open* 2024; 14:e075526. doi: 10.1136/bmjopen-2023-075526

References

1. Chongsuvivatwong V, Phua KH, Yap MT, Pocock NS, Hashim JH, Chhem R, et al. Health and health-care systems in southeast Asia: diversity and transitions. Lancet. 2011;377(9763):429-37.

2. World Bank. GDP (current US$). Available from: https://data.worldbank.org/indicator/NY.GDP.MKTP.CD

3. Tangcharoensathien V, Limwattananon S, Patcharanarumol W, Thammatacharee J. Monitoring and evaluating progress towards Universal Health Coverage in Thailand. PLoS Med. 2014;11(9):e1001726.

4. Sumriddetchkajorn K, Shimazaki K, Ono T, Kusaba T, Sato K, Kobayashi N. Universal health coverage and primary care, Thailand. Bull World Health Organ. 2019;97(6):415-22.

5. Le QN, Blizzard L, Si L, Giang LT, Neil AL. The evolution of social health insurance in Vietnam and its role towards achieving universal health coverage. Health Policy OPEN. 2020;1:100011.

6. Thuan NT, Lofgren C, Lindholm L, Chuc NT. Choice of healthcare provider following reform in Vietnam. BMC Health Serv Res. 2008;8:162.

7. WHO. Out-of-pocket expenditure (OOP) per capita in US$. Available from: https://www.who.int/data/gho/data/indicators/indicator-details/GHO/out-of-pocket-expenditure-(oop)-per-capita-in-us

8. Michie S, van Stralen MM, West R. The behaviour change wheel: A new method for characterising and designing behaviour change interventions. Implementation Science. 2011;6(1):42.
