# Supplementary material for: Barriers and enablers to blood culture sampling in Indonesia, Thailand and Viet Nam: a Theoretical Domains Framework-based survey
Source: BMJ Open. 2024 Feb 19;14(2):e075526. doi: 10.1136/bmjopen-2023-075526 (PMC10882306; doi:10.1136/bmjopen-2023-075526)
Supplement: Author's manuscript [file bmjopen-2023-075526.draft_revisions.pdf]

BMJ Open

BMJ Open is committed to open peer review. As part of this commitment we make the peer review history of every article we publish publicly available.

When an article is published we post the peer reviewers' comments and the authors' responses online. We also post the versions of the paper that were used during peer review. These are the versions that the peer review comments apply to.

The versions of the paper that follow are the versions that were submitted during the peer review process. They are not the versions of record or the final published versions. They should not be cited or distributed as the published version of this manuscript.

BMJ Open is an open access journal and the full, final, typeset and author-corrected version of record of the manuscript is available on our site with no access controls, subscription charges or pay-per-view fees (http://bmjopen.bmj.com).

If you have any questions on BMJ Open's open peer review process please email info.bmjopen@bmj.com

BMJ Open

Barriers and enablers to blood culture sampling in Indonesia, Thailand and Vietnam

Journal:	*BMJ Open*
Manuscript ID	bmjopen-2023-075526
Article Type:	Original research
Date Submitted by the Author:	11-May-2023

Complete List of Authors:	Suntornsut, Pornpan; Mahidol Oxford Tropical Medicine Research Unit<br>Asadinia, Koe Stella; Eijkman-Oxford Clinical Research Unit<br>Limato, Ralalicia; Oxford University, Eijkman-Oxford Clinical Research Unit; Oxford University, Centre for Tropical Medicine and Global Health, Nuffield Department of Medicine<br>Tamara, Alice; Faculty of Medicine Universitas Indonesia<br>Rotty, Linda W.A.; Prof Dr RD Kandou Central Hospital<br>Bramanti, Rendra; Dr. Iskak District Hospital<br>Nusantara, Dwi U.; Pasar Minggu District Hospital<br>Nelwan, Erni J.; Universitas Indonesia; Cipto Mangukusumo National General Hospital, Department of Internal Medicine<br>Khusuwan, Suwimon; Chiangrai Prachanukroh Hospital<br>Suphamongkholchaikul, Watthanapong; Sunpasitthiprasong Hospital<br>Chamnan, Parinya; Sunpasithiprasong Hospital<br>Piyaphanee, Watcharapong; Mahidol University<br>Vu, Huong; Oxford University Clinical Research Unit Viet Nam, Hanoi Unit<br>Hai Yen, Nguyen; Oxford University Clinical Research Unit<br>Nguyen Hong, Khanh; Oxford University Clinical Research Unit<br>Pham, Ngoc Thach; National Hospital of Tropical Diseases<br>Le, Minh Quang; Viet Tiep Hospital<br>Vu, Hai Vinh; Assistance Publique des Hôpitaux de Marseille, Pôle de Maladies Infectieuses,<br>Duc, Chau Minh; Dong Thap Hospital<br>Vo, Thi Hoang Dung Em; Oxford University Clinical Research Unit<br>Harriss, Elinor; University of Oxford, Bodleian Health Care Libraries<br>van Doorn, H. Rogier; Oxford University Clinical Research Unit; Nuffield Department of Clinical Medicine<br>Hamers, Raph Leonardus; Eijkman-Oxford Clinical Research Unit; Centre for Tropical Medicine and Global Health, Nuffield Department of Medicine, University of Oxford,<br>Lorencatto, Fabiana; UCL<br>Atkins, Lou; UCL<br>Limmathurotsakul, Direk; Mahidol University; University of Oxford, Nuffield Department of Medicine

Keywords:	Diagnostic microbiology < INFECTIOUS DISEASES, Public Hospitals < Hospitals, Public, INFECTIOUS DISEASES, MICROBIOLOGY, QUALITATIVE RESEARCH, Surveys and Questionnaires

I, the Submitting Author has the right to grant and does grant on behalf of all authors of the Work (as defined in the below author licence), an exclusive licence and/or a non-exclusive licence for contributions from authors who are: i) UK Crown employees; ii) where BMJ has agreed a CC-BY licence shall apply, and/or iii) in accordance with the terms applicable for US Federal Government officers or employees acting as part of their official duties; on a worldwide, perpetual, irrevocable, royalty-free basis to BMJ Publishing Group Ltd ("BMJ") its licensees and where the relevant Journal is co-owned by BMJ to the co-owners of the Journal, to publish the Work in this journal and any other BMJ products and to exploit all rights, as set out in our licence.

The Submitting Author accepts and understands that any supply made under these terms is made by BMJ to the Submitting Author unless you are acting as an employee on behalf of your employer or a postgraduate student of an affiliated institution which is paying any applicable article publishing charge ("APC") for Open Access articles. Where the Submitting Author wishes to make the Work available on an Open Access basis (and intends to pay the relevant APC), the terms of reuse of such Open Access shall be governed by a Creative Commons licence – details of these licences and which Creative Commons licence will apply to this Work are set out in our licence referred to above.

Other than as permitted in any relevant BMJ Author's Self Archiving Policies, I confirm this Work has not been accepted for publication elsewhere, is not being considered for publication elsewhere and does not duplicate material already published. I confirm all authors consent to publication of this Work and authorise the granting of this licence.

For peer review only

Barriers and enablers to blood culture sampling in Indonesia, Thailand and Vietnam

Pornpan Suntornsut, MSc[1], Koe Stella Asadinia, MD[2], Ralalicia Limato, MD[2,3], Alice Tamara, MD[2], Linda W.A. Rotty, MD[4], Rendra Bramanti, MD[5], Dwi U. Nusantara, MD[6], Erni J. Nelwan, MD[7,8] Suwimon Khusuwan, MD[9], Watthanapong Suphamongkholchaikul, MD[10], Parinya Chamnan, MD[10], Watcharapong Piyaphanee, MD[11], Vu Thi Lan Huong, MD[12], Nguyen Hai Yen, MPH[12], Khanh Nguyen Hong, MSc[12], Pham Ngoc Thach, MD[13], Le Minh Quang, MD[14], Vu Hai Vinh, PhD[14], Chau Minh Duc, PhD[15], Vo Thi Hoang Dung Em MD[15], Elinor Harriss, MSc[16], H Rogier van Doorn, MD[3,12], Raph L. Hamers MD[2,3], Fabiana Lorencatto, PhD[17], Lou Atkins, PhD[17], Direk Limmathurotsakul, MD[1,3,18]

[1] Mahidol-Oxford Tropical Medicine Research Unit, Faculty of Tropical Medicine, Mahidol University, Bangkok, Thailand

[2] Oxford University Clinical Research Unit Indonesia, Faculty of Medicine Universitas Indonesia, Jakarta, Indonesia

[3] Centre for Tropical Medicine and Global Health, Nuffield Department of Medicine, University of Oxford, Oxford, United Kingdom

[4] Prof. Dr. R.D. Kandou Central Hospital, Manado, Indonesia

[5] Dr. Iskak District Hospital, Tulungagung, Indonesia

[6] Pasar Minggu District Hospital, Jakarta, Indonesia

[7] Faculty of Medicine, Universitas Indonesia, Jakarta, Indonesia

[8] Department of Internal Medicine, Division of Infectious Diseases, Cipto Mangukusumo National General Hospital, Jakarta, Indonesia

For peer review only

For peer review only - http://bmjopen.bmj.com/site/about/guidelines.xhtml

[9] Chiangrai Prachanukroh Hospital, Chiang Rai, Thailand

[10] Sunpasitthiprasong Hospital, Ubon Ratchathani, Thailand

[11] Hospital for Tropical Diseases, Faculty of Tropical Medicine, Mahidol University, Bangkok, Thailand

[12] Oxford University Clinical Research Unit, Hanoi, Vietnam.

[13] National Hospital for Tropical Diseases, 78 Giai Phong, Ha Noi, Viet Nam

[14] Viet Tiep Hospital, 1 Nha Thuong, Cat Dai, Le Chan, Hai Phong, Viet Nam

[15] Dong Thap Hospital, 144 Mai Van Khai, My Tan, Cao Lanh City, Dong Thap, Viet Nam

[16] Bodleian Health Care Libraries, University of Oxford, Oxford, United Kingdom

[17] Centre for Behaviour Change, University College London, London, United Kingdom

[18] Department of Tropical Hygiene, Faculty of Tropical Medicine, Mahidol University, Bangkok, Thailand

* Correspondence to:

Direk Limmathurotsakul

Mahidol-Oxford Tropical Medicine Research Unit, Faculty of Tropical Medicine, Mahidol University, Bangkok, Thailand, 10400. email: direk@tropmedres.ac

Word count: abstract 300, text 3,776

For peer review only

Abstract

Objective

Blood culture (BC) sampling is recommended for all suspected sepsis patients prior to antibiotic administration. We examine barriers and enablers to BC sampling in three Southeast Asian countries.

Design

A Theoretical Domains Framework (TDF)-based survey, comprising a case scenario of a patient presenting with community-acquired sepsis and all 14 TDF domains of barriers/enablers to BC sampling.

Setting

Hospitals in Indonesia, Thailand and Vietnam

Participants

1,070 medical doctors and 238 final-year medical students from 24 of 34 provinces in Indonesia, 39 of 77 provinces in Thailand, and 25 of 63 provinces in Vietnam. Half of respondents were female (n=680, 52%) and most worked in governmental hospitals (n=980, 75.4%)

Primary outcome measures

Barrier and enabler to blood culture sampling. The key TDF domains were mapped to the Capability, Opportunity, Motivation, Behaviour model, and potentially relevant intervention strategies were identified.

Results

The proportion of respondents who answered that they would definitely take BC in the case scenario was highest at 89.8% (273/304) in Thailand, followed by 50.5% (252/499) in Vietnam and 31.3% (157/501) in Indonesia (p<0.001). Barriers/enablers in nine TDF domains were

For peer review only - http://bmjopen.bmj.com/site/about/guidelines.xhtml

considered key in influencing BC sampling, including 'priority of BC [TDF-goals]', 'perception about their role to order or initiate an order for BC [TDF-social professional role and identity]', 'perception that BC is helpful [TDF-beliefs about consequences]', 'intention to follow guidelines [TDF-intention]', 'awareness of guidelines [TDF-knowledge]', 'norms of BC sampling [TDF-social influence]', 'consequences that discourage BC sampling [TDF-reinforcement]', 'perceived cost-effectiveness of BC [TDF-environmental context and resources]' and 'regulation on cost reimbursement [TDF-behavioural regulation]'. There was substantial heterogeneity between the countries across most domains. A range of suggested intervention types and policy options were identified.

Conclusions

Barriers and enablers to BC sampling are varied and heterogenous. Context-specific multifaceted interventions at both hospital and policy levels are required to improve diagnostic stewardship practices.

For peer review only

Strenght and limitations of this study

- The theoretical domains framework survey comprehensively identified individual, socio-cultural and environmental barriers and enablers to blood culture sampling across study countries
- A convenient sampling approach, distributing invitations in letters, emails, pamphlets and online social media platforms, through existing collaborations in hospitals in the three survey countries was used.
- The target sample size was not reached in Thailand, but the study had enough power to compare barriers and enablers between study countries.
- The findings may not be generalizable to all low and middle-income countries because barriers and enablers to blood culture sampling can be varied and local evaluations are needed.

For peer review only

Introduction

Blood culture (BC) is a crucial diagnostic, which can guide antibiotic treatment decisions of severe bacterial infections, and may improve patient outcomes.[1,2] The cumulative results of BC are also crucial to inform antimicrobial resistance (AMR) surveillance, at the hospital, country and global levels.[3] International guidelines on sepsis management have been stressing the importance of obtaining BC before or, when not possible, within 24 hours after administration of antibiotics.[1,4]

Nonetheless, BC is generally underutilized, both in high-income countries (HICs) and low and middle-income countries (LMICs), with wide variations in reported BC sampling rates between hospitals and global regions. Reported BC sampling rates ranged from 196 to 308 per 1,000 patient-days in the United States,[5,6] from 6.7 to 86.5 per 1,000 patient-days in the European Union,[7] from 0 to 82 per 1,000 patient-days in the Central Asian and European Surveillance of AMR network (CAESAR),[8] and 31, 82 and 10 per 1,000 patient-days in selected hospitals in Indonesia,[9] Thailand[10] and Vietnam[11], respectively.

A range of barriers and enablers have been identified that influence BC sampling, based on different study designs, theories and frameworks. Lack of clear guidelines, training, microbiological infrastructure, and positive attitudes regarding BC among medical practitioners, are commonly reported barriers.[8,12-15]

Changing the behavior of medical practitioners is complex, and a systematic approach has been shown useful to understand factors influencing adherence to guidelines or recommendations so

For peer review only - http://bmjopen.bmj.com/site/about/guidelines.xhtml

as to inform the design of future interventions.[16-18] The Theoretical Domains Framework (TDF) has been developed by synthesizing a wide range of theories, and enables researchers to investigate a broader range of individual, socio-cultural and environmental behavioral influences than they would with a single theory alone.[16-18] The TDF has been widely used to explore barriers and enablers to healthcare professional behaviors, including diagnostic testing, antimicrobial stewardship, and infection prevention control.[19-22]

Here, we aimed to identify barriers and enablers to BC sampling in three middle-income countries in Southeast Asia (SEA) using a theory-based approach informed by the TDF.

Methods

The TDF survey

We developed a TDF survey questionnaire, comprising a hypothetical case scenario and all 14 TDF domains of barriers/enablers to BC sampling, through an iterative process of systematic literature review and previous TDF surveys on other health topics (Table 1; Appendix S1 and S2).[23-26] Each question used a five-point Likert scale representing the level of perceived barriers/enablers to BC sampling under all TDF domains.

For peer review only

Table 1: Key questions for barriers and enablers to blood culture (BC) sampling

TDF Domains	Questions
Knowledge	Do you know of any recommendation(s) or guideline(s) for BC sampling being used in your hospital?
	In your hospital, are there any training, lectures, classes or meetings that provide you knowledge about local/national/international guidelines for BC sampling?
Skills	How skilled are you in drawing blood?
Social professional role and identity	In your current hospital setting, which types of professionals/staff can order a BC?
	Do you think that it is an appropriate part of your current job to order a BC?
	Do you think that it is an appropriate part of your current job to draw blood for BC?
Beliefs about capabilities	If you have to draw blood yourself, are you confident that you can draw blood successfully? "Successfully" means obtaining blood.
Optimism	In your current hospital setting, how optimistic are you that a BC will be sampled and processed in the laboratory appropriately if you order a BC?
Beliefs about consequences	Do you agree or disagree about the following potential advantages of BC, making BC helpful in your current hospital setting?
	Do you agree or disagree about the following disadvantages of BC, making BC unnecessary in your current hospital setting?
Reinforcement	Are there any positive consequences to you, if you order BC when recommended?
	Are there any negative consequences to you, if you do not order BC when recommended?
	Are there any negative consequences to you, if you order BC when recommended?
Intentions	How often do you plan to follow the recommendation(s) or guideline(s) for BC sampling being used in your hospital?
Goals	How often do you obtain BC prior to administration of empirical antibiotics in patients presenting with sepsis?
Memory, attention and decision processes	Apart from the recommendation(s) or guideline(s) being used at your hospital, do you have any additional reasons for deciding to do BC sampling?
Environmental context and resources	Regardless of who pays for the cost of BC, would you say that the benefits of BC outweigh the cost?
	In your hospital, how often could you not order BC because consumables (such as blood culture bottles, needles, syringes, blood collection set, etc.) are not available?
Social influences	To what extent do you order BC sampling because you are following local norms? "Norms" mean usual practice that are typical of or accepted within your hospital.
	Do following people have any positive or negative influence on you to order BC?
Emotion	Apart from your logical considerations, do you think that any emotional factors of anyone involved in ordering and sampling for BC
Behavioural regulation	In your hospital, are there any procedures that support you to order or regulate ordering of BC per local/national/international guidelines?

TDF = Theoretical Domain Framework

For peer review only - http://bmjopen.bmj.com/site/about/guidelines.xhtml

The initial questionnaire was translated into Thai, Vietnamese and Indonesian language and piloted among 10-19 medical doctors and 3-6 final-year medical students in each country (a total of 54 respondents) to test the clarity of questions and choice answers in each language and to ensure no potential key barriers/enablers were omitted. We asked respondents to complete the survey and provide feedback using 1:1 interviews via phone or using online meeting software. The questionnaire was revised and finalized based on the pilot study results. During the pilot survey, we included 'monetary reward' and 'monetary fine' as examples of positive and negative consequences to BC sampling, respectively. We received strong feedbacks that those are not present for BC sampling in Indonesia, Thailand and Vietnam. Therefore, the word 'monetary reward' and 'monetary fine' were removed. One free-text question was added (i.e. Question 6-5, "Additional comments about emotional factors…"), a total of 27 choice answers were added, and languages and wordings were revised. The final questionnaire included 54 questions about barriers/enablers to BC sampling and respondents' demographic characteristics (Appendix S3).

Study participants

We invited medical doctors and final-year medical doctors in Indonesia, Thailand and Vietnam to complete the online TDF survey. We used a convenient sampling approach, distributing invitations in letters, emails, pamphlets and online social media platforms, through existing collaborations in hospitals in the three survey countries. The online survey was conducted using the Qualtrics survey platform.

We used a simple formula for calculating the sample size.[27] Assuming prevalence of a barrier or enabler to be 50% among medical doctors, with a margin of error 5%, the sample size of medical

For peer review only - http://bmjopen.bmj.com/site/about/guidelines.xhtml

doctors was estimated to be at least 385 per country. Assuming prevalence of a barrier or enabler to be 50% among final-year medical students, with a margin of error 10%, the sample size of final-year medical students was estimated to be at least 97 per country. Therefore, we aimed to enroll 400 medical doctors and 100 final-year medical students in each country (a total of 1,500 respondents).

Analysis

For each question, we defined that respondents who answered "definitely"/"likely", "all the time"/"often" or "strongly agree"/"agree" perceived the importance or agreement with that barrier/enabler. The proportion of respondents who answered likewise, after excluding respondents who answered 'I do not know' or 'I do not want to answer', was presented. Groups were compared by Chi-squared or Fisher exact tests as appropriate. Logistic regression models with random effects for countries, for hospital type nested in the same country, and for professional roles nested in the same hospital type were used to evaluate the association between respondents' answers about each barrier/enabler and to the case scenario. Multivariable logistic regression model was not used because we considered that each key TDF domain could influence BC sampling practice via a causal relationship and should be addressed in future interventions. Statistical analyses were performed using Stata 15.1 (StataCorp, US).

We identified and ranked important TDF domains by scoring them based on an established set of four 'importance criteria' (modified from a previous TDF study[28]): (1) 'frequency' (the proportion of respondents who perceived the importance or agreement with a barrier/enabler); (2) 'elaboration' (number of themes within each domain); (3) 'expressed importance' (quotes

For peer review only - http://bmjopen.bmj.com/site/about/guidelines.xhtml

from respondents expressing importance or agreement); and (4) ‘association between reported barriers/enablers and BC practice’ (size of effect and strength of association, i.e., odds ratios [ORs] and p values, obtained from the logistic regression models, respectively).

Lastly, we mapped identified TDF domains to the COM-B ('Capability', 'Opportunity', 'Motivation' and 'Behaviour') model.[16-18] COM-B forms the hub of the Behaviour Change Wheel (BCW), a framework which signposts to potentially relevant intervention strategies. This allowed us to list all intervention types and policy options that were likely to be effective in addressing identified barriers and enablers.

Patient and public involvement

Patients and the public were not involved in this research’s design, conduct, reporting or dissemination plans.

Results

From 1 December 2021 to 30 April 2022, 1,070 medical doctors and 238 final-year medical students in Indonesia, Thailand and Vietnam completed the online TDF survey. Half of respondents were female (n=680, 52%) and most worked in governmental hospitals (n=980, 75.4%) (Table 2). The most common department was internal medicine (n=450, 34.4%), followed by emergency (n=175, 13.4%) and pediatrics (n=153, 11.7%). Respondents were from 24 of 34 provinces in Indonesia, 39 of 77 provinces in Thailand, and 25 of 63 provinces in Vietnam.

For peer review only

Table 2: Demographics and responses to the hypothetical case scenario

Variables	Indonesia (n=503)	Thailand (n=304)	Vietnam (n=501)	P values
**Female gender**	263 (52.3%)	195 (64.1%)	222 (44.3%)	<0.001
**Hospital types**				
Government hospital	340 (67.6%)	209 (68.8%)	431 (86.0%)	<0.001
Private hospital	113 (22.5%)	15 (4.9%)	17 (3.4%)	
University hospital	26 (5.2%)	76 (25.0%)	29 (5.8%)	
Other[1]	19 (3.8%)	2 (0.7%)	22 (4.4%)	
I do not want to answer	5 (1.0%)	2 (0.7%)	2 (0.4%)	
**Hospital bed size**				
<200	99 (19.7%)	35 (11.5%)	24 (4.8%)	<0.001
201-400	107 (21.3%)	46 (15.1%)	29 (5.8%)	
401-600	72 (14.3%)	39 (12.8%)	62 (12.4%)	
601-1,000	66 (13.1%)	45 (14.8%)	144 (28.7%)	
1,001-2,000	39 (7.8%)	82 (27.0%)	125 (25.0%)	
>2,000	27 (5.4%)	30 (9.9%)	74 (14.8%)	
I do not know	89 (17.7%)	27 (8.9%)	35 (7.0%)	
I do not want to answer	4 (0.8%)	0 (0%)	8 (1.6%)	
**Current job[2]**				
Medical doctor – executive level	13 (2.6%)	5 (1.6%)	17 (3.4%)	<0.001
Medical doctor – consultant level	74 (14.7%)	75 (24.7%)	198 (39.5%)	
Medical doctor – physician level	124 (24.7%)	38 (12.5%)	112 (22.4%)	
Medical doctor – resident level	168 (33.4%)	63 (20.7%)	101 (20.2%)	
Medical doctor – intern level	33 (6.6%)	35 (11.5%)	14 (2.8%)	
Final-year medical student	91 (18.1%)	88 (28.9%)	59 (11.8%)	
**Department**				
Internal medicine	149 (29.6%)	155 (51.0%)	146 (29.1%)	<0.001
Pediatrics	65 (12.9%)	43 (14.1%)	45 (9.0%)	0.05
Infection disease division/department	12 (2.4%)	5 (1.6%)	56 (11.2%)	<0.001
Surgery	21 (4.2%)	45 (14.8%)	81 (16.2%)	<0.001
Orthopaedics	6 (1.2%)	18 (5.9%)	14 (2.8%)	0.001
Obstetrics / Gynaecology	20 (4.0%)	29 (9.5%)	7 (1.4%)	<0.001
Emergency department	112 (22.3%)	34 (11.2%)	29 (5.8%)	<0.001
Intensive care unit	45 (8.9%)	13 (4.3%)	51 (10.2%)	0.01
**Case-study: Would you take a BC sample in a case presenting with community-acquired sepsis?** [3,4]				
Definitely (>95-100% of the time)	157 (31.2%)	273 (89.8%)	252 (50.3%)	<0.001
Likely (75-95% of the time)	138 (27.4%)	23 (7.6%)	149 (29.7%)	
Maybe (25-74% of the time)	116 (23.1%)	5 (1.6%)	70 (14.0%)	
Unlikely (5-24% of the time)	44 (8.7%)	2 (0.7%)	19 (3.8%)	
Rarely (ranging from never to <5% of the time)	46 (9.1%)	1 (0.3%)	9 (1.8%)	
I do not know	1 (0.2%)	0 (0%)	1 (0.2%)	
I do not want to answer	1 (0.2%)	0 (0%)	1 (0.2%)	

See Appendix S3 for the questionnaire and Appendix S4 for the complete results of the TDF survey. [1] Included clinics (n=3) and text answers that could not be used to determine the hospital type such as internship and medical students. [2] In the survey, for a medical doctor, 'executive level' was defined as having an administrative position without clinical work, 'consultant' was defined as having a clinical specialty degree, 'resident' as currently under postgraduate clinical training, 'physician' as having no clinical specialty/subspecialty degree and not under postgraduate clinical training, and 'intern' as a recent medical school graduate in the first year of post-graduate on-the-job training. [3] In the survey, for the questions asking "Would you …" or "How often …", the Likert scale

For peer review only - http://bmjopen.bmj.com/site/about/guidelines.xhtml

options were defined as 'definitely' or 'all the time' (reflecting >95-100% of the time or case, respectively), 'likely' or 'often' (75-95%), 'uncertain' or 'moderately' (25-74%), 'unlikely' or 'occasionally' (5-24%), and 'rarely' (<5%). Each question also included 'I do not know' and 'I do not want to answer' options.[4] Hypothetical case scenario. "A 72-year-old woman who was brought to the emergency department of your hospital by her daughter when she noticed the patient was more confused than her baseline and was found to have a high fever and fast breathing. She had an auscultatory finding compatible with pneumonia. It is decided that this patient will be admitted to your hospital." If you have an authority to take a blood culture, would you take blood culture sample(s) in this case on admission?

Based on the case scenario of a patient presenting with community-acquired sepsis, half of respondents (52.3%, 682/1,304) answered that they would definitely take BC. However, the responses were significantly different between the three countries (p<0.001). Most Thai respondents (89.8%, 273/304) answered that they would definitely take BC, while half of Vietnamese respondents (50.5%, 252/499) and about a third of Indonesian respondents (31.3%, 157/501) did.

We present, in rank order, the nine TDF domains that were considered very important (i.e. key) in the three countries in SEA in the section below (Appendix S4, S5, S6 and S7).

TDF-Goals

Theme: Priority of BC. In many settings, ordering or initiating an order for BC can take only few seconds by writing "blood culture" in the doctor order form. We used a question asking about the priority of BC compared to that of empirical antibiotics, and 91.3% (274/300) of Thai respondents answered that they obtain BC prior to administration of empirical antibiotics all the time or often, while 80.0% (380/475) of Vietnamese respondents and 54.2% (251/463) of Indonesian respondents answered likewise (p<0.001). Respondents who gave priority to BC were more likely to answer with "definitely take BC" in the case scenario (OR 4.25, 95%CI 3.04-5.94, p<0.001).

For peer review only - http://bmjopen.bmj.com/site/about/guidelines.xhtml

TDF-Social professional role and identity

Theme: Perception about their role to order or initiate an order for BC. Most medical doctors (86.5%, 905/1,046) answered that it is very appropriate or appropriate for them to order BC or initiate an order for BC, while only about half of final-year medical students (49.8%; 115/231) answered likewise ($p<0.001$). Among medical doctors, 95.8% (207/216) of Thai respondents answered that it is very appropriate or appropriate for them to order BC or initiate an order for BC, while 87.0% (368/423) of Vietnamese respondents and 81.1% (330/407) of Indonesia respondents answered likewise ($p<0.001$). The respondents who answered that it is their role to order or initiate an order for BC were more likely to answer with "definitely take BC" in the case scenario (OR 3.36, 95%CI 2.50-4.51, $p<0.001$).

Theme: Level of doctors who can order or initiate an order for BC. More than 75% of Thai respondents answered that all levels of medical doctors (consultants, physicians, residents and interns) can order or initiate an order for BC in their hospitals, while most Indonesian and Vietnamese respondents (87.9%, 870/990) answered that consultants can, but fewer answered that physicians (61.8%, 612/990), residents (59.1%, 585/990) and interns (20.3%, 201/990) can ($p<0.001$). A quarter of Thai respondents (28.7%, 87/303) answered that final-year medical students can order or initiate an order for BC under supervision of attending medical doctors, while Indonesian respondents (2.2%, 11/500) and Vietnamese respondents (0.6%, 3/490) rarely answered likewise ($p<0.001$). None reported that nurses can order or initiate an order for BC.

For peer review only - http://bmjopen.bmj.com/site/about/guidelines.xhtml

Theme: perception about their role to draw blood for BC. Most respondents (72.8%, 949/1,303) answered that registered nurses are tasked to draw blood from patients for BC, followed by microbiology laboratory team (36.0%, 469/1,303), specialized blood draw team (27.4%, 357/1,303), residents (25.4%, 331/1,303), physicians (23.5%, 306/1,303), consultants (23.2%, 302/1,303), interns (17.8%, 229/1,303) and final-year medical students (11.6%, 151/1,303). Of respondents who answered that they are tasked to draw blood from patients for BC, 69.1% (248/359) responded that it is very appropriate or appropriate for their role to draw blood for BC. Those respondents were more likely to answer with "definitely take BC" in the case scenario (OR 1.94, 95%CI 1.04-3.64, p=0.04).

TDF-Belief about consequences

Theme: Perceived that BC is helpful. Respondents who answered that BC is helpful in clinical decisions (OR 2.96, 95%CI 1.71-5.12, p<0.001), reducing patient mortality (OR 1.61; 95%CI 1.18-2.20, p=0.003), ruling in an infection (OR 1.58, 95%CI 1.04-2.39, p=0.03), reducing length of hospital stay (OR 1.53, 95%CI, 1.14-2.04, p=0.004) or understanding epidemiology of AMR bacterial infections (OR 2.89, 95%CI 1.60-5.19, p<0.001) were more likely to answer with "definitely take BC" in the case scenario. The proportion of respondents who answered that BC is helpful in clinical decisions was highest in Thai (97.7%, 297/304), followed by Indonesia (96.6%, 483/500) and Vietnam (88.2%, 440/499, p<0.001).

Theme: Perceived that BC is unnecessary. Respondents who answered that BC is unnecessary because BC is not benefiting the patients (OR 0.37; 95%CI 0.24-0.57, p<0.001), antibiotic therapy can be determined based on clinical presentation (OR 0.51, 95%CI 0.36-0.73, p<0.001),

For peer review only - http://bmjopen.bmj.com/site/about/guidelines.xhtml

the scientific basis of the guideline on BC is questionable (OR 0.66, 95%CI 0.45-0.98, p=0.04), BC results are often delayed (OR 0.48, 95%CI 0.33-0.69, p<0.001), BC results are often not interpretable (OR 0.54, 95%CI 0.34-0.87, p=0.01), BC results are often negative or no growth (OR 0.58, 95%CI 0.39-0.88, p=0.01), cultures are often contaminated (OR 0.64, 95%CI 0.42-0.98, p=0.04), a contaminated result often leads to wrong therapeutic approach (OR 0.53; 95%CI 0.30-0.95, p=0.03), it is not too late to collect BC later, particularly if patients do not improve after receiving empirical antibiotic treatment (OR 0.37; 95%CI 0.27-0.52, p<0.001), quality of laboratory is questionable (OR 0.48; 95%CI 0.33-0.70, p<0.001) or levels of local antibiotic resistance are low (OR 0.64; 95%CI 0.41-0.98, p=0.04) were less likely to answer with "definitely take BC" in the case scenario. The proportion of respondents who answered that BC is not benefitting the patients was not different between countries (5.9%, 76/1,297, p=0.38).

TDF-Intention

Theme: Intention to follow guidelines. Among those who answered that they know of local guidelines, 92.9% (157/169) of Thai respondents answered that they plan to follow local guidelines all the time or often, while 82.0% (283/345) of Vietnamese respondents and 74.1% (172/232) of Indonesian respondents answered likewise (p<0.001). Respondents who intended to follow local guidelines were more likely to answer with "definitely take BC" in the case scenario (OR 2.92, 95% CI 1.88-4.53, p<0.001).

TDF-Knowledge

Theme. Awareness of guidelines. The proportion of respondents who answered that they know of local guidelines for BC sampling was highest in Vietnam (70.7%; 347/491), followed by

For peer review only

For peer review only - http://bmjopen.bmj.com/site/about/guidelines.xhtml

Thailand (56.3%, 169/300) and Indonesia (48.9%, 240/503, p<0.001). The proportion of respondents who answered that they know of international guidelines for BC sampling (47.8%, 596/1,248) was not different between countries (p=0.73). Respondents who answered that they know of local guidelines (OR 2.55, 95% confidence interval [CI] 1.93-3.38, p<0.001) or international guidelines (OR 1.97, 95%CI 1.50-2.57, p<0.001) were more likely to answer with "definitely take BC" in the case scenario.

Theme: Training. The proportion of respondents who answered that there were no training, lectures, classes or meetings that provide knowledge about local/national/international guidelines for BC sampling in their hospitals was highest in Indonesia (37.8%, 153/407), followed by Thailand (24.9%, 64/257) and Vietnam (12.5%, 52/421, p<0.001). Respondents who answered that there are training, lectures, classes or meetings that provide knowledge about guidelines for BC sampling were more likely to answer with "definitely take BC" in the case scenario (OR 1.68; 95%CI 1.18-2.38, p=0.004).

TDF-Social influence

Theme: Norms of BC sampling. Most Thai respondents (78.5%, 233/297) answered that they order BC because they are following local norms all the time or often, while 51.5% (238/462) of Vietnamese respondents and 43.8% (180/411) of Indonesian respondents answered likewise (p<0.001). The respondents who answered that they order BC because they are following local norms were more likely to answer with "definitely take BC" in the case scenario (OR 2.20, 95%CI 1.67-2.90, p<0.001).

For peer review only - http://bmjopen.bmj.com/site/about/guidelines.xhtml

Theme: Influences from healthcare workers, patients and family of patients. Most respondents (79.4%) answered that there are very positive or positive influences on BC sampling from consultants, followed by residents (64.5%), doctors (64.6%), heads of department (65.9%), executive levels (50.6%), nurses (47.6%), interns (45.2%), patients (43.0%) and family of patients (31.9%). Some respondents said that there are negative or very negative influence in BC sampling from family of patients (6.8%), nurses (5.2%), patients (4.3%) and executives of the hospital (3.6%). Numerous quotes on this theme as a barrier were noted (Appendix S6).

TDF-Reinforcement

Theme: Consequences that discourage BC sampling. Some respondents (32.5%, 300/923) answered that, if they order a BC when it is recommended, there are either negative social consequences (e.g. verbal reprimand or any pressure from supervisors/executives of the hospital as the hospital (may) have to pay for the (extra) cost of BC) or negative material consequences (e.g. a negative score, that doctors are at risk of having to spend extra time and effort to reimburse the cost of BC from any health scheme or insurance, or that doctors are at risk of having to pay for the [extra] cost of BC themselves). The proportion of those who answered likewise was highest in Vietnam (42.2%, 153/363), followed by Thailand (27.0%, 60/222) and Indonesia (25.7%, 87/338). Those who answered that there are negative consequences were less likely to answer with “definitely take BC” in the case scenario (OR 0.48; 95%CI 0.34-0.67, $p<0.001$).

TDF-Behavioural regulation

For peer review only - http://bmjopen.bmj.com/site/about/guidelines.xhtml

Theme: Regulation of cost reimbursement. Some respondents stated that 'whether patients have a health scheme or insurance that covers the cost of BC' (15.0%, 196/1,308) and that 'whether patients are likely to have a final diagnosis that includes the cost of BC in the package of fee for service' are their additional reasons for deciding to order BC (11.6%, 152/1,308). Those respondents were not associated with answering with "definitely take BC" in the case scenario (p>0.20, both). However, numerous quotes on this theme were noted (Appendix S6).

Theme: Procedures to support or regulate doctors to order BC. Overall, the most common procedures to support or regulate doctors to order BC in respondents' hospitals were case reviews (e.g. grand rounds or morning ward rounds, and BC is often mentioned; 30.8%, 326/1,060), followed by standard order forms to remind ordering BC (29.9%, 317/1,060), stewardship programmes and reviewing BC is included in the programmes (19.5%, 207/1,060), posters (15.4%, 163/1,060) and computer systems to remind ordering BC (10.7%, 113/1,060). Respondents who answered that there were case reviews (OR 1.55, 95%CI 1.14-2.13, p=0.006) and stewardship programmes (OR 1.65, 95%CI 1.16-2.34, p=0.005) were more likely to answer with "definitely take BC" in the case scenario

TDF-Environmental context and resources

Theme: Perceived cost-effectiveness of BC. Most Vietnamese respondents (85.9%, 407/474) considered that BC is very likely or likely to be cost-effective, while 79.5% (232/292) of Thai respondents and 68.8% (311/452) of Indonesian respondents considered likewise. The respondents who considered that BC is cost-effective were more likely to answer with "definitely take BC" in the case scenario (OR 1.63, 95%CI 1.17-2.26, p<0.001).

For peer review only - http://bmjopen.bmj.com/site/about/guidelines.xhtml

Theme: Availability of microbiology laboratories, transport modalities, resources and consumables. Some respondents answered that they could not order BC because microbiology laboratories are not available or not functioning (13.4%, 157/1,174) or consumables (such as BC bottles, needles, syringes, blood collection set, etc.) are not available (12.7%, 150/1,181) all the time or often. Those respondents were not associated with answering with "definitely take BC" in the case scenario (p>0.20 both)

Theme: Out-of-pocket. About a quarter of Indonesian respondents (23.3%, 78/335) answered that patients have to pay for BC using their own money (i.e. out of pocket) all the time or often, while 12.2% (28/230) of Thai participant and 8.3% (34/408) of Vietnamese participant answered likewise (p<0.001). Those respondents were not associated with answering with "definitely take BC" in the case scenario (p=0.29).

Intervention types and policy options to improve BC sampling practice

We used the links between TDF, COM-B, and BCW, and listed all suggested intervention types and policy options related to very important TDF domains in Indonesia, Thailand and Vietnam (Table 3 and Appendix S8). A range of potential strategies were identified. Some strategies target individual reinforcement, environmental structure and social influence (e.g. providing an example for physicians to aspire to or imitate the BC sampling practice [Intervention type-modelling] and increasing means and reducing barriers to increase capability and opportunity for all levels of doctors to order BC [Intervention type-enablement]). Some strategies operate at the policy or service provision level (e.g. changing regulation of cost reimbursement [Policy option-

For peer review only - http://bmjopen.bmj.com/site/about/guidelines.xhtml

fiscal], development or implementation of local guidelines [Policy option-guideline] and establishing rules or principles of BC practice [Policy option-regulation]).

For peer review only

For peer review only - http://bmjopen.bmj.com/site/about/guidelines.xhtml

Table 3. Suggested intervention types and policy options to improve BC sampling practice based on very important TDF domains in Indonesia, Thailand and Vietnam

	COM-B components [1]				
	Psychological capability (TDF: knowledge, and behavioural regulation)	Reflective motivation (TDF: goals, beliefs about consequence, and intention)	Automatic motivation (TDF: reinforcement)	Physical opportunity (TDF: environmental context, and resources)	Social opportunity (TDF: social influence)
**Intervention types [2]**					
Education	√	√			
Persuasion		√	√		
Incentivisation		√	√		
Coercion		√	√		
Training	√				
Restriction				√	√
Environmental restructuring			√	√	√
Modelling			√		
Enablement	√		√	√	√
**Policy options [2]**					
Communication/marketing		√	√		
Guidelines	√	√	√	√	√
Fiscal	√	√	√	√	√
Regulation	√	√	√	√	√
Legislation	√	√	√	√	√
Environmental/social planning	√		√	√	√
Service provision	√	√	√	√	√

[1] COM-B component stands for Capability (Physical capability or Psychological capability), Opportunity (Physical opportunity or Social opportunity), Motivation (Automatic motivation or Reflective motivation)–Behaviour, represents source of the behaviours and is the core of the Behaviour Change Wheel (BCW). TDF = Theoretical domain framework. [2] Suggested intervention types and policy options were identified using the links between TDF, the components of the COM-B and the BCW.[16-18]

For peer review only

For peer review only - http://bmjopen.bmj.com/site/about/guidelines.xhtml

Discussion

Our study shows that barriers and enablers to BC sampling in Southeast Asia are varied and heterogenous. We consider that ‘priority of BC [TDF-goals]’, ‘perception about their role to order or initiate an order for BC [TDF-social professional role and identity]’, ‘intention to follow guidelines [TDF-intention]’, ‘norms of BC sampling [TDF-social influence]’, ‘consequences that discourage BC sampling [TDF-reinforcement]’ and ‘regulation on cost reimbursement [TDF-behavioural regulation]’ are key barriers/enablers. In Thailand,[10] where BC utilization rate is relatively high compared to Indonesia[9] and Vietnam,[11] the frequencies of each barrier (enabler) being reported by respondents is lower (higher) for most domains.

‘Priority to BC [TDF-goals]’, ‘perception about their role to order or initiate an order for BC [TDF-social professional role and identity]’, ‘intention to follow guidelines [TDF-intention]’ and ‘norms of BC sampling [TDF-social influence]’ are likely key barriers to BC sampling in both HICs and other LMICs where resources for BC sampling are available to some extent.[8,12-15]

To our knowledge, ‘priority of BC [TDF-goals]’, ‘level of doctors who can order or initiate an order for BC [TDF-social professional role and identity]’ and ‘influence from healthcare workers, patients and families of patients [TDF-social influence]’ have never been evaluated in LMICs.[8,12-15] Those are important barriers/enablers. ‘Priority of BC’ has the highest odds ratio for the association with “definitely take BC” in the case scenario in our study (OR 4.25). The importance of ‘priority of BC’ was previously reported from HICs.[13] In addition, in many hospitals in both HICs and LMICs, final-year medical students and interns are responsible for

For peer review only

most BC ordering and acquisition[29] and influences from other parties can discourage BC sampling.

Remarkably, the cost of BC seems to have influence on executive level doctors, patients, families of patients, medical doctors, and those who set regulations on cost reimbursement of BC. This is shown by many quotes related to the cost of BC in the theme ‘influences from healthcare workers, patients and family of patients [TDF-social influence]’, ‘consequences that discourage BC sampling [TDF-reinforcement]’, ‘perceived cost-effectiveness of BC [TDF-environmental context and resources]’ and ‘regulation on cost reimbursement [TDF-behavioural regulation]’ (Appendix S6). To improve diagnostic stewardship practices, all stakeholders will need to consider all suggested intervention types and policy options and develop intervention content based on local context.[16-18]. To overcome cost-related barriers, interventions such as providing clear posters emphasizing local guidelines for BC sampling over wide areas in hospitals to reduce negative influences from all parties on BC practice [Intervention type-environmental restructuring], changing regulation of cost reimbursement and finding financial support for BC sampling per local guidelines [Policy option-fiscal], repeatedly announcing to all levels of healthcare workers that negative consequences that discourage BC sampling per local guidelines will not be tolerated [Intervention type-enablement] could be considered and implemented.

Fear of ‘blood stealing’ or ‘blood selling’ is reported as a barrier to blood specimen collection in many countries in sub-Saharan Africa; including Kenya, Zambia, Mozambique, The Gambia, Tanzania and Uganda.[30] We observed fears of pain, needles, drawing a lot of blood, anaemia,

For peer review only

blood-transmitted diseases, etc. (Appendix S6), but did not observe fear of ‘blood stealing’ or ‘blood selling’. Emotional barriers to BC sampling are likely different depending on local regions.

This study has several limitations. First, we used a convenient sample of hospitals and practitioners, which might have led to selection bias. This limited our ability to draw definite conclusions on the contemporary situation on barriers/enablers to BC sampling in each country and in Southeast Asia. Second, the survey could not reach the target sample size in Thailand despite substantial efforts. Nonetheless, the study had enough power to estimate the prevalence of barriers and enablers in Thailand, and compare that with those observed in Indonesia and Vietnam. Third, the findings may not be generalizable to all LMICs because barriers and enablers to BC sampling can be varied and local evaluations are needed.

In conclusion, this comprehensive analysis using TDF gives information across the entire spectrum of behavioral influences of BC sampling. These results can help local healthcare providers and policy makers to develop and implement interventions aiming to improve diagnostic stewardship practices.

For peer review only

For peer review only - http://bmjopen.bmj.com/site/about/guidelines.xhtml

Declaration of interests

The authors declare no competing financial interests.

Data sharing

The data used will be made available on request to the corresponding author

Acknowledgments

The authors thank the respondents and staff at all the study hospitals and Mahidol-Oxford Tropical Medicine Research Unit. The authors thank Surveillance and Epidemiology of Drug-resistant Infections Consortium (SEDRIC) for the support and comments on the study. We thank Le Nguyen Minh Hoa, Vu Minh Duy, Dam Thi Hong Hanh and Hoang Bao Long for support in data collection.

Author contributions

F.L., L.A. and D.L. designed and supervised the study. P.S., K.S.A., R.L., V.T.L.H., H.R.v.D. and R.L.H. participated in project design and facilitated data collection. A.T., L.W.A.R., R.B., E.J.N., D.U.N., S.K., W.S., P.C., W.P., N.H.Y., P.N.T., L.M.Q., V.H.V., C.M.D., V.T.H.D.E. and E.H. facilitated data collection. P.S. analyzed the data and wrote the first draft of the manuscript. All authors contributed to the writing or revision of the manuscript. P.S. and D.L. verified the data.

For peer review only

Ethics Approval Statement

The study was approved by the Oxford University Tropical Research Ethics Committee (OXTREC545-21) and local ethical committees at Iskak Tulungagung Hospital (070/7303/407.206/2021), Prof. Dr. R.D. Kandou Hospital (156/EC/KEPK-KANDOU/IX/2021), Pasar Minggu Hospital (EOCRU/RCH.216/10.2021/1145) in Indonesia, The National Hospital for Tropical Diseases (14HDDD/NDTU) in Vietnam, Faculty of Tropical Medicine, Mahidol University (TMEC21-069), Sunpasitthiprasong Hospital (065/64S) and Chiangrai Prachanukroh Hospital (CR 0032.102/EC023) in Thailand. Electronic informed consent was obtained from study respondents prior to participation.

Funding.

This research was funded by the Wellcome Trust (220557/Z/20/Z). For the purpose of Open Access, the author has applied a CC BY public copyright licence to any Author Accepted Manuscript version arising from this submission.

For peer review only

References

1. Evans L, Rhodes A, Alhazzani W, et al. Surviving sepsis campaign: international guidelines for management of sepsis and septic shock 2021. *Intensive Care Med* 2021; **47**(11): 1181-247.

2. Schuts EC, Hulscher M, Mouton JW, et al. Current evidence on hospital antimicrobial stewardship objectives: a systematic review and meta-analysis. *Lancet Infect Dis* 2016; **16**(7): 847-56.

3. WHO. Diagnostic stewardship: a guide to implementation in antimicrobial resistance surveillance sites. 2016. https://apps.who.int/iris/handle/10665/251553

4. Singer M, Deutschman CS, Seymour CW, et al. The Third International Consensus Definitions for Sepsis and Septic Shock (Sepsis-3). *JAMA* 2016; **315**(8): 801-10.

5. Warren BG, Yarrington ME, Polage CR, Anderson DJ, Moehring RW. Evaluation of hospital blood culture utilization rates to identify opportunities for diagnostic stewardship. *Infect Control Hosp Epidemiol* 2022: 1-6.

6. Chen AI, Bilker WB, Hamilton KW, O'Donnell JA, Nachamkin I. Blood culture utilization at an academic hospital: Addressing a gap in benchmarking. *Infect Control Hosp Epidemiol* 2018; **39**(11): 1353-9.

7. Cassini A, Hogberg LD, Plachouras D, et al. Attributable deaths and disability-adjusted life-years caused by infections with antibiotic-resistant bacteria in the EU and the European Economic Area in 2015: a population-level modelling analysis. *Lancet Infect Dis* 2019; **19**(1): 56-66.

8. WHO. Central Asian and Eastern European Surveillance of Antimicrobial Resistance. Annual Report 2018. 2018.

For peer review only - http://bmjopen.bmj.com/site/about/guidelines.xhtml

https://www.euro.who.int/__data/assets/pdf_file/0007/386161/52238-WHO-CAESAR-AR-2018_low_V11_web.pdf

9. Sinto R, Lie KC, Setiati S, et al. Blood culture utilization and epidemiology of antimicrobial-resistant bloodstream infections before and during the COVID-19 pandemic in the Indonesian national referral hospital. *Antimicrob Resist Infect Control* 2022; **11**(1): 73.

10. Teerawattanasook N, Tauran PM, Teparrukkul P, et al. Capacity and Utilization of Blood Culture in Two Referral Hospitals in Indonesia and Thailand. *Am J Trop Med Hyg* 2017; **97**(4): 1257-61.

11. Takeshita N, Anh NQ, Phuong DM, et al. Assessment of Bacteremia in a Large Tertiary Care Hospital in Northern Vietnam: a Single-Center Retrospective Surveillance Study. *Jpn J Infect Dis* 2019; **72**(2): 118-20.

12. She RC, Alrabaa S, Lee SH, Norvell M, Wilson A, Petti CA. Survey of physicians' perspectives and knowledge about diagnostic tests for bloodstream infections. *PLoS One* 2015; **10**(3): e0121493.

13. Raupach-Rosin H, Duddeck A, Gehrlich M, et al. Deficits in knowledge, attitude, and practice towards blood culture sampling: results of a nationwide mixed-methods study among inpatient care physicians in Germany. *Infection* 2017; **45**(4): 433-41.

14. Ojide CK, Onwuezobe IA, Asuquo EE, Obiagwu CS. Knowledge, attitude and practice of blood culture: A cross sectional study among medical doctors in a nigerian tertiary hospital. *African Journal of Clinical and Experimental Microbiology* 2013; **14**(3): 174-9.

15. Chew KS, Mohd Hashairi F, Jusoh AF, Aziz AA, Nik Hisamuddin NA, Siti Asma H. Knowledge of good blood culture sampling practice among healthcare staffs in an emergency department - Are we getting it right? *Medical Journal of Malaysia* 2013; **68**(4): 323-5.

For peer review only - http://bmjopen.bmj.com/site/about/guidelines.xhtml

16. Michie S, van Stralen MM, West R. The behaviour change wheel: a new method for characterising and designing behaviour change interventions. *Implement Sci* 2011; **6**: 42.

17. Cane J, O'Connor D, Michie S. Validation of the theoretical domains framework for use in behaviour change and implementation research. *Implement Sci* 2012; **7**: 37.

18. Atkins L, Francis J, Islam R, et al. A guide to using the Theoretical Domains Framework of behaviour change to investigate implementation problems. *Implementation Science* 2017; **12**(1): 77.

19. McDonagh LK, Saunders JM, Cassell J, et al. Application of the COM-B model to barriers and facilitators to chlamydia testing in general practice for young people and primary care practitioners: a systematic review. *Implement Sci* 2018; **13**(1): 130.

20. Sargent L, McCullough A, Del Mar C, Lowe J. Using theory to explore facilitators and barriers to delayed prescribing in Australia: a qualitative study using the Theoretical Domains Framework and the Behaviour Change Wheel. *BMC Fam Pract* 2017; **18**(1): 20.

21. Lohiniva AL, Heweidy I, Girgis S, et al. Developing a theory-based behavior change intervention to improve the prescription of surgical prophylaxis. *Int J Clin Pharm* 2021.

22. Smith JD, Corace KM, MacDonald TK, et al. Application of the Theoretical Domains Framework to identify factors that influence hand hygiene compliance in long-term care. *J Hosp Infect* 2019; **101**(4): 393-8.

23. Mazza D, Chapman A, Michie S. Barriers to the implementation of preconception care guidelines as perceived by general practitioners: a qualitative study. *BMC Health Serv Res* 2013; **13**: 36.

For peer review only

For peer review only - http://bmjopen.bmj.com/site/about/guidelines.xhtml

24. Suntornsut P, Wongsuwan N, Malasit M, et al. Barriers and Recommended Interventions to Prevent Melioidosis in Northeast Thailand: A Focus Group Study Using the Behaviour Change Wheel. *PLoS Negl Trop Dis* 2016; **10**(7): e0004823.

25. Wong E, Mavondo F, Horvat L, McKinlay L, Fisher J. Healthcare professionals' perspective on delivering personalised and holistic care: using the Theoretical Domains Framework. *BMC Health Serv Res* 2022; **22**(1): 281.

26. Huijg JM, Gebhardt WA, Crone MR, Dusseldorp E, Presseau J. Discriminant content validity of a theoretical domains framework questionnaire for use in implementation research. *Implementation Science* 2014; **9**(1): 11.

27. Eng J. Sample size estimation: how many individuals should be studied? *Radiology* 2003; **227**(2): 309-13.

28. Patey AM, Islam R, Francis JJ, Bryson GL, Grimshaw JM, Canada PPT. Anesthesiologists' and surgeons' perceptions about routine pre-operative testing in low-risk patients: application of the Theoretical Domains Framework (TDF) to identify factors that influence physicians' decisions to order pre-operative tests. *Implement Sci* 2012; **7**: 52.

29. Parada JP, Schwartz DN, Schiff GD, Weiss KB. Effects of type and level of training on variation in physician knowledge in the use and acquisition of blood cultures: A cross sectional survey. *BMC Infectious Diseases* 2005; **5**

30. Peeters Grietens K, Ribera JM, Erhart A, et al. Doctors and vampires in sub-Saharan Africa: ethical challenges in clinical trial research. *Am J Trop Med Hyg* 2014; **91**(2): 213-5.

For peer review only

Appendices: Barriers and enablers to blood culture sampling in Indonesia, Thailand and Vietnam

Appendix S1: Supplementary Text

Supplementary Methods

The healthcare systems in SEA are highly diverse.[2] In 2020, Indonesia (GDP per capita: 3,869.6 US$) and Vietnam (GDP per capita: 2,785.7 US$) were a lower-middle-income country and Thailand (GDP per capita:7,186.9 US$) was an upper-middle-income country in SEA.[1] Indonesia has a decentralised public healthcare system, in which provincial or district-level governments have the authority over most public hospitals, and a substantial private health sector. To achieve the goal of universal healthcare coverage (UHC), in 2014 the Government introduced national health insurance (Jaminan Kesehatan Nasional), which had reached 84% of the population by 2021. Thailand achieved the status of UHC in 2002 in terms of insurance entitlement, when the gross national income per capita was 1,900 US$.[3] It is shown that UHC in Thailand can improve quality of care without undermining the efficiency and equity of the policy.[4] Vietnam has implemented social health insurance (SHI) since 1992, and SHI had a role as a financial mechanism towards achieving UHC,[5] which had reached 82% of the population in 2018. The benefit package of universal SHI in Vietnam is considered generous, particularly regarding the drugs subsidized.[5] However, out-of-pocket payments are still high.[5,6] In 2019, percentages of out-of-pocket expenditure among all health expenditure were 35%, 9% and 43% in Indonesia, Thailand and Vietnam, respectively.[7]

Analysis

We explored the agreement between two themes of the TDF domain reinforcement. The degree of agreement between responses to the questions for barriers/enablers was estimated using the Kappa index. This describes the level of association, both positive and negative, beyond that caused by chance, as follows: 0.00–0.20, slight; 0.21–0.40, fair; 0.41–0.60, moderate; 0.61–0.80, substantial; 0.81–1.00, high.

Additional analysis

For peer review only - http://bmjopen.bmj.com/site/about/guidelines.xhtml

We explored whether the answers of respondents who completed the survey were different from the answers of respondents who did not complete the survey. We compared the answers to the case scenario between those who completed the questionnaire and those who answered the case scenario (Question 1-3 in the questionnaire) but did not complete the questionnaire. Logistic regression model with random effects for countries was used for the analysis.

Supplementary Results

Additional results and the content themes in the domains that were identified as key domains are described in further detail in the sections below.

Belief about consequences

Theme: Perceived that BC is helpful. Most respondents strongly agreed or agreed that BC is helpful in adjusting antibiotics (94.0%, 1,224/1,302), clinical decisions (93.6%, 1,220/1,303), detecting AMR bacterial infections (92.1%, 1,199/1,302) and ruling in an infection (90.2%, 1,172/1,299), reducing overuse of antibiotics (87.4%, 1,140/1,304), reducing patient mortality (79.2%, 1,027/1,297). 72.3% (938/1,298) of respondents strongly agreed or agreed that BC is helpful in reducing length of hospital stay. 60.5% (786/1,300) of respondents strongly agreed or agreed that BC is helpful in ruling out an infection. Most respondents strongly agreed or agreed that accumulative results of BC are helpful in understanding epidemiology of AMR bacterial infections (94.5%, 1,228/1,299).

Respondents who answered that BC is helpful in clinical decisions (OR 2.96, 95%CI 1.71-5.12, $p<0.001$), to reduce patient mortality (OR 1.61; 95%CI 1.18-2.20, $p=0.003$), to rule in an infection (OR 1.58, 95%CI 1.04-2.39, $p=0.03$), or to reduce length of hospital stay (OR 1.53, 95%CI, 1.14-2.04, $p=0.004$) and those who answered that accumulative results of BC are helping in understanding epidemiology of AMR bacterial infections (OR 2.89, 95%CI 1.60-5.19, $p<0.001$) were more likely to answer with “definitely take BC” in the case scenario. Respondents who answered that BC is helpful to rule out an infection, to detecting AMR bacterial infection, in adjusting antibiotics, or reduce overuse of antibiotics were not associated with answering with “definitely take BC” in the case scenario ($p>0.10$, all).

For peer review only - http://bmjopen.bmj.com/site/about/guidelines.xhtml

Theme: Perceived that BC is unnecessary. Some respondents strongly agreed or agreed that BC is unnecessary because it is not too late to collect BC later, particularly if patients do not improve after receiving empirical antibiotic treatment (32.7%, 423/1,293), the therapeutic consequence of BC sampling is questionable (18.6%, 238/1,277), antibiotic therapy can be determined based on clinical presentations (17.5%, 228/1,301), results are often delayed (17.0%, 220/1,298) quality of laboratory is questionable (15.3%, 194/1,269), the scientific basis of the guideline on BC is questionable (15.0%, 191/1,277), results are often negative or no growth (11.4%, 148/1,295), and results are often contaminated (11.1%, 143/1,288).

Respondents who answered that BC is unnecessary because antibiotic therapy can be determined based on clinical presentation (OR 0.51, 95%CI 0.36-0.73, $p<0.001$), the scientific basis of the guideline on BC is questionable (OR 0.66, 95%CI 0.45-0.98, $p=0.04$), results are often delayed (OR 0.48, 95%CI 0.33-0.69, $p<0.001$), results are often not interpretable (OR 0.54, 95%CI 0.34-0.87, $p=0.01$), results are often negative or no growth (OR 0.58, 95%CI 0.39-0.88, $p=0.01$), cultures are often contaminated (OR 0.64, 95%CI 0.42-0.98, $p=0.04$), BC is not benefiting the patients (OR 0.37; 95%CI 0.24-0.57, $p<0.001$), a contaminated result often leads to wrong therapeutic approach (OR 0.53; 95%CI 0.30-0.95, $p=0.03$), it is not too late to collect BC later, particularly if patients do not improve after receiving empirical antibiotic treatment (OR 0.37; 95%CI 0.27-0.52, $p<0.001$), quality of laboratory is questionable (OR 0.48; 95%CI 0.33-0.70, $p<0.001$) or levels of local antibiotic resistance are low (OR 0.64; 95%CI 0.41-0.98, $p=0.04$) were less likely to answer with “definitely take BC” in the case scenario. Respondents who answered that BC is unnecessary because the therapeutic consequence of BC is questionable, or results often do not agree with clinical signs were not associated with answering with “definitely take BC” in the case scenario ($p>0.20$, both).

Reinforcement

Theme: Consequences that encourage BC sampling. Some respondents (23.7%, 294/1,243) answered that there are either positive social (e.g. praise) or positive material (e.g. a positive score) consequences if they order a BC when it is recommended. Those respondents were less likely to answer with “definitely take BC” in the case scenario (OR 0.53; 95%CI 0.37-0.74,

For peer review only - http://bmjopen.bmj.com/site/about/guidelines.xhtml

p<0.001). We explored and found that respondents who answered that there are positive consequences that encourage BC sampling when recommended were also answered that there are negative consequences that discourage BC sampling when recommended with moderate agreement beyond that expected by chance (Kappa value 0.46, p<0.001).

We also evaluated whether they are negative consequences if practitioners do not order a BC when it is recommended. Some respondents (37.7%, 464/1,230) answered that there are either negative social (e.g. verbal reprimand) or negative material (e.g. a negative score) consequences if they do not order a BC when it is recommended. Those respondents were not associated with answering with "definitely order BC" in the case scenario (p=0.42).

Additional results and the content themes in the domains that were not identified as key domains are described in further detail in the sections below.

Emotion

Theme: Fear or anxiety of healthcare providers and *Fear or anxiety of patients or family of patients.* Some respondents (7.1%, 93/1,308) stated that there are emotional factors associated with ordering BC. Those include fear or anxiety related to pain, needles, blood-borne diseases, high volume of blood being drawn, anaemia, etc. (Table 3). Those respondents were not associated with answering "definitely take BC" in the case scenario (p=0.82). Numerous quotes on this theme as a barrier were noted (Appendix S6).

Optimism

Theme: Optimism about the laboratory. Most (80.5%, 1,034/1,285) respondents answered that they are strongly optimistic or optimistic that a BC will be sampled and processed in the laboratory appropriately if they order a BC. Respondents who were strongly optimistic or optimistic about the laboratory were more likely to answer with "definitely take BC" in the case scenario (OR 1.78, 95%CI 1.29-2.46, p<0.001)

Skills

For peer review only - http://bmjopen.bmj.com/site/about/guidelines.xhtml

Theme: Skills in drawing blood for BC. Among respondents whom were tasked to draw blood from patients for BC in their hospitals, 44.1% (143/324) answered that their skill of drawing blood from patients for BC is very good or good, 44.8% (145/324) fair, and 11.1% (36/324) poor or very poor. Respondents who answered that they have very good or good skill in drawing blood for BC was more likely to answer with "definitely take BC" in the case scenario (OR 1.74; 95%CI 1.02-2.07, p=0.04).

Memory, attention and decision processes

Theme: Patients who are already on antibiotics or have anemia. Some respondents (10.2%, 131/1,287) stated that they will definite or likely not order BC when patients are already on antibiotics even if BC is recommended. Those respondents were not associated with answering with "definitely take BC" in the case scenario (p=0.13). Some respondents (22.3%, 280/1,258) answered that they will definite or likely not order BC when patients have anemia even if BC is recommended. Those respondents were not associated with answering with "definitely take BC" in the case scenario (p=0.55).

Theme: Clinical presentations for deciding to order BC. Among respondents who responded that they know of local guidelines, some stated that patients with no clinical improvement after receiving empirical antibiotics (36.2%, 274/756), presenting with fever of unknown origin (30.6%, 231/756), suspected of hospital-acquired infection (30.8%, 233/756), presenting with chronic fever (28.6%, 216/756) or suspected of infection caused by antimicrobial-resistant organisms (28.6%, 216/756) are their additional reasons to order BC.

Belief about capabilities

Theme: Belief in their own capability to draw blood. Most respondents (73.9%, 244/358) answered that they are strongly confident or confident that they can draw BC successfully. Those respondents were not associated with answering with "definitely take BC" in the case scenario (p=0.36). Most respondents (74.8%, 246/329) also answered that they are strongly confident or confident that they can draw BC appropriately using aseptic technique. Those respondents were not associated with answering with "definitely take BC" in the case scenario (p=0.11).

For peer review only - http://bmjopen.bmj.com/site/about/guidelines.xhtml

Theme: Belief in capability of those who are tasked to draw blood. Most respondents (88.5%, 1,151/1,300) answered that they are strongly confident or confident that those who are tasked to draw BC can draw BC successfully. Those respondents were not associated with answering with "definitely take BC" in the case scenario (p=0.13). Most respondents (76.7%, 996/1,298) also answered that they are strongly confident or confident that those who are tasked to draw BC can draw BC appropriately using aseptic technique. Those respondents were not associated with answering with "definitely take BC" in the case scenario (p=0.23).

Additional analysis

We explored whether there was any evidence showing a difference between respondents who completed and did not complete the survey. Of 2,095 respondents who agreed to participate the online survey, 1,308 (62.4%) completed the questionnaire, 256 (12.2%) answered the question about the case-study (Question 1-3) but did not completed the questionnaire, and 531 (25.3%) did not answer the question about the case-study. The proportion of patients who answered that they would definitely take BC for the case scenario was not different between those who completed the questionnaire (52.1%; 682/1,308) and those who answered the question about the case scenario but did not complete the questionnaire (51.2%; 131/256) (p=0.08).

For peer review only

Appendix S2. Theoretical Domains Framework: Definitions and examples

TDF domain and definition	Examples related to blood culture sampling
**TDF-1 Knowledge:** awareness of the existence of something	In the context of this study, knowledge of the condition/scientific rationale could relate to their knowledge of:<br>• when and whom blood culture (BC) should be sampled<br>• local and international guidelines for BC sampling<br><br>Knowledge may be both correct and incorrect
**TDF-2 Skills:** ability or proficiency acquired through practice	In the context of this study, skills/competence include skill of participant to draw blood for BC sample collection.<br><br>Skills may be both present and absent
**TDF-3 Social professional role and identity:** a coherent set of behaviours and displayed personal qualities of an individual in a social or work setting	In the context of this study, professional role may relate to the extent that healthcare professionals feel that ordering or initiating an order for BC are part of their professional role or their job description.<br><br>Personal identity may relate to how a participant views their role of<br>• ordering or initiating an order for BC<br>• drawing blood for BC
**TDF-4 Beliefs about capabilities:** acceptance of the truth/reality about or validity of an ability, talent or facility that a person can put to constructive use	In the context of this study, beliefs about capabilities relates to the judgments on medical doctor/final-year medical student's ability to:<br>• draw blood successfully<br>• draw blood appropriately<br><br>As BC may be ordered by respondents but collected by other professionals, beliefs about capabilities also include their judgments on the ability of persons who are tasked to draw blood<br><br>"Successfully" means obtaining blood, and "Appropriately" means that general guidelines for BC specimen collection such as aseptic technique are followed.

For peer review only - http://bmjopen.bmj.com/site/about/guidelines.xhtml

TDF domain and definition	Examples related to blood culture sampling
**TDF-5 Optimism:** confidence that things will happen for the best or that desired goals will be attained	In the context of this study, optimism related to their judgment regarding that a BC will be sampled and processed in the laboratory appropriately if they order a BC.<br><br>This includes optimism and pessimism.
**TDF-6 Beliefs about consequences*:*** acceptance of the truth/reality about or validity of outcomes of a behaviour in a given situation	In the context of this study, beliefs about their judgments on:<br>• the purpose, value, and effectiveness of BC<br>• negative/positive outcomes of BC
**TDF-7 Reinforcement:** increasing the probability of a response by arranging a dependent relationship, or contingency, between the response and a given stimulus	In the context of this study, reinforcements relate to their judgments on:<br>• receiving an incentive or reward (these can be social [e.g. praise] or material [e.g. a positive score]) for ordering a BC when recommended<br>• receiving any negative consequences (these can be social [e.g. verbal reprimand or that you/doctors are at risk of being scrutinized] or material [e.g. a negative score]) for not ordering BC when recommended<br><br>As feedbacks could discourage the behavior, reinforcement also include judgements on:<br>• receiving any negative consequences for ordering BC when recommended
**TDF-8 Intentions:** conscious decision to perform a behaviour or a resolve to act in a certain way	In the context of this study, intentions relate to the statements on their intention to order BC.
**TDF-9 Goals:** mental representation of outcomes or end states that an individual wants to achieve	In the context of this study, goals relate to the statements on:<br>• the goals they wish to collect BC prior to giving empirical antibiotics<br>• competing goals (goals that might conflict with BC collection; e.g. giving empirical antibiotics)
**TDF-10 Memory, attention and decision processes:** ability to retain information, focus selectively on aspects of the environment and choose between two or more alternatives	In the context of this study, memory, attention and decision processes relate the statements on how they decide whether to order or not order BC

For peer review only

For peer review only - http://bmjopen.bmj.com/site/about/guidelines.xhtml

TDF domain and definition	Examples related to blood culture sampling
**TDF-11 Environmental context and resources:** any circumstances of a person's situation or environment that discourages or encourages the development of skills and abilities, independence, social competence, and adaptive behaviour	In the context of this study, environmental context and resources relates to their perceptions of the:<br>• Availability of consumables such as bottles, needles, syringes, blood collection set, etc.<br>• Availability of microbiology laboratories<br>• Financial resources, whether patients have to pay out-of-pocket<br>• Cost-effectiveness of BC
**TDF-12 Social influences:** interpersonal processes that can cause an individual to change their thoughts, feeling or behaviours.	In the context of this study, social influences relate to the statements expressing the influence of others on attending BC. Including:<br>• norms<br>• influences from nurses, other medical doctors, consultants, head of department, executive of the hospitals, patients and family of patients<br><br>"Norms" mean usual practice that are typical of or accepted within their hospital.
**TDF-13 Emotion:** a complex reaction pattern, involving experiential, behavioural and physiological elements, by which the individual attempts to deal with a personally significant matter or event	In the context of this study, emotions relate to the statements of expressing their emotional reaction/state relating to order and sample for BC<br><br>Any logical reasons or social influence which are stated as "fear of" are categorized as "Memory, attention and decision processes" or "Social influence" as appropriate.
**TDF-14 Behavioural regulation:** anything aimed at managing or changing objectively observed or measured actions	In the context of this study, behavioural regulation relates to the statements about managements or steps taken to<br>• order BC<br>• adopt local/national/international guidelines for BC sampling

For peer review only - http://bmjopen.bmj.com/site/about/guidelines.xhtml

Appendix S3. TDF survey questionnaire

Online research participant information sheet and electronic consent form

You are invited to participate in a web-based online survey on **"Barriers and facilitators to ordering blood culture samples in Indonesia, Thailand and Viet Nam".** This is a research project being conducted under the collaboration between Eijkman Oxford Clinical Research Unit (EOCRU), **Indonesia,** and Mahidol Oxford Tropical Medicine Research Unit (MORU), Faculty of Tropical Medicine, Mahidol University, **Thailand**, Oxford University Clinical Research Unit (OUCRU), **Viet Nam,** Centre for Behaviour Change, University College London, **United Kingdom**.

PROPOSE: This study aim to identify barrier and facilitators to the adoption of blood culture sampling recommendations in Indonesia, Thailand and Viet Nam

PARTICIPATION: The participants include 1,500 medical doctors and final-year medical students in Indonesia, Thailand and Viet Nam (500 participants per country). The survey is voluntary. You may refuse to take part in the research or exit the survey at any time without penalty. You are free to decline to answer any particular question you do not wish to answer for any reason.

PROCEDURE: You may have received an invitation from clinical directors, head of final-year medical student, or head of recently graduated medical doctors to do this online survey. You may also receive two email reminders about the invitation. We also ask final-year medical students and medical doctors in those hospitals to share the invitation to the survey to any final-year medical students and medical doctors in the country using their networks such as Facebook, Line and WhatsApp application.

In this survey, we will ask whether you know of any local and international guidelines on when to perform blood culture sampling, whether you would perform blood culture sampling for the constructed case scenario, and why you do or do not perform blood culture sampling. It should take approximately 30 – 40 minutes to complete.

All study data will be entered on a Qualtrics. The participants will be identified by a unique study specific number and/or code in any database. We will ask for your email account or telephone number in order to provide you an electronic gift. You may refuse to providing your email account or telephone number and to receiving an electronic gift. The name and any other identifying detail will NOT be included in any study data electronic file.

BENEFITS: You will receive no direct benefits from participating in this research study. However, your responses may help us learn more about what are barriers and facilitators of doctors to order and collect blood culture samples per local, national or international recommendations in different countries. The questionnaire focuses only on when and why blood culture is sampled. Participants will receive a gift or cash (about $4 USD in value) for completing the questionnaire. Participants could receive the gift electronically if email account or telephone number is provided.

RISKS: There is the risk that you may find some of the questions to be sensitive, and that some questions may cause emotional discomfort. Nonetheless, the possible risks or discomforts of the study are minimal. If you feel uncomfortable or distressed at any time during this survey, you should feel free to terminate participation. You are free to decline to answer any particular question you do not wish to answer for any reason. The study team does not expect any risks for participants beyond the minimal risks described above regarding confidentiality surrounding sensitive comments that might arise when participating in the qualitative interviews.

WITHDRAWAL: The survey is voluntary. You can withdraw from the study without penalty at any time and you are free to decline to answer any particular question you do not wish to answer for any reason with no obligation to give the reason for withdrawal.

For peer review only - http://bmjopen.bmj.com/site/about/guidelines.xhtml

CONFIDENTIALITY: Although we will collect your identifying information such as your medical license number (student identification no if you are a medical student), email address and telephone number, your identifying information are needed for compensation and your identifying information will be known only to the researchers performing this study or to specific groups for auditing purposes (if requested). These groups are government institutions or organisations authorised to conduct audits such as the ethics committee. Only summary results will be published and anonymous information will be put in open-access scientific database. No one will be able to identify you or your answers, and no one will know whether you participated in the study.

ETHICAL: The study protocol, informed consent form, participant information sheet and any proposed advertising material will be submitted to OxTREC, the ethics Committee of the Faculty of Tropical Medicine, Mahidol University, Thailand and (FTMEC), and local ethics committees for written approval.

CONTACT: If you have questions at any time about the study or the procedures, you may contact Dr Ralalicia Limato (rlimato@eocru.org) in Indonesia, Pornpan Suntornsut (pornpan@tropmedres.ac) in Thailand, and Dr Vu Thi Lan Huong (huongvtl@oucru.org) in Viet Nam.

DATA PROTECTION: The University of Oxford is responsible for ensuring the safe and proper use of any personal information you provide, solely for research purposes.

DATA SHARING: Data collected for this study will be de-identified and may be shared with other groups of researchers in accordance with the current MORU Data Sharing Policy. All applications will be carefully reviewed by the MORU Data Access Committee before granting any approvals to access data. All researchers accessing the data need to adhere to a set of terms and conditions that aim to protect the interests of research participants and other relevant stakeholders.

INTERNET AND DEVICE REQUIREMENT: This online questionnaire requires good internet connection and relatively up-to-date devices. Mobile devices with small screens may not show the questions clearly. If your devices are relatively out-of-date or with small screens, we recommend you to use a desktop computer at a place with good internet connection. If you have a problem with the online questionnaire, you may ask for the word file (.doc) or the paper questionnaire by contacting Dr Ralalicia Limato (rlimato@eocru.org) in Indonesia, Pornpan Suntornsut (pornpan@tropmedres.ac) in Thailand, and Dr Vu Thi Lan Huong (huongvtl@oucru.org) in Viet Nam.

ELECTRONIC CONSENT: Please select your choice below. You may print a copy of this consent form for your records. Clicking on the "Agree" button indicates that I agree to participate in the research study. I have read the above information and I am participating voluntarily.

○ Agree
○ Disagree

EXPLANATION: The questionnaire may contain ○ for radio button (can take only one answer) ☐ for multiple choices (can take more than one answer)) and open text answer as well. Please indicate your level of opinion and mark in the button or box of your answer.

Q1-1. **At which type of hospital are you currently working?** If you are currently working at more than one hospital, select where you are currently spending most time. (please select the most relevant answer)

○ Government hospital (including National hospital, Provincial hospital, District hospital)
○ Private hospital
○ University hospital
○ I do not want to answer

For peer review only - http://bmjopen.bmj.com/site/about/guidelines.xhtml

o Other: ……………
Q1-2. **What is your Medical license number or student ID number?** This is to confirm that you are a medical doctor or a final-year medical student in Indonesia, Thailand or Viet Nam. If you are not a medical doctor or a final-year medical student in Indonesia, Thailand or Viet Nam, you should not participate in this questionnaire. Your identifying information will be known only to the researchers. No one will be able to identify you or your answers, and no one will know whether you participated in the study.<br>…………………………………………………………………
Q1-3. **As an introduction to the topic blood culture sampling, we present a case scenario to you. We would like to know if you consider taking blood culture samples in your everyday clinical practice and your current hospital setting.**<br><br>**If you are currently working at more than one hospital, please consider the hospital you are spending most time as your current hospital setting.**<br><br>**case scenario.** "A 72-year-old woman who was brought to the emergency department of your hospital by her daughter when she noticed the patient was more confused than her baseline and was found to have a high fever and fast breathing. She had an auscultatory finding compatible with pneumonia. It is decided that this patient will be admitted to your hospital."<br><br>If you have an authority to take a blood culture, would you take blood culture sample(s) in this case on admission?<br><br>o Definitely (>95-100% of the time)<br>o Likely (75-95% of the time)<br>o Maybe (25-74% of the time)<br>o Unlikely (5-24% of the time)<br>o Rarely (ranging from never <5% of the time)<br>o I do not know<br>o I do not want to answer
Q1-4. Do you know of any recommendation(s) or guideline(s) for blood culture sampling being used in your hospital?<br><br>o Yes<br>o No, my hospital does not use any recommendations or guidelines for blood culture sampling (go to Q1-8)<br>o I do not know if my hospital uses any recommendations or guidelines. (go to Q1-8)<br>o I do not want to answer (go to Q1-8)
(Page break)
Q1-5. **Based on your understanding**, do any following statement(s) represent the recommendation(s) or guideline(s) for blood culture sampling being used in your hospital? (you can select more than one answer)<br><br>☐ Recommend blood culture sampling in all patients presenting with SIRS (Systemic inflammatory Response Syndrome [SIRS] is defined as having at least two of the following criteria: fever or hypothermia, tachycardia, tachypnea, and leukocytosis or leucopenia)<br>☐ Recommend blood culture sampling in all patients presenting with sepsis ('sepsis' here is defined as an acute change in total Sequential Organ Failure Assessment [SOFA] score ≥2 points consequent to the infection based on the most recent definition of sepsis [Sepsis-3 criteria])<br>☐ Recommend blood culture sampling in all patients presenting with septic shock<br>☐ Recommend blood culture sampling in all patients starting parenteral antibiotic treatment<br>☐ Recommend blood culture sampling in all patients with no clinical improvement after receiving empirical antibiotics

For peer review only - http://bmjopen.bmj.com/site/about/guidelines.xhtml

☐ Recommend blood culture sampling in all patients presenting with infection and having underlying diseases
☐ Recommend blood culture sampling in all patients with chronic fever
☐ Recommend blood culture sampling in all patients with fever of unknown origins
☐ Recommend blood culture sampling in all patients suspected of infections caused by atypical organisms
☐ Recommend blood culture sampling in all patients suspected of infections caused by antimicrobial-resistant organisms
☐ Recommend blood culture sampling in all patients suspected of infections caused by multiple-drug-resistant organisms
☐ Recommend blood culture sampling in all patients suspected of hospital-acquired infections
☐ I do not know
☐ I do not want to answer
☐ Other: ……………

Due to many factors, there are times that doctors can not follow the recommendation(s) or guideline(s).

Q1-6. In your current hospital setting, how often do you plan to follow the recommendation(s) or guideline(s) for blood culture sampling being used in your hospital?

○ All the time (>95-100% of the cases)
○ Often (75-95% of the cases)
○ Moderately (25-74% of the cases)
○ Occasionally (5-24% of the cases)
○ Rarely (ranging from never to <5% of the cases)
○ I do not know
○ I do not want to answer

Q1-7. Apart from the recommendation(s) or guideline(s) being used at your hospital (as you answered in the previous question), do you have **any additional reasons** for deciding to do blood culture sampling? (you can select more than one answers that are applicable to your current hospital setting)

☐ No. All reasons are stated in the recommendation(s) or guideline(s) being used in my hospital.
☐ Patients presenting with chills
☐ Patients presenting with sepsis
☐ Patients presenting with septic shock
☐ Patients starting parenteral antibiotic treatment
☐ Patients with no clinical improvement after receiving empirical antibiotics
☐ Patients presenting with infection and having underlying diseases
☐ Patients presenting with chronic fever
☐ Patients presenting with fever of unknown origin
☐ Patients suspected of infections caused by atypical organisms
☐ Patients suspected of infections caused by antimicrobial-resistant organisms
☐ Patients suspected of infections caused by multiple-drug-resistant organisms
☐ Patients suspected of hospital-acquired infections
☐ Laboratory results showing leukocytosis
☐ Laboratory results showing neutropenia
☐ Laboratory results showing left shift in blood count (i.e. showing immature white blood cells)
☐ Laboratory results showing CRP increase
☐ Laboratory results showing procalcitonin increase
☐ Patients can afford the cost of blood culture
☐ Patients have a health scheme or insurance that covers the cost of blood culture

For peer review only - http://bmjopen.bmj.com/site/about/guidelines.xhtml

☐ Patients are likely to have a final diagnosis that includes the cost of blood culture in the package of fee for service<br>☐ I do not know<br>☐ I do not want to answer<br>☐ Other: …………..<br><br>(Skip to Q1-9 after this question)
(Page break)
Q1-8. In your current hospital setting, what are the **reasons** for deciding to do blood culture sampling? (you can select more than one answer that are applicable for your current hospital setting)<br><br>☐ Patients presenting with chills<br>☐ Patients presenting with sepsis<br>☐ Patients presenting with septic shock<br>☐ Patients presenting with infection and having underlying diseases<br>☐ Patients starting parenteral antibiotic treatment<br>☐ Patients with no clinical improvement after receiving empirical antibiotics<br>☐ Patients presenting with infection and having underlying diseases<br>☐ Patients presenting with chronic fever<br>☐ Patients presenting with fever of unknown origin<br>☐ Patients suspected of infections caused by atypical organisms<br>☐ Patients suspected of infections caused by antimicrobial-resistant organisms<br>☐ Patients suspected of infections caused by multiple-drug-resistant organisms<br>☐ Patients suspected of hospital-acquired infections<br>☐ Laboratory results showing leukocytosis<br>☐ Laboratory results showing neutropenia<br>☐ Laboratory results showing left shift in blood count<br>☐ Laboratory results showing CRP increase<br>☐ Laboratory results showing procalcitonin increase<br>☐ Patients can afford the cost of blood culture<br>☐ Patients have a health scheme or insurance that covers the cost of blood culture<br>☐ Patients are likely to have a final diagnosis that includes the cost of blood culture in the package of fee for service<br>☐ I do not know<br>☐ I do not want to answer<br>☐ Other: …………..
(Page break)
Q1-9. Are you aware of any international recommendation(s) or guideline(s) for blood culture sampling? Examples of international recommendations are surviving sepsis campaign (SSC), the diagnostic stewardship of the World Health Organization (WHO), The Infectious Diseases Society of America (IDSA) and The National Institute for Health and Care Excellence (NICE)<br><br>○ Yes<br>○ No (go to Q2-1)<br>○ I do not want to answer (go to Q2-1)

For Peer review only

Q1-10. **Based on your understanding**, can any following statement(s) represent international recommendation(s) for blood culture sampling (you can select more than one answers)<br><br>☐ Recommend collecting blood culture in all patients presenting with sepsis<br>☐ Recommend collecting blood culture in all patients starting parenteral antibiotic treatment<br>☐ I do not know<br>☐ I do not want to answer<br>☐ Other:……………………
(Page break)
**We would like to understand your current job and how doctors in different positions are involved in ordering and collecting blood culture in your current hospital setting.**<br><br>Q2-1. First, please state your current job. (please select the most relevant answer)<br>○ Medical doctor – working in an executive or administrative position (not doing clinical work)<br>○ Medical doctor – working as a consultant (defined as a doctor with a clinical specialty/subspecialty degree)<br>○ Medical doctor – working as a physician (defined as a doctor without a clinical specialty/subspecialty degree and not under any postgraduate clinical training)<br>○ Medical doctor – working as a resident/registra/fellow (defined as a doctor who is currently under any postgraduate clinical training)<br>○ Intern (defined as a recent medical school graduate who is in the first year of post-graduate on-the-job training)<br>○ Final-year medical student<br>○ Other:……………
Final-year medical students (and interns) in some countries or some settings can **initiate an order** for a blood culture under authority of residents, consultants or other medical doctors. The order may be supervised, signed or co-signed by residents, consultants or other medical doctors later.<br><br>Q2-2. In your current hospital setting, which types of professionals/staff **can order** a blood culture. **"Order"** means initiating an order either verbally or in writing. (you can select more than one answers)<br><br>☐ Medical doctors – working in executive or administrative positions (not doing clinical work)<br>☐ Medical doctors – working as consultants (defined as a doctor with a clinical specialty/subspecialty degree)<br>☐ Medical doctors – working as physicians (defined as a doctor without a clinical specialty/subspecialty degree and not under any postgraduate clinical training)<br>☐ Medical doctors – working as residents/registras/fellows (defined as a doctor who is currently under any postgraduate clinical training)<br>☐ Interns (defined as recent medical school graduates who are in the first year of post-graduate on-the-job training)<br>☐ Final-year medical students<br>☐ I do not want to answer<br>☐ Other:……………
Q2-3. Do you know when and which patients should receive an **order** for a blood culture in your hospital?<br><br>○ Definitely (>95-100% of the case)<br>○ Likely (75-95% of the case)<br>○ Uncertain (25-74% of the case)<br>○ Unlikely (5-24% of the case)<br>○ Rarely (ranging from never to <5% of the case)<br>○ I do not know

For peer review only - http://bmjopen.bmj.com/site/about/guidelines.xhtml

○ I do not want to answer
Q2-4. If you **can order** for a blood culture as per your current job description or position, do you think that it is an appropriate part of your current job (as per your job description or position) to **order** a blood culture?<br><br>○ Very appropriate<br>○ Appropriate<br>○ Uncertain<br>○ Inappropriate<br>○ Very inappropriate<br>○ I cannot order blood culture. It is not part of my job (Go to Q2-5).<br>○ I do not know<br>○ I do not want to answer<br><br>(Skip to Q2-6 after this question, except answering "I cannot order blood culture. It is not part of my job")
(Page break)
Q2-5. As you **cannot order** for a blood culture as per your current job description or position, do you think that it would be an appropriate part of your current job (as per your job description or position) to **order** a blood culture?<br><br>○ Very appropriate<br>○ Appropriate<br>○ Uncertain<br>○ Inappropriate<br>○ Very inappropriate<br>○ I do not know<br>○ I do not want to answer
(Page break)
Q2-6. In your current hospital setting, which **types of professionals** are tasked to **draw blood** from patients for blood culture. (you can select more than one answers)<br><br>☐ Medical doctors – working in executive or administrative positions (not doing clinical work)<br>☐ Medical doctors – working as consultants (defined as a doctor with a clinical specialty/subspecialty degree)<br>☐ Medical doctors – working as physicians (defined as a doctor without a clinical specialty/subspecialty degree and not under any postgraduate clinical training)<br>☐ Medical doctors – working as residents/registras/fellows (defined as a doctor who is currently under any postgraduate clinical training)<br>☐ Interns (defined as recent medical school graduates who are in the first year of post-graduate on-the-job training)☐ Interns<br>☐ Final-year medical students<br>☐ Registered nurses<br>☐ Microbiology laboratory team<br>☐ Specialized blood draw team<br>☐ I do not want to answer<br>☐ Other:……………
Q2-7. Do you think that it is an appropriate part of your job (as per your job description or position) to **draw blood**?

For peer review only

o Very appropriate<br>o Appropriate<br>o Uncertain<br>o Inappropriate<br>o Very inappropriate<br>o It is not part of my job to draw blood from patients for blood culture (go to Q2-11)<br>o I do not know<br>o I do not want to answer
(Page break)
Q2-8. How skilled are you in **drawing blood**?<br>o Very good skill<br>o Good skill<br>o Fair skill<br>o Poor skill<br>o Very poor skill<br>o I do not know<br>o I do not want to answer
**Having confidence is different from having skills. Due to many factors, there are times that blood could not be drawn even though we are skilled.**<br><br>Q2-9. If you have to draw blood yourself, are you confident that **you can draw blood successfully**? "Successfully" means obtaining blood.<br>o Strongly confident<br>o Confident<br>o Uncertain<br>o Doubtful<br>o Strongly doubtful<br>o It is not part of my job to draw blood from patients for blood culture<br>o I do not know<br>o I do not want to answer
Q2-10. Are you confident that **you can draw blood appropriately**? "Appropriately" means that general recommendations for blood culture specimen collection such as aseptic technique are followed.<br>o Strongly confident<br>o Confident<br>o Uncertain<br>o Doubtful<br>o Strongly doubtful<br>o It is not part of my job to draw blood from patients for blood culture<br>o I do not know<br>o I do not want to answer
(Page break)
Q2-11. Are you confident that **others (who are tasked to draw blood in your hospital) can draw blood successfully**?<br>o Strongly confident<br>o Confident<br>o Uncertain

For peer review only - http://bmjopen.bmj.com/site/about/guidelines.xhtml

o Doubtful<br>o Strongly doubtful<br>o I do not know<br>o I do not want to answer<br>o I do not want to answer
Q2-12. Are you confident that **others (who are tasked to draw blood in your hospital) can draw blood appropriately**? "Appropriately" means that general recommendations for blood culture specimen collection such as aseptic technique are followed.<br><br>o Strongly confident<br>o Confident<br>o Uncertain<br>o Doubtful<br>o Strongly doubtful<br>o I do not know<br>o I do not want to answer
Q2-13. In your current hospital setting, how **optimistic** are you that a blood culture will be sampled and processed in the laboratory appropriately if you order a blood culture? "Optimistic" means the confidence that things will happen for the best or that desired goals will be attained.<br><br>o Strongly optimistic<br>o Optimistic<br>o Neither optimistic nor pessimistic<br>o Pessimistic<br>o Strongly pessimistic<br>o I do not know<br>o I do not want to answer
(Page break)

Many advantages and disadvantages of blood culture have been mentioned in surveys in different countries. This advantages and disadvantages could differ between settings.

Please answer of all following question to the best of your ability. Please a check mark "V " in the appropriate answer for each question.

Q3-1. Do you agree or disagree about the following potential advantages of blood culture, making blood culture helpful in your current hospital setting?	Strongly agree	Agree	Uncertain	Disagree	Strongly disagree	I do not know	I do not want to answer
• Blood culture is helpful in clinical decisions.							
• Blood culture is helpful to rule in an infection.							
• Blood culture is helpful to rule out an infection.							
• Blood culture is helpful in detecting antimicrobial-resistant bacterial infections.							
• Blood culture is helpful in adjusting antibiotics.							
• Blood culture can reduce overuse of antibiotics.							
• Blood culture can reduce length of hospital stay.							

For peer review only - http://bmjopen.bmj.com/site/about/guidelines.xhtml

• Blood culture can reduce patient mortality.							
• Accumulative results of blood culture (i.e. antimicrobial-resistance surveillance report) are helpful in understanding epidemiology of antimicrobial-resistant bacterial infections.							

Q3-2. Additional comments why blood culture is helpful in your current hospital setting (Note: limit to 2,000 characters)
………………………………………………………………

Please answer of all following question to the best of your ability. Please a check mark "√ " in the appropriate answer for each question.

Q3-3. Do you agree or disagree about the following disadvantages of blood culture, **making blood culture unnecessary in your current hospital setting?**	Strongly agree	Agree	Uncertain	Disagree	Strongly disagree	I do not know	I do not want to answer
• Blood culture is unnecessary because antibiotic therapy can be determined based on clinical presentations.							
• The therapeutic consequence of blood culture sampling is questionable.							
• The scientific basis of the guideline on blood culture is questionable							
• Blood culture is unnecessary because results are often delayed.							
• Blood culture is unnecessary because results are often not interpretable.							
• Blood culture is unnecessary because results are often negative or no growth.							
• Blood culture is unnecessary because cultures are often contaminated.							
• Blood culture is unnecessary because results often do not agree with clinical signs.							
• Blood culture is unnecessary because a contaminated result often leads to wrong therapeutic approaches.							
• Blood culture is unnecessary because it is too expensive.							
• Blood culture is not benefiting the patients.							
• It is not too late to collect blood culture later, particularly if patients do not improve after receiving empirical antibiotic treatment.							
• Quality of laboratory is questionable.							
• Levels of local antibiotic resistance are low.							

Q3-4. Additional comments why blood culture is not helpful in your current hospital setting (Note: limit to 2,000 characters)
………………………………………………………………

(Page break)

In different settings, other tasks may be considered more urgent than collecting blood culture samples.

Q3-5. In your current hospital setting, how often do you obtain blood culture **prior to administration of empirical antibiotics** in patients presenting with **sepsis**? '('sepsis' here is defined as an acute change in total Sequential Organ Failure Assessment [SOFA] score ≥2 points consequent to the infection based on the most recent definition of sepsis [Sepsis-3 criteria])

○ All the time (>95-100% of the time)

For peer review only - http://bmjopen.bmj.com/site/about/guidelines.xhtml

○ Often (75-95% of the time)
○ Moderately (25-74% of the time)
○ Occasionally (5-24% of the time)
○ Rarely (ranging from never to <5% of the time)
○ I do not know
○ I do not want to answer

Q3-6. In your current hospital setting, how often do you obtain blood culture **prior to administration of empirical antibiotics** in patients presenting with **septic shock**?
○ All the time (>95-100% of the time)
○ Often (75-95% of the time)
○ Moderately (25-74% of the time)
○ Occasionally (5-24% of the time)
○ Rarely (ranging from never to <5% of the time) ○ Rarely (ranging from never to <5% of the time)
○ I do not know
○ I do not want to answer

Even if blood culture is recommended, doctors may decide not to order blood culture in some situations.

Please answer of all following question to the best of your ability. Please a check mark "√ " in the appropriate answer for each question.

Q3-7. Would you still order blood culture in the following situation?	Definitely not order	Likely not order	Maybe not order	Likely to still order	Very likely to still order	I do not Know	I do not want to answer
• Patients are already on antibiotics.							
• Patients have anemia.							
• Blood should be used for other laboratory tests.							
• There are no local guidelines/recommendations for blood culture sampling							
• Patients do not meet certain conditions for a blood culture following the local guidelines							
• Patients do not have a health scheme or insurance that covers the cost of blood culture							
• Microbiology laboratory in your hospital is not available							

Q3-8. Additional comments why you do not order blood culture regarding situations mentioned above (Note: limit to 2,000 characters)
………………………………………………………………

(Page break)

Resources are commonly limited in many settings worldwide.

Q4-1. In your hospital, how often could you (or doctors in your hospital) **not order blood culture** because consumables (such as blood culture bottles, needles, syringes, blood collection set, etc.) are **not available**?

○ All the time (>95-100% of the time)
○ Often (75-95% of the time)
○ Moderately (25-74% of the time)

For peer review only - http://bmjopen.bmj.com/site/about/guidelines.xhtml

○ Occasionally (5-24% of the time)
○ Rarely (ranging from never to <5% of the time)
○ I do not know
○ I do not want to answer

Q4-2. In your hospital, how often could you (or doctors in your hospital) **not order blood culture** because the microbiology laboratory is **not available** or not functioning?

○ All the time (>95-100% of the time)
○ Often (75-95% of the time)
○ Moderately (25-74% of the time)
○ Occasionally (5-24% of the time)
○ Rarely (ranging from never to <5% of the time)
○ I do not know
○ I do not want to answer

Q4-3. In your hospital, how often do patients have to pay for blood culture using their own money (i.e. out of pocket)?

○ All the time (>95-100% of the patients)
○ Often (75-95% of the patients)
○ Moderately (25-74% of the patients)
○ Occasionally (5-24% of the patients)
○ Rarely (ranging from never to <5% of the patients)
○ I do not know I do not know
○ I do not want to answer

Q4-4. Regardless of who pays for the cost of blood culture, would you say that the benefits of blood culture outweigh the cost?

○ Very likely
○ Likely
○ Uncertain
○ Unlikely
○ Very unlikely
○ I do not know
○ I do not want to answer

(Page break)

Positive and negative consequences could encourage us to follow guidelines.

Q5-1. Are there **any positive consequences, incentives or rewards** (these can be social [e.g. praise] or material [e.g. a positive score]) if you or doctors in your hospital **order a blood culture when recommended**? (you can select more than one answer)

☐ No
☐ Yes- social
☐ Yes- material
☐ Yes- both social and material
☐ I do not know
☐ I do not want to answer

For peer review only

☐ Other: ....

Q5-2. Are there **any negative consequences** to you or doctors (these can be social [e.g. verbal reprimand or that you/doctors are at risk of being scrutinized] or material [e.g. a negative score]) if you or doctors in your hospital **do not order a blood culture when recommended**? (you can select more than one answer)

☐ No
☐ Yes- social
☐ Yes- material
☐ Yes- both social and material
☐ I do not know
☐ I do not want to answer
☐ Other: ....

Sometimes there are feedbacks that could discourage us to follow guidelines. This could be due to many reasons based on local context.

Q5-3. Are there **any negative consequences** to you or doctors (these can be social [e.g. verbal reprimand or any pressure from your supervisors/executives of your hospital as the hospital (may) have to pay for the (extra) cost of blood culture] or material [e.g. a negative score, that you/doctors are at risk of having to spend extra time and effort to reimburse the cost of blood culture from any health scheme or insurance, or that you/doctors are at risk of having to pay for the (extra) cost of blood culture yourselves]), if you or doctors in your hospital **order blood culture when recommended**? (you can select more than one answer)

☐ No
☐ Yes- social
☐ Yes- material
☐ Yes- both social and material
☐ I do not know
☐ I do not want to answer
☐ Other: ....

Q5-4. Additional comments about feedbacks (including encouragement, punishments or any positive and negative consequences) on blood culture sampling in your hospital setting. Also, please provide more comments about whether any consequences you would recommend to implement in your hospital to support blood culture ordering.

....

(Page break)

Q5-5. In your hospital, are there **any training, lectures, classes or meetings** that provide you knowledge about local/national/international guidelines for blood culture sampling? (you can select more than one answers)

☐ No
☐ Yes, infrequently (less than once a year)
☐ Yes, occasionally (at least once a year)
☐ Yes, regularly (more than once a year)
☐ I do not know
☐ I do not want to answer

For peer review only - http://bmjopen.bmj.com/site/about/guidelines.xhtml

☐ Other: ....

Q5-6. In your hospital, are there **any procedures** that support you or doctors in your hospital to order or regulate ordering of blood culture per local/national/international guidelines? (you can select more than one answers)

☐ No
☐ Yes, there is a poster (and blood culture is mentioned)
☐ Yes, there is a standard order form for patients presenting with sepsis (and blood culture is already written in the order form)
☐ Yes, there is a computer system to remind ordering blood culture
☐ Yes, there is a case review (e.g. grand round; morning ward round, clinical meetings, etc and blood culture is often mentioned)
☐ Yes, there is a stewardship programme and reviewing blood culture is included in the programme (e.g. post-prescription review and stewardship round, etc.)
☐ Yes, there is a local hospital guideline (e.g. standard operating procedure [SOP])
☐ I do not know
☐ I do not want to answer
☐ Other: ....

(Page break)

Due to different personal beliefs, norms and limitations, blood culture sampling is encouraged or discouraged by peers and co-workers in different settings.

Q6-1. **To what extent do you or doctors in your hospital order blood culture sampling because you are following local norms? "**Norms" mean usual practice that are typical of or accepted within your hospital.

○ All the time (>95-100% of the time)
○ Often (75-95% of the time)
○ Moderately (25-74% of the time)
○ Occasionally (5-24% of the time)
○ Rarely (ranging from never to <5% of the time)
○ I do not know
○ I do not want to answer

Please answer of all following question to the best of your ability. Please a check mark "V " in the appropriate answer for each question.

Q6-2. **Do following people have any positive or negative influence on you or doctors in your hospital to order blood culture?** Positive influence could mean facilitate, support or encourage blood culture sampling. Negative influence could mean hinder or discourage blood culture sampling.	Very positive influence	Positive influence	Neither positive nor negative influence	Negative influence	Very negative influence	I do not know	I do not want to answer
• Nurses							
• Final-year medical students							
• Interns							
• Residents (any postgraduate clinical training)							

For peer review only - http://bmjopen.bmj.com/site/about/guidelines.xhtml

• Doctors (defined as a doctor without a specialty/subspecialty degree and not under any postgraduate clinical training)							
• Consultants (defined as a doctor with a clinical specialty/subspecialty degree)							
• Head of the Department							
• Executives of the hospital							
• Patients							
• Family of patients							

Q6-3. Additional comments about social influence on blood culture sampling
……………………………………………………………………

Q6-4. Apart from your logical considerations, do you think that **any emotional factors** of anyone involved in ordering and sampling for blood culture (including patients and family of patients) could influence whether blood culture is ordered or sampled? (for example: fear of blood, fear of needle, fear of blood transmitted diseases, etc)

○ No
○ Other: ……………

Q6-5. Additional comments about emotional factors (from anyone involved in ordering and sampling for blood culture; including patients and family of patients) on blood culture sampling
……………………………………………………………………

(Page break)

Finally, we have some questions about yourself

Q7-1. Which country do you currently work in?

○ Thailand
○ Vietnam
○ Indonesia
○ I do not want to answer

Province of your current hospital:…………………………………… (Dropdown list for each country)

Q7-2. Are you female or male?

○ Female
○ Male
○ Other
○ I do not want to answer

For peer review only - http://bmjopen.bmj.com/site/about/guidelines.xhtml

Q7-3. What is the number of beds in your hospital? (Please use the official number, and please estimate if you are uncertain.)

○ < 200
○ 201 - 400
○ 401 - 600
○ 601 - 1,000
○ 1,001 - 2,000
○ > 2,000
○ I do not know
○ I do not want to answer

Q7-4. In which department are you **currently working**? If your role (such as medical students) moves from one department to another department over time, please state the current department you are working in.
(you can select more than one answers; for example both internal medicine and infectious disease devision)

☐ Internal Medicine
☐ Pediatrics
☐ Infection disease division/department
☐ Surgery
☐ Orthopaedics
☐ Obstetrics / Gynaecology
☐ Emergency department
☐ Intensive care unit
☐ I do not want to answer
☐ Other: ……………

(Page break)

Q7-5. Do you want to be contacted for further studies?

○ Yes
○ No

Q7-6. Do you want to be informed the results of this study?

○ Yes
○ No

Q7-7. Your email address (If you want to be contacted via email address. Please leave it blank, if you do not want to be contact via email address)
………………………………………………………………

Q7-8. Your phone number (if you want to be contacted via phone. Please leave it blank, if you do not want to be contact via phone)
………………………………………………………………

Please note that a gift or cash (about $4 in value) for completing the survey is to be provided to you. Participants could receive the gift electronically if email account or telephone number is provided.

For peer review only

Please make sure that you click "submit" on the next page to complete the questionnaire. Otherwise, all answers that you made and your information for compensation will not be submitted to us via the system.
(Page break)
We are grateful for your participation. Thank you very much.

For peer review only

For peer review only - http://bmjopen.bmj.com/site/about/guidelines.xhtml

Appendix S4. Criteria and rank of TDF domains

**TDF domains**	(1) 'frequency' or the proportion of respondents who perceived the importance or agreement with a barrier/enabler within each domain *	(2) 'elaboration' or number of themes within each domain **	(3) 'expressed importance' or quotes from respondents expressing importance or agreement with a barrier/enabler within each domain ***	(4) 'association between reported barriers or enablers and BC practice' or size of effect and strength of association, i.e. odds ratio [OR] and p values, obtained from the logistic regression model, respectively ****	Overall rank *****
Goals	Moderate (25-74%)	1	A few quotes	OR 4.25, strongly associated	Very important
Social professional role and identity	High (75-95%)	3	A few quotes	OR 3.36, strongly associated	Very important
Beliefs about consequences	High (75-95%)	2	A number of quotes	OR 2.96, strongly associated	Very important
Intentions	Moderate (25-74%)	1	A few quotes	OR 2.92, strongly associated	Very important
Knowledge	Moderate (25-74%)	2	A few quotes	OR 2.55, strongly associated	Very important
Social influences	Moderate (25-74%)	2	A number of quotes	OR 2.20, strongly associated	Very important
Reinforcement	Moderate (25-74%)	2	A number of quotes	OR 0.48, strongly associated	Very important
Behavioural regulation	Moderate (25-74%)	2	A number of quotes	OR 1.65, strongly associated	Very important
Environmental context and resources	High (75-95%)	3	A number of quotes	OR 1.63, strongly associated	Very important
Emotion	Low (5-24%)	2	A number of quotes	Not observed	Important
Optimism	High (75-95%)	1	None	OR 1.78, strongly associated	Important
Skills	Moderate (25-74%)	1	None	OR 1.74, associated	Important
Memory, attention and decision processes	Moderate (25-74%)	2	A few quotes	Not observed	Important
Beliefs about capabilities	Moderate (25-74%)	2	None	Not observed	Important

* For each question, we defined that respondents who answered "definitely"/"likely", "all the time"/"often" or "strongly agree"/"agree" perceived the importance or agreement with that barrier/enabler. The highest proportion for a barrier/enabler in each domain is presented. Details are present in the Appendix S5 ** Details are presented in the main manuscript and in the Appendix S1 *** Details are presented in the Appendix S6. **** Size of effect (OR) and p values were considered.[40,41] P values <0.05 was not used as a simple cutoff whether an association was present or absent. P values less than 0.001 was regarded as providing strong evidence against the null hypothesis. For a negative association (OR<1.0), the inversed OR (1/OR) was considered as the size effect when compared with other positive associations. Details are present in the Appendix S7 ***** Overall rank was decided based on detailed presentation of the ratings of each criterion.

For peer review only - http://bmjopen.bmj.com/site/about/guidelines.xhtml

Appendix S5. Survey results

Questions	Indonesia (n=503)	Thailand (n=304)	Viet Nam (n=501)	P value
**Type of hospitals (Q1-1)**				
Government hospital	340 (67.6%)	209 (68.8%)	431 (86.0%)	<0.001
Private hospital	113 (22.5%)	15 (4.9%)	17 (3.4%)	
University hospital	26 (5.2%)	76 (25.0%)	29 (5.8%)	
Other[1]	19 (3.8%)	2 (0.7%)	22 (4.4%)	
I do not want to answer	5 (1.0%)	2 (0.7%)	2 (0.4%)	
**Case-study: Would you take BC sample from a hypothetical sepsis case? (Q1-3)**				
Definitely (>95-100% of the time)	157 (31.2%)	273 (89.8%)	252 (50.3%)	<0.001
Likely (75-95% of the time)	138 (27.4%)	23 (7.6%)	149 (29.7%)	
Maybe (25-74% of the time)	116 (23.1%)	5 (1.6%)	70 (14.0%)	
Unlikely (5-24% of the time)	44 (8.7%)	2 (0.7%)	19 (3.8%)	
Rarely (ranging from never <5% of the time)	46 (9.1%)	1 (0.3%)	9 (1.8%)	
I do not know	1 (0.2%)	0 (0%)	1 (0.2%)	
I do not want to answer	1 (0.2%)	0 (0%)	1 (0.2%)	
**Knowledge (TDF-1): Do you know of any guideline(s) or guideline(s) used in my hospital (Q1-4)?**				
Yes	240 (47.7%)	169 (55.6%)	347 (69.3%)	<0.001
No, my hospital does not have any	68 (13.5%)	33 (10.9%)	49 (9.8%)	
No, I do not know if my hospital uses any	183 (36.4%)	98 (32.2%)	95 (19.0%)	
I do not want to answer	12 (2.4%)	4 (1.3%)	10 (2.0%)	
**Knowledge (TDF-1): known local guideline among those who answered that they know of local guideline (Q1-5)**				
All patients presenting with SIRS	155/240 (64.6%)	147/169 (87.0%)	218/347 (62.8%)	<0.001
All patients presenting with sepsis	183/240 (76.2%)	138/169 (81.7%)	291/347 (83.9%)	0.07
All patients presenting with septic shock	147/240 (61.3%)	131/169 (77.5%)	270/347 (77.8%)	<0.001
All patients starting parenteral antibiotic treatment	92/240 (38.3%)	92/169 (54.4%)	73/347 (21.0%)	<0.001
All patients with no clinical improvement after receiving empirical antibiotics	141/240 (58.7%)	99/169 (58.6%)	160/347 (46.1%)	0.003
All patients presenting with infection and having underlying diseases	76/240 (31.7%)	61/169 (36.1%)	94/347 (27.1%)	0.10
All patients with chronic fever	97/240 (40.4%)	87/169 (51.5%)	208/347 (59.9%)	<0.001
All patients with fever of unknown origins	114/240 (47.5%)	100/169 (59.2%)	185/347 (53.3%)	0.06
All patients suspected of infections caused by atypical organisms	97/240 (40.4%)	74/169 (43.8%)	94/347 (27.1%)	<0.001
All patients suspected of infections caused by antimicrobial-resistant organisms	131/240 (54.6%)	96/169 (56.8%)	168/347 (48.4%)	0.14
All patients suspected of infections caused by multiple-drug-resistant organisms	136/240 (56.7%)	103/169 (60.9%)	194/347 (55.9%)	0.54
All patients suspected of hospital-acquired infections	116/240 (48.3%)	99/169 (58.6%)	184/347 (53.0%)	0.12
**Intention (TDF-8): How often do you plan to follow the local guideline among those who answered that they know of local guideline (Q1-6)?**				
All the time (>95-100% of the cases)	70/240 (29.2%)	76/169 (45.0%)	88/347 (25.4%)	<0.001

For peer review only - http://bmjopen.bmj.com/site/about/guidelines.xhtml

Questions	Indonesia (n=503)	Thailand (n=304)	Viet Nam (n=501)	P value
Often (75-95% of the cases)	102/240 (42.5%)	81/169 (47.9%)	195/347 (56.2%)	
Moderately (25-74% of the cases)	33/240 (13.8%)	11/169 (6.5%)	49/347 (14.1%)	
Occasionally (5-24% of the cases)	16/240 (6.7%)	0/169 (0%)	11/347 (3.2%)	
Rarely (ranging from never <5% of the cases)	11/240 (4.6%)	1/169 (0.6%)	2/347 (0.6%)	
I do not know	7/240 (2.9%)	0/169 (0%)	2/347 (0.6%)	
I do not want to answer	1/240 (0.4%)	0/169 (0%)	0/347 (0%)	
**Memory, attention and decision processes (TDF-10): any additional reasons for deciding to do BC among those who answered that they know of local guideline (Q1-7)?**				
No additional reasons	77/240 (32.1%)	35/169 (20.7%)	110/347 (31.7%)	0.02
Patients presenting with chills	15/240 (6.3%)	39/169 (23.1%)	23/347 (6.6%)	<0.001
Patients presenting with sepsis	102/240 (42.5%)	101/169 (59.8%)	113/347 (32.6%)	<0.001
Patients presenting with septic shock	86/240 (35.8%)	96/169 (56.8%)	139/347 (40.1%)	<0.001
Patients starting parenteral antibiotic treatment	48/240 (20.0%)	59/169 (34.9%)	35/347 (10.1%)	<0.001
Patient with no clinical improvement after receiving empirical antibiotics	102/240 (42.5%)	75/169 (44.4%)	97/347 (28.0%)	<0.001
Patients with infection and having underlying diseases	42/240 (17.5%)	36/169 (21.3%)	56/347 (16.1%)	0.35
Patients presenting with chronic fever	54/240 (22.5%)	55/169 (32.5%)	107/347 (30.8%)	0.04
Patients presenting with fever of unknown origin	72/240 (30.0%)	63/169 (37.3%)	96/347 (27.7%)	0.08
Patients suspected of infections caused by atypical organisms	52/240 (21.7%)	46/169 (27.2%)	48/347 (13.8%)	0.001
Patients suspected of infections caused by antimicrobial-resistant organisms	77/240 (32.1%)	53/169 (31.4%)	86/347 (24.8%)	0.10
Patients suspected of infections caused by multiple-drug-resistant organisms	82/240 (34.2%)	63/169 (37.3%)	92/347 (26.5%)	0.03
Patients suspected of hospital-acquired infections	77/240 (32.1%)	59/169 (34.9%)	97/347 (28.0%)	0.24
Laboratory results showing leukocytosis	29/240 (12.1%)	42/169 (24.9%)	25/347 (7.2%)	<0.001
Laboratory results showing neutropenia	36/240 (15.0%)	54/169 (32.0%)	28/347 (8.1%)	<0.001
Laboratory results showing left shift in blood count	31/240 (12.9%)	26/169 (15.4%)	14/347 (4.0%)	<0.001
Laboratory results showing CRP increase	37/240 (15.4%)	22/169 (13.0%)	42/347 (12.1%)	0.51
Laboratory results showing procalcitonin increase	55/240 (22.9%)	22/169 (13.0%)	94/347 (27.1%)	0.002
Patients can afford the cost of BC	25/240 (10.4%)	9/169 (5.3%)	32/347 (9.2%)	0.18
Patients have a health scheme or insurance that covers the cost of BC	24/240 (10.0%)	8/169 (4.7%)	26/347 (7.5%)	0.14
Patients are likely to have a final diagnosis that includes the cost of BC in the package of fee for service	18/240 (7.5%)	0/169 (0%)	25/347 (7.2%)	0.001
**Memory, attention and decision processes (TDF-10): any reasons for deciding to do BC among those who did not answer that they know of local guideline (Q1-8)?**				
Patients presenting with chills	20/263 (7.6%)	49/135 (36.3%)	29/154 (18.8%)	<0.001
Patients presenting with sepsis	188/263 (71.5%)	132/135 (97.8%)	109/154 (70.8%)	<0.001
Patients presenting with septic shock	165/263 (62.7%)	128/135 (94.8%)	135/154 (87.7%)	<0.001
Patients starting parenteral antibiotic treatment	48/263 (18.3%)	95/135 (70.4%)	26/154 (16.9%)	<0.001

Questions	Indonesia (n=503)	Thailand (n=304)	Viet Nam (n=501)	P value
Patient with no clinical improvement after receiving empirical antibiotics	188/263 (71.5%)	119/135 (88.1%)	84/154 (54.5%)	<0.001
Patients with infection and having underlying diseases	85/263 (32.3%)	79/135 (58.5%)	52/154 (33.8%)	<0.001
Patients presenting with chronic fever	91/263 (34.6%)	89/135 (65.9%)	108/154 (70.1%)	<0.001
Patients presenting with fever of unknown origin	138/263 (52.5%)	110/135 (81.5%)	100/154 (64.9%)	<0.001
Patients suspected of infections caused by atypical organisms	123/263 (46.8%)	81/135 (60.0%)	55/154 (35.7%)	<0.001
Patients suspected of infections caused by antimicrobial-resistant organisms	177/263 (67.3%)	108/135 (80.0%)	85/154 (55.2%)	<0.001
Patients suspected of infections caused by multiple-drug-resistant organisms	183/263 (69.6%)	113/135 (83.7%)	85/354 (24.0%)	<0.001
Patients suspected of hospital-acquired infections	136/263 (51.7%)	107/135 (79.3%)	78/154 (50.6%)	<0.001
Laboratory results showing leukocytosis	41/263 (15.6%)	52/135 (38.5%)	15/154 (9.7%)	<0.001
Laboratory results showing neutropenia	34/263 (12.9%)	59/135 (43.7%)	18/154 (11.7%)	<0.001
Laboratory results showing left shift in blood count	47/263 (17.9%)	47/135 (34.8%)	16/154 (10.4%)	<0.001
Laboratory results showing CRP increase	59/263 (22.4%)	23/135 (17.0%)	26/154 (16.9%)	0.27
Laboratory results showing procalcitonin increase	73/263 (27.8%)	28/135 (20.7%)	53/154 (34.4%)	0.04
Patients can afford the cost of BC	81/263 (30.8%)	18/135 (13.3%)	32/154 (20.8%)	<0.001
Patients have a health scheme or insurance that covers the cost of BC	88/263 (33.5%)	19/135 (14.1%)	31/154 (20.1%)	<0.001
Patients are likely to have a final diagnosis that includes the cost of BC in the package of fee for service	51/263 (19.4%)	0/135 (0%)	30/154 (19.5%)	<0.001
**Knowledge (TDF-1): Do you know of any international guideline(s) or guideline(s) (Q1-9)?**				
Yes	229 (45.5%)	142 (46.7%)	225 (44.9%)	<0.001
No	263 (52.3%)	156 (51.3%)	233 (46.5%)	
I do not want to answer	11 (2.2%)	6 (2.0%)	43 (8.6%)	
**Knowledge (TDF-1): known international guideline or guideline among those who answered that they know of any international guideline(s) or guideline(s) (Q1-10)**				
BC sampling in all patients presenting with sepsis	220/229 (96.1%)	138/142 (97.2%)	208/225 (92.4%)	0.08
BC sampling in all patients starting parenteral antibiotic treatment	125/229 (54.6%)	87/142 (61.3%)	147/225 (65.3%)	<0.001
**Professional role (Q2-1): Current job**				
Medical doctor – an executive level	13 (2.6%)	5 (1.6%)	17 (3.4%)	<0.001
Medical doctor – a consultant level	74 (14.7%)	75 (24.7%)	198 (39.5%)	
Medical doctor – a general physician level	124 (24.7%)	38 (12.5%)	112 (22.4%)	
Medical doctor – a resident/registra/fellow level	168 (33.4%)	63 (20.7%)	101 (20.2%)	
Intern – recent medical school graduate	33 (6.6%)	35 (11.5%)	14 (2.8%)	
Final-year medical student	91 (18.1%)	88 (28.9%)	59 (11.8%)	
**Professional role (Q2-2): Which types of professionals/staff can order or initiate an order for a BC?**				

Questions	Indonesia (n=503)	Thailand (n=304)	Viet Nam (n=501)	P value
Medical doctor – an executive level	61 (12.1%)	163 (53.6%)	59 (11.8%)	<0.001
Medical doctor – a consultant level	431 (85.7%)	250 (82.2%)	439 (87.6%)	0.11
Medical doctor – a general physician level	265 (52.7%)	240 (78.9%)	347 (69.3%)	<0.001
Medical doctor – a resident (postgrad training) level	268 (53.3%)	242 (79.6%)	317 (63.3%)	<0.001
Intern – a recent medical school graduate level	83 (16.5%)	231 (76.0%)	118 (23.6%)	<0.001
Final-year medical student	11 (2.2%)	87 (28.6%)	3 (0.6%)	<0.001
I do not want to answer	3 (0.6%)	1 (0.3%)	11 (2.2%)	0.03
Other	0 (0%)	0 (0%)	0 (0%)	>0.99
**Knowledge (TDF-1): Do you know when and which patients should receive an order for a BC in your hospital (Q2-3)?**				
Definitely (>95-100% of the case)	65 (12.9%)	106 (34.9%)	72 (14.4%)	<0.001
Likely (75-95% of the case)	200 (39.8%)	168 (55.3%)	245 (48.9%)	
Uncertain (25-74% of the case)	148 (29.4%)	28 (9.2%)	128 (25.5%)	
Unlikely (5-24% of the case)	59 (11.7%)	0 (0%)	31 (6.2%)	
Rarely (ranging from never <5% of the case)	19 (3.8%)	0 (0%)	6 (1.2%)	
I do not know	10 (2.0%)	1 (0.3%)	8 (1.6%)	
I do not want to answer	2 (0.4%)	1 (0.3%)	11 (2.2%)	
**Social professional role and identity (TDF-3): Is it an appropriate part of your current job to order BC (Q2-4)?**				
Very appropriate	119 (23.7%)	103 (33.9%)	110 (22.0%)	<0.001
Appropriate	232 (46.1%)	166 (54.6%)	290 (57.9%)	
Uncertain	62 (12.3%)	20 (6.6%)	48 (9.6%)	
Inappropriate	21 (4.2%)	2 (0.7%)	12 (2.4%)	
Very inappropriate	2 (0.4%)	0 (0%)	0 (0%)	
I do not know	10 (2.0%)	0 (0%)	0 (0%)	
I do not want to answer	2 (0.4%)	0 (0%)	19 (3.8%)	
I cannot order BC. It is not part of my job	55 (10.9%)	13 (4.3%)	22 (4.4%)	
**Social professional role and identity (TDF-3): Would it be an appropriate part of your current job to order BC among those who answered that they cannot order for a BC (Q2-5)?**				
Very appropriate	4/55 (7.3%)	0/13 (0%)	0/22 (0%)	0.009
Appropriate	19/55 (34.5%)	8/13 (61.5%)	4/22 (18.2%)	
Uncertain	10/55 (18.2%)	4/13 (30.8%)	2/22 (9.1%)	
Inappropriate	15/55 (27.3%)	1/13 (7.7%)	8/22 (36.4%)	
Very inappropriate	3/55 (5.5%)	0/13 (0%)	2/22 (9.1%)	
I do not know	4/55 (7.3%)	0/13 (0%)	2/22 (9.1%)	
I do not want to answer	0/55 (0%)	0/13 (0%)	4/22 (18.2%)	
**Professional role (Q2-6): Which types of professionals/staff are tasked to draw blood from patients for BC?**				
Medical doctor – executive level	12 (2.4%)	44 (14.5%)	23 (4.6%)	<0.001
Medical doctor – a consultant level	60 (11.9%)	90 (29.6%)	152 (30.3%)	0.11
Medical doctor – a general physician level	72 (14.3%)	105 (34.5%)	129 (25.7%)	<0.001
Medical doctor – a resident level	96 (19.1%)	122 (40.1%)	113 (22.6%)	<0.001
Intern – recent medical school graduate	39 (7.8%)	105 (34.5%)	85 (17.0%)	<0.001
Final-year medical student	27 (5.4%)	99 (32.6%)	25 (5.0%)	<0.001
Registered nurses	342 (68.0%)	215 (70.7%)	392 (78.2%)	0.001

For peer review only - http://bmjopen.bmj.com/site/about/guidelines.xhtml

Questions	Indonesia (n=503)	Thailand (n=304)	Viet Nam (n=501)	P value
Microbiology laboratory team	227 (45.1%)	91 (29.9%)	151 (30.1%)	<0.001
Specialized blood draw team	197 (39.2%)	91 (29.9%)	69 (13.8%)	<0.001
I do not want to answer	3 (0.6%)	0 (0%)	2 (0.4%)	0.41
**Social professional role and identity (TDF-3): Is it an appropriate part of your current job to draw blood (Q2-7)?**				
Very appropriate	34 (6.8%)	36 (11.8%)	49 (9.8%)	0.01
Appropriate	179 (35.6%)	102 (33.6%)	179 (35.7%)	
Uncertain	109 (21.7%)	52 (17.1%)	68 (13.6%)	
Inappropriate	89 (17.7%)	46 (15.1%)	85 (17.0%)	
Very inappropriate	7 (1.4%)	6 (2.0%)	3 (0.6%)	
I do not know	8 (1.6%)	4 (1.3%)	4 (0.8%)	
I do not want to answer	4 (0.8%)	1 (0.3%)	4 (0.8%)	
It is not part of my job to draw blood	73 (14.5%)	57 (18.8%)	109 (21.8%)	
**Skill (TDF-2): How skilled are you in drawing blood excluding those whose jobs did not include drawing blood (Q2-8)?**				
Very good skill	18/430 (4.2%)	12/247 (4.9%)	32/392 (8.2%)	<0.001
Good skill	138/430 (32.1%)	46/247 (18.6%)	112/392 (28.6%)	
Fair skill	202/430 (47.0%)	118/247 (47.8%)	196/392 (50.0%)	
Poor skill	20/430 (4.7%)	52/247 (21.1%)	33/392 (8.4%)	
Very poor skill	4/430 (0.9%)	16/247 (6.5%)	1/392 (0.3%)	
I do not know	39/430 (9.1%)	3/247 (1.2%)	11/392 (2.8%)	
I do not want to answer	9/430 (2.1%)	0/247 (0%)	7/392 (1.8%)	
**Beliefs about capabilities (TDF-4): How confident that you can draw blood successfully excluding those whose jobs did not include drawing blood (Q2-9)?**				
Strongly confident	32/430 (7.4%)	20/247 (8.1%)	42/392 (10.7%)	<0.001
Confident	271/430 (63.0%)	93/247 (37.7%)	231/392 (58.9%)	
Uncertain	74/430 (17.2%)	81/247 (32.8%)	90/392 (23.0%)	
Doubtful	42/430 (9.8%)	34/247 (13.8%)	22/392 (5.6%)	
Strongly doubtful	2/430 (0.5%)	19/247 (7.7%)	6/392 (1.5%)	
I do not know	4/430 (0.9%)	0/247 (0%)	0/392 (0%)	
I do not want to answer	5/430 (1.2%)	0/247 (0%)	1/392 (0.3%)	
**Beliefs about capabilities (TDF-4): How confident that you can draw blood appropriately excluding those whose jobs did not include drawing blood (Q2-10)?**				
Strongly confident	28/430 (6.5%)	30/247 (12.1%)	37/392 (9.4%)	<0.001
Confident	262/430 (60.9%)	109/247 (44.1%)	222/392 (56.6%)	
Uncertain	86/430 (20.0%)	61/247 (24.7%)	109/392 (27.8%)	
Doubtful	44/430 (10.2%)	33/247 (13.4%)	17/392 (4.3%)	
Strongly doubtful	3/430 (0.7%)	11/247 (4.5%)	2/392 (0.5%)	
I do not know	3/430 (0.7%)	1/247 (0.4%)	1/392 (0.3%)	
I do not want to answer	4/430 (0.9%)	2/247 (0.8%)	4/392 (1.0%)	
**Beliefs about capabilities (TDF-4): Are you confident that others can draw blood successfully (Q2-11)?**				
Strongly confident	99 (19.7%)	106 (34.9%)	71 (14.2%)	<0.001
Confident	366 (72.8%)	176 (57.9%)	333 (66.5%)	
Uncertain	17 (3.4%)	14 (4.6%)	88 (17.6%)	

For peer review only - http://bmjopen.bmj.com/site/about/guidelines.xhtml

Questions	Indonesia (n=503)	Thailand (n=304)	Viet Nam (n=501)	P value
Doubtful	16 (3.2%)	7 (2.3%)	6 (1.2%)	
Strongly doubtful	0 (0%)	0 (0%)	1 (0.2%)	
I do not know	2 (0.4%)	1 (0.3%)	1 (0.2%)	
I do not want to answer	3 (0.6%)	0 (0%)	1 (0.2%)	
**Beliefs about capabilities (TDF-4): Are you confident that others can draw blood appropriately (Q2-12)?**				
Strongly confident	86 (17.1%)	66 (21.7%)	45 (9.0%)	<0.001
Confident	342 (68.0%)	184 (60.5%)	273 (54.5%)	
Uncertain	42 (8.3%)	45 (14.8%)	170 (33.9%)	
Doubtful	26 (5.2%)	6 (2.0%)	8 (1.6%)	
Strongly doubtful	1 (0.2%)	2 (0.7%)	2 (0.4%)	
I do not know	4 (0.8%)	1 (0.3%)	1 (0.2%)	
I do not want to answer	2 (0.4%)	0 (0%)	2 (0.4%)	
**Optimism (TDF-5): how optimistic are you that a BC will be sampled and processed in the laboratory appropriately (Q2-13)?**				
Strongly optimistic	70 (13.9%)	38 (12.5%)	31 (6.2%)	<0.001
Optimistic	332 (66.0%)	225 (74.0%)	338 (67.5%)	
Neither optimistic nor pessimistic	74 (14.7%)	31 (10.2%)	124 (24.8%)	
Pessimistic	8 (1.6%)	4 (1.3%)	4 (0.8%)	
Strongly pessimistic	5 (1.0%)	0 (0%)	1 (0.2%)	
I do not know	10 (2.0%)	5 (1.6%)	2 (0.4%)	
I do not want to answer	4 (0.8%)	1 (0.3%)	1 (0.2%)	
**Beliefs about consequence (TDF-6): BC is helpful in clinical decisions (Q3-1-1).**				
Strongly agree	204 (40.6%)	153 (50.3%)	194 (38.7%)	<0.001
Agree	279 (55.5%)	144 (47.4%)	246 (49.1%)	
Uncertain	13 (2.6%)	6 (2.0%)	47 (9.4%)	
Disagree	4 (0.8%)	1 (0.3%)	11 (2.2%)	
Strongly disagree	0 (0%)	0 (0%)	1 (0.2%)	
I do not know	2 (0.4%)	0 (0%)	0 (0%)	
I do not want to answer	1 (0.2%)	0 (0%)	2 (0.4%)	
**Beliefs about consequence (TDF-6): BC is helpful to rule in an infection (Q3-1-2).**				
Strongly agree	192 (38.2%)	123 (40.5%)	162 (32.3%)	<0.001
Agree	276 (54.9%)	159 (52.3%)	260 (51.9%)	
Uncertain	14 (2.8%)	10 (3.3%)	51 (10.2%)	
Disagree	18 (3.6%)	7 (2.3%)	24 (4.8%)	
Strongly disagree	0 (0%)	1 (0.3%)	2 (0.4%)	
I do not know	2 (0.4%)	4 (1.3%)	0 (0%)	
I do not want to answer	1 (0.2%)	0 (0%)	2 (0.4%)	
**Beliefs about consequence (TDF-6): BC is helpful to rule out an infection (Q3-1-3).**				
Strongly agree	137 (27.2%)	72 (23.7%)	59 (11.8%)	<0.001
Agree	258 (51.3%)	97 (31.9%)	163 (32.5%)	
Uncertain	44 (8.7%)	32 (10.5%)	126 (25.1%)	
Disagree	56 (11.1%)	79 (26.0%)	127 (25.3%)	
Strongly disagree	5 (1.0%)	22 (7.2%)	23 (4.6%)	
I do not know	2 (0.4%)	2 (0.7%)	0 (0%)	
I do not want to answer	1 (0.2%)	0 (0%)	3 (0.6%)	

For peer review only - http://bmjopen.bmj.com/site/about/guidelines.xhtml

Questions	Indonesia (n=503)	Thailand (n=304)	Viet Nam (n=501)	P value
**Beliefs about consequence (TDF-6): BC is helpful in detecting AMR infections (Q3-1-4).**				
Strongly agree	267 (53.1%)	147 (48.4%)	154 (30.7%)	<0.001
Agree	219 (43.5%)	140 (46.1%)	272 (54.3%)	
Uncertain	10 (2.0%)	11 (3.6%)	51 (10.2%)	
Disagree	4 (0.8%)	4 (1.3%)	18 (3.6%)	
Strongly disagree	0 (0%)	1 (0.3%)	4 (0.8%)	
I do not know	2 (0.4%)	1 (0.3%)	1 (0.2%)	
I do not want to answer	1 (0.2%)	0 (0%)	1 (0.2%)	
**Beliefs about consequence (TDF-6): BC is helpful in adjusting antibiotics (Q3-1-5).**				
Strongly agree	285 (56.7%)	172 (56.6%)	177 (35.3%)	<0.001
Agree	206 (41.0%)	128 (42.1%)	256 (51.1%)	
Uncertain	9 (1.8%)	2 (0.7%)	40 (8.0%)	
Disagree	0 (0%)	1 (0.3%)	21 (4.2%)	
Strongly disagree	1 (0.2%)	1 (0.3%)	3 (0.6%)	
I do not know	1 (0.2%)	0 (0%)	0 (0%)	
I do not want to answer	1 (0.2%)	0 (0%)	4 (0.8%)	
**Beliefs about consequence (TDF-6): BC can reduce overuse of antibiotics (Q3-1-6).**				
Strongly agree	241 (47.9%)	142 (46.7%)	157 (31.3%)	<0.001
Agree	220 (43.7%)	131 (43.1%)	249 (49.7%)	
Uncertain	30 (6.0%)	19 (6.3%)	59 (11.8%)	
Disagree	9 (1.8%)	11 (3.6%)	30 (6.0%)	
Strongly disagree	1 (0.2%)	1 (0.3%)	4 (0.8%)	
I do not know	1 (0.2%)	0 (0%)	0 (0%)	
I do not want to answer	1 (0.2%)	0 (0%)	2 (0.4%)	
**Beliefs about consequence (TDF-6): BC can reduce length of hospital stay (Q3-1-7).**				
Strongly agree	167 (33.2%)	101 (33.2%)	106 (21.2%)	<0.001
Agree	215 (42.7%)	122 (40.1%)	227 (45.3%)	
Uncertain	97 (19.3%)	54 (17.8%)	124 (24.8%)	
Disagree	18 (3.6%)	23 (7.6%)	39 (7.8%)	
Strongly disagree	0 (0%)	2 (0.7%)	3 (0.6%)	
I do not know	4 (0.8%)	1 (0.3%)	0 (0%)	
I do not want to answer	2 (0.4%)	1 (0.3%)	2 (0.4%)	
**Beliefs about consequence (TDF-6): BC can reduce patient mortality (Q3-1-8).**				
Strongly agree	178 (35.4%)	120 (39.5%)	124 (24.8%)	<0.001
Agree	228 (45.3%)	135 (44.4%)	242 (48.3%)	
Uncertain	79 (15.7%)	38 (12.5%)	98 (19.6%)	
Disagree	12 (2.4%)	8 (2.6%)	31 (6.2%)	
Strongly disagree	1 (0.2%)	0 (0%)	3 (0.6%)	
I do not know	4 (0.8%)	3 (1.0%)	1 (0.2%)	
I do not want to answer	1 (0.2%)	0 (0%)	2 (0.4%)	
**Beliefs about consequence (TDF-6): Accumulative results of BC are helpful in understanding epidemiology of AMR bacterial infections (Q3-1-9).**				
Strongly agree	237 (47.1%)	144 (47.4%)	193 (38.5%)	0.003
Agree	247 (49.1%)	141 (46.4%)	266 (53.1%)	
Uncertain	13 (2.6%)	16 (5.3%)	32 (6.4%)	

For peer review only - http://bmjopen.bmj.com/site/about/guidelines.xhtml

Questions	Indonesia (n=503)	Thailand (n=304)	Viet Nam (n=501)	P value
Disagree	0 (0%)	1 (0.3%)	7 (1.4%)	
Strongly disagree	1 (0.2%)	0 (0%)	1 (0.2%)	
I do not know	4 (0.8%)	2 (0.7%)	0 (0%)	
I do not want to answer	1 (0.2%)	0 (0%)	2 (0.4%)	
**Beliefs about consequence (TDF-6): BC is unnecessary because antibiotic therapy can be determined based on clinical presentations (Q3-3-1).**				
Strongly agree	13 (2.6%)	7 (2.3%)	18 (3.6%)	<0.001
Agree	89 (17.7%)	48 (15.8%)	53 (10.6%)	
Uncertain	154 (30.6%)	48 (15.8%)	113 (22.6%)	
Disagree	199 (39.6%)	146 (48.0%)	264 (52.7%)	
Strongly disagree	42 (8.3%)	54 (17.8%)	53 (10.6%)	
I do not know	6 (1.2%)	1 (0.3%)	0 (0%)	
I do not want to answer	0 (0%)	0 (0%)	0 (0%)	
**Beliefs about consequence (TDF-6): The therapeutic consequence of BC sampling is questionable (Q3-3-2).**				
Strongly agree	12 (2.4%)	25 (8.2%)	16 (3.2%)	<0.001
Agree	82 (16.3%)	58 (19.1%)	45 (9.0%)	
Uncertain	167 (33.2%)	60 (19.7%)	123 (24.6%)	
Disagree	191 (38.0%)	116 (38.2%)	275 (54.9%)	
Strongly disagree	34 (6.8%)	39 (12.8%)	34 (6.8%)	
I do not know	17 (3.4%)	5 (1.6%)	2 (0.4%)	
I do not want to answer	0 (0%)	1 (0.3%)	6 (1.2%)	
**Beliefs about consequence (TDF-6): The scientific basis of the guideline on BC is questionable (Q3-3-3).**				
Strongly agree	9 (1.8%)	16 (5.3%)	15 (3.0%)	<0.001
Agree	45 (8.9%)	63 (20.7%)	43 (8.6%)	
Uncertain	106 (21.1%)	58 (19.1%)	141 (28.1%)	
Disagree	248 (49.3%)	120 (39.5%)	254 (50.7%)	
Strongly disagree	79 (15.7%)	39 (12.8%)	41 (8.2%)	
I do not know	15 (3.0%)	7 (2.3%)	4 (0.8%)	
I do not want to answer	1 (0.2%)	1 (0.3%)	3 (0.6%)	
**Beliefs about consequence (TDF-6): BC is unnecessary because results are often delayed (Q3-3-4).**				
Strongly agree	15 (3.0%)	8 (2.6%)	15 (3.0%)	<0.001
Agree	113 (22.5%)	31 (10.2%)	38 (7.6%)	
Uncertain	119 (23.7%)	23 (7.6%)	82 (16.4%)	
Disagree	212 (42.1%)	161 (53.0%)	303 (60.5%)	
Strongly disagree	36 (7.2%)	80 (26.3%)	62 (12.4%)	
I do not know	8 (1.6%)	0 (0%)	0 (0%)	
I do not want to answer	0 (0%)	1 (0.3%)	1 (0.2%)	
**Beliefs about consequence (TDF-6): BC is unnecessary because results are often not interpretable (Q3-3-5).**				
Strongly agree	7 (1.4%)	4 (1.3%)	11 (2.2%)	<0.001
Agree	46 (9.1%)	18 (5.9%)	26 (5.2%)	
Uncertain	120 (23.9%)	18 (5.9%)	70 (14.0%)	
Disagree	275 (54.7%)	166 (54.6%)	326 (65.1%)	

For peer review only - http://bmjopen.bmj.com/site/about/guidelines.xhtml

Questions	Indonesia (n=503)	Thailand (n=304)	Viet Nam (n=501)	P value
Strongly disagree	47 (9.3%)	97 (31.9%)	67 (13.4%)	
I do not know	7 (1.4%)	1 (0.3%)	0 (0%)	
I do not want to answer	1 (0.2%)	0 (0%)	1 (0.2%)	
**Beliefs about consequence (TDF-6): BC is unnecessary because results are often negative or no growth (Q3-3-6).**				
Strongly agree	9 (1.8%)	6 (2.0%)	11 (2.2%)	<0.001
Agree	57 (11.3%)	26 (8.6%)	39 (7.8%)	
Uncertain	114 (22.7%)	37 (12.2%)	83 (16.6%)	
Disagree	261 (51.9%)	149 (49.0%)	312 (62.3%)	
Strongly disagree	51 (10.1%)	85 (28.0%)	55 (11.0%)	
I do not know	10 (2.0%)	1 (0.3%)	0 (0%)	
I do not want to answer	1 (0.2%)	0 (0%)	1 (0.2%)	
**Beliefs about consequence (TDF-6): BC is unnecessary because cultures are often contaminated (Q3-3-7).**				
Strongly agree	8 (1.6%)	6 (2.0%)	10 (2.0%)	<0.001
Agree	65 (12.9%)	23 (7.6%)	31 (6.2%)	
Uncertain	166 (33.0%)	44 (14.5%)	105 (21.0%)	
Disagree	212 (42.1%)	153 (50.3%)	290 (57.9%)	
Strongly disagree	39 (7.8%)	77 (25.3%)	59 (11.8%)	
I do not know	12 (2.4%)	0 (0%)	1 (0.2%)	
I do not want to answer	1 (0.2%)	1 (0.3%)	5 (1.0%)	
**Beliefs about consequence (TDF-6): BC is unnecessary because results often do not agree with clinical signs (Q3-3-8).**				
Strongly agree	8 (1.6%)	5 (1.6%)	13 (2.6%)	<0.001
Agree	46 (9.1%)	22 (7.2%)	21 (4.2%)	
Uncertain	147 (29.2%)	36 (11.8%)	84 (16.8%)	
Disagree	249 (49.5%)	158 (52.0%)	325 (64.9%)	
Strongly disagree	43 (8.5%)	83 (27.3%)	49 (9.8%)	
I do not know	10 (2.0%)	0 (0%)	0 (0%)	
I do not want to answer	0 (0%)	0 (0%)	9 (1.8%)	
**Beliefs about consequence (TDF-6): BC is unnecessary because a contaminated result often leads to wrong therapeutic approaches (Q3-3-9).**				
Strongly agree	10 (2.0%)	7 (2.3%)	14 (2.8%)	<0.001
Agree	85 (16.9%)	23 (7.6%)	38 (7.6%)	
Uncertain	128 (25.4%)	42 (13.8%)	116 (23.2%)	
Disagree	229 (45.5%)	148 (48.7%)	277 (55.3%)	
Strongly disagree	41 (8.2%)	83 (27.3%)	42 (8.4%)	
I do not know	9 (1.8%)	1 (0.3%)	3 (0.6%)	
I do not want to answer	1 (0.2%)	0 (0%)	11 (2.2%)	
**Environmental context and resources (TDF-11): BC is unnecessary because it is too expensive (Q3-3-10).**				
Strongly agree	25 (5.0%)	6 (2.0%)	12 (2.4%)	<0.001
Agree	83 (16.5%)	19 (6.3%)	24 (4.8%)	
Uncertain	114 (22.7%)	37 (12.2%)	79 (15.8%)	
Disagree	227 (45.1%)	133 (43.8%)	310 (61.9%)	
Strongly disagree	39 (7.8%)	103 (33.9%)	64 (12.8%)	

For peer review only - http://bmjopen.bmj.com/site/about/guidelines.xhtml

Questions	Indonesia (n=503)	Thailand (n=304)	Viet Nam (n=501)	P value
I do not know	12 (2.4%)	5 (1.6%)	2 (0.4%)	
I do not want to answer	3 (0.6%)	1 (0.3%)	10 (2.0%)	
**Beliefs about consequence (TDF-6): BC is not benefiting the patients (Q3-3-11).**				
Strongly agree	5 (1.0%)	5 (1.6%)	10 (2.0%)	<0.001
Agree	19 (3.8%)	17 (5.6%)	20 (4.0%)	
Uncertain	88 (17.5%)	13 (4.3%)	46 (9.2%)	
Disagree	290 (57.7%)	139 (45.7%)	302 (60.3%)	
Strongly disagree	92 (18.3%)	130 (42.8%)	121 (24.2%)	
I do not know	8 (1.6%)	0 (0%)	0 (0%)	
I do not want to answer	1 (0.2%)	0 (0%)	2 (0.4%)	
**Beliefs about consequence (TDF-6): It is not too late to collect BC later, particularly if patients do not improve after receiving empirical antibiotic treatment (Q3-3-12).**				
Strongly agree	23 (4.6%)	48 (15.8%)	15 (3.0%)	<0.001
Agree	116 (23.1%)	114 (37.5%)	107 (21.4%)	
Uncertain	95 (18.9%)	32 (10.5%)	89 (17.8%)	
Disagree	208 (41.4%)	65 (21.4%)	226 (45.1%)	
Strongly disagree	49 (9.7%)	45 (14.8%)	61 (12.2%)	
I do not know	11 (2.2%)	0 (0%)	3 (0.6%)	
I do not want to answer	1 (0.2%)	0 (0%)	0 (0%)	
**Beliefs about consequence (TDF-6): Quality of laboratory is questionable (Q3-3-13).**				
Strongly agree	15 (3.0%)	11 (3.6%)	9 (1.8%)	<0.001
Agree	77 (15.3%)	27 (8.9%)	55 (11.0%)	
Uncertain	147 (29.2%)	81 (26.6%)	148 (29.5%)	
Disagree	196 (39.0%)	114 (37.5%)	239 (47.7%)	
Strongly disagree	48 (9.5%)	62 (20.4%)	40 (8.0%)	
I do not know	18 (3.6%)	8 (2.6%)	5 (1.0%)	
I do not want to answer	2 (0.4%)	1 (0.3%)	5 (1.0%)	
**Beliefs about consequence (TDF-6): Levels of local antibiotic resistance are low (Q3-3-14).**				
Strongly agree	5 (1.0%)	4 (1.3%)	8 (1.6%)	<0.001
Agree	45 (8.9%)	22 (7.2%)	42 (8.4%)	
Uncertain	120 (23.9%)	63 (20.7%)	111 (22.2%)	
Disagree	225 (44.7%)	130 (42.8%)	268 (53.5%)	
Strongly disagree	87 (17.3%)	77 (25.3%)	68 (13.6%)	
I do not know	21 (4.2%)	7 (2.3%)	3 (0.6%)	
I do not want to answer	0 (0%)	1 (0.3%)	1 (0.2%)	
**Goals (TDF-9): How often do you obtain BC prior to administration of empirical antibiotics in patients presenting with sepsis (Q3-5)?**				
All the time (>95-100% of the time)	95 (18.9%)	158 (52.0%)	150 (29.9%)	<0.001
Often (75-95% of the time)	156 (31.0%)	116 (38.2%)	230 (45.9%)	
Moderately (25-74% of the time)	85 (16.9%)	21 (6.9%)	64 (12.8%)	
Occasionally (5-24% of the time)	45 (8.9%)	5 (1.6%)	12 (2.4%)	
Rarely (ranging from never <5% of the time)	82 (16.3%)	0 (0%)	19 (3.8%)	
I do not know	34 (6.8%)	4 (1.3%)	11 (2.2%)	
I do not want to answer	6 (1.2%)	0 (0%)	15 (3.0%)	

For peer review only - http://bmjopen.bmj.com/site/about/guidelines.xhtml

Questions	Indonesia (n=503)	Thailand (n=304)	Viet Nam (n=501)	P value
**Goals (TDF-9): How often do you obtain BC prior to administration of empirical antibiotics in patients presenting with septic shock (Q3-6)?**				
All the time (>95-100% of the time)	90 (17.9%)	234 (77.0%)	218 (43.5%)	<0.001
Often (75-95% of the time)	160 (31.8%)	59 (19.4%)	175 (34.9%)	
Moderately (25-74% of the time)	76 (15.1%)	6 (2.0%)	48 (9.6%)	
Occasionally (5-24% of the time)	48 (9.5%)	0 (0%)	18 (3.6%)	
Rarely (ranging from never <5% of the time)	84 (16.7%)	0 (0%)	20 (4.0%)	
I do not know	40 (8.0%)	3 (1.0%)	9 (1.8%)	
I do not want to answer	5 (1.0%)	2 (0.7%)	13 (2.6%)	
**Memory, attention and decision processes (TDF-10): Would you still order BC if patients are already on antibiotics (Q3-7-1)?**				
Definitely not order	11 (2.2%)	14 (4.6%)	6 (1.2%)	<0.001
Likely not order	19 (3.8%)	53 (17.4%)	28 (5.6%)	
Maybe not order	295 (58.6%)	38 (12.5%)	85 (17.0%)	
Likely to still order	143 (28.4%)	116 (38.2%)	308 (61.5%)	
Very likely to still order	18 (3.6%)	81 (26.6%)	72 (14.4%)	
I do not know	16 (3.2%)	2 (0.7%)	1 (0.2%)	
I do not want to answer	1 (0.2%)	0 (0%)	1 (0.2%)	
**Memory, attention and decision processes (TDF-10): Would you still order BC if patients have anemia (Q3-7-2)?**				
Definitely not order	16 (3.2%)	84 (27.6%)	24 (4.8%)	<0.001
Likely not order	59 (11.7%)	64 (21.1%)	33 (6.6%)	
Maybe not order	255 (50.7%)	52 (17.1%)	58 (11.6%)	
Likely to still order	124 (24.7%)	52 (17.1%)	257 (51.3%)	
Very likely to still order	20 (4.0%)	45 (14.8%)	115 (23.0%)	
I do not know	28 (5.6%)	5 (1.6%)	2 (0.4%)	
I do not want to answer	1 (0.2%)	2 (0.7%)	12 (2.4%)	
**Memory, attention and decision processes (TDF-10): Would you still order BC if blood should be used for other laboratory tests (Q3-7-3)?**				
Definitely not order	7 (1.4%)	57 (18.8%)	59 (11.8%)	<0.001
Likely not order	43 (8.5%)	57 (18.8%)	64 (12.8%)	
Maybe not order	228 (45.3%)	75 (24.7%)	117 (23.4%)	
Likely to still order	158 (31.4%)	63 (20.7%)	172 (34.3%)	
Very likely to still order	20 (4.0%)	40 (13.2%)	60 (12.0%)	
I do not know	41 (8.2%)	12 (3.9%)	21 (4.2%)	
I do not want to answer	6 (1.2%)	0 (0%)	8 (1.6%)	
**Memory, attention and decision processes (TDF-10): Would you still order BC if there are no local guidelines/guidelines for BC sampling (Q3-7-4)?**				
Definitely not order	11 (2.2%)	42 (13.8%)	42 (8.4%)	<0.001
Likely not order	41 (8.2%)	43 (14.1%)	66 (13.2%)	
Maybe not order	241 (47.9%)	95 (31.3%)	136 (27.1%)	
Likely to still order	152 (30.2%)	66 (21.7%)	174 (34.7%)	
Very likely to still order	19 (3.8%)	33 (10.9%)	41 (8.2%)	
I do not know	32 (6.4%)	24 (7.9%)	35 (7.0%)	
I do not want to answer	7 (1.4%)	1 (0.3%)	7 (1.4%)	

For peer review only - http://bmjopen.bmj.com/site/about/guidelines.xhtml

Questions	Indonesia (n=503)	Thailand (n=304)	Viet Nam (n=501)	P value
**Memory, attention and decision processes (TDF-10): Would you still order BC if patients do not meet certain conditions for a BC following the local guidelines (Q3-7-5)?**				
Definitely not order	28 (5.6%)	39 (12.8%)	54 (10.8%)	<0.001
Likely not order	131 (26.0%)	80 (26.3%)	93 (18.6%)	
Maybe not order	250 (49.7%)	93 (30.6%)	177 (35.3%)	
Likely to still order	58 (11.5%)	54 (17.8%)	121 (24.2%)	
Very likely to still order	11 (2.2%)	22 (7.2%)	44 (8.8%)	
I do not know	23 (4.6%)	15 (4.9%)	8 (1.6%)	
I do not want to answer	2 (0.4%)	1 (0.3%)	4 (0.8%)	
**Memory, attention and decision processes (TDF-10): Would you still order BC if patients do not have a health scheme or insurance that covers the cost of BC (Q3-7-6)?**				
Definitely not order	39 (7.8%)	7 (2.3%)	21 (4.2%)	<0.001
Likely not order	56 (11.1%)	33 (10.9%)	43 (8.6%)	
Maybe not order	306 (60.8%)	95 (31.3%)	101 (20.2%)	
Likely to still order	68 (13.5%)	87 (28.6%)	265 (52.9%)	
Very likely to still order	6 (1.2%)	63 (20.7%)	61 (12.2%)	
I do not know	23 (4.6%)	14 (4.6%)	5 (1.0%)	
I do not want to answer	5 (1.0%)	5 (1.6%)	5 (1.0%)	
**Memory, attention and decision processes (TDF-10): Would you still order BC if microbiology laboratory in your hospital is not available (Q3-7-7)?**				
Definitely not order	53 (10.5%)	21 (6.9%)	97 (19.4%)	<0.001
Likely not order	114 (22.7%)	53 (17.4%)	101 (20.2%)	
Maybe not order	229 (45.5%)	77 (25.3%)	120 (24.0%)	
Likely to still order	74 (14.7%)	79 (26.0%)	109 (21.8%)	
Very likely to still order	10 (2.0%)	54 (17.8%)	36 (7.2%)	
I do not know	19 (3.8%)	12 (3.9%)	30 (6.0%)	
I do not want to answer	4 (0.8%)	8 (2.6%)	8 (1.6%)	
**Environmental context and resources (TDF-11): How often could you not order BC because consumables are not available (Q4-1)?**				
All the time (>95-100% of the time)	24 (4.8%)	12 (3.9%)	19 (3.8%)	<0.001
Often (75-95% of the time)	61 (12.1%)	15 (4.9%)	19 (3.8%)	
Moderately (25-74% of the time)	52 (10.3%)	11 (3.6%)	56 (11.2%)	
Occasionally (5-24% of the time)	86 (17.1%)	15 (4.9%)	51 (10.2%)	
Rarely (ranging from never <5% of the time)	219 (43.5%)	232 (76.3%)	309 (61.7%)	
I do not know	53 (10.5%)	18 (5.9%)	25 (5.0%)	
I do not want to answer	8 (1.6%)	1 (0.3%)	22 (4.4%)	
**Environmental context and resources (TDF-11): How often could you not order BC because the microbiology laboratory is not available or not functioning (Q4-2)?**				
All the time (>95-100% of the time)	34 (6.8%)	9 (3.0%)	15 (3.0%)	<0.001
Often (75-95% of the time)	58 (11.5%)	13 (4.3%)	28 (5.6%)	
Moderately (25-74% of the time)	48 (9.5%)	9 (3.0%)	37 (7.4%)	
Occasionally (5-24% of the time)	78 (15.5%)	14 (4.6%)	27 (5.4%)	
Rarely (ranging from never <5% of the time)	224 (44.5%)	238 (78.3%)	342 (68.3%)	

For peer review only - http://bmjopen.bmj.com/site/about/guidelines.xhtml

Questions	Indonesia (n=503)	Thailand (n=304)	Viet Nam (n=501)	P value
I do not know	56 (11.1%)	21 (6.9%)	28 (5.6%)	
I do not want to answer	5 (1.0%)	0 (0%)	24 (4.8%)	
**Environmental context and resources (TDF-11): How often do patients have to pay for BC using their own money (i.e. out of pocket) (Q4-3)?**				
All the time (>95-100% of the time)	26 (5.2%)	11 (3.6%)	6 (1.2%)	<0.001
Often (75-95% of the time)	52 (10.3%)	17 (5.6%)	28 (5.6%)	
Moderately (25-74% of the time)	50 (9.9%)	19 (6.3%)	67 (13.4%)	
Occasionally (5-24% of the time)	69 (13.7%)	48 (15.8%)	134 (26.7%)	
Rarely (ranging from never <5% of the time)	138 (27.4%)	135 (44.4%)	173 (34.5%)	
I do not know	163 (32.4%)	73 (24.0%)	72 (14.4%)	
I do not want to answer	5 (1.0%)	1 (0.3%)	21 (4.2%)	
**Environmental context and resources (TDF-11): Would you say that the benefits of BC outweigh the cost (Q4-4)?**				
Very likely	101 (20.1%)	135 (44.4%)	184 (36.7%)	<0.001
Likely	210 (41.7%)	97 (31.9%)	223 (44.5%)	
Uncertain	93 (18.5%)	37 (12.2%)	34 (6.8%)	
Unlikely	45 (8.9%)	10 (3.3%)	16 (3.2%)	
Very unlikely	3 (0.6%)	13 (4.3%)	17 (3.4%)	
I do not know	49 (9.7%)	12 (3.9%)	17 (3.4%)	
I do not want to answer	2 (0.4%)	0 (0%)	10 (2.0%)	
**Reinforcement (TDF-7): Are there any positive consequences if you order a BC when recommended (Q5-1)?**				
No	283 (56.3%)	187 (61.5%)	206 (41.1%)	<0.001
Yes, social	31 (6.2%)	37 (12.2%)	59 (11.8%)	
Yes, material	4 (0.8%)	2 (0.7%)	8 (1.6%)	
Yes, both social and material	33 (6.6%)	18 (5.9%)	103 (20.6%)	
I do not know	143 (28.4%)	58 (19.1%)	75 (15.0%)	
I do not want to answer	8 (1.6%)	1 (0.3%)	45 (9.0%)	
Other	1 (0.2%)	1 (0.3%)	5 (1.0%)	
**Reinforcement (TDF-7): Are there any negative consequences if you do not order a BC when recommended (Q5-2)?**				
No	248 (49.3%)	101 (33.2%)	134 (26.7%)	<0.001
Yes, social	65 (12.9%)	115 (37.8%)	100 (20.0%)	
Yes, material	8 (1.6%)	4 (1.3%)	13 (2.6%)	
Yes, both social and material	27 (5.4%)	22 (7.2%)	111 (22.2%)	
I do not know	142 (28.2%)	60 (19.7%)	83 (16.6%)	
I do not want to answer	12 (2.4%)	2 (0.7%)	55 (11.0%)	
Other	1 (0.2%)	0 (0%)	5 (1.0%)	
**Reinforcement (TDF-7): Are there any negative consequences if you order a BC when recommended (Q5-3)?**				
No	251 (49.9%)	162 (53.3%)	210 (41.9%)	<0.001
Yes, social	47 (9.3%)	43 (14.1%)	31 (6.2%)	
Yes, material	10 (2.0%)	3 (1.0%)	31 (6.2%)	
Yes, both social and material	30 (6.0%)	14 (4.6%)	91 (18.2%)	
I do not know	150 (29.8%)	78 (25.7%)	83 (16.6%)	
I do not want to answer	14 (2.8%)	4 (1.3%)	53 (10.6%)	

For peer review only

Questions	Indonesia (n=503)	Thailand (n=304)	Viet Nam (n=501)	P value
Other	1 (0.2%)	0 (0%)	2 (0.4%)	
**Behaviour regulation (TDF-14): Any training, lectures, classes or meetings that provide you knowledge about local/national/international guidelines for BC sampling (Q5-5)?**				
No	153 (30.4%)	64 (21.1%)	52 (10.4%)	<0.001
Yes, infrequent (less than once a year)	90 (17.9%)	87 (28.6%)	111 (22.2%)	
Yes, occasionally (at least once a year)	109 (21.7%)	84 (27.6%)	196 (39.1%)	
Yes, regularly	53 (10.5%)	22 (7.2%)	61 (12.2%)	
I do not know	91 (18.1%)	46 (15.1%)	74 (14.8%)	
I do not want to answer	5 (1.0%)	1 (0.3%)	6 (1.2%)	
Other	2 (0.4%)	0 (0%)	1 (0.2%)	
**Behaviour regulation (TDF-14): any procedures that support you or doctors to order or regulate ordering of BC per local/national/international guidelines (Q5-6)?**				
No	129 (25.7%)	71 (23.4%)	76 (15.2%)	<0.001
Poster	57 (11.3%)	40 (13.2%)	66 (13.2%)	0.62
Standard order form	120 (23.9%)	90 (29.6%)	107 (21.4%)	0.03
Computer system to remind ordering BC	25 (5.0%)	14 (4.6%)	74 (14.8%)	<0.001
case review (e.g. grand round; morning ward round, clinical meetings, and BC is often mentioned)	76 (15.1%)	86 (28.3%)	164 (32.7%)	<0.001
Stewardship programme and reviewing BC is included in the programme	61 (12.1%)	25 (8.2%)	121 (24.2%)	<0.001
Local hospital guideline (e.g. standard operating procedure [SOP])	113 (22.5%)	77 (25.3%)	162 (32.3%)	0.002
I do not know	107 (21.3%)	49 (16.1%)	66 (13.2%)	0.003
I do not want to answer	9 (1.8%)	2 (0.7%)	15 (3.0%)	0.07
**Social influence (TDF-12): To what extent do you order BC because you are following local norms (Q6-1)?**				
All the time (>95-100% of the time)	50 (9.9%)	67 (22.0%)	64 (12.8%)	<0.001
Often (75-95% of the time)	130 (25.8%)	166 (54.6%)	174 (34.7%)	
Moderately (25-74% of the time)	84 (16.7%)	41 (13.5%)	144 (28.7%)	
Occasionally (5-24% of the time)	67 (13.3%)	15 (4.9%)	40 (8.0%)	
Rarely (ranging from never <5% of the time)	80 (15.9%)	8 (2.6%)	40 (8.0%)	
I do not know	87 (17.3%)	7 (2.3%)	25 (5.0%)	
I do not want to answer	5 (1.0%)	0 (0%)	14 (2.8%)	
**Social influence (TDF-12): Influence from nurses (Q6-2-1)? Positive influence could mean facilitate, support or encourage BC sampling. Negative influence could mean hinder or discourage BC sampling.**				
Very positive influence	46 (9.1%)	29 (9.5%)	60 (12.0%)	<0.001
Positive influence	230 (45.7%)	103 (33.9%)	154 (30.7%)	
Neither positive nor negative influence	162 (32.2%)	122 (40.1%)	228 (45.5%)	
Negative influence	15 (3.0%)	26 (8.6%)	25 (5.0%)	
Very negative influence	1 (0.2%)	1 (0.3%)	0 (0%)	
I do not know	45 (8.9%)	19 (6.3%)	30 (6.0%)	
I do not want to answer	4 (0.8%)	4 (1.3%)	4 (0.8%)	

For peer review only

Questions	Indonesia (n=503)	Thailand (n=304)	Viet Nam (n=501)	P value
**Social influence (TDF-12): Influence from final-year medical students (Q6-2-2)?**				
Very positive influence	29 (5.8%)	22 (7.2%)	30 (6.0%)	0.004
Positive influence	155 (30.8%)	87 (28.6%)	104 (20.8%)	
Neither positive nor negative influence	249 (49.5%)	157 (51.6%)	315 (62.9%)	
Negative influence	4 (0.8%)	3 (1.0%)	6 (1.2%)	
Very negative influence	1 (0.2%)	1 (0.3%)	0 (0%)	
I do not know	60 (11.9%)	27 (8.9%)	42 (8.4%)	
I do not want to answer	5 (1.0%)	7 (2.3%)	4 (0.8%)	
**Social influence (TDF-12): Influence from Interns (Q6-2-3)?**				
Very positive influence	31 (6.2%)	41 (13.5%)	33 (6.6%)	<0.001
Positive influence	182 (36.2%)	134 (44.1%)	170 (33.9%)	
Neither positive nor negative influence	205 (40.8%)	96 (31.6%)	251 (50.1%)	
Negative influence	5 (1.0%)	4 (1.3%)	3 (0.6%)	
Very negative influence	1 (0.2%)	0 (0%)	1 (0.2%)	
I do not know	70 (13.9%)	24 (7.9%)	38 (7.6%)	
I do not want to answer	9 (1.8%)	5 (1.6%)	5 (1.0%)	
**Social influence (TDF-12): Influence from residents (Q6-2-4)?**				
Very positive influence	64 (12.7%)	73 (24.0%)	79 (15.8%)	<0.001
Positive influence	270 (53.7%)	138 (45.4%)	219 (43.7%)	
Neither positive nor negative influence	109 (21.7%)	63 (20.7%)	161 (32.1%)	
Negative influence	2 (0.4%)	3 (1.0%)	1 (0.2%)	
Very negative influence	0 (0%)	0 (0%)	1 (0.2%)	
I do not know	51 (10.1%)	23 (7.6%)	37 (7.4%)	
I do not want to answer	7 (1.4%)	4 (1.3%)	3 (0.6%)	
**Social influence (TDF-12): Influence from doctors (Q6-2-5)?**				
Very positive influence	82 (16.3%)	62 (20.4%)	67 (13.4%)	<0.001
Positive influence	293 (58.3%)	125 (41.1%)	216 (43.1%)	
Neither positive nor negative influence	90 (17.9%)	85 (28.0%)	188 (37.5%)	
Negative influence	6 (1.2%)	3 (1.0%)	3 (0.6%)	
Very negative influence	0 (0%)	3 (1.0%)	1 (0.2%)	
I do not know	29 (5.8%)	23 (7.6%)	15 (3.0%)	
I do not want to answer	3 (0.6%)	3 (1.0%)	11 (2.2%)	
**Social influence (TDF-12): Influence from consultants (Q6-2-6)?**				
Very positive influence	172 (34.2%)	117 (38.5%)	109 (21.8%)	<0.001
Positive influence	255 (50.7%)	125 (41.1%)	261 (52.1%)	
Neither positive nor negative influence	38 (7.6%)	41 (13.5%)	113 (22.6%)	
Negative influence	5 (1.0%)	4 (1.3%)	4 (0.8%)	
Very negative influence	1 (0.2%)	2 (0.7%)	0 (0%)	
I do not know	26 (5.2%)	11 (3.6%)	13 (2.6%)	
I do not want to answer	6 (1.2%)	4 (1.3%)	1 (0.2%)	
**Social influence (TDF-12): Influence from head of department (Q6-2-7)?**				
Very positive influence	81 (16.1%)	51 (16.8%)	135 (26.9%)	<0.001
Positive influence	254 (50.5%)	89 (29.3%)	252 (50.3%)	
Neither positive nor negative influence	104 (20.7%)	119 (39.1%)	95 (19.0%)	
Negative influence	10 (2.0%)	6 (2.0%)	6 (1.2%)	
Very negative influence	0 (0%)	1 (0.3%)	0 (0%)	

For peer review only - http://bmjopen.bmj.com/site/about/guidelines.xhtml

Questions	Indonesia (n=503)	Thailand (n=304)	Viet Nam (n=501)	P value
I do not know	48 (9.5%)	34 (11.2%)	11 (2.2%)	
I do not want to answer	6 (1.2%)	4 (1.3%)	2 (0.4%)	
**Social influence (TDF-12): Influence from executive or administrative level of the hospital (Q6-2-8)?**				
Very positive influence	55 (10.9%)	35 (11.5%)	101 (20.2%)	<0.001
Positive influence	188 (37.4%)	67 (22.0%)	216 (43.1%)	
Neither positive nor negative influence	169 (33.6%)	145 (47.7%)	154 (30.7%)	
Negative influence	21 (4.2%)	8 (2.6%)	7 (1.4%)	
Very negative influence	8 (1.6%)	2 (0.7%)	1 (0.2%)	
I do not know	57 (11.3%)	42 (13.8%)	19 (3.8%)	
I do not want to answer	5 (1.0%)	5 (1.6%)	3 (0.6%)	
**Social influence (TDF-12): Influence from patients (Q6-2-9)?**				
Very positive influence	43 (8.5%)	44 (14.5%)	57 (11.4%)	<0.001
Positive influence	197 (39.2%)	74 (24.3%)	148 (29.5%)	
Neither positive nor negative influence	197 (39.2%)	141 (46.4%)	250 (49.9%)	
Negative influence	18 (3.6%)	14 (4.6%)	21 (4.2%)	
Very negative influence	1 (0.2%)	1 (0.3%)	1 (0.2%)	
I do not know	44 (8.7%)	26 (8.6%)	20 (4.0%)	
I do not want to answer	3 (0.6%)	4 (1.3%)	4 (0.8%)	
**Social influence (TDF-12): Influence from family of patients (Q6-2-10)?**				
Very positive influence	32 (6.4%)	21 (6.9%)	34 (6.8%)	<0.001
Positive influence	171 (34.0%)	40 (13.2%)	119 (23.8%)	
Neither positive nor negative influence	221 (43.9%)	186 (61.2%)	282 (56.3%)	
Negative influence	23 (4.6%)	20 (6.6%)	39 (7.8%)	
Very negative influence	3 (0.6%)	2 (0.7%)	2 (0.4%)	
I do not know	50 (9.9%)	30 (9.9%)	19 (3.8%)	
I do not want to answer	3 (0.6%)	5 (1.6%)	6 (1.2%)	
**Emotions (TDF-13): Any emotional factors (Q6-4)?**				
Yes	51 (10.1%)	10 (3.3%)	32 (6.4%)	0.001
**Gender (Q7-2)**				
Female	263 (52.3%)	195 (64.1%)	222 (44.3%)	<0.001
Male	236 (46.9%)	106 (34.9%)	263 (52.5%)	
Other	1 (0.2%)	0 (0%)	0 (0%)	
I do not want to answer	3 (0.6%)	3 (1.0%)	16 (3.2%)	
**Hospital bed size (Q7-3)**				
<200	99 (19.7%)	35 (11.5%)	24 (4.8%)	<0.001
201-400	107 (21.3%)	46 (15.1%)	29 (5.8%)	
401-600	72 (14.3%)	39 (12.8%)	62 (12.4%)	
601-1,000	66 (13.1%)	45 (14.8%)	144 (28.7%)	
1,001-2,000	39 (7.8%)	82 (27.0%)	125 (25.0%)	
> 2,000	27 (5.4%)	30 (9.9%)	74 (14.8%)	
I do not know	89 (17.7%)	27 (8.9%)	35 (7.0%)	
I do not want to answer	4 (0.8%)	0 (0%)	8 (1.6%)	
**Department (Q7-4)**				
Internal medicine	149 (29.6%)	155 (51.0%)	146 (29.1%)	<0.001
Pediatrics	65 (12.9%)	43 (14.1%)	45 (9.0%)	0.05
Infection disease division/department	12 (2.4%)	5 (1.6%)	56 (11.2%)	<0.001
Surgery	21 (4.2%)	45 (14.8%)	81 (16.2%)	<0.001

For peer review only - http://bmjopen.bmj.com/site/about/guidelines.xhtml

Questions	Indonesia (n=503)	Thailand (n=304)	Viet Nam (n=501)	P value
Orthopaedics	6 (1.2%)	18 (5.9%)	14 (2.8%)	0.001
Obstetrics / Gynaecology	20 (4.0%)	29 (9.5%)	7 (1.4%)	<0.001
Emergency department	112 (22.3%)	34 (11.2%)	29 (5.8%)	<0.001
Intensive care unit	45 (8.9%)	13 (4.3%)	51 (10.2%)	0.01
I do not want to answer	24 (4.8%)	25 (8.2%)	52 (10.4%)	0.004
Other	137 (27.2%)	29 (9.5%)	58 (11.6%)	<0.001

Gray color represents questions that were asked to subsets of participants. [1] Included primary health care, clinic, retired and answers as role of doctors (including residents, interns and medical students).

For peer review only

Appendix S6: Sample quotes

TDF Domains	Themes	Sample quotes
Goal	Priority of BC	• "If other urgent examinations are to be required, BC could be delayed." (Vietnamese respondent [barrier]).<br>• "Early blood cultures should be encouraged for patients presenting with infection before antibiotics are given" (Vietnamese respondent [enabler])<br>• "BC should be performed, although the results are often negative. We can't wait for patients not responding to empirical antibiotics before starting BC. It could lead to a prolonged hospital stay" (Indonesian respondent [enabler])
Social professional role and identity	Level of doctors who can order or initiate an order for BC	• "Medical students can order BC; however, medical students must have a signature of a supervising medical doctor together all the time." (Thai respondent [enabler])<br>• "Medical doctors in charge hold the decisions of ordering BC. However, residents (medical doctors who are currently under postgraduate clinical doctors) could report (to medical doctors in charge) which patients need BC." (Indonesian respondent [barrier])
	Perception about their role to order or initiate an order for BC.	
	Perception about their role to draw blood for BC	
Belief about consequences	Perception that BC is helpful	• "(BC is helpful because) immediate use of BC and prior to giving antibiotics can inform whether a patient has bacteraemia or not, what organism is the cause, and which antibiotic would be appropriate." (Thai respondents [enabler])<br>• "(BC is helpful because) BC shortens the time to find the agent and shortens the treatment time for the patient" (Vietnamese respondent)<br>• "(BC is helpful because) BC can reduce irrational antibiotic prescriptions." (Indonesian respondent)
	Perception that BC is unnecessary	• "(BC is unnecessary because) BC often requires a long time to generate the results. Hence, the patient's condition has improved with empirical antibiotics when BC results are generated." (Indonesian respondent [barrier])<br>• "(BC is unnecessary because) laboratory often causes contamination, making the result irrelevant to clinical signs." (Thai respondent [barrier])<br>• "(BC is unnecessary because) most patients have self-medication with antibiotics at home, so BC often yields undesirable results." (Vietnamese respondent [barrier])<br>• "(BC is unnecessary because) time to return results is slow and most of them do not find pathogenic bacteria." (Vietnamese respondent [barrier])

For peer review only - http://bmjopen.bmj.com/site/about/guidelines.xhtml

TDF Domains	Themes	Sample quotes
		• "BCs are not useful when the focal point of the infection is clear and the patient responds well to treatment." (Vietnamese respondent [barrier])
Intention	Intention to follow guidelines	• A guideline on BC examination should be written in detail, reviewed multiple times, monitored and followed with the appropriate rewards and punishment. (Vietnamese respondent [enabler]
Knowledge	Awareness of guidelines	
	Training	• "I have not learnt about the local recommendation for BC sampling in my university hospital." (Indonesian respondent [barrier]).<br>• "BC has not been highlighted in the clinics when I have Bed Side Teaching, Case Review, Tutorials, etc. It is recommended to do as ideal as is written in the literature." (Indonesian respondent [barrier])
Social influence	Norms of BC sampling	• "Social factors could influence diagnosis and therapy." (Indonesian respondent [barrier/enabler])
	Influences from healthcare workers, patients and family of patients	• "The patient's families often have a strong influence on patients. They often decide not to provide consent to BC." (Indonesian respondent [barrier])<br>• "Negative influence in the order of BC is cost. Supervisor or the executives (of the hospitals) gave an order to control the cost." (Thai respondent [barrier])<br>• "The patient's relatives are not satisfied with the cost of (BC) testing." (Vietnamese respondent [barrier]).<br>• "Because people do not understand, when ordering BC, they often complain." (Vietnamese respondent [barrier])<br>• "Some patients think that physicians and other healthcare workers only perform BC examinations for money." (Indonesian respondent [barrier]).<br>• "Sometimes, when the blood puncture fails on the first try, patients and their families refuse to collect more blood samples." (Indonesian respondents [barrier]).
Reinforcement	Consequences that discourage BC sampling	• "Warnings are given due to the costly examination, especially for patients insured with the Healthcare and Social Security Agency." (Indonesian respondent [barrier])<br>• "Sometimes, the cost of BC cannot be reimbursed, and the doctor has to pay." (Vietnamese respondent [barrier])<br>• "Occasionally, the insurance assessment agency often asks questions, requires explanations and can make it difficult to limit the order of BC for patients." (Vietnamese respondent [barrier])
	Consequences that encourage BC sampling	• "The consequences are usually minimal. The hospital prioritizes the clinical improvement and satisfaction of the patients and their families instead of conducting according to the guidelines or minimizing antibiotic resistance." (Vietnamese respondent [barrier])<br>• "If the patient dies without BC testing, it will be questioned in the death case report." (Indonesian respondent [enabler])<br>• "If (we) do not follow the recommendation for (BC) diagnostic tests, there will be a verbal reprimand in order

For peer review only - http://bmjopen.bmj.com/site/about/guidelines.xhtml

TDF Domains	Themes	Sample quotes
		to make sure that the care is up to the standard." (Thai respondent [enabler])<br>• "There are no incentives, rewards or penalties." (Vietnamese respondent [lack of enabler])<br>• "The case of septic shock without a BC will be reprimanded." (Vietnamese respondent [enabler])
Behavioural regulation	Regulations on cost reimbursement	• "National insurance coverage and hospital regulation could inhibit BC examination." (Indonesian respondent [barrier])<br>• "The insurance often disapproves of BC examination. It is only approved when patients are admitted to the ICU or HCU [High Care Unit]." (Indonesian respondent [barrier])<br>• "It is affected by the insurance. Healthcare and Social Security Agency in Indonesia only covers septic patients around two million rupiahs/patient [about 138 US$], it is not sufficient to cover the resources required, including BC examinations." (Indonesian respondent [barrier].<br>• "Some hospitals allow only three laboratory tests; therefore, (doctors) must select laboratory tests for patients." (Thai respondent [barrier])<br>• "When the final diagnosis does not match, (the cost of BC) will not be paid by Health Insurance." (Vietnamese respondent [barrier])<br>• "Medical professionals often object to BC due to tiredness [disheartened feeling] and the consequence of reduced reimbursement." (Vietnamese respondent [barrier])<br>• "It is difficult (to order BC) because there are restrictions from the financial coverage on the Healthcare and Social Security Agency." (Indonesian respondent)
	Procedures to support or regulate doctors to order BC	•
Environmental context and resources	Perceived cost-effectiveness of BC	• "BC is still not cost-effective for my hospital" (Indonesian respondent [barrier]).<br>• "BC is not cost-effective" (Vietnamese respondent [barrier])
	Availability of microbiology laboratories, transport modalities, resources and consumables	• "Hospitals that do not have a microbiology laboratory cannot obtain culture results. If you still want to take BC, you have to send it to another hospital, it will cost the patient more" (Vietnamese respondent [barrier])
	Out-of-pocket	• "BC is essential, but it costs a lot (Indonesia Rp 750.000,00 [about 52US$]), and many patients could not afford it." (Indonesian respondent [barrier])<br>• "Patients usually refuse BC due to the cost." (Indonesian respondent [barrier])
Emotion	Fear or anxiety of healthcare providers	• "In some patients with blood-borne infectious diseases, doctors are afraid to draw blood." (Vietnamese respondent [barrier])<br>• "Nurses are afraid to draw a lot of blood." (Vietnamese respondent [barrier])

For peer review only

TDF Domains	Themes	Sample quotes
	Fear or anxiety of patients or families of patients	• "Patient and their families are afraid of contracting blood-transmitted diseases." (Indonesian respondent [barrier])<br>• "Patient are afraid to be drawn a lot of blood." (Vietnamese respondent [barrier])<br>• "Fear of pain. Fear of needle" (Thai respondent [barrier]<br>• "Anxiety, panic or uncooperative attitude." (Vietnamese respondent [barrier])<br>• "Patients are afraid that taking a lot of blood will cause anemia." (Vietnamese respondent [barrier])
Optimism	Confidence that BC will be appropriately sampled and processed in the laboratory	
Skill	Skill in drawing blood for BC	
Memory, attention and decision processes	Patients who are already on antibiotics or have anemia	"In patients who have already received antibiotics, BC is not meaningful." (Vietnamese respondent [barrier]
	Clinical presentations for deciding to order BC	"Patients who are receiving palliative-care may not be tested for BC, even though there are criteria for it" (Thai respondent [barrier])
Beliefs about capabilities	Belief in their own capability to draw blood	
	Belief in capability of those who are tasked to draw blood	

For peer review only - http://bmjopen.bmj.com/site/about/guidelines.xhtml

Appendix S7. Associations between barriers/enablers and the responses that they would definitely take BC in the case scenario

Barriers or enablers	Indonesia[1] (n=503)	Thailand[1] (n=304)	Viet Nam[1] (n=501)	Odds ratio[2]	P value
**TDF Domain: Knowledge**					
**Awareness of local guidelines**					
Yes	42.7% (102/239)	91.1% (154/169)	59.5% (206/346)	2.55 (1.93-3.38)	<0.001
No[1]	21.1% (53/251)	89.3% (117/131)	29.4% (42/143)	1.0	
**Awareness of international guidelines**					
Yes	38.9% (138/226)	90.8% (128/141)	65.9% (147/223)	1.97 (1.50-2.57)	<0.001
No	25.4% (67/264)	89.9% (143/159)	38.0% (101/266)	1.0	
**Any training, lectures, classes or meetings that provide knowledge about guidelines for BC sampling**					
Available	36.2% (92/254)	92.2% (178/193)	53.5% (197/368)	1.68 (1.18-2.38)	0.004
Not available	21.7% (33/152)	82.8% (53/64)	46.2% (24/52)	1.0	
**TDF Domain: Goals**					
How often do you obtain BC prior to receiving empirical antibiotic in patients presenting with sepsis?					
All the time / Often (>75-100% of the time)	45.4% (113/249)	91.6% (251/274)	58.6% (222/379)	4.25 (3.04-5.94)	<0.001
Moderately / Occasionally / Rarely (0-74% of the time)	15.6% (33/212)	73.1% (19/26)	22.1% (21/95)	1.0	
How often do you obtain BC prior to receiving empirical antibiotic in patients presenting with septic shock?					
All the time / Often (>75-100% of the time)	44.8% (111/248)	90.1% (264/293)	56.4% (221/392)	3.71 (2.61-5.27)	<0.001
Moderately / Occasionally / Rarely (0-74% of the time)	15.4% (32/208)	83.3% (5/6)	25.6% (22/86)	1.0	
**TDF Domain: Intention**					
**Intention to follow local guidelines[3]**					
All the time / Often (>75-100% of the cases)	51.7% (89/172)	90.5% (142/157)	64.7% (183/283)	2.92 (1.88-4.53)	<0.001
Moderately / Occasionally / Rarely (0-74% of the cases)	18.6% (11/59)	100% (12/12)	37.7% (23/61)	1.0	
**TDF Domain: Social professional role and identity**					
**Current job**					
Medical doctor – an executive level	15.4% (2/13)	60.0% (2/3)	35.3% (6/17)	0.20 (0.09-0.47)	<0.001
Medical doctor – a consultant level	34.4% (25/73)	90.7% (68/75)	49.2% (97/197)	0.48 (0.33-0.69)	
Medical doctor – a general physician level	10.5% (13/124)	81.6% (31/38)	46.0% (51/111)	0.27 (0.18-0.40)	
Medical doctor – a resident/registra/fellow level	48.8% (82/168)	93.7% (59/63)	68.3% (69/101)	1.0	
Intern – recent medical school graduate	12.1% (4/33)	88.6% (31/35)	35.7% (5/14)	0.26 (0.14-0.49)	
Final-year medical student	34.4% (31/90)	92.1% (81/88)	40.7% (24/59)	0.50 (0.33-0.76)	
**Perception about their role to order or initiate an order for BC**					

For peer review only - http://bmjopen.bmj.com/site/about/guidelines.xhtml

Barriers or enablers	Indonesia[1] (n=503)	Thailand[1] (n=304)	Viet Nam[1] (n=501)	Odds ratio[2]	P value
Very appropriate / Appropriate	45.5% (120/264)	91.2% (250/274)	61.2% (195/317)	3.36 (2.50-4.51)	<0.001
Uncertain / Inappropriate / Very inappropriate	16% (36/225)	78.6% (22/28)	33.3% (55/165)	1.0	
**Perception about their role to draw blood for BC[3]**					
Very appropriate / Appropriate	38.0% (27/71)	87.8% (65/74)	52.4% (54/103)	1.94 (1.04-3.64)	0.04
Uncertain / Inappropriate / Very inappropriate	28.6% (4/14)	94.8% (55/58)	25.6% (10/39)	1.0	
**TDF Domain: Social influences**					
To what extent do you order BC in your hospital because you are following local norms? [5]					
All the time / Often (>75-100% of the time)	45.3% (81/179)	90.1% (210/233)	61.3% (146/238)	2.20 (1.67-2.90)	<0.001
Moderately / Occasionally / Rarely (0-74% of the time)	22.2% (51/230)	90.6% (58/64)	41.3% (92/223)	1.0	
**TDF Domain: Environmental context and resources**					
**Do the benefits of BC outweigh the cost?**					
Very likely / likely	35.3% (109/309)	91.0% (211/232)	53.1% (216/407)	1.63 (1.17-2.26)	0.004
Uncertain / Unlikely / Very unlikely	22.0% (31/141)	86.7% (52/60)	42.3% (29/67)	1.0	
**How often are consumables for BC not available?**					
All the time / Often (>75-100% of the time)	31.3% (26/83)	88.9% (24/27)	34.2% (13/38)	0.81 (0.53-1.22)	0.32
Moderately / Occasionally / Rarely (0-74% of the time)	31.9% (114/357)	89.5% (231/258)	53.5% (222/415)	1.0	
**How often are laboratories not available or not functioning?**					
All the time / Often (>75-100% of the time)	28.9% (26/90)	90.9% (2/22)	48.8% (21/43)	0.94 (0.63-1.41)	0.78
Moderately / Occasionally / Rarely (0-74% of the time)	32.6% (114/350)	89.3% (233/261)	53.3% (216/405)	1.0	
**How often do patients have to pay for BC using their own money?**					
All the time / Often (>75-100% of the time)	22.4% (17/76)	92.7% (26/28)	47.1% (16/34)	0.79 (0.51-1.22)	0.29
Moderately / Occasionally / Rarely (0-74% of the time)	36.2% (93/257)	88.1% (178/202)	55.8% (208/373)	1.0	
**Considering whether "patients can afford the cost of BC" as another reason for deciding to do BC sampling**					
Yes	31.1% (33/106)	92.6% (25/27)	46.9% (30/64)	1.12 (0.79-1.61)	0.53
No	31.4% (124/395)	89.5% (248/277)	51.0% (222/435)	1.0	
**TDF Domain: Behavioural regulation**					
**Considering whether "patients have a health scheme or insurance that covers the cost of BC" as another reason for deciding to do BC sampling [6]**					
Yes	27.7% (31/112)	92.6% (25/27)	38.6% (22/57)	0.82 (0.57-1.18)	0.29
No	32.4% (126/389)	89.5% (248/277)	52.0% (230/442)	1.0	
**Considering whether "Patients are likely to have a final diagnosis that includes the cost of BC in the package of fee for service" as another reason for deciding to do BC sampling [6]**					

For peer review only - http://bmjopen.bmj.com/site/about/guidelines.xhtml

Barriers or enablers	Indonesia[1] (n=503)	Thailand[1] (n=304)	Viet Nam[1] (n=501)	Odds ratio[2]	P value
Yes	33.8% (24/69)	96.4% (27/28)	41.8% (23/55)	1.04 (0.70-1.54)	0.85
No	30.8% (133/432)	89.1% (246/276)	51.6% (229/444)	1.0	
**Procedures that support doctors to order or regulate ordering of BC**					
No	44.7% (34/76)	88.7% (63/71)	24.2% (31/128)	1.0	0.006
Poster (and BC is mentioned)	36.8% 921/57)	92.5% (37/40)	51.5% (34/66)	1.13 (0.76-1.69)	
Standard order form for patients with sepsis (with BC written)	32.5% (39/120)	92.2% (83/90)	46.7% (50/107)	0.82 (0.59-1.14)	
Computer system to remind ordering BC	36.0% (9/25)	92.9% (13/14)	45% (33/73)	0.72 (0.48-1.15)	
case reviews (e.g. grand round; with BC often mentioned)	44.7% (34/76)	90.7% (78/86)	57.3% (94/164)	1.38 (0.94-2.00)	
Stewardship programmes (including BC)	49.2% (30/61)	92.0% (23/25)	58.7% (71/121)	1.33 (0.87-2.03)	
Local hospital guideline (e.g. standard operating procedure)	37.2% (42/113)	94.8% (73/77)	58.6% (95/162)	1.45 (1.06-1.99)	
**TDF Domain: Reinforcement**					
Positive consequences if doctors order a BC when it is recommended					
Yes	29.9% (20/67)	86.0% (49/57)	42.4% (72/170)	0.53 (0.37-0.74)	<0.001
No	32.0% (136/425)	90.6% (222/245)	57.4% (160/279)	1.0	
Negative consequences if doctors do not order a BC when it is recommended					
Yes	39.4% (39/99)	90.1% (127/141)	50.0% (112/224)	0.87 (0.63-1.21)	0.42
No	30.1% (117/389)	89.4% (144/161)	55.6% (120/216)	1.0	
Negative consequences if doctors order a BC when it is recommended					
Yes	29.2% (19/65)	86.0% (49/57)	41.4% (67/162)	0.48 (0.34-0.67)	<0.001
No	32.3% (136/421)	90.5% (220/243)	60.1% (170/283)	1.0	
**TDF Domain: Belief about consequences**					
BC is helpful in clinical decision					
Strongly agree / Agree	31.5% (152/482)	89.9% (267/297)	54.1% (237/438)	2.96 (1.71-5.12)	<0.001
Uncertain / Disagree / Strongly disagree	23.5% (4/17)	85.7% (6/7)	23.7% (14/59)	1.0	
BC is helpful to rule in an infection					
Strongly agree / Agree	31.9% (149/467)	90.1% (254/282)	52.4% (220/420)	1.58 (1.04-2.39)	0.03
Uncertain / Disagree / Strongly disagree	21.9% (7/32)	100% (18/18)	40.3% (31/77)	1.0	
BC is helpful to rule out an infection					
Strongly agree / Agree	31.2% (123/394)	88.2% (149/169)	47.7% (105/220)	0.91 (0.69-1.19)	0.49
Uncertain / Disagree / Strongly disagree	31.4% (33/105)	91.7% (122/133)	52.9% (146/276)	1.0	
BC is helpful to detecting AMR bacterial infections					
Strongly agree / Agree	31.3% (152/485)	89.2% (256/287)	51.2% (217/424)	1.26 (0.80-1.98)	0.32
Uncertain / Disagree / Strongly disagree	28.6% (4/14)	100% (16/16)	45.2% (33/73)	1.0	

For peer review only - http://bmjopen.bmj.com/site/about/guidelines.xhtml

Barriers or enablers	Indonesia[1] (n=503)	Thailand[1] (n=304)	Viet Nam[1] (n=501)	Odds ratio[2]	P value
BC is helpful in adjusting antibiotics					
Strongly agree / Agree	31.0% (152/490)	89.7% (269/300)	52.2% (225/431)	1.50 (0.90-2.50)	0.12
Uncertain / Disagree / Strongly disagree	44.4% (4/9)	100% (4/4)	39.1% (25/64)	1.0	
BC can reduce overuse of antibiotics					
Strongly agree / Agree	30.7% (141/460)	89.0% (243/273)	52.2% (211/404)	1.08 (0.74-1.58)	0.68
Uncertain / Disagree / Strongly disagree	38.5% (15/39)	97% (30/31)	42.0% (40/93)	1.0	
BC can reduce length of hospital stay					
Strongly agree / Agree	31.5% (120/381)	91.5% (204/223)	55.3% (183/331)	1.53 (1.14-2.04)	0.004
Uncertain / Disagree / Strongly disagree	29.6% (34/115)	86.1% (68/79)	41.0% (68/166)	1.0	
BC can reduce patient mortality					
Strongly agree / Agree	32.8% (133/405)	89.0% (227/255)	55.0% (200/364)	1.61 (1.18-2.20)	0.003
Uncertain / Disagree / Strongly disagree	23.9% (22/92)	95.7% (44/46)	38.6% (51/132)	1.0	
Accumulative results of BC are helpful in understanding epidemiology of AMR bacterial infections					
Strongly agree / Agree	31.5% (152/483)	90.5% (258/285)	52.5% (240/457)	2.89 (1.60-5.19)	<0.001
Uncertain / Disagree / Strongly disagree	21.4% (3/14)	76.5% (13/17)	25% (10/40)	1.0	
BC is unnecessary because antibiotic therapy can be determined based on clinical presentation					
Strongly agree / Agree	20.8% (21/101)	83.6% (46/44)	33.8% (24/71)	0.51 (0.36-0.73)	<0.001
Uncertain / Disagree / Strongly disagree	33.9% (134/395)	91.1% (226/248)	53.3% (228/428)	1.0	
The therapeutic consequence of BC is questionable					
Strongly agree / Agree	32.3% (30/93)	88.0% (73/83)	41.0% (25/61)	0.84 (0.59-1.19)	0.32
Uncertain / Disagree / Strongly disagree	30.6% (120/392)	91.2% (196/215)	51.9% (223/430)	1.0	
The scientific basis of the guideline on BC is questionable					
Strongly agree / Agree	32.0% (17/53)	87.3% (69/79)	32.8% (19/58)	0.66 (0.45-0.98)	0.04
Uncertain / Disagree / Strongly disagree	30.4% (132/433)	91.2% (198/217)	53.2% (231/434)	1.0	
BC is unnecessary because results are often delayed					
Strongly agree / Agree	18.9% (24/127)	82.1% (32/39)	30.2% (16/53)	0.48 (0.33-0.69)	<0.001
Uncertain / Disagree / Strongly disagree	35.2% (129/367)	90.9% (240/264)	53.0% (236/445)	1.0	
BC is unnecessary because results are often not interpretable					
Strongly agree / Agree	25.0% (13/52)	77.3% (17/22)	29.7% (11/37)	0.54 (0.34-0.87)	0.01
Uncertain / Disagree / Strongly disagree	31.7% (140/442)	90.8% (255/281)	52.3% (241/461)	1.0	
BC is unnecessary because results are often negative or no growth					
Strongly agree / Agree	30.8% (20/65)	81.3% (26/32)	28.0% (14/50)	0.58 (0.39-0.88)	0.01
Uncertain / Disagree / Strongly disagree	30.8% (131/426)	91.1% (247/271)	53.1% (238/448)	1.0	
BC is unnecessary because cultures are often contaminated					
Strongly agree / Agree	26.3% (19/72)	79.3% (23/29)	34.2% (14/41)	0.64 (0.42-0.98)	0.04

For peer review only - http://bmjopen.bmj.com/site/about/guidelines.xhtml

Barriers or enablers	Indonesia[1] (n=503)	Thailand[1] (n=304)	Viet Nam[1] (n=501)	Odds ratio[2]	P value
Uncertain / Disagree / Strongly disagree	31.9% (133/417)	90.9% (249/274)	52.2% (236/452)	1.0	
BC is unnecessary because results often do not agree with clinical signs					
Strongly agree / Agree	34.0% (18/53)	88.9% (24/27)	23.5% (8/34)	0.77 (0.48-1.22)	0.27
Uncertain / Disagree / Strongly disagree	30.8% (135/439)	89.9% (249/277)	52.9% (241/456)	1.0	
BC is unnecessary because it is too expensive					
Strongly agree / Agree	25.5% (24/94)	80.0% (24/30)	32.7% (17/52)	0.62 (0.42-0.92)	0.02
Uncertain / Disagree / Strongly disagree	32.4% (129/398)	91.2% (249/273)	52.9% (229/443)	1.0	
BC is not benefiting the patients					
Strongly agree / Agree	14.0% (15/107)	84.0% (21/25)	19.4% (7/36)	0.37 (0.24-0.57)	<0.001
Uncertain / Disagree / Strongly disagree	35.8% (136/380)	90.1% (246/273)	53.0% (239/451)	1.0	
BC is unnecessary because a contaminated result often leads to wrong therapeutic approaches					
Strongly agree / Agree	30.4% (7/23)	86.4% (19/22)	20.0% (6/30)	0.53 (0.30-0.95)	0.03
Uncertain / Disagree / Strongly disagree	31.5% (148/470)	90.1% (254/282)	52.5% (245/467)	1.0	
It is not too late to collect BC later, particularly if patients do not improve after receiving empirical antibiotic treatment					
Strongly agree / Agree	13.8% (19/138)	88.3% (143/162)	31.2% (38/122)	0.37 (0.27-0.51)	<0.001
Uncertain / Disagree / Strongly disagree	38.1% (134/352)	91.6% (130/142)	57.2% (214/373)	1.0	
Quality of laboratory is questionable					
Strongly agree / Agree	24.2% (22/91)	84.2% (32/38)	26.6% (17/64)	0.48 (0.33-0.70)	<0.001
Uncertain / Disagree / Strongly disagree	32.7% (128/391)	90.3% (232/257)	54.1% (230/435)	1.0	
Levels of local antibiotic resistance are low					
Strongly agree / Agree	34.7% (17/49)	76.9% (20/26)	32.0% (16/50)	0.64 (0.41-0.98)	0.04
Uncertain / Disagree / Strongly disagree	31.3% (135/432)	91.1% (246/270)	52.8% (235/445)	1.0	
**TDF Domain: Memory, attention and decision processes**					
Deciding to do BC in patients presenting with sepsis					
Yes	34.1% (130/381)	90.2% (259/287)	54.2% (219/404)	1.79 (1.27-2.52)	0.001
No	22.5 (27/120)	82.4% (14/17)	34.7% (33/95)	1.0	
Deciding to do BC in patients presenting with septic shock					
Yes	35.2% (114/324)	89.8% (246/274)	50.7% (216/426)	1.27 (0.93-1.75)	0.14
No	24.3% (43/177)	90.0% (27/30)	49.3% (36/73)	1.0	
Deciding to do BC in patients starting parenteral antibiotic treatment					
Yes	47.0% (71/151)	93.1% (190/204)	65.0% (76/117)	2.32 (1.75-3.09)	<0.001
No	24.6% (86/350)	83.0% (83/100)	46.1% (176/382)	1.0	

For peer review only - http://bmjopen.bmj.com/site/about/guidelines.xhtml

Barriers or enablers	Indonesia[1] (n=503)	Thailand[1] (n=304)	Viet Nam[1] (n=501)	Odds ratio[2]	P value
Even when BC is recommended, would you still order BC if patients are already on antibiotics					
Definitely not order / likely not order	20.0% (6/30)	86.6% (58/67)	41.2% (14/34)	0.69 (0.42-1.11)	0.13
Maybe not order/ likely to still order / very likely to still order	31.2% (142/455)	90.6% (213/235)	51.3% (238/464)	1.0	
Even when BC is recommended, would you still order BC if patients have anemia					
Definitely not order / likely not order	21.3% (16/75)	91.9% (136/148)	47.4% (27/57)	0.89 (0.62-1.28)	0.55
Maybe not order/ likely to still order / very likely to still order	32.2% (128/398)	87.3% (130/149)	51.3% (220/429)	1.0	
**TDF: Optimism**					
Strongly optimistic / Optimistic	33.3% (133/400)	90.5% (238/263)	54.4% (200/368)	1.78 (1.29-2.46)	<0.001
Neither / Pessimistic / Strongly pessimistic	20.7% (18/87)	88.6% (31/35)	39.8% (51/128)	1.0	
**TDF: Skills**					
How skilled are you in drawing blood? [4]					
Very good / Good	38.5% (15/39)	88.2% (30/34)	57.1% (40/70)	1.74 (1.02-2.97)	0.04
Fair / Poor / Very poor	31.8% (14/44)	93.1% (81/87)	35.1% (20/57)	1.0	
**TDF: Beliefs about capabilities**					
Are you confident that you can draw blood successfully? [4,7]					
Strongly confident / Confident	34.7% (25/72)	89.1% (57/64)	51.9% (56/108)	1.39 (0.69-2.79)	0.36
Uncertain / Doubtful / Strongly doubtful	36.4% (4/11)	94.7% (54/57)	22.2% (4/18)	1.0	
Are you confident that you can draw blood appropriately? [4,7]					
Strongly confident / Confident	34.8% (24/69)	89.7% (70/78)	54.6% (54/99)	1.67 (0.88-3.17)	0.11
Uncertain / Doubtful / Strongly doubtful	35.7% (5/14)	95.2% (40/42)	22.2% (6/27)	1.0	
Are you confident that others (who are tasked to draw blood in your hospital) can draw blood successfully? [7]					
Strongly confident / Confident	30.7% (142/463)	90.1% (254/282)	52.5% (212/404)	1.35 (0.91-2.00)	0.13
Uncertain / Doubtful / Strongly doubtful	33.3% (11/33)	85.7% (18/21)	43.0% (40/93)	1.0	
Are you confident that others (who are tasked to draw blood in your hospital) can draw blood appropriately? [7]					
Strongly confident / Confident	31.0% (132/426)	89.6% (224/250)	52.8% (168/318)	1.20 (0.89-1.62)	0.23
Uncertain / Doubtful / Strongly doubtful	31.9% (22/69)	90.6% (48/53)	46.6% (83/178)	1.0	
**TDF: Emotion**					
Any emotional factors					
Yes	25.5% (13/51)	80% (8/10)	65.6% (21/32)	1.06 (0.65-1.71)	0.82
No	32.0% (144/450)	90.1% (265/294)	49.5% (231/467)	1.0	

For peer review only - http://bmjopen.bmj.com/site/about/guidelines.xhtml

[1] Percentage of participants who answered with "definitely take BC" in the case scenario are presented. For each question, participants who answered 'I do not know' or 'I do not want to answer' were excluded. [2] Estimated by using logistic regression models with random effects for countries, for types of hospital nested in the same country, and for professional roles nested in the same types of hospital. [3] Among those who answered that they know of local guidelines. [4] Among those who answered that their professional roles are tasked of drawing blood for BC. [5] "Norms" means usual practice that are typical of or accepted within your hospital. [6] Included answers in Q1-7 (which were asked to those who answered that they knew of local guideline) and Q1-8 (which were asked to those who answered that they did not know of local guideline) (Appendix S3). [7] "Successfully" means obtaining blood; "Appropriately" means that general recommendations for BC specimen collection such as aseptic technique are followed.

For peer review only

Appendix S8. Links between TDF, COM-B components (Capability, Opportunity, motivation and behaviour components), and suggested intervention types and policy options.

Links between TDF and COM-B components*

COM-B components		TDF Domains
**Capability**	**Psychological**	**Knowledge**<br>**Skills**<br>**Memory, attention and decision processes**<br>**Behavioural regulation**
	**Physical**	**Skills**
**Opportunity**	**Social**	**Social Influences**
	**Physical**	**Environmental Context and Resources**
**Motivation**	**Reflective**	**Social/professional role and Identity**<br>**Beliefs about capabalities**<br>**Optimism**<br>**Beliefs about Consequences**<br>**Intentions**<br>**Goals**
	**Automatic**	**Social/professional role and Identity**<br>**Optimism**<br>**Reinforcement**<br>**Emotion**

*as previously published.[39]

For peer review only

Links between COM-B components and intervention types*

**Intervention types**	**COM-B components**					
	**Capability**		**Opportunity**		**Motivation**	
	**Psychological**	**Physical**	**Social**	**Physical**	**Reflective**	**Automatic**
**Education**		X			X	
**Persuasion**					X	X
**Incentivisation**					X	X
**Coercion**					X	X

**Training**	X	X				
**Restriction**			X	X		
**Environmental restructuring**			X	X		X
**Modelling**						X
**Enablement**	X	X	X	X		X

* as previously published.[40]

Links between intervention types and policy categories*

**Intervention types**	**Policy categories**						
	**Communication /Marketing**	**Guidelines**	**Fiscal**	**Regulation**	**Legislation**	**Environmental/ social planning**	**Service Provision**
**Education**	X	X		X	X		X
**Persuasion**	X	X		X	X		X
**Incentivisation**	X	X	X	X	X		X
**Coerction**	X	X	X	X	X		X
**Training**		X	X	X	X		X
**Restriction**		X		X	X		
**Environmental restructuring**		X	X	X	X	X	
**Modelling**	X						X
**Enablement**		X	X	X	X	X	X

* as previously published.[40]

For peer review only

For peer review only - http://bmjopen.bmj.com/site/about/guidelines.xhtml

Appendix S9. References

1. Worldbank. GDP (current US$). https://data.worldbank.org/indicator/NY.GDP.MKTP.CD (accessed 11 May 2023).

2. Chongsuvivatwong V, Phua KH, Yap MT, et al. Health and health-care systems in southeast Asia: diversity and transitions. *Lancet* 2011; **377**(9763): 429-37.

3. Tangcharoensathien V, Limwattananon S, Patcharanarumol W, Thammatacharee J. Monitoring and evaluating progress towards Universal Health Coverage in Thailand. *PLoS Med* 2014; **11**(9): e1001726.

4. Sumriddetchkajorn K, Shimazaki K, Ono T, Kusaba T, Sato K, Kobayashi N. Universal health coverage and primary care, Thailand. *Bull World Health Organ* 2019; **97**(6): 415-22.

5. Le QN, Blizzard L, Si L, Giang LT, Neil AL. The evolution of social health insurance in Vietnam and its role towards achieving universal health coverage. *Health Policy OPEN* 2020; **1**: 100011.

6. Thuan NT, Lofgren C, Lindholm L, Chuc NT. Choice of healthcare provider following reform in Vietnam. *BMC Health Serv Res* 2008; **8**: 162.

7. WHO. Out-of-pocket expenditure (OOP) per capita in US$. https://www.who.int/data/gho/data/indicators/indicator-details/GHO/out-of-pocket-expenditure-(oop)-per-capita-in-us (accessed 11 May 2023).

8. Eng J. Sample size estimation: how many individuals should be studied? *Radiology* 2003; **227**(2): 309-13.

9. She RC, Alrabaa S, Lee SH, Norvell M, Wilson A, Petti CA. Survey of physicians' perspectives and knowledge about diagnostic tests for bloodstream infections. *PLoS One* 2015; **10**(3): e0121493.

10. Ojide CK, Onwuezobe IA, Asuquo EE, Obiagwu CS. Knowledge, attitude and practice of blood culture: A cross sectional study among medical doctors in a nigerian tertiary hospital. *African Journal of Clinical and Experimental Microbiology* 2013; **14**(3): 174-9.

11. Raupach-Rosin H, Duddeck A, Gehrlich M, et al. Deficits in knowledge, attitude, and practice towards blood culture sampling: results of a nationwide mixed-methods study among inpatient care physicians in Germany. *Infection* 2017; **45**(4): 433-41.

For peer review only - http://bmjopen.bmj.com/site/about/guidelines.xhtml

12. Dailey PJ, Osborn J, Ashley EA, et al. Defining System Requirements for Simplified Blood Culture to Enable Widespread Use in Resource-Limited Settings. *Diagnostics (Basel)* 2019; **9**(1).

13. Schmitz RP, Keller PM, Baier M, Hagel S, Pletz MW, Brunkhorst FM. Quality of blood culture testing - a survey in intensive care units and microbiological laboratories across four European countries. *Crit Care* 2013; **17**(5): R248.

14. WHO. Central Asian and Eastern European Surveillance of Antimicrobial Resistance. Annual Report 2018. 2018. https://www.euro.who.int/__data/assets/pdf_file/0007/386161/52238-WHO-CAESAR-AR-2018_low_V11_web.pdf (accessed 11 May 2023).

15. Kerur B, Salvador A, Arbeter A, Schutzman DL. Neonatal blood cultures: Survey of neonatologists' practices. *World Journal of Pediatrics* 2012; **8**(3): 260-2.

16. Thé T, Curfman A, Burnham CD, Hayes E, Schnadower D. Pediatric Anaerobic Blood Culture Practices in Industrialized Countries. *J Appl Lab Med* 2019; **3**(4): 553-8.

17. Tabriz MS, Riederer K, Baran J, Jr., Khatib R. Repeating blood cultures during hospital stay: Practice pattern at a teaching hospital and a proposal for guidelines. *Clin Microbiol Infect* 2004; **10**(7): 624-7.

18. Diallo K, Thilly N, Luc A, et al. Management of bloodstream infections by infection specialists: an international ESCMID cross-sectional survey. *Int J Antimicrob Agents* 2018; **51**(5): 794-8.

19. Donner LM, Campbell WS, Lyden E, Van Schooneveld TC. Assessment of Rapid-Blood-Culture-Identification Result Interpretation and Antibiotic Prescribing Practices. *J Clin Microbiol* 2017; **55**(5): 1496-507.

20. Peeters Grietens K, Ribera JM, Erhart A, et al. Doctors and vampires in sub-Saharan Africa: ethical challenges in clinical trial research. *Am J Trop Med Hyg* 2014; **91**(2): 213-5.

21. Gross PA, Van Antwerpen CL, Hess WA, Reilly KA. Use and abuse of blood cultures: Program to limit use. *American Journal of Infection Control* 1988; **16**(3): 114-7.

22. Salluzzo R, Reilly K. The rational ordering of blood cultures in the emergency department. *Qual Assur Util Rev* 1991; **6**(1): 28-31.

For peer review only

23. Parada JP, Schwartz DN, Schiff GD, Weiss KB. Effects of type and level of training on variation in physician knowledge in the use and acquisition of blood cultures: A cross sectional survey. *BMC Infectious Diseases* 2005; **5**

24. Falagas ME, Ierodiakonou V, Alexiou VG. Clinical Practice of Obtaining Blood Cultures From Patients with a Central Venous Catheter (CVC) in Place: an International Survey. *Abstracts of the Interscience Conference on Antimicrobial Agents and Chemotherapy* 2008; **48**: 530-.

25. Chew KS, Mohd Hashairi F, Jusoh AF, Aziz AA, Nik Hisamuddin NA, Siti Asma H. Knowledge of good blood culture sampling practice among healthcare staffs in an emergency department - Are we getting it right? *Medical Journal of Malaysia* 2013; **68**(4): 323-5.

26. Heine D, Cochran C, Moore M, Titus MO, Andrews AL. The prevalence of bacteremia in pediatric patients with community-acquired pneumonia: Guidelines to reduce the frequency of obtaining blood cultures. *Hospital Pediatrics* 2013; **3**(2): 92-6.

27. Pluye P, Hong QN. Combining the Power of Stories and the Power of Numbers: Mixed Methods Research and Mixed Studies Reviews. *Annual Review of Public Health* 2014; **35**(1): 29-45.

28. Pavese P, Maillet M, Vitrat-Hincky V, et al. Evaluation of an intervention to improve blood culture practices: a cluster randomised trial. *European Journal of Clinical Microbiology and Infectious Diseases* 2014; **33**(12): 2207-13.

29. Murtagh Kurowski E, Shah SS, Thomson J, et al. Improvement methodology increases guideline recommended blood cultures in children with pneumonia. *Pediatrics* 2015; **135**(4): e1052-9.

30. Ong I, Jayagobi PA, Raut P, Chong CY. Judicious utilization of healthcare resources: Reducing unindicated pediatric anaerobic blood cultures in a pediatric hospital. *J Healthc Qual* 2015; **37**(3): 199-205.

31. Casu S, Häske D. Severe sepsis and septic shock in pre-hospital emergency medicine: survey results of medical directors of emergency medical services concerning antibiotics, blood cultures and algorithms. *Internal and emergency medicine* 2016; **11**(4): 571-6.

32. Sloane AJ, Pressel DM. Culture Pus, Not Blood: Decreasing Routine Laboratory Testing in Patients With Uncomplicated Skin and Soft Tissue Infections. *Hosp Pediatr* 2016; **6**(7): 394-8.

For peer review only - http://bmjopen.bmj.com/site/about/guidelines.xhtml

33. Garcia RA, Spitzer ED, Kranz B, Barnes S. A national survey of interventions and practices in the prevention of blood culture contamination and associated adverse health care events. *Am J Infect Control* 2018; **46**(5): 571-6.

34. Idelevich EA, Seifert H, Sundqvist M, et al. Microbiological diagnostics of bloodstream infections in Europe—an ESGBIES survey. *Clin Microbiol Infect* 2019; **25**(11): 1399-407.

35. Tran P, Dowell E, Hamilton S, et al. Two blood cultures with age-appropriate volume enhance suspected sepsis decision-making. *Open Forum Infectious Diseases* 2020; **7**(2).

36. Jaglal MV, Laber DA, Arnold FW, Miller DM, Chesney JA, Kloecker GH. Obtaining routine blood cultures during interleukin-2-containing therapy is unnecessary. *American Journal of Clinical Oncology: Cancer Clinical Trials* 2009; **32**(4): 429-31.

37. Yaddanapudi LN. The American Statistical Association statement on P-values explained. *J Anaesthesiol Clin Pharmacol* 2016; **32**(4): 421-3.

38. Sterne JA, Davey Smith G. Sifting the evidence-what's wrong with significance tests? *BMJ* 2001; **322**(7280): 226-31.

39. Cane J, O’Connor D, Michie S. Validation of the theoretical domains framework for use in behaviour change and implementation research. *Implementation Science* 2012; **7**(1): 37.

40. Michie S, van Stralen MM, West R. The behaviour change wheel: A new method for characterising and designing behaviour change interventions. *Implementation Science* 2011; **6**(1): 42.

For peer review only

For peer review only - http://bmjopen.bmj.com/site/about/guidelines.xhtml

STROBE Statement—Checklist of items that should be included in reports of ***cross-sectional studies***

	Item No	Recommendation	Page No
**Title and abstract**	1	(*a*) Indicate the study's design with a commonly used term in the title or the abstract	3
		(*b*) Provide in the abstract an informative and balanced summary of what was done and what was found	3-4
**Introduction**			
Background/rationale	2	Explain the scientific background and rationale for the investigation being reported	6-7
Objectives	3	State specific objectives, including any prespecified hypotheses	7
**Methods**			
Study design	4	Present key elements of study design early in the paper	7
Setting	5	Describe the setting, locations, and relevant dates, including periods of recruitment, exposure, follow-up, and data collection	9-10
Participants	6	(*a*) Give the eligibility criteria, and the sources and methods of selection of participants	9-10
Variables	7	Clearly define all outcomes, exposures, predictors, potential confounders, and effect modifiers. Give diagnostic criteria, if applicable	8-11
Data sources/ measurement	8*	For each variable of interest, give sources of data and details of methods of assessment (measurement). Describe comparability of assessment methods if there is more than one group	8-11
Bias	9	Describe any efforts to address potential sources of bias	Appendix S1
Study size	10	Explain how the study size was arrived at	9-10
Quantitative variables	11	Explain how quantitative variables were handled in the analyses. If applicable, describe which groupings were chosen and why	10-11
Statistical methods	12	(*a*) Describe all statistical methods, including those used to control for confounding	10-11
		(*b*) Describe any methods used to examine subgroups and interactions	10-11
		(*c*) Explain how missing data were addressed	10-11
		(*d*) If applicable, describe analytical methods taking account of sampling strategy	10-11
		(*e*) Describe any sensitivity analyses	10-11 Appendix S1
**Results**			
Participants	13*	(a) Report numbers of individuals at each stage of study—eg numbers potentially eligible, examined for eligibility, confirmed eligible, included in the study, completing follow-up, and analysed	11
		(b) Give reasons for non-participation at each stage	Not applicable
		(c) Consider use of a flow diagram	Not applicable

For peer review only - http://bmjopen.bmj.com/site/about/guidelines.xhtml

Descriptive data	14*	(a) Give characteristics of study participants (eg demographic, clinical, social) and information on exposures and potential confounders	11
		(b) Indicate number of participants with missing data for each variable of interest	Appendix S1
Outcome data	15*	Report numbers of outcome events or summary measures	12-20 Appendix S5
Main results	16	(*a*) Give unadjusted estimates and, if applicable, confounder-adjusted estimates and their precision (eg, 95% confidence interval). Make clear which confounders were adjusted for and why they were included	12-20 Appendix S1, S5, S7
		(*b*) Report category boundaries when continuous variables were categorized	12-20 Appendix S1, S5, S7
		(*c*) If relevant, consider translating estimates of relative risk into absolute risk for a meaningful time period	12-20 Appendix S7
Other analyses	17	Report other analyses done—eg analyses of subgroups and interactions, and sensitivity analyses	Appendix S1
**Discussion**			
Key results	18	Summarise key results with reference to study objectives	22
Limitations	19	Discuss limitations of the study, taking into account sources of potential bias or imprecision. Discuss both direction and magnitude of any potential bias	24
Interpretation	20	Give a cautious overall interpretation of results considering objectives, limitations, multiplicity of analyses, results from similar studies, and other relevant evidence	22-24
Generalisability	21	Discuss the generalisability (external validity) of the study results	24
**Other information**			
Funding	22	Give the source of funding and the role of the funders for the present study and, if applicable, for the original study on which the present article is based	25

*Give information separately for exposed and unexposed groups.

Note: An Explanation and Elaboration article discusses each checklist item and gives methodological background and published examples of transparent reporting. The STROBE checklist is best used in conjunction with this article (freely available on the Web sites of PLoS Medicine at http://www.plosmedicine.org/, Annals of Internal Medicine at http://www.annals.org/, and Epidemiology at http://www.epidem.com/). Information on the STROBE Initiative is available at www.strobe-statement.org.

For peer review only

BMJ Open

Barriers and enablers to blood culture sampling in Indonesia, Thailand and Vietnam: a Theoretical Domains Framework-based survey

Journal:	*BMJ Open*
Manuscript ID	bmjopen-2023-075526.R1
Article Type:	Original research
Date Submitted by the Author:	27-Dec-2023
Complete List of Authors:	Suntornsut, Pornpan; Mahidol Oxford Tropical Medicine Research Unit<br>Asadinia, Koe Stella; Eijkman-Oxford Clinical Research Unit<br>Limato, Ralalicia; Oxford University, Eijkman-Oxford Clinical Research Unit; Oxford University, Centre for Tropical Medicine and Global Health, Nuffield Department of Medicine<br>Tamara, Alice; Faculty of Medicine Universitas Indonesia<br>Rotty, Linda W.A.; Prof Dr RD Kandou Central Hospital<br>Bramanti, Rendra; Dr. Iskak District Hospital<br>Nusantara, Dwi U.; Pasar Minggu District Hospital<br>Nelwan, Erni J.; Universitas Indonesia; Cipto Mangukusumo National General Hospital, Department of Internal Medicine<br>Khusuwan, Suwimon; Chiangrai Prachanukroh Hospital<br>Suphamongkholchaikul, Watthanapong; Sunpasitthiprasong Hospital<br>Chamnan, Parinya; Sunpasithiprasong Hospital<br>Piyaphanee, Watcharapong; Mahidol University<br>Vu, Huong; Oxford University Clinical Research Unit Viet Nam, Hanoi Unit<br>Hai Yen, Nguyen; Oxford University Clinical Research Unit<br>Nguyen Hong, Khanh; Oxford University Clinical Research Unit<br>Pham, Ngoc Thach; National Hospital of Tropical Diseases<br>Le, Minh Quang; Viet Tiep Hospital<br>Vu, Hai Vinh; Assistance Publique des Hôpitaux de Marseille, Pôle de Maladies Infectieuses,<br>Duc, Chau Minh; Dong Thap Hospital<br>Vo, Thi Hoang Dung Em; Oxford University Clinical Research Unit<br>Harriss, Elinor; University of Oxford, Bodleian Health Care Libraries<br>van Doorn, H. Rogier; Oxford University Clinical Research Unit; Nuffield Department of Clinical Medicine<br>Hamers, Raph Leonardus; Eijkman-Oxford Clinical Research Unit; Centre for Tropical Medicine and Global Health, Nuffield Department of Medicine, University of Oxford,<br>Lorencatto, Fabiana; UCL<br>Atkins, Lou; UCL<br>Limmathurotsakul, Direk; Mahidol University; University of Oxford, Nuffield Department of Medicine
**Primary Subject Heading**:	Diagnostics
Secondary Subject Heading:	Diagnostics, Emergency medicine, Health services research, Infectious

	diseases
Keywords:	Diagnostic microbiology < INFECTIOUS DISEASES, Public Hospitals < Hospitals, Public, INFECTIOUS DISEASES, MICROBIOLOGY, QUALITATIVE RESEARCH, Surveys and Questionnaires

SCHOLARONE™
Manuscripts

I, the Submitting Author has the right to grant and does grant on behalf of all authors of the Work (as defined in the below author licence), an exclusive licence and/or a non-exclusive licence for contributions from authors who are: i) UK Crown employees; ii) where BMJ has agreed a CC-BY licence shall apply, and/or iii) in accordance with the terms applicable for US Federal Government officers or employees acting as part of their official duties; on a worldwide, perpetual, irrevocable, royalty-free basis to BMJ Publishing Group Ltd ("BMJ") its licensees and where the relevant Journal is co-owned by BMJ to the co-owners of the Journal, to publish the Work in this journal and any other BMJ products and to exploit all rights, as set out in our licence.

The Submitting Author accepts and understands that any supply made under these terms is made by BMJ to the Submitting Author unless you are acting as an employee on behalf of your employer or a postgraduate student of an affiliated institution which is paying any applicable article publishing charge ("APC") for Open Access articles. Where the Submitting Author wishes to make the Work available on an Open Access basis (and intends to pay the relevant APC), the terms of reuse of such Open Access shall be governed by a Creative Commons licence – details of these licences and which Creative Commons licence will apply to this Work are set out in our licence referred to above.

Other than as permitted in any relevant BMJ Author's Self Archiving Policies, I confirm this Work has not been accepted for publication elsewhere, is not being considered for publication elsewhere and does not duplicate material already published. I confirm all authors consent to publication of this Work and authorise the granting of this licence.

For peer review only

Barriers and enablers to blood culture sampling in Indonesia, Thailand and Vietnam: a Theoretical Domains Framework-based survey

Pornpan Suntornsut, MSc[1], Koe Stella Asadinia, MD[2], Ralalicia Limato, MD[2,3], Alice Tamara, MD[2], Linda W.A. Rotty, MD[4], Rendra Bramanti, MD[5], Dwi U. Nusantara, MD[6], Erni J. Nelwan, MD[7,8] Suwimon Khusuwan, MD[9], Watthanapong Suphamongkholchaikul, MD[10], Parinya Chamnan, MD[10], Watcharapong Piyaphanee, MD[11], Vu Thi Lan Huong, MD[12], Nguyen Hai Yen, MPH[12], Khanh Nguyen Hong, MSc[12], Pham Ngoc Thach, MD[13], Le Minh Quang, MD[14], Vu Hai Vinh, PhD[14], Chau Minh Duc, PhD[15], Vo Thi Hoang Dung Em MD[15], Elinor Harriss, MSc[16], H Rogier van Doorn, MD[3,12], Raph L. Hamers MD[2,3], Fabiana Lorencatto, PhD[17], Lou Atkins, PhD[17], Direk Limmathurotsakul, MD[1,3,18]

[1] Mahidol-Oxford Tropical Medicine Research Unit, Faculty of Tropical Medicine, Mahidol University, Bangkok, Thailand

[2] Oxford University Clinical Research Unit Indonesia, Faculty of Medicine Universitas Indonesia, Jakarta, Indonesia

[3] Centre for Tropical Medicine and Global Health, Nuffield Department of Medicine, University of Oxford, Oxford, UK

[4] Prof. Dr. R.D. Kandou Central Hospital, Manado, Indonesia

[5] Dr. Iskak District Hospital, Tulungagung, Indonesia

[6] Pasar Minggu District Hospital, Jakarta, Indonesia

[7] Faculty of Medicine, Universitas Indonesia, Jakarta, Indonesia

For peer review only

For peer review only - http://bmjopen.bmj.com/site/about/guidelines.xhtml

[8] Department of Internal Medicine, Division of Infectious Diseases, Cipto Mangukusumo National General Hospital, Jakarta, Indonesia

[9] Chiangrai Prachanukroh Hospital, Chiang Rai, Thailand

[10] Sunpasitthiprasong Hospital, Ubon Ratchathani, Thailand

[11] Hospital for Tropical Diseases, Faculty of Tropical Medicine, Mahidol University, Bangkok, Thailand

[12] Oxford University Clinical Research Unit, Hanoi, Vietnam.

[13] National Hospital for Tropical Diseases, 78 Giai Phong, Ha Noi, Vietnam

[14] Viet Tiep Hospital, 1 Nha Thuong, Cat Dai, Le Chan, Hai Phong, Vietnam

[15] Dong Thap Hospital, 144 Mai Van Khai, My Tan, Cao Lanh City, Dong Thap, Vietnam

[16] Bodleian Health Care Libraries, University of Oxford, Oxford, UK

[17] Centre for Behaviour Change, University College London, London, UK

[18] Department of Tropical Hygiene, Faculty of Tropical Medicine, Mahidol University, Bangkok, Thailand

* Correspondence to:

Direk Limmathurotsakul

Mahidol-Oxford Tropical Medicine Research Unit, Faculty of Tropical Medicine, Mahidol University, Bangkok, Thailand, 10400

Email: direk@tropmedres.ac

For peer review only

Abstract

Objective

Blood culture (BC) sampling is recommended for all suspected sepsis patients prior to antibiotic administration. We examine barriers and enablers to BC sampling in three Southeast Asian countries.

Design

A Theoretical Domains Framework (TDF)-based survey, comprising a case scenario of a patient presenting with community-acquired sepsis and all 14 TDF domains of barriers/enablers to BC sampling.

Setting

Hospitals in Indonesia, Thailand and Vietnam, December 2021 to 30 April 2022.

Participants

1,070 medical doctors and 238 final-year medical students. Half were female (n=680, 52%) and most worked in governmental hospitals (n=980, 75.4%).

Outcome measures

Barriers and enablers to blood culture sampling.

Results

The proportion of respondents who answered that they would definitely take BC in the case scenario was highest at 89.8% (273/304) in Thailand, followed by 50.5% (252/499) in Vietnam and 31.3% (157/501) in Indonesia ($p<0.001$). Barriers/enablers in nine TDF domains were considered key in influencing BC sampling, including 'priority of BC [TDF-goals]', 'perception about their role to order or initiate an order for BC [TDF-social professional role and identity]', 'perception that BC is helpful [TDF-beliefs about consequences]', 'intention to follow guidelines

For peer review only

[TDF-intention]', 'awareness of guidelines [TDF-knowledge]', 'norms of BC sampling [TDF-social influence]', 'consequences that discourage BC sampling [TDF-reinforcement]', 'perceived cost-effectiveness of BC [TDF-environmental context and resources]' and 'regulation on cost reimbursement [TDF-behavioural regulation]'. There was substantial heterogeneity between the countries. In most domains, the lower (higher) proportion of Thai respondents experienced the barriers (enablers) compared to that of Indonesian and Vietnamese respondents. A range of suggested intervention types and policy options were identified.

Conclusions

Barriers and enablers to BC sampling are varied and heterogenous. Cost-related barriers are more common in more resource-limited countries, while many barriers are not directly related to cost. Context-specific multifaceted interventions at both hospital and policy levels are required to improve diagnostic stewardship practices.

For peer review only

Strengths and limitations of this study

- The Theoretical Domains Framework-based survey comprehensively identified individual, socio-cultural and environmental barriers and enablers to blood culture sampling across study countries.
- A convenience sampling approach, distributing invitations in letters, emails, pamphlets and online social media platforms, through existing collaborations in hospitals in the three survey countries was used and might have led to selection bias.
- The target sample size was not reached in Thailand.
- The findings may not be generalisable to all low and middle-income countries because barriers and enablers to blood culture sampling can be varied and local evaluations are needed.

For peer review only

For peer review only - http://bmjopen.bmj.com/site/about/guidelines.xhtml

INTRODUCTION

Blood culture (BC) is a crucial diagnostic, which can guide antibiotic treatment decisions of severe bacterial infections, and may improve patient outcomes.[1, 2] The cumulative results of BC are also crucial to inform antimicrobial resistance (AMR) surveillance, at the hospital, country and global levels.[3] International guidelines on sepsis management have been stressing the importance of obtaining BC before or, when not possible, within 24 hours after administration of antibiotics.[1, 4]

Nonetheless, BC is generally underutilised, both in high-income countries (HICs) and low and middle-income countries (LMICs), with wide variations in reported BC sampling rates between hospitals and global regions. Reported BC sampling rates ranged from 196 to 308 per 1,000 patient-days in the United States,[5, 6] from 6.7 to 86.5 per 1,000 patient-days in the European Union,[7] from 0 to 82 per 1,000 patient-days in the Central Asian and European Surveillance of AMR network (CAESAR),[8] and 31, 82 and 10 per 1,000 patient-days in selected hospitals in Indonesia,[9] Thailand[10] and Vietnam,[11] respectively.

A range of barriers and enablers have been identified that influence BC sampling, based on different study designs, theories and frameworks. Lack of clear guidelines, training, microbiological infrastructure, and positive attitudes regarding BC among medical practitioners, are commonly reported barriers.[8, 12-15]

Changing the behaviour of medical practitioners is complex, and a systematic approach has been shown useful to understand factors influencing adherence to guidelines or recommendations so

For peer review only - http://bmjopen.bmj.com/site/about/guidelines.xhtml

as to inform the design of future interventions.[16-18] The Theoretical Domains Framework (TDF) has been developed by synthesising a wide range of theories, and enables researchers to investigate a broader range of individual, socio-cultural and environmental behavioural influences than they would with a single theory alone.[16-18] The TDF has been widely used to explore barriers and enablers to healthcare professional behaviours, including diagnostic testing, antimicrobial stewardship, and infection prevention control.[19-22]

Here, we aimed to identify barriers and enablers to BC sampling in three middle-income countries in Southeast Asia (SEA) using a theory-based approach informed by the TDF.

METHODS

The TDF survey

We developed a TDF survey questionnaire, comprising a hypothetical case scenario and all 14 TDF domains of barriers/enablers to BC sampling, through an iterative process of systematic literature review and previous TDF surveys on other health topics (Table 1; Appendix S1 and S2).[23-26] Each question used a five-point Likert scale representing the level of perceived barriers/enablers to BC sampling under all TDF domains.

For peer review only

91 **Table 1.** Key questions for barriers and enablers to blood culture (BC) sampling

TDF Domains	Questions
Knowledge	Do you know of any recommendation(s) or guideline(s) for BC sampling being used in your hospital?
	Are you aware of any international recommendation(s) or guideline(s) for blood culture sampling?
	In your hospital, are there any training, lectures, classes or meetings that provide you knowledge about local/national/international guidelines for BC sampling?
Skills	In your current hospital setting, which types of professionals are tasked to draw blood from patients for BC?
	How skilled are you in drawing blood?
Social professional role and identity	In your current hospital setting, which types of professionals/staff can order BC?
	Do you think that it is an appropriate part of your current job to order BC?
	Do you think that it is an appropriate part of your current job to draw blood for BC?
Beliefs about capabilities	If you have to draw blood yourself, are you confident that you can draw blood successfully? "Successfully" means obtaining blood.
	Are you confident that others (who are tasked to draw blood in your hospital) can draw blood successfully?
	Are you confident that you can draw blood appropriately? "Appropriately" means that general recommendations for blood culture specimen collection such as aseptic technique are followed.
	Are you confident that others (who are tasked to draw blood in your hospital) can draw blood appropriately?
Optimism	In your current hospital setting, how optimistic are you that a BC will be sampled and processed in the laboratory appropriately if you order a BC?
Beliefs about consequences	Do you agree or disagree about the following potential advantages of BC, making BC helpful in your current hospital setting?
	Do you agree or disagree about the following disadvantages of BC, making BC unnecessary in your current hospital setting?
Reinforcement	Are there any positive consequences to you, if you order BC when recommended?
	Are there any negative consequences to you, if you do not order BC when recommended?
	Are there any negative consequences to you, if you order BC when recommended?
Intentions	How often do you plan to follow the recommendation(s) or guideline(s) for BC sampling being used in your hospital?
Goals	How often do you obtain BC prior to administration of empirical antibiotics in patients presenting with sepsis?
Memory, attention and decision processes	Apart from the recommendation(s) or guideline(s) being used at your hospital, do you have any additional reasons for deciding to do BC sampling?
	Would you still order blood culture in case patients are already on antibiotics?
	Would you still order blood culture in case patients have anaemia?
Environmental context and resources	Regardless of who pays for the cost of BC, would you say that the benefits of BC outweigh the cost?
	How often do patients have to pay for BC using their own money (i.e. out of pocket)?
	Do you consider whether patients can afford the cost of BC as a reason for deciding to do BC sampling?
	In your hospital, how often could you not order BC because consumables (such as blood culture bottles, needles, syringes, blood collection set, etc.) are not available?
Social influences	To what extent do you order BC sampling because you are following local norms? "Norms" mean usual practice that are typical of or accepted within your hospital.

For peer review only - http://bmjopen.bmj.com/site/about/guidelines.xhtml

	Do following people (such as consultants, head of the department, executives of the hospital, patients and family of patients) have any positive or negative influence on you to order BC?
Emotion	Apart from your logical considerations, do you think that any emotional factors of anyone are involved in ordering and sampling for BC
Behavioural regulation	In your hospital, are there any procedures that support you to order or regulate ordering of BC per local/national/international guidelines?
	Do you consider whether patients have a health scheme or insurance that covers the cost of BC as a reason for deciding to do BC sampling?

TDF = Theoretical Domain Framework.

For peer review only

The initial questionnaire was translated into Thai, Vietnamese and Indonesian language and piloted among 10-19 medical doctors and 3-6 final-year medical students in each country (a total of 54 respondents) to test the clarity of questions and choice answers in each language and to ensure no potential key barriers/enablers were omitted. We asked respondents to complete the survey and provide feedback using 1:1 interviews via phone or using online meeting software. The questionnaire was revised and finalised based on the pilot study results. During the pilot survey, we included 'monetary reward' and 'monetary fine' as examples of positive and negative consequences to BC sampling, respectively. We received strong feedback that those are not present for BC sampling in Indonesia, Thailand and Vietnam. Therefore, the word 'monetary reward' and 'monetary fine' were removed. One free-text question was added (i.e. Question 6-5, "Additional comments about emotional factors…"), a total of 27 choice answers were added, and languages and wordings were revised. The final questionnaire included 54 questions about barriers/enablers to BC sampling and respondents' demographic characteristics (Appendix S3).

Study participants

We invited medical doctors and final-year medical doctors in Indonesia, Thailand and Vietnam to complete the online TDF survey. We used a convenience sampling approach, distributing

For peer review only - http://bmjopen.bmj.com/site/about/guidelines.xhtml

invitations in letters, emails, pamphlets and online social media platforms, through existing collaborations in hospitals in the three survey countries. The online cross-sectional survey was conducted using the Qualtrics survey platform. Multiple participation was prevented by using the Prevent Ballot Box Stuffing Option within Qualtrics.

We used a simple formula for calculating the sample size.[27] Assuming prevalence of a barrier or enabler to be 50% among medical doctors, with a margin of error 5%, the sample size of medical doctors was estimated to be at least 385 per country. Assuming prevalence of a barrier or enabler to be 50% among final-year medical students, with a margin of error 10%, the sample size of final-year medical students was estimated to be at least 97 per country. Therefore, we aimed to enrol 400 medical doctors and 100 final-year medical students in each country (a total of 1,500 respondents).

Analysis

For each question, we defined that respondents who answered “definitely”/“likely”, “all the time”/“often” or “strongly agree”/“agree” perceived the importance or agreement with that barrier/enabler. The proportion of respondents who answered likewise, after excluding respondents who answered ‘I do not know’ or ‘I do not want to answer’, was presented. Groups were compared by Chi-squared or Fisher exact tests as appropriate. Logistic regression models with random effects for countries, for hospital type nested in the same country, and for professional roles nested in the same hospital type were used to evaluate the association between respondents’ answers about each barrier/enabler and to the case scenario. Multivariable logistic regression model was not used because we considered that each key TDF domain could

For peer review only - http://bmjopen.bmj.com/site/about/guidelines.xhtml

influence BC sampling practice via a causal relationship and should be addressed in future interventions. Statistical analyses were performed using Stata 15.1 (StataCorp, US).

We identified and ranked important TDF domains by scoring them based on an established set of four ‘importance criteria’ (modified from a previous TDF study[28]): (a) ‘frequency’ (the proportion of respondents who perceived the importance or agreement with a barrier/enabler); (b) ‘elaboration’ (number of themes within each domain); (c) ‘expressed importance’ (quotes from respondents expressing importance or agreement); and (d) ‘association between reported barriers/enablers and BC practice’ (size of effect and strength of association, i.e., odds ratios [ORs] and p values, obtained from the logistic regression models, respectively). P values <0.05 was not used as a simple cutoff whether an association was present or absent [29, 30]. P values less than 0.001 was regarded as providing strong evidence against the null hypothesis. For a negative association (OR<1.0), the inversed OR (1/OR) was considered as the size effect when compared with other positive associations. Overall rank was decided based on detailed presentation of the ratings of each criterion.

Lastly, we mapped identified TDF domains to the COM-B ('Capability', 'Opportunity', 'Motivation' and 'Behaviour') model (Table 2).[16-18] COM-B forms the hub of the Behaviour Change Wheel (BCW), a framework which signposts to potentially relevant intervention strategies. This allowed us to list all intervention types and policy options that were likely to be effective in addressing identified barriers and enablers.

For peer review only

For peer review only - http://bmjopen.bmj.com/site/about/guidelines.xhtml

159 **Table 2.** Links between TDF and COM-B components*

COM-B components		TDF Domains
**Capability**	**Psychological**	**Knowledge**
		**Skills**
		**Memory, attention and decision processes**
		**Behavioural regulation**
	**Physical**	**Skills**
**Opportunity**	**Social**	**Social Influences**
	**Physical**	**Environmental Context and Resources**
**Motivation**	**Reflective**	**Social/professional role and Identity**
		**Beliefs about capabilities**
		**Optimism**
		**Beliefs about Consequences**
		**Intentions**
		**Goals**
	**Automatic**	**Social/professional role and Identity**
		**Optimism**
		**Reinforcement**
		**Emotion**

160

161 * COM-B component stands for Capability (Physical capability or Psychological capability), Opportunity (Physical
162 opportunity or Social opportunity), Motivation (Automatic motivation or Reflective motivation)–Behaviour,
163 represents source of the behaviours and is the core of the Behaviour Change Wheel (BCW).[16-18]

164

165

For peer review only - http://bmjopen.bmj.com/site/about/guidelines.xhtml

Patient and public involvement

None.

RESULTS

From 1 December 2021 to 30 April 2022, 1,070 medical doctors and 238 final-year medical students in Indonesia, Thailand and Vietnam completed the online TDF survey. Half of respondents were female (n=680, 52%) and most worked in governmental hospitals (n=980, 75.4%) (Table 3 and Appendix S4). The most common department was internal medicine (n=450, 34.4%), followed by emergency (n=175, 13.4%) and paediatrics (n=153, 11.7%). Respondents were from 24 of 34 provinces in Indonesia, 39 of 77 provinces in Thailand, and 25 of 63 provinces in Vietnam.

For peer review only

Table 3. Demographics and responses to the hypothetical case scenario

Variables	Indonesia (n=503)	Thailand (n=304)	Vietnam (n=501)	P values
**Female gender**	263 (52.3%)	195 (64.1%)	222 (44.3%)	<0.001
**Hospital types**				
Government hospital	340 (67.6%)	209 (68.8%)	431 (86.0%)	<0.001
Private hospital	113 (22.5%)	15 (4.9%)	17 (3.4%)	
University hospital	26 (5.2%)	76 (25.0%)	29 (5.8%)	
Other *	19 (3.8%)	2 (0.7%)	22 (4.4%)	
I do not want to answer	5 (1.0%)	2 (0.7%)	2 (0.4%)	
**Hospital bed size**				
<200	99 (19.7%)	35 (11.5%)	24 (4.8%)	<0.001
201-400	107 (21.3%)	46 (15.1%)	29 (5.8%)	
401-600	72 (14.3%)	39 (12.8%)	62 (12.4%)	
601-1,000	66 (13.1%)	45 (14.8%)	144 (28.7%)	
1,001-2,000	39 (7.8%)	82 (27.0%)	125 (25.0%)	
>2,000	27 (5.4%)	30 (9.9%)	74 (14.8%)	
I do not know	89 (17.7%)	27 (8.9%)	35 (7.0%)	
I do not want to answer	4 (0.8%)	0 (0%)	8 (1.6%)	
**Current job** **				
Medical doctor – executive level	13 (2.6%)	5 (1.6%)	17 (3.4%)	<0.001
Medical doctor – consultant level	74 (14.7%)	75 (24.7%)	198 (39.5%)	
Medical doctor – physician level	124 (24.7%)	38 (12.5%)	112 (22.4%)	
Medical doctor – resident level	168 (33.4%)	63 (20.7%)	101 (20.2%)	
Medical doctor – intern level	33 (6.6%)	35 (11.5%)	14 (2.8%)	
Final-year medical student	91 (18.1%)	88 (28.9%)	59 (11.8%)	
**Department**				
Internal medicine	149 (29.6%)	155 (51.0%)	146 (29.1%)	<0.001
Paediatrics	65 (12.9%)	43 (14.1%)	45 (9.0%)	0.05
Infection disease division/department	12 (2.4%)	5 (1.6%)	56 (11.2%)	<0.001
Surgery	21 (4.2%)	45 (14.8%)	81 (16.2%)	<0.001
Orthopaedics	6 (1.2%)	18 (5.9%)	14 (2.8%)	0.001
Obstetrics / Gynaecology	20 (4.0%)	29 (9.5%)	7 (1.4%)	<0.001
Emergency department	112 (22.3%)	34 (11.2%)	29 (5.8%)	<0.001
Intensive care unit	45 (8.9%)	13 (4.3%)	51 (10.2%)	0.01
**Would you take a blood culture sample in the hypothetical case scenario (presenting with community-acquired sepsis)?** ***				
Definitely (>95-100% of the time)	157 (31.2%)	273 (89.8%)	252 (50.3%)	<0.001
Likely (75-95% of the time)	138 (27.4%)	23 (7.6%)	149 (29.7%)	
Maybe (25-74% of the time)	116 (23.1%)	5 (1.6%)	70 (14.0%)	
Unlikely (5-24% of the time)	44 (8.7%)	2 (0.7%)	19 (3.8%)	
Rarely (ranging from never to <5% of the time)	46 (9.1%)	1 (0.3%)	9 (1.8%)	
I do not know	1 (0.2%)	0 (0%)	1 (0.2%)	
I do not want to answer	1 (0.2%)	0 (0%)	1 (0.2%)	

* Included clinics (n=3) and text answers that could not be used to determine the hospital type such as internship and medical students. ** In the survey, for a medical doctor, 'executive level' was defined as having an administrative position without clinical work, 'consultant' was defined as having a clinical specialty degree, 'resident' as currently under postgraduate clinical training, 'physician' as having no clinical specialty/subspecialty degree and not under postgraduate clinical training, and 'intern' as a recent medical school graduate in the first year of post-graduate on-the-job training. *** Hypothetical case scenario. "A 72-year-old woman who was brought to the emergency

For peer review only - http://bmjopen.bmj.com/site/about/guidelines.xhtml

For peer review only

department of your hospital by her daughter when she noticed the patient was more confused than her baseline and was found to have a high fever and fast breathing. She had an auscultatory finding compatible with pneumonia. It is decided that this patient will be admitted to your hospital.” If you have an authority to take a blood culture, would you take blood culture sample(s) in this case on admission?

Based on the case scenario of a patient presenting with community-acquired sepsis, half of respondents (52.3%, 682/1,304) answered that they would definitely take BC. However, the responses were significantly different between the three countries ($p<0.001$). Most Thai respondents (89.8%, 273/304) answered that they would definitely take BC, while half of Vietnamese respondents (50.5%, 252/499) and about a third of Indonesian respondents (31.3%, 157/501) did.

Using an established set of four ‘importance criteria’, we ranked important TDF domains by scoring as shown in Table 4. We present, in rank order, the nine TDF domains that were considered very important (i.e. key) in the three countries in SEA in the section below.

For peer review only

Table 4. Criteria and rank of TDF domains regarding barriers/enablers to BC sampling

**TDF domains**	(1) 'Frequency' or the proportion of respondents who perceived the importance or agreement with a barrier/enabler within each domain *	(2) 'Elaboration' or number of themes within each domain **	(3) 'Expressed importance' or quotes from respondents expressing importance or agreement with a barrier/enabler within each domain ***	(4) 'Association between reported barriers or enablers and BC practice' or size of effect and strength of association, i.e. odds ratio [OR] and p values, obtained from the logistic regression model, respectively ****	Overall rank *****
Goals	Moderate (25-74%)	1	A few quotes	OR 4.25, strongly associated	Very important
Social professional role and identity	High (75-95%)	3	A few quotes	OR 3.36, strongly associated	Very important
Beliefs about consequences	High (75-95%)	2	A number of quotes	OR 2.96, strongly associated	Very important
Intentions	Moderate (25-74%)	1	A few quotes	OR 2.92, strongly associated	Very important
Knowledge	Moderate (25-74%)	2	A few quotes	OR 2.55, strongly associated	Very important
Social influences	Moderate (25-74%)	2	A number of quotes	OR 2.20, strongly associated	Very important
Reinforcement	Moderate (25-74%)	2	A number of quotes	OR 0.48, strongly associated	Very important
Behavioural regulation	Moderate (25-74%)	2	A number of quotes	OR 1.65, strongly associated	Very important
Environmental context and resources	High (75-95%)	3	A number of quotes	OR 1.63, strongly associated	Very important
Emotion	Low (5-24%)	2	A number of quotes	Not observed	Important
Optimism	High (75-95%)	1	None	OR 1.78, strongly associated	Important
Skills	Moderate (25-74%)	1	None	OR 1.74, associated	Important
Memory, attention and decision processes	Moderate (25-74%)	2	A few quotes	Not observed	Important
Beliefs about capabilities	Moderate (25-74%)	2	None	Not observed	Important

* For each question, we defined that respondents who answered "definitely"/"likely", "all the time"/"often" or "strongly agree"/"agree" perceived the importance or agreement with that barrier/enabler. The highest proportion for a barrier/enabler in each domain is presented. Details are present in the Appendix S4 ** Additional details are presented in the Appendix S1 *** Details are presented in the Appendix S5. **** Details are present in the Appendix S6 ***** Overall rank was decided based on detailed presentation of the ratings of each criterion.

For peer review only

TDF-goals

TDF-goals domain covers mental representations of outcomes that an individual wants to achieve, goal priority and implementation intention.[16-18]

Theme: Priority of BC

In many settings, ordering or initiating an order for BC can take only few seconds by writing “blood culture” in the doctor order form. We used a question asking about the priority of BC compared to that of empirical antibiotics, and 91.3% (274/300) of Thai respondents answered that they obtain BC prior to administration of empirical antibiotics all the time or often, while 80.0% (380/475) of Vietnamese respondents and 54.2% (251/463) of Indonesian respondents answered likewise (p<0.001, Appendix S4). Respondents who gave priority to BC were more likely to answer with “definitely take BC” in the case scenario (OR 4.25, 95% confidence interval [CI] 3.04-5.94, p<0.001, Appendix S6). Example quotes related to the priority of BC were “If other urgent examinations are to be required, BC could be delayed (Vietnamese respondent [barrier])” and “BC should be performed, although the results are often negative. We can't wait for patients not responding to empirical antibiotics before starting BC (Indonesian respondent [enabler])” (Appendix S5).

For peer review only

TDF-social professional role and identity

Theme: Perception about their role to order or initiate an order for BC

Most medical doctors (86.5%, 905/1,046) answered that it is very appropriate or appropriate for them to order BC or initiate an order for BC, while only about half of final-year medical students (49.8%; 115/231) answered likewise (p<0.001). Among medical doctors, 95.8% (207/216) of Thai respondents answered that it is very appropriate or appropriate for them to order BC or

For peer review only - http://bmjopen.bmj.com/site/about/guidelines.xhtml

initiate an order for BC, while 87.0% (368/423) of Vietnamese respondents and 81.1% (330/407) of Indonesia respondents answered likewise ($p<0.001$). The respondents who answered that it is their role to order or initiate an order for BC were more likely to answer with "definitely take BC" in the case scenario (OR 3.36, 95%CI 2.50-4.51, $p<0.001$).

Theme: Level of doctors who can order or initiate an order for BC

More than 75% of Thai respondents answered that all levels of medical doctors (consultants, physicians, residents and interns) can order or initiate an order for BC in their hospitals, while most Indonesian and Vietnamese respondents (87.9%, 870/990) answered that consultants can, but fewer answered that physicians (61.8%, 612/990), residents (59.1%, 585/990) and interns (20.3%, 201/990) can ($p<0.001$). A quarter of Thai respondents (28.7%, 87/303) answered that final-year medical students can order or initiate an order for BC under supervision of attending medical doctors, while Indonesian respondents (2.2%, 11/500) and Vietnamese respondents (0.6%, 3/490) rarely answered likewise ($p<0.001$). None reported that nurses can order or initiate an order for BC.

Theme: perception about their role to draw blood for BC

Most respondents (72.8%, 949/1,303) answered that registered nurses are tasked to draw blood from patients for BC, followed by microbiology laboratory team (36.0%, 469/1,303), specialised blood draw team (27.4%, 357/1,303), residents (25.4%, 331/1,303), physicians (23.5%, 306/1,303), consultants (23.2%, 302/1,303), interns (17.8%, 229/1,303) and final-year medical students (11.6%, 151/1,303). Of respondents who answered that they are tasked to draw blood for BC themselves, 69.1% (248/359) responded that it is very appropriate or appropriate for their

For peer review only - http://bmjopen.bmj.com/site/about/guidelines.xhtml

role to draw blood for BC. Those respondents were more likely to answer with "definitely take BC" in the case scenario (OR 1.94, 95%CI 1.04-3.64, p=0.04).

TDF-belief about consequences

Theme: Perceived that BC is helpful

Most respondents strongly agreed or agreed that BC is helpful in adjusting antibiotics (94.0%, 1,224/1,302), clinical decisions (93.6%, 1,220/1,303), detecting AMR bacterial infections (92.1%, 1,199/1,302), ruling in an infection (90.2%, 1,172/1,299), reducing overuse of antibiotics (87.4%, 1,140/1,304) and reducing patient mortality (79.2%, 1,027/1,297). Most respondents strongly agreed or agreed that accumulative results of BC are helpful in understanding epidemiology of AMR bacterial infections (94.5%, 1,228/1,299). More than half of respondents strongly agreed or agreed that BC is helpful in reducing length of hospital stay (72.3%, 938/1,298) and ruling out an infection (60.5%, 786/1,300).

Respondents who perceived that BC is helpful in clinical decisions (OR 2.96, 95%CI 1.71-5.12, p<0.001), reducing patient mortality (OR 1.61; 95%CI 1.18-2.20, p=0.003), ruling in an infection (OR 1.58, 95%CI 1.04-2.39, p=0.03), reducing length of hospital stay (OR 1.53, 95%CI, 1.14-2.04, p=0.004) or understanding epidemiology of AMR bacterial infections (OR 2.89, 95%CI 1.60-5.19, p<0.001) were more likely to answer with "definitely take BC" in the case scenario. The proportion of respondents who answered that BC is helpful in clinical decisions was highest in Thai (97.7%, 297/304), followed by Indonesia (96.6%, 483/500) and Vietnam (88.2%, 440/499, p<0.001).

For peer review only

For peer review only - http://bmjopen.bmj.com/site/about/guidelines.xhtml

Theme: Perceived that BC is unnecessary

Some respondents strongly agreed or agreed that BC is unnecessary because it is not too late to collect BC later, particularly if patients do not improve after receiving empirical antibiotic treatment (32.7%, 423/1,293), the therapeutic consequence of BC sampling is questionable (18.6%, 238/1,277), antibiotic therapy can be determined based on clinical presentations (17.5%, 228/1,301), results are often delayed (17.0%, 220/1,298) quality of laboratory is questionable (15.3%, 194/1,269), the scientific basis of the guideline on BC is questionable (15.0%, 191/1,277), results are often negative or no growth (11.4%, 148/1,295), and results are often contaminated (11.1%, 143/1,288).

Respondents who perceived that BC is unnecessary because BC is not benefiting the patients (OR 0.37; 95%CI 0.24-0.57, p<0.001), it is not too late to collect BC later, particularly if patients do not improve after receiving empirical antibiotic treatment (OR 0.37; 95%CI 0.27-0.52, p<0.001), BC results are often delayed (OR 0.48, 95%CI 0.33-0.69, p<0.001), quality of laboratory is questionable (OR 0.48; 95%CI 0.33-0.70, p<0.001), antibiotic therapy can be determined based on clinical presentation (OR 0.51, 95%CI 0.36-0.73, p<0.001), a contaminated result often leads to wrong therapeutic approach (OR 0.53; 95%CI 0.30-0.95, p=0.03), BC results are often not interpretable (OR 0.54, 95%CI 0.34-0.87, p=0.01), BC results are often negative or no growth (OR 0.58, 95%CI 0.39-0.88, p=0.01), levels of local antibiotic resistance are low (OR 0.64; 95%CI 0.41-0.98, p=0.04), cultures are often contaminated (OR 0.64, 95%CI 0.42-0.98, p=0.04) and the scientific basis of the guideline on BC is questionable (OR 0.66, 95%CI 0.45-0.98, p=0.04) were less likely to answer with “definitely take BC” in the case

For peer review only

For peer review only - http://bmjopen.bmj.com/site/about/guidelines.xhtml

scenario. The proportion of respondents who answered that BC is not benefitting the patients was not different between countries (5.9%, 76/1,297, p=0.38).

TDF-intention

TDF-intention domain covers a conscious decision to perform or a resolve to act in a certain way, and stability of intentions.[16-18]

Theme: Intention to follow guidelines

Among those who answered that they know of local guidelines, 92.9% (157/169) of Thai respondents answered that they plan to follow local guidelines all the time or often, while 82.0% (283/345) of Vietnamese respondents and 74.1% (172/232) of Indonesian respondents answered likewise ($p<0.001$). Respondents who intended to follow local guidelines were more likely to answer with “definitely take BC” in the case scenario (OR 2.92, 95% CI 1.88-4.53, $p<0.001$).

TDF-knowledge

Theme: Awareness of guidelines

The proportion of respondents who answered that they know of local guidelines for BC sampling was highest in Vietnam (70.7%; 347/491), followed by Thailand (56.3%, 169/300) and Indonesia (48.9%, 240/503, $p<0.001$). The proportion of respondents who answered that they know of international guidelines for BC sampling (47.8%, 596/1,248) was not different between countries (p=0.73). Respondents who answered that they know of local guidelines (OR 2.55, 95%CI 1.93-3.38, $p<0.001$) or international guidelines (OR 1.97, 95%CI 1.50-2.57, $p<0.001$) were more likely to answer with “definitely take BC” in the case scenario.

Theme: Training

For peer review only - http://bmjopen.bmj.com/site/about/guidelines.xhtml

The proportion of respondents who answered that there were no training, lectures, classes or meetings that provide knowledge about local/national/international guidelines for BC sampling in their hospitals was highest in Indonesia (37.8%, 153/407), followed by Thailand (24.9%, 64/257) and Vietnam (12.5%, 52/421, p<0.001). Respondents who answered that there are training, lectures, classes or meetings that provide knowledge about guidelines for BC sampling were more likely to answer with "definitely take BC" in the case scenario (OR 1.68; 95%CI 1.18-2.38, p=0.004).

TDF-social influence

Theme: Norms of BC sampling

Most Thai respondents (78.5%, 233/297) answered that they order BC because they are following local norms all the time or often, while 51.5% (238/462) of Vietnamese respondents and 43.8% (180/411) of Indonesian respondents answered likewise (p<0.001). The respondents who answered that they order BC because they are following local norms were more likely to answer with "definitely take BC" in the case scenario (OR 2.20, 95%CI 1.67-2.90, p<0.001).

Theme: Influences from healthcare workers, patients and family of patients

Most respondents (79.4%) answered that there are very positive or positive influences on BC sampling from consultants, followed by residents (64.5%), doctors (64.6%), heads of department (65.9%), executive levels (50.6%), nurses (47.6%), interns (45.2%), patients (43.0%) and family of patients (31.9%). Some respondents said that there are negative or very negative influence in BC sampling from family of patients (6.8%), nurses (5.2%), patients (4.3%) and executives of the hospital (3.6%). A number of quotes on this theme were noted; including "Negative influence

For peer review only

in the order of BC is cost. Supervisor or the executives (of the hospitals) gave an order to control the cost (Thai respondent [barrier])" and "Sometimes, when the blood puncture fails on the first try, patients and their families refuse to have more blood drawn (Indonesian respondent [barrier])" (Appendix S5).

TDF-reinforcement

Theme: Consequences that discourage BC sampling

Some respondents (32.5%, 300/923) answered that, if they order a BC when it is recommended, there are either negative social consequences (e.g. verbal reprimand or any pressure from supervisors/executives of the hospital as the hospital (may) have to pay for the (extra) cost of BC) or negative material consequences (e.g. a negative score, that doctors are at risk of having to spend extra time and effort to reimburse the cost of BC from any health scheme or insurance, or that doctors are at risk of having to pay for the [extra] cost of BC themselves). The proportion of those who answered likewise was highest in Vietnam (42.2%, 153/363), followed by Thailand (27.0%, 60/222) and Indonesia (25.7%, 87/338). Those who answered that there are negative consequences were less likely to answer with "definitely take BC" in the case scenario (OR 0.48; 95%CI 0.34-0.67, p<0.001). A number of quotes on this theme was noted; including "Warnings are given due to the costly examination, especially for patients insured with the Healthcare and Social Security Agency (Indonesian respondent [barrier])" and "Sometimes, the cost of BC cannot be reimbursed, and the doctor has to pay (Vietnamese respondent [barrier])" (Appendix S5).

TDF-behavioural regulation

For peer review only - http://bmjopen.bmj.com/site/about/guidelines.xhtml

Theme: Regulation of cost reimbursement

Some respondents stated that ‘whether patients have a health scheme or insurance that covers the cost of BC’ (15.0%, 196/1,308) and that ‘whether patients are likely to have a final diagnosis that includes the cost of BC in the package of fee for service’ are their additional reasons for deciding to order BC (11.6%, 152/1,308). Those respondents were not associated with answering with “definitely take BC” in the case scenario (p>0.20, both). However, a number of quotes on this theme were noted; including “The insurance often disapproves of BC examination. It is only approved when patients are admitted to the ICU or HCU [High Care Unit] (Indonesian respondent [barrier])” and “Medical professionals often object to BC due to tiredness [disheartened feeling] and the consequence of reduced reimbursement (Vietnamese respondent [barrier])” (Appendix S5).

For peer review only

Theme: Procedures to support or regulate doctors to order BC

Overall, the most common procedures to support or regulate doctors to order BC in respondents’ hospitals were case reviews (e.g. grand rounds or morning ward rounds, and BC is often mentioned; 30.8%, 326/1,060), followed by standard order forms to remind ordering BC (29.9%, 317/1,060), stewardship programmes and reviewing BC is included in the programmes (19.5%, 207/1,060), posters (15.4%, 163/1,060) and computer systems to remind ordering BC (10.7%, 113/1,060). Respondents who answered that there were case reviews (OR 1.55, 95%CI 1.14-2.13, p=0.006) or stewardship programmes (OR 1.65, 95%CI 1.16-2.34, p=0.005) were more likely to answer with “definitely take BC” in the case scenario

TDF-environmental context and resources

Theme: Perceived cost-effectiveness of BC

Most Vietnamese respondents (85.9%, 407/474) considered that BC is very likely or likely to be cost-effective, while 79.5% (232/292) of Thai respondents and 68.8% (311/452) of Indonesian respondents considered likewise. The respondents who considered that BC is cost-effective were more likely to answer with “definitely take BC” in the case scenario (OR 1.63, 95%CI 1.17-2.26, p<0.001).

Theme: Availability of microbiology laboratories, transport modalities, resources and consumables

Some respondents answered that they could not order BC because microbiology laboratories are not available or not functioning (13.4%, 157/1,174) or consumables (such as BC bottles, needles, syringes, blood collection set, etc.) are not available (12.7%, 150/1,181) all the time or often. Those respondents were not associated with answering with “definitely take BC” in the case scenario (p>0.20 both)

Theme: Out-of-pocket

About a quarter of Indonesian respondents (23.3%, 78/335) answered that patients have to pay for BC using their own money (i.e. out of pocket) all the time or often, while 12.2% (28/230) of Thai participant and 8.3% (34/408) of Vietnamese participant answered likewise (p<0.001). Those respondents were not associated with answering with “definitely take BC” in the case scenario (p=0.29).

For peer review only - http://bmjopen.bmj.com/site/about/guidelines.xhtml

Additional results and the content themes in the domains that were not identified as key domains are described in Appendix S1. We observed that presence of many barriers/enablers was different between countries. However, the presence of those barriers/enablers was not strongly associated with the answer in the case scenario. For example, patients who are already on antibiotics. A quarter of Thai respondents (26.6%, 81/304) answered that they were very likely to still order BC, while only 14.4% (72/501) of Vietnamese respondents and 3.2% (16/503) of Indonesian respondents did ($p<0.001$). Those respondents were not associated with answering with "definitely take BC" in the case scenario ($p=0.13$).

Intervention types and policy options to improve BC sampling practice

We used the links between TDF, COM-B, and BCW, and listed all suggested intervention types and policy options related to very important TDF domains in Indonesia, Thailand and Vietnam (Table 5 and Appendix S7). A range of potential strategies were identified. Some strategies target individual reinforcement, environmental structure and social influence (e.g. providing an example for physicians to aspire to or imitate the BC sampling practice [Intervention type-modelling] and increasing means and reducing barriers to increase capability and opportunity for all levels of doctors to order or initiate an order for BC [Intervention type-enablement]). Some strategies operate at the policy or service provision level (e.g. changing regulation of cost reimbursement [Policy option-fiscal], development or implementation of local guidelines [Policy option-guideline] and establishing rules or principles of BC practice [Policy option-regulation]).

For peer review only - http://bmjopen.bmj.com/site/about/guidelines.xhtml

Table 5. Suggested intervention types and policy options to improve BC sampling practice based on very important TDF domains in Indonesia, Thailand and Vietnam

	COM-B components				
	Psychological capability (TDF: knowledge, and behavioural regulation)	Reflective motivation (TDF: goals, beliefs about consequence, and intention)	Automatic motivation (TDF: reinforcement)	Physical opportunity (TDF: environmental context, and resources)	Social opportunity (TDF: social influence)
**Intervention types** *					
Education	√	√			
Persuasion		√	√		
Incentivisation		√	√		
Coercion		√	√		
Training	√				
Restriction				√	√
Environmental restructuring			√	√	√
Modelling			√		
Enablement	√		√	√	√
**Policy options** *					
Communication/marketing		√	√		
Guidelines	√	√	√	√	√
Fiscal	√	√	√	√	√
Regulation	√	√	√	√	√
Legislation	√	√	√	√	√
Environmental/social planning	√		√	√	√
Service provision	√	√	√	√	√

* Suggested intervention types and policy options were identified using the links between TDF, the components of the COM-B and the BCW.[16-18]

For peer review only

DISCUSSION

Our study shows that barriers and enablers to BC sampling in Southeast Asia are varied and heterogenous. We consider that 'priority of BC [TDF-goals]', 'perception about their role to order or initiate an order for BC [TDF-social professional role and identity]', 'intention to follow guidelines [TDF-intention]', 'norms of BC sampling [TDF-social influence]', 'consequences that discourage BC sampling [TDF-reinforcement]' and 'regulation on cost reimbursement [TDF-behavioural regulation]' are key barriers/enablers. In Thailand,[10] where BC utilisation rate is relatively high compared to Indonesia[9] and Vietnam,[11] the proportions of each enabler being reported by respondents is higher for many domains. For example, the proportion of respondents who gave priority to BC was highest in Thailand at 91.3%. Likewise, the proportions of each barrier being reported by Thai respondents is lower for many domains. For example, the proportion of respondents who answered that there are consequences that discourage BC sampling was highest in Vietnam (42.2%) and the proportion of respondents who answered that patients have to pay for BC using their own money (i.e. out of pocket) was highest in Indonesia (23.3%). To improve diagnostic stewardship practices, all stakeholders will need to consider all suggested intervention types and policy options and develop intervention content based on local context.[16-18]

'Priority to BC [TDF-goals]', 'perception about their role to order or initiate an order for BC [TDF-social professional role and identity]', 'intention to follow guidelines [TDF-intention]' and 'norms of BC sampling [TDF-social influence]' are likely key barriers to BC sampling in both HICs and other LMICs where resources for BC sampling are available to some extent.[8, 12-15]

For peer review only - http://bmjopen.bmj.com/site/about/guidelines.xhtml

To our knowledge, ‘priority of BC [TDF-goals]’, ‘level of doctors who can order or initiate an order for BC [TDF-social professional role and identity]’ and ‘influence from healthcare workers, patients and families of patients [TDF-social influence]’ have never been evaluated in LMICs.[8, 12-15] Those are important barriers/enablers. ‘Priority of BC’ has the highest OR for the association with “definitely take BC” in the case scenario in our study (OR 4.25). The importance of ‘priority of BC’ was previously reported from HICs.[13] In addition, in many hospitals in both HICs and LMICs, final-year medical students and interns are responsible for most BC ordering and acquisition[31] and influences from other parties can discourage BC sampling.

Remarkably, the cost of BC seems to have influence on executive level doctors, patients, families of patients, medical doctors, and those who set regulations on cost reimbursement of BC. This is shown by many quotes related to the cost of BC in the theme ‘influences from healthcare workers, patients and family of patients [TDF-social influence]’, ‘consequences that discourage BC sampling [TDF-reinforcement]’, ‘perceived cost-effectiveness of BC [TDF-environmental context and resources]’ and ‘regulation on cost reimbursement [TDF-behavioural regulation]’ (Appendix S5).

It is worth noting that the quotes related to the cost-related barriers are more common in Indonesian and Vietnamese respondents than in Thai respondents. Nonetheless, ‘no priority of BC’, ‘lack of role to order BC’, ‘perceived that BC is unnecessary’, ‘no local guidelines for BC’ and ‘no intention to follow local guidelines’ are examples of many barriers that are not directly related to cost.

For peer review only - http://bmjopen.bmj.com/site/about/guidelines.xhtml

To overcome cost-related barriers, multi-facet interventions based on local context should be considered and implemented. For example, the interventions may include providing clear posters emphasising local guidelines for BC sampling over wide areas in hospitals [Intervention type-environmental restructuring]. This intervention type is aimed to increase social opportunity, physical opportunity and automatic motivation for medical doctors to adopt and practice the local guidelines for BC sampling (Appendix S7).[16-18] This intervention could reduce the barrier '(negative) influences from healthcare workers, patients and family of patients [TDF-social influence]' and 'perceived cost-effectiveness of BC [TDF-environmental context and resources]' if the importance and benefit of BC sampling are clearly present on the posters endorsed by the local hospitals and national authorities. Repeatedly announcing to all levels of healthcare workers that negative consequences that discourage BC sampling per local guidelines will not be tolerated [Intervention type-enablement] could be considered and implemented to reduce the barrier '(negative) consequences that discourage BC sampling [TDF-reinforcement]'. Changing regulation of cost reimbursement and finding financial support for BC sampling per local guidelines [Policy option-fiscal] could be considered and implemented to reduce the barrier 'regulation on cost reimbursement [TDF-behavioural regulation]'. Most importantly, multi-facet interventions are recommended to be systematically designed based on barriers and enablers locally identified and based on local context.[16-18]

Fear of 'blood stealing' or 'blood selling' is reported as a barrier to blood specimen collection in many countries in sub-Saharan Africa; including Kenya, Zambia, Mozambique, The Gambia, Tanzania and Uganda.[32] We observed fears of pain, needles, drawing a lot of blood, anaemia,

For peer review only

For peer review only - http://bmjopen.bmj.com/site/about/guidelines.xhtml

blood-transmitted diseases, etc. (Appendix S5), but did not observe fear of ‘blood stealing’ or ‘blood selling’. Emotional barriers to BC sampling are likely different depending on local regions.

This study has several limitations. First, we used a convenience sample of hospitals and practitioners, which might have led to selection bias. The sampling frame size and the response rate are unknown. It is possible that those who did not receive the invitation and those received the invitation but did not respond to the survey had different frequencies of or different barriers/enablers to BC sampling than those who participated in the study. This limited our ability to draw definite conclusions on the contemporary situation on barriers/enablers to BC sampling in each country and in Southeast Asia. Second, the survey could not reach the target sample size in Thailand despite substantial efforts. The study might not have enough power to evaluate all barriers and enablers adequately. Third, the findings may not be generalisable to all LMICs because barriers and enablers to BC sampling can be varied and local evaluations are needed.

In conclusion, this comprehensive analysis using TDF gives information across the entire spectrum of behavioural influences of BC sampling. These results can help local healthcare providers and policy makers to develop and implement interventions aiming to improve diagnostic stewardship practices.

For peer review only

Competing interests

The authors declare no competing interests.

Data availability statement

Data are available upon reasonable request. All authors recognise the value of sharing individual level data. We aim to ensure that data generated from all our research are collected, curated, managed and shared in a way that maximises their benefit. Data underlying this publication are available upon request to the Mahidol Oxford Tropical Medicine Research Uni Data Access Committee at https://www.tropmedres.ac/units/moru-bangkok/bioethics-engagement/data-sharing.

Acknowledgements

The authors thank the respondents and staff at all the study hospitals and Mahidol-Oxford Tropical Medicine Research Unit. The authors thank Surveillance and Epidemiology of Drug-resistant Infections Consortium (SEDRIC) for the support and comments on the study. We thank Le Nguyen Minh Hoa, Vu Minh Duy, Dam Thi Hong Hanh and Hoang Bao Long for support in data collection.

Contributors

F.L., L.A. and D.L. designed and supervised the study. P.S., K.S.A., R.L., V.T.L.H., H.R.v.D. and R.L.H. participated in project design and facilitated data collection. A.T., L.W.A.R., R.B., E.J.N., D.U.N., S.K., W.S., P.C., W.P., N.H.Y., P.N.T., L.M.Q., V.H.V., C.M.D., V.T.H.D.E. and E.H. facilitated data collection. P.S. analysed the data and wrote the first draft of the

For peer review only

For peer review only - http://bmjopen.bmj.com/site/about/guidelines.xhtml

manuscript. All authors contributed to the writing or revision of the manuscript. P.S. and D.L. verified the data.

Ethics approval

The study was approved by the Oxford University Tropical Research Ethics Committee (OXTREC545-21) and local ethical committees at Iskak Tulungagung Hospital (070/7303/407.206/2021), Prof. Dr. R.D. Kandou Hospital (156/EC/KEPK-KANDOU/IX/2021), Pasar Minggu Hospital (EOCRU/RCH.216/10.2021/1145) in Indonesia, The National Hospital for Tropical Diseases (14HDDD/NDTU) in Vietnam, Faculty of Tropical Medicine, Mahidol University (TMEC21-069), Sunpasitthiprasong Hospital (065/64S) and Chiangrai Prachanukroh Hospital (CR 0032.102/EC023) in Thailand. Electronic informed consent was obtained from study respondents prior to participation.

Funding

This research was funded by the Wellcome Trust (220557/Z/20/Z). The views expressed are those of the author(s) and not necessarily those of the funders. For the purpose of Open Access, the author has applied a CC BY public copyright licence to any Author Accepted Manuscript version arising from this submission.

For peer review only

References

1. Evans L, Rhodes A, Alhazzani W, Antonelli M, Coopersmith CM, French C, et al. Surviving sepsis campaign: international guidelines for management of sepsis and septic shock 2021. Intensive Care Med. 2021;47(11):1181-247.

2. Schuts EC, Hulscher M, Mouton JW, Verduin CM, Stuart J, Overdiek H, et al. Current evidence on hospital antimicrobial stewardship objectives: a systematic review and meta-analysis. Lancet Infect Dis. 2016;16(7):847-56.

3. WHO. Diagnostic stewardship: a guide to implementation in antimicrobial resistance surveillance sites. 2016. Available from: https://www.who.int/publications/i/item/WHO-DGO-AMR-2016.3

4. Singer M, Deutschman CS, Seymour CW, Shankar-Hari M, Annane D, Bauer M, et al. The Third International Consensus Definitions for Sepsis and Septic Shock (Sepsis-3). JAMA. 2016;315(8):801-10.

5. Warren BG, Yarrington ME, Polage CR, Anderson DJ, Moehring RW. Evaluation of hospital blood culture utilization rates to identify opportunities for diagnostic stewardship. Infect Control Hosp Epidemiol. 2022:1-6.

6. Chen AI, Bilker WB, Hamilton KW, O'Donnell JA, Nachamkin I. Blood culture utilization at an academic hospital: Addressing a gap in benchmarking. Infect Control Hosp Epidemiol. 2018;39(11):1353-9.

7. Cassini A, Hogberg LD, Plachouras D, Quattrocchi A, Hoxha A, Simonsen GS, et al. Attributable deaths and disability-adjusted life-years caused by infections with antibiotic-resistant bacteria in the EU and the European Economic Area in 2015: a population-level modelling analysis. Lancet Infect Dis. 2019;19(1):56-66.

For peer review only

For peer review only - http://bmjopen.bmj.com/site/about/guidelines.xhtml

8. WHO. Central Asian and Eastern European Surveillance of Antimicrobial Resistance. Annual Report 2018. 2018. Available from: https://www.euro.who.int/__data/assets/pdf_file/0007/386161/52238-WHO-CAESAR-AR-2018_low_V11_web.pdf

9. Sinto R, Lie KC, Setiati S, Suwarto S, Nelwan EJ, Djumaryo DH, et al. Blood culture utilization and epidemiology of antimicrobial-resistant bloodstream infections before and during the COVID-19 pandemic in the Indonesian national referral hospital. Antimicrob Resist Infect Control. 2022;11(1):73.

10. Teerawattanasook N, Tauran PM, Teparrukkul P, Wuthiekanun V, Dance DAB, Arif M, et al. Capacity and Utilization of Blood Culture in Two Referral Hospitals in Indonesia and Thailand. Am J Trop Med Hyg. 2017;97(4):1257-61.

11. Takeshita N, Anh NQ, Phuong DM, Thanh DV, Thuy PP, Huong MTL, et al. Assessment of Bacteremia in a Large Tertiary Care Hospital in Northern Vietnam: a Single-Center Retrospective Surveillance Study. Jpn J Infect Dis. 2019;72(2):118-20.

12. She RC, Alrabaa S, Lee SH, Norvell M, Wilson A, Petti CA. Survey of physicians' perspectives and knowledge about diagnostic tests for bloodstream infections. PLoS One. 2015;10(3):e0121493.

13. Raupach-Rosin H, Duddeck A, Gehrlich M, Helmke C, Huebner J, Pletz MW, et al. Deficits in knowledge, attitude, and practice towards blood culture sampling: results of a nationwide mixed-methods study among inpatient care physicians in Germany. Infection. 2017;45(4):433-41.

For peer review only

For peer review only - http://bmjopen.bmj.com/site/about/guidelines.xhtml

14. Ojide CK, Onwuezobe IA, Asuquo EE, Obiagwu CS. Knowledge, attitude and practice of blood culture: A cross sectional study among medical doctors in a nigerian tertiary hospital. African Journal of Clinical and Experimental Microbiology. 2013;14(3):174-9.

15. Chew KS, Mohd Hashairi F, Jusoh AF, Aziz AA, Nik Hisamuddin NA, Siti Asma H. Knowledge of good blood culture sampling practice among healthcare staffs in an emergency department - Are we getting it right? Medical Journal of Malaysia. 2013;68(4):323-5.

16. Michie S, van Stralen MM, West R. The behaviour change wheel: a new method for characterising and designing behaviour change interventions. Implement Sci. 2011;6:42.

17. Cane J, O'Connor D, Michie S. Validation of the theoretical domains framework for use in behaviour change and implementation research. Implement Sci. 2012;7:37.

18. Atkins L, Francis J, Islam R, O'Connor D, Patey A, Ivers N, et al. A guide to using the Theoretical Domains Framework of behaviour change to investigate implementation problems. Implementation Science. 2017;12(1):77.

19. McDonagh LK, Saunders JM, Cassell J, Curtis T, Bastaki H, Hartney T, et al. Application of the COM-B model to barriers and facilitators to chlamydia testing in general practice for young people and primary care practitioners: a systematic review. Implement Sci. 2018;13(1):130.

20. Sargent L, McCullough A, Del Mar C, Lowe J. Using theory to explore facilitators and barriers to delayed prescribing in Australia: a qualitative study using the Theoretical Domains Framework and the Behaviour Change Wheel. BMC Fam Pract. 2017;18(1):20.

21. Lohiniva AL, Heweidy I, Girgis S, Abouelata O, Ackley C, Samir S, et al. Developing a theory-based behavior change intervention to improve the prescription of surgical prophylaxis. Int J Clin Pharm. 2021.

For peer review only - http://bmjopen.bmj.com/site/about/guidelines.xhtml

637 22. Smith JD, Corace KM, MacDonald TK, Fabrigar LR, Saedi A, Chaplin A, et al. Application of the Theoretical Domains Framework to identify factors that influence hand hygiene compliance in long-term care. J Hosp Infect. 2019;101(4):393-8.

640 23. Mazza D, Chapman A, Michie S. Barriers to the implementation of preconception care guidelines as perceived by general practitioners: a qualitative study. BMC Health Serv Res. 2013;13:36.

643 24. Suntornsut P, Wongsuwan N, Malasit M, Kitphati R, Michie S, Peacock SJ, et al. Barriers and Recommended Interventions to Prevent Melioidosis in Northeast Thailand: A Focus Group Study Using the Behaviour Change Wheel. PLoS Negl Trop Dis. 2016;10(7):e0004823.

646 25. Wong E, Mavondo F, Horvat L, McKinlay L, Fisher J. Healthcare professionals' perspective on delivering personalised and holistic care: using the Theoretical Domains Framework. BMC Health Serv Res. 2022;22(1):281.

649 26. Huijg JM, Gebhardt WA, Crone MR, Dusseldorp E, Presseau J. Discriminant content validity of a theoretical domains framework questionnaire for use in implementation research. Implementation Science. 2014;9(1):11.

652 27. Eng J. Sample size estimation: how many individuals should be studied? Radiology. 2003;227(2):309-13.

654 28. Patey AM, Islam R, Francis JJ, Bryson GL, Grimshaw JM, Canada PPT. Anesthesiologists' and surgeons' perceptions about routine pre-operative testing in low-risk patients: application of the Theoretical Domains Framework (TDF) to identify factors that influence physicians' decisions to order pre-operative tests. Implement Sci. 2012;7:52.

658 29. Yaddanapudi LN. The American Statistical Association statement on P-values explained. J Anaesthesiol Clin Pharmacol. 2016;32(4):421-3.

For peer review only

30. Sterne JA, Davey Smith G. Sifting the evidence-what's wrong with significance tests? BMJ. 2001;322(7280):226-31.

31. Parada JP, Schwartz DN, Schiff GD, Weiss KB. Effects of type and level of training on variation in physician knowledge in the use and acquisition of blood cultures: A cross sectional survey. BMC Infectious Diseases. 2005;5.

32. Peeters Grietens K, Ribera JM, Erhart A, Hoibak S, Ravinetto RM, Gryseels C, et al. Doctors and vampires in sub-Saharan Africa: ethical challenges in clinical trial research. Am J Trop Med Hyg. 2014;91(2):213-5.

For peer review only

For peer review only - http://bmjopen.bmj.com/site/about/guidelines.xhtml

Appendices: Barriers and enablers to blood culture sampling in Indonesia, Thailand and Vietnam: a Theoretical Domains Framework (TDF)-based survey

Appendix S1: Supplementary Text

Supplementary Methods

The healthcare systems in SEA are highly diverse.[1] In 2020, Indonesia (GDP per capita: 3,869.6 US$) and Vietnam (GDP per capita: 2,785.7 US$) were a lower-middle-income country and Thailand (GDP per capita:7,186.9 US$) was an upper-middle-income country in SEA.[2] Indonesia has a decentralised public healthcare system, in which provincial or district-level governments have the authority over most public hospitals, and a substantial private health sector. To achieve the goal of universal healthcare coverage (UHC), in 2014 the Government introduced national health insurance (Jaminan Kesehatan Nasional), which had reached 84% of the population by 2021. Thailand achieved the status of UHC in 2002 in terms of insurance entitlement, when the gross national income per capita was 1,900 US$.[3] It is shown that UHC in Thailand can improve quality of care without undermining the efficiency and equity of the policy.[4] Vietnam has implemented social health insurance (SHI) since 1992, and SHI had a role as a financial mechanism towards achieving UHC,[5] which had reached 82% of the population in 2018. The benefit package of universal SHI in Vietnam is considered generous, particularly regarding the drugs subsidized.[5] However, out-of-pocket payments are still high.[5, 6] In 2019, percentages of out-of-pocket expenditure among all health expenditure were 35%, 9% and 43% in Indonesia, Thailand and Vietnam, respectively.[7]

Analysis

We explored the agreement between two themes of the TDF domain reinforcement. The degree of agreement between responses to the questions for barriers/enablers was estimated using the Kappa index. This describes the level of association, both positive and negative, beyond that caused by chance, as follows: 0.00–0.20, slight; 0.21–0.40, fair; 0.41–0.60, moderate; 0.61–0.80, substantial; 0.81–1.00, high.

For peer review only - http://bmjopen.bmj.com/site/about/guidelines.xhtml

Additional analysis

We explored whether the answers of respondents who completed the survey were different from the answers of respondents who did not complete the survey. We compared the answers to the case scenario between those who completed the questionnaire and those who answered the case scenario (Question 1-3 in the questionnaire) but did not complete the questionnaire. Logistic regression model with random effects for countries was used for the analysis.

Supplementary Results

Additional results and the content themes in the domains that were identified as key domains are described in further detail in the sections below.

TDF-Reinforcement

Theme: Consequences that encourage BC sampling. Some respondents (23.7%, 294/1,243) answered that there are either positive social (e.g. praise) or positive material (e.g. a positive score) consequences if they order a BC when it is recommended. Those respondents were less likely to answer with “definitely take BC” in the case scenario (OR 0.53; 95%CI 0.37-0.74, p<0.001). We explored and found that respondents who answered that there are positive consequences that encourage BC sampling when recommended also answered that there are negative consequences that discourage BC sampling when recommended with moderate agreement beyond that expected by chance (Kappa value 0.46, p<0.001).

We also evaluated whether they are negative consequences if practitioners do not order a BC when it is recommended. Some respondents (37.7%, 464/1,230) answered that there are either negative social (e.g. verbal reprimand) or negative material (e.g. a negative score) consequences if they do not order a BC when it is recommended. Those respondents were not associated with answering with “definitely order BC” in the case scenario (p=0.42).

TDF-Emotion

Theme: Fear or anxiety of healthcare providers and *Fear or anxiety of patients or family of patients.* Some respondents (7.1%, 93/1,308) stated that there are emotional factors associated

For peer review only - http://bmjopen.bmj.com/site/about/guidelines.xhtml

with ordering BC. Those include fear or anxiety related to pain, needles, blood-borne diseases, high volume of blood being drawn, anaemia, etc. Those respondents were not associated with answering "definitely take BC" in the case scenario (p=0.82). Numerous quotes on this theme as a barrier were noted (Appendix S5).

TDF-Optimism

Theme: Optimism about the BC sampling and the laboratory. Most (80.5%, 1,034/1,285) respondents answered that they are strongly optimistic or optimistic that a BC will be sampled and processed in the laboratory appropriately if they order a BC. Respondents who were strongly optimistic or optimistic about the laboratory were more likely to answer with "definitely take BC" in the case scenario (OR 1.78, 95%CI 1.29-2.46, p<0.001). Most of the Thai respondents (88.3%, 263/298) are optimistic about the BC sampling and the laboratory, while 82.4% (400/487) of Indonesian respondents and 74.2% (368/496) of Vietnam respondents are (p<0.001).

TDF-Skills

Theme: Skills in drawing blood for BC. Among respondents whom were tasked to draw blood from patients for BC in their hospitals, 44.1% (143/324) answered that their skill of drawing blood from patients for BC is very good or good, 44.8% (145/324) fair, and 11.1% (36/324) poor or very poor. Respondents who answered that they have very good or good skill in drawing blood for BC was more likely to answer with "definitely take BC" in the case scenario (OR 1.74; 95%CI 1.02-2.07, p=0.04).

TDF-Memory, attention and decision processes

Theme: Patients who are already on antibiotics or have anemia. Some respondents (10.2%, 131/1,287) stated that they will definite or likely not order BC when patients are already on antibiotics even if BC is recommended. A quarter of Thai respondents (26.6%, 81/304) answered that they were very likely to still order BC, while 14.4% (72/501) of Vietnamese respondents and 3.2% (16/503) did (p<0.001, Appendix S6). Those respondents were not associated with answering with "definitely take BC" in the case scenario (p=0.13).

For peer review only - http://bmjopen.bmj.com/site/about/guidelines.xhtml

Some respondents (22.3%, 280/1,258) answered that they will definite or likely not order BC when patients have anemia even if BC is recommended. Those respondents were not associated with answering with "definitely take BC" in the case scenario (p=0.55).

Theme: Clinical presentations for deciding to order BC. Among respondents who responded that they know of local guidelines, some stated that patients with no clinical improvement after receiving empirical antibiotics (36.2%, 274/756), presenting with fever of unknown origin (30.6%, 231/756), suspected of hospital-acquired infection (30.8%, 233/756), presenting with chronic fever (28.6%, 216/756) or suspected of infection caused by antimicrobial-resistant organisms (28.6%, 216/756) are their additional reasons to order BC.

TDF-Belief about capabilities

Theme: Belief in their own capability to draw blood. Most respondents (73.9%, 244/358) answered that they are strongly confident or confident that they can draw BC successfully. Those respondents were not associated with answering with "definitely take BC" in the case scenario (p=0.36). Most respondents (74.8%, 246/329) also answered that they are strongly confident or confident that they can draw BC appropriately using aseptic technique. Those respondents were not associated with answering with "definitely take BC" in the case scenario (p=0.11).

Theme: Belief in capability of those who are tasked to draw blood. Most respondents (88.5%, 1,151/1,300) answered that they are strongly confident or confident that those who are tasked to draw BC can draw BC successfully. Those respondents were not associated with answering with "definitely take BC" in the case scenario (p=0.13). Most respondents (76.7%, 996/1,298) also answered that they are strongly confident or confident that those who are tasked to draw BC can draw BC appropriately using aseptic technique. Those respondents were not associated with answering with "definitely take BC" in the case scenario (p=0.23).

Additional analysis

We explored whether there was any evidence showing a difference between respondents who completed and did not complete the survey. Of 2,095 respondents who agreed to participate the online survey, 1,308 (62.4%) completed the questionnaire, 256 (12.2%) answered the question

For peer review only

about the case scenario (Question 1-3) but did not complete the questionnaire, and 531 (25.3%) did not answer up to the question about the case scenario. The proportion of patients who answered that they would definitely take BC for the case scenario was not different between those who completed the questionnaire (52.1%; 682/1,308) and those who answered the question about the case scenario but did not complete the questionnaire (51.2%; 131/256) (p=0.08).

For peer review only

Appendix S2. Theoretical Domains Framework: Definitions and examples

TDF domain and definition	Examples related to blood culture (BC) sampling
**TDF-1 Knowledge:** awareness of the existence of something	In the context of this study, knowledge of the condition/scientific rationale could relate to their knowledge of:<br>• when and whom BC should be sampled<br>• local and international guidelines for BC sampling<br><br>Knowledge may be both correct and incorrect
**TDF-2 Skills:** ability or proficiency acquired through practice	In the context of this study, skills/competence include skill of participant to draw blood for BC sample collection.<br><br>Skills may be both present and absent
**TDF-3 Social professional role and identity:** a coherent set of behaviours and displayed personal qualities of an individual in a social or work setting	In the context of this study, professional role may relate to the extent that healthcare professionals feel that ordering or initiating an order for BC are part of their professional role or their job description.<br><br>Personal identity may relate to how a participant views their role of<br>• ordering or initiating an order for BC<br>• drawing blood for BC
**TDF-4 Beliefs about capabilities:** acceptance of the truth/reality about or validity of an ability, talent or facility that a person can put to constructive use	In the context of this study, beliefs about capabilities relates to the judgments on medical doctor/final-year medical student's ability to:<br>• draw blood successfully<br>• draw blood appropriately<br><br>As BC may be ordered by respondents but collected by other professionals, beliefs about capabilities also include their judgments on the ability of persons who are tasked to draw blood<br><br>"Successfully" means obtaining blood, and "Appropriately" means that general guidelines for BC specimen collection such as aseptic technique are followed.
**TDF-5 Optimism:** confidence that things will happen for the best or that desired goals will be attained	In the context of this study, optimism related to their judgment regarding that a BC will be sampled and processed in the laboratory appropriately if they order a BC.

For peer review only - http://bmjopen.bmj.com/site/about/guidelines.xhtml

TDF domain and definition	Examples related to blood culture (BC) sampling
	This includes optimism and pessimism.
**TDF-6 Beliefs about consequences*:*** acceptance of the truth/reality about or validity of outcomes of a behaviour in a given situation	In the context of this study, beliefs about their judgments on:<br>• the purpose, value, and effectiveness of BC<br>• negative/positive outcomes of BC
**TDF-7 Reinforcement:** increasing the probability of a response by arranging a dependent relationship, or contingency, between the response and a given stimulus	In the context of this study, reinforcements relate to their judgments on:<br>• receiving an incentive or reward (these can be social [e.g. praise] or material [e.g. a positive score]) for ordering a BC when recommended<br>• receiving any negative consequences (these can be social [e.g. verbal reprimand or that you/doctors are at risk of being scrutinized] or material [e.g. a negative score]) for not ordering BC when recommended<br><br>As feedbacks could discourage the behavior, reinforcement also include judgements on:<br>• receiving any negative consequences for ordering BC when recommended
**TDF-8 Intentions:** conscious decision to perform a behaviour or a resolve to act in a certain way	In the context of this study, intentions relate to the statements on their intention to order BC.
**TDF-9 Goals:** mental representation of outcomes or end states that an individual wants to achieve	In the context of this study, goals relate to the statements on:<br>• the goals they wish to collect BC prior to giving empirical antibiotics<br>• competing goals (goals that might conflict with BC collection; e.g. giving empirical antibiotics)
**TDF-10 Memory, attention and decision processes:** ability to retain information, focus selectively on aspects of the environment and choose between two or more alternatives	In the context of this study, memory, attention and decision processes relate the statements on how they decide whether to order or not order BC
**TDF-11 Environmental context and resources:** any circumstances of a person's situation or environment that discourages or encourages the development of skills and abilities, independence, social competence, and adaptive behaviour	In the context of this study, environmental context and resources relates to their perceptions of the:<br>• Availability of consumables such as bottles, needles, syringes, blood collection set, etc.<br>• Availability of microbiology laboratories<br>• Financial resources, whether patients have to pay out-of-pocket<br>• Cost-effectiveness of BC

For peer review only - http://bmjopen.bmj.com/site/about/guidelines.xhtml

TDF domain and definition	Examples related to blood culture (BC) sampling
**TDF-12 Social influences:** interpersonal processes that can cause an individual to change their thoughts, feeling or behaviours.	In the context of this study, social influences relate to the statements expressing the influence of others on attending BC. Including:<br>• norms<br>• influences from nurses, other medical doctors, consultants, head of department, executive of the hospitals, patients and family of patients<br><br>"Norms" mean usual practice that are typical of or accepted within their hospital.
**TDF-13 Emotion:** a complex reaction pattern, involving experiential, behavioural and physiological elements, by which the individual attempts to deal with a personally significant matter or event	In the context of this study, emotions relate to the statements of expressing their emotional reaction/state relating to order and sample for BC<br><br>Any logical reasons or social influence which are stated as "fear of" are categorized as "Memory, attention and decision processes" or "Social influence" as appropriate.
**TDF-14 Behavioural regulation:** anything aimed at managing or changing objectively observed or measured actions	In the context of this study, behavioural regulation relates to the statements about managements or steps taken to<br>• order BC<br>• adopt local/national/international guidelines for BC sampling

For peer review only - http://bmjopen.bmj.com/site/about/guidelines.xhtml

Appendix S3. TDF-based questionnaire

Online research participant information sheet and electronic consent form

You are invited to participate in a web-based online survey on **"Barriers and facilitators to ordering blood culture samples in Indonesia, Thailand and Viet Nam".** This is a research project being conducted under the collaboration between Eijkman Oxford Clinical Research Unit (EOCRU), **Indonesia,** and Mahidol Oxford Tropical Medicine Research Unit (MORU), Faculty of Tropical Medicine, Mahidol University, **Thailand**, Oxford University Clinical Research Unit (OUCRU), **Viet Nam,** Centre for Behaviour Change, University College London, **United Kingdom**.

PROPOSE: This study aim to identify barrier and facilitators to the adoption of blood culture sampling recommendations in Indonesia, Thailand and Viet Nam

PARTICIPATION: The participants include 1,500 medical doctors and final-year medical students in Indonesia, Thailand and Viet Nam (500 participants per country). The survey is voluntary. You may refuse to take part in the research or exit the survey at any time without penalty. You are free to decline to answer any particular question you do not wish to answer for any reason.

PROCEDURE: You may have received an invitation from clinical directors, head of final-year medical student, or head of recently graduated medical doctors to do this online survey. You may also receive two email reminders about the invitation. We also ask final-year medical students and medical doctors in those hospitals to share the invitation to the survey to any final-year medical students and medical doctors in the country using their networks such as Facebook, Line and WhatsApp application.

In this survey, we will ask whether you know of any local and international guidelines on when to perform blood culture sampling, whether you would perform blood culture sampling for the constructed case scenario, and why you do or do not perform blood culture sampling. It should take approximately 30 – 40 minutes to complete.

All study data will be entered on a Qualtrics. The participants will be identified by a unique study specific number and/or code in any database. We will ask for your email account or telephone number in order to provide you an electronic gift. You may refuse to providing your email account or telephone number and to receiving an electronic gift. The name and any other identifying detail will NOT be included in any study data electronic file.

BENEFITS: You will receive no direct benefits from participating in this research study. However, your responses may help us learn more about what are barriers and facilitators of doctors to order and collect blood culture samples per local, national or international recommendations in different countries. The questionnaire focuses only on when and why blood culture is sampled. Participants will receive a gift or cash (about $4 USD in value) for completing the questionnaire. Participants could receive the gift electronically if email account or telephone number is provided.

RISKS: There is the risk that you may find some of the questions to be sensitive, and that some questions may cause emotional discomfort. Nonetheless, the possible risks or discomforts of the study are minimal. If you feel uncomfortable or distressed at any time during this survey, you should feel free to terminate participation. You are free to decline to answer any particular question you do not wish to answer for any reason. The study team does not expect any risks for participants beyond the minimal risks described above regarding confidentiality surrounding sensitive comments that might arise when participating in the qualitative interviews.

WITHDRAWAL: The survey is voluntary. You can withdraw from the study without penalty at any time and you are free to decline to answer any particular question you do not wish to answer for any reason with no obligation to give the reason for withdrawal.

For peer review only - http://bmjopen.bmj.com/site/about/guidelines.xhtml

CONFIDENTIALITY: Although we will collect your identifying information such as your medical license number (student identification no if you are a medical student), email address and telephone number, your identifying information are needed for compensation and your identifying information will be known only to the researchers performing this study or to specific groups for auditing purposes (if requested). These groups are government institutions or organisations authorised to conduct audits such as the ethics committee. Only summary results will be published and anonymous information will be put in open-access scientific database. No one will be able to identify you or your answers, and no one will know whether you participated in the study.

ETHICAL: The study protocol, informed consent form, participant information sheet and any proposed advertising material will be submitted to OxTREC, the ethics Committee of the Faculty of Tropical Medicine, Mahidol University, Thailand and (FTMEC), and local ethics committees for written approval.

CONTACT: If you have questions at any time about the study or the procedures, you may contact Dr Ralalicia Limato (rlimato@eocru.org) in Indonesia, Pornpan Suntornsut (pornpan@tropmedres.ac) in Thailand, and Dr Vu Thi Lan Huong (huongvtl@oucru.org) in Viet Nam.

DATA PROTECTION: The University of Oxford is responsible for ensuring the safe and proper use of any personal information you provide, solely for research purposes.

DATA SHARING: Data collected for this study will be de-identified and may be shared with other groups of researchers in accordance with the current MORU Data Sharing Policy. All applications will be carefully reviewed by the MORU Data Access Committee before granting any approvals to access data. All researchers accessing the data need to adhere to a set of terms and conditions that aim to protect the interests of research participants and other relevant stakeholders.

INTERNET AND DEVICE REQUIREMENT: This online questionnaire requires good internet connection and relatively up-to-date devices. Mobile devices with small screens may not show the questions clearly. If your devices are relatively out-of-date or with small screens, we recommend you to use a desktop computer at a place with good internet connection. If you have a problem with the online questionnaire, you may ask for the word file (.doc) or the paper questionnaire by contacting Dr Ralalicia Limato (rlimato@eocru.org) in Indonesia, Pornpan Suntornsut (pornpan@tropmedres.ac) in Thailand, and Dr Vu Thi Lan Huong (huongvtl@oucru.org) in Viet Nam.

ELECTRONIC CONSENT: Please select your choice below. You may print a copy of this consent form for your records. Clicking on the "Agree" button indicates that I agree to participate in the research study. I have read the above information and I am participating voluntarily.

○ Agree
○ Disagree

EXPLANATION: The questionnaire may contain ○ for radio button (can take only one answer) ☐ for multiple choices (can take more than one answer)) and open text answer as well. Please indicate your level of opinion and mark in the button or box of your answer.

Q1-1. **At which type of hospital are you currently working?** If you are currently working at more than one hospital, select where you are currently spending most time. (please select the most relevant answer)

○ Government hospital (including National hospital, Provincial hospital, District hospital)
○ Private hospital
○ University hospital
○ I do not want to answer

For peer review only - http://bmjopen.bmj.com/site/about/guidelines.xhtml

○ Other: ……………
Q1-2. **What is your Medical license number or student ID number?** This is to confirm that you are a medical doctor or a final-year medical student in Indonesia, Thailand or Viet Nam. If you are not a medical doctor or a final-year medical student in Indonesia, Thailand or Viet Nam, you should not participate in this questionnaire. Your identifying information will be known only to the researchers. No one will be able to identify you or your answers, and no one will know whether you participated in the study.<br>…………………………………………………………………
Q1-3. **As an introduction to the topic blood culture sampling, we present a case scenario to you. We would like to know if you consider taking blood culture samples in your everyday clinical practice and your current hospital setting.**<br><br>**If you are currently working at more than one hospital, please consider the hospital you are spending most time as your current hospital setting.**<br><br>**Case scenario.** "A 72-year-old woman who was brought to the emergency department of your hospital by her daughter when she noticed the patient was more confused than her baseline and was found to have a high fever and fast breathing. She had an auscultatory finding compatible with pneumonia. It is decided that this patient will be admitted to your hospital."<br><br>If you have an authority to take a blood culture, would you take blood culture sample(s) in this case on admission?<br><br>○ Definitely (>95-100% of the time)<br>○ Likely (75-95% of the time)<br>○ Maybe (25-74% of the time)<br>○ Unlikely (5-24% of the time)<br>○ Rarely (ranging from never <5% of the time)<br>○ I do not know<br>○ I do not want to answer
Q1-4. Do you know of any recommendation(s) or guideline(s) for blood culture sampling being used in your hospital?<br><br>○ Yes<br>○ No, my hospital does not use any recommendations or guidelines for blood culture sampling (go to Q1-8)<br>○ I do not know if my hospital uses any recommendations or guidelines. (go to Q1-8)<br>○ I do not want to answer (go to Q1-8)
(Page break)
Q1-5. **Based on your understanding**, do any following statement(s) represent the recommendation(s) or guideline(s) for blood culture sampling being used in your hospital? (you can select more than one answer)<br><br>☐ Recommend blood culture sampling in all patients presenting with SIRS (Systemic inflammatory Response Syndrome [SIRS] is defined as having at least two of the following criteria: fever or hypothermia, tachycardia, tachypnea, and leukocytosis or leucopenia)<br>☐ Recommend blood culture sampling in all patients presenting with sepsis ('sepsis' here is defined as an acute change in total Sequential Organ Failure Assessment [SOFA] score ≥2 points consequent to the infection based on the most recent definition of sepsis [Sepsis-3 criteria])<br>☐ Recommend blood culture sampling in all patients presenting with septic shock<br>☐ Recommend blood culture sampling in all patients starting parenteral antibiotic treatment<br>☐ Recommend blood culture sampling in all patients with no clinical improvement after receiving empirical antibiotics

For peer review only

☐ Recommend blood culture sampling in all patients presenting with infection and having underlying diseases
☐ Recommend blood culture sampling in all patients with chronic fever
☐ Recommend blood culture sampling in all patients with fever of unknown origins
☐ Recommend blood culture sampling in all patients suspected of infections caused by atypical organisms
☐ Recommend blood culture sampling in all patients suspected of infections caused by antimicrobial-resistant organisms
☐ Recommend blood culture sampling in all patients suspected of infections caused by multiple-drug-resistant organisms
☐ Recommend blood culture sampling in all patients suspected of hospital-acquired infections
☐ I do not know
☐ I do not want to answer
☐ Other: ……………

Due to many factors, there are times that doctors can not follow the recommendation(s) or guideline(s).

Q1-6. In your current hospital setting, how often do you plan to follow the recommendation(s) or guideline(s) for blood culture sampling being used in your hospital?

○ All the time (>95-100% of the cases)
○ Often (75-95% of the cases)
○ Moderately (25-74% of the cases)
○ Occasionally (5-24% of the cases)
○ Rarely (ranging from never to <5% of the cases)
○ I do not know
○ I do not want to answer

Q1-7. Apart from the recommendation(s) or guideline(s) being used at your hospital (as you answered in the previous question), do you have **any additional reasons** for deciding to do blood culture sampling? (you can select more than one answers that are applicable to your current hospital setting)

☐ No. All reasons are stated in the recommendation(s) or guideline(s) being used in my hospital.
☐ Patients presenting with chills
☐ Patients presenting with sepsis
☐ Patients presenting with septic shock
☐ Patients starting parenteral antibiotic treatment
☐ Patients with no clinical improvement after receiving empirical antibiotics
☐ Patients presenting with infection and having underlying diseases
☐ Patients presenting with chronic fever
☐ Patients presenting with fever of unknown origin
☐ Patients suspected of infections caused by atypical organisms
☐ Patients suspected of infections caused by antimicrobial-resistant organisms
☐ Patients suspected of infections caused by multiple-drug-resistant organisms
☐ Patients suspected of hospital-acquired infections
☐ Laboratory results showing leukocytosis
☐ Laboratory results showing neutropenia
☐ Laboratory results showing left shift in blood count (i.e. showing immature white blood cells)
☐ Laboratory results showing CRP increase
☐ Laboratory results showing procalcitonin increase
☐ Patients can afford the cost of blood culture
☐ Patients have a health scheme or insurance that covers the cost of blood culture

For peer review only - http://bmjopen.bmj.com/site/about/guidelines.xhtml

☐ Patients are likely to have a final diagnosis that includes the cost of blood culture in the package of fee for service
☐ I do not know
☐ I do not want to answer
☐ Other: ……………

(Skip to Q1-9 after this question)

(Page break)

Q1-8. In your current hospital setting, what are the **reasons** for deciding to do blood culture sampling? (you can select more than one answer that are applicable for your current hospital setting)

☐ Patients presenting with chills
☐ Patients presenting with sepsis
☐ Patients presenting with septic shock
☐ Patients presenting with infection and having underlying diseases
☐ Patients starting parenteral antibiotic treatment
☐ Patients with no clinical improvement after receiving empirical antibiotics
☐ Patients presenting with infection and having underlying diseases
☐ Patients presenting with chronic fever
☐ Patients presenting with fever of unknown origin
☐ Patients suspected of infections caused by atypical organisms
☐ Patients suspected of infections caused by antimicrobial-resistant organisms
☐ Patients suspected of infections caused by multiple-drug-resistant organisms
☐ Patients suspected of hospital-acquired infections
☐ Laboratory results showing leukocytosis
☐ Laboratory results showing neutropenia
☐ Laboratory results showing left shift in blood count
☐ Laboratory results showing CRP increase
☐ Laboratory results showing procalcitonin increase
☐ Patients can afford the cost of blood culture
☐ Patients have a health scheme or insurance that covers the cost of blood culture
☐ Patients are likely to have a final diagnosis that includes the cost of blood culture in the package of fee for service
☐ I do not know
☐ I do not want to answer
☐ Other: ……………

(Page break)

Q1-9. Are you aware of any international recommendation(s) or guideline(s) for blood culture sampling? Examples of international recommendations are surviving sepsis campaign (SSC), the diagnostic stewardship of the World Health Organization (WHO), The Infectious Diseases Society of America (IDSA) and The National Institute for Health and Care Excellence (NICE)

○ Yes
○ No (go to Q2-1)
○ I do not want to answer (go to Q2-1)

For peer review only - http://bmjopen.bmj.com/site/about/guidelines.xhtml

Q1-10. **Based on your understanding**, can any following statement(s) represent international recommendation(s) for blood culture sampling (you can select more than one answers)<br><br>☐ Recommend collecting blood culture in all patients presenting with sepsis<br>☐ Recommend collecting blood culture in all patients starting parenteral antibiotic treatment<br>☐ I do not know<br>☐ I do not want to answer<br>☐ Other:……………………
(Page break)
**We would like to understand your current job and how doctors in different positions are involved in ordering and collecting blood culture in your current hospital setting.**<br><br>Q2-1. First, please state your current job. (please select the most relevant answer)<br>○ Medical doctor – working in an executive or administrative position (not doing clinical work)<br>○ Medical doctor – working as a consultant (defined as a doctor with a clinical specialty/subspecialty degree)<br>○ Medical doctor – working as a physician (defined as a doctor without a clinical specialty/subspecialty degree and not under any postgraduate clinical training)<br>○ Medical doctor – working as a resident/registra/fellow (defined as a doctor who is currently under any postgraduate clinical training)<br>○ Intern (defined as a recent medical school graduate who is in the first year of post-graduate on-the-job training)<br>○ Final-year medical student<br>○ Other:……………
Final-year medical students (and interns) in some countries or some settings can **initiate an order** for a blood culture under authority of residents, consultants or other medical doctors. The order may be supervised, signed or co-signed by residents, consultants or other medical doctors later.<br><br>Q2-2. In your current hospital setting, which types of professionals/staff **can order** a blood culture. **"Order"** means initiating an order either verbally or in writing. (you can select more than one answers)<br><br>☐ Medical doctors – working in executive or administrative positions (not doing clinical work)<br>☐ Medical doctors – working as consultants (defined as a doctor with a clinical specialty/subspecialty degree)<br>☐ Medical doctors – working as physicians (defined as a doctor without a clinical specialty/subspecialty degree and not under any postgraduate clinical training)<br>☐ Medical doctors – working as residents/registras/fellows (defined as a doctor who is currently under any postgraduate clinical training)<br>☐ Interns (defined as recent medical school graduates who are in the first year of post-graduate on-the-job training)<br>☐ Final-year medical students<br>☐ I do not want to answer<br>☐ Other:……………
Q2-3. Do you know when and which patients should receive an **order** for a blood culture in your hospital?<br><br>○ Definitely (>95-100% of the case)<br>○ Likely (75-95% of the case)<br>○ Uncertain (25-74% of the case)<br>○ Unlikely (5-24% of the case)<br>○ Rarely (ranging from never to <5% of the case)<br>○ I do not know

For peer review only - http://bmjopen.bmj.com/site/about/guidelines.xhtml

○ I do not want to answer
Q2-4. If you **can order** for a blood culture as per your current job description or position, do you think that it is an appropriate part of your current job (as per your job description or position) to **order** a blood culture?<br><br>○ Very appropriate<br>○ Appropriate<br>○ Uncertain<br>○ Inappropriate<br>○ Very inappropriate<br>○ I cannot order blood culture. It is not part of my job (Go to Q2-5).<br>○ I do not know<br>○ I do not want to answer<br><br>(Skip to Q2-6 after this question, except answering “I cannot order blood culture. It is not part of my job”)
(Page break)
Q2-5. As you **cannot order** for a blood culture as per your current job description or position, do you think that it would be an appropriate part of your current job (as per your job description or position) to **order** a blood culture?<br><br>○ Very appropriate<br>○ Appropriate<br>○ Uncertain<br>○ Inappropriate<br>○ Very inappropriate<br>○ I do not know<br>○ I do not want to answer
(Page break)
Q2-6. In your current hospital setting, which **types of professionals** are tasked to **draw blood** from patients for blood culture. (you can select more than one answers)<br><br>☐ Medical doctors – working in executive or administrative positions (not doing clinical work)<br>☐ Medical doctors – working as consultants (defined as a doctor with a clinical specialty/subspecialty degree)<br>☐ Medical doctors – working as physicians (defined as a doctor without a clinical specialty/subspecialty degree and not under any postgraduate clinical training)<br>☐ Medical doctors – working as residents/registras/fellows (defined as a doctor who is currently under any postgraduate clinical training)<br>☐ Interns (defined as recent medical school graduates who are in the first year of post-graduate on-the-job training)☐ Interns<br>☐ Final-year medical students<br>☐ Registered nurses<br>☐ Microbiology laboratory team<br>☐ Specialized blood draw team<br>☐ I do not want to answer<br>☐ Other:……………
Q2-7. Do you think that it is an appropriate part of your job (as per your job description or position) to **draw blood**?

For peer review only - http://bmjopen.bmj.com/site/about/guidelines.xhtml

○ Very appropriate
○ Appropriate
○ Uncertain
○ Inappropriate
○ Very inappropriate
○ It is not part of my job to draw blood from patients for blood culture (go to Q2-11)
○ I do not know
○ I do not want to answer

(Page break)

Q2-8. How skilled are you in **drawing blood**?
○ Very good skill
○ Good skill
○ Fair skill
○ Poor skill
○ Very poor skill
○ I do not know
○ I do not want to answer

Having confidence is different from having skills. Due to many factors, there are times that blood could not be drawn even though we are skilled.

Q2-9. If you have to draw blood yourself, are you confident that **you can draw blood successfully**? "Successfully" means obtaining blood.
○ Strongly confident
○ Confident
○ Uncertain
○ Doubtful
○ Strongly doubtful
○ It is not part of my job to draw blood from patients for blood culture
○ I do not know
○ I do not want to answer

Q2-10. Are you confident that **you can draw blood appropriately**? "Appropriately" means that general recommendations for blood culture specimen collection such as aseptic technique are followed.
○ Strongly confident
○ Confident
○ Uncertain
○ Doubtful
○ Strongly doubtful
○ It is not part of my job to draw blood from patients for blood culture
○ I do not know
○ I do not want to answer

(Page break)

Q2-11. Are you confident that **others (who are tasked to draw blood in your hospital) can draw blood successfully**?
○ Strongly confident
○ Confident
○ Uncertain

For peer review only - http://bmjopen.bmj.com/site/about/guidelines.xhtml

○ Doubtful
○ Strongly doubtful
○ I do not know
○ I do not want to answer
○ I do not want to answer

Q2-12. Are you confident that **others (who are tasked to draw blood in your hospital) can draw blood appropriately**? "Appropriately" means that general recommendations for blood culture specimen collection such as aseptic technique are followed.

○ Strongly confident
○ Confident
○ Uncertain
○ Doubtful
○ Strongly doubtful
○ I do not know
○ I do not want to answer

Q2-13. In your current hospital setting, how **optimistic** are you that a blood culture will be sampled and processed in the laboratory appropriately if you order a blood culture? "Optimistic" means the confidence that things will happen for the best or that desired goals will be attained.

○ Strongly optimistic
○ Optimistic
○ Neither optimistic nor pessimistic
○ Pessimistic
○ Strongly pessimistic
○ I do not know
○ I do not want to answer

(Page break)

Many advantages and disadvantages of blood culture have been mentioned in surveys in different countries. This advantages and disadvantages could differ between settings.

Please answer of all following question to the best of your ability. Please a check mark "V " in the appropriate answer for each question.

Q3-1. Do you agree or disagree about the following potential advantages of blood culture, making blood culture helpful in your current hospital setting?	Strongly agree	Agree	Uncertain	Disagree	Strongly disagree	I do not know	I do not want to answer
• Blood culture is helpful in clinical decisions.							
• Blood culture is helpful to rule in an infection.							
• Blood culture is helpful to rule out an infection.							
• Blood culture is helpful in detecting antimicrobial-resistant bacterial infections.							
• Blood culture is helpful in adjusting antibiotics.							
• Blood culture can reduce overuse of antibiotics.							
• Blood culture can reduce length of hospital stay.							

For peer review only - http://bmjopen.bmj.com/site/about/guidelines.xhtml

• Blood culture can reduce patient mortality.							
• Accumulative results of blood culture (i.e. antimicrobial-resistance surveillance report) are helpful in understanding epidemiology of antimicrobial-resistant bacterial infections.							

Q3-2. Additional comments why blood culture is helpful in your current hospital setting (Note: limit to 2,000 characters)
………………………………………………………………

Please answer of all following question to the best of your ability. Please a check mark "√ " in the appropriate answer for each question.

Q3-3. Do you agree or disagree about the following disadvantages of blood culture, **making blood culture unnecessary in your current hospital setting?**	Strongly agree	Agree	Uncertain	Disagree	Strongly disagree	I do not know	I do not want to answer
• Blood culture is unnecessary because antibiotic therapy can be determined based on clinical presentations.							
• The therapeutic consequence of blood culture sampling is questionable.							
• The scientific basis of the guideline on blood culture is questionable							
• Blood culture is unnecessary because results are often delayed.							
• Blood culture is unnecessary because results are often not interpretable.							
• Blood culture is unnecessary because results are often negative or no growth.							
• Blood culture is unnecessary because cultures are often contaminated.							
• Blood culture is unnecessary because results often do not agree with clinical signs.							
• Blood culture is unnecessary because a contaminated result often leads to wrong therapeutic approaches.							
• Blood culture is unnecessary because it is too expensive.							
• Blood culture is not benefiting the patients.							
• It is not too late to collect blood culture later, particularly if patients do not improve after receiving empirical antibiotic treatment.							
• Quality of laboratory is questionable.							
• Levels of local antibiotic resistance are low.							

Q3-4. Additional comments why blood culture is not helpful in your current hospital setting (Note: limit to 2,000 characters)
………………………………………………………………

(Page break)

In different settings, other tasks may be considered more urgent than collecting blood culture samples.

Q3-5. In your current hospital setting, how often do you obtain blood culture **prior to administration of empirical antibiotics** in patients presenting with **sepsis**? '('sepsis' here is defined as an acute change in total Sequential Organ Failure Assessment [SOFA] score ≥2 points consequent to the infection based on the most recent definition of sepsis [Sepsis-3 criteria])

○ All the time (>95-100% of the time)

For peer review only - http://bmjopen.bmj.com/site/about/guidelines.xhtml

○ Often (75-95% of the time)
○ Moderately (25-74% of the time)
○ Occasionally (5-24% of the time)
○ Rarely (ranging from never to <5% of the time)
○ I do not know
○ I do not want to answer

Q3-6. In your current hospital setting, how often do you obtain blood culture **prior to administration of empirical antibiotics** in patients presenting with **septic shock**?
○ All the time (>95-100% of the time)
○ Often (75-95% of the time)
○ Moderately (25-74% of the time)
○ Occasionally (5-24% of the time)
○ Rarely (ranging from never to <5% of the time) ○ Rarely (ranging from never to <5% of the time)
○ I do not know
○ I do not want to answer

Even if blood culture is recommended, doctors may decide not to order blood culture in some situations.

Please answer of all following question to the best of your ability. Please a check mark "√ " in the appropriate answer for each question.

Q3-7. Would you still order blood culture in the following situation?	Definitely not order	Likely not order	Maybe not order	Likely to still order	Very likely to still order	I do not Know	I do not want to answer
• Patients are already on antibiotics.							
• Patients have anemia.							
• Blood should be used for other laboratory tests.							
• There are no local guidelines/recommendations for blood culture sampling							
• Patients do not meet certain conditions for a blood culture following the local guidelines							
• Patients do not have a health scheme or insurance that covers the cost of blood culture							
• Microbiology laboratory in your hospital is not available							

Q3-8. Additional comments why you do not order blood culture regarding situations mentioned above (Note: limit to 2,000 characters)
……………………………………………………………………

(Page break)

Resources are commonly limited in many settings worldwide.

Q4-1. In your hospital, how often could you (or doctors in your hospital) **not order blood culture** because consumables (such as blood culture bottles, needles, syringes, blood collection set, etc.) are **not available**?

○ All the time (>95-100% of the time)
○ Often (75-95% of the time)
○ Moderately (25-74% of the time)

For peer review only - http://bmjopen.bmj.com/site/about/guidelines.xhtml

○ Occasionally (5-24% of the time)
○ Rarely (ranging from never to <5% of the time)
○ I do not know
○ I do not want to answer

Q4-2. In your hospital, how often could you (or doctors in your hospital) **not order blood culture** because the microbiology laboratory is **not available** or not functioning?

○ All the time (>95-100% of the time)
○ Often (75-95% of the time)
○ Moderately (25-74% of the time)
○ Occasionally (5-24% of the time)
○ Rarely (ranging from never to <5% of the time)
○ I do not know
○ I do not want to answer

Q4-3. In your hospital, how often do patients have to pay for blood culture using their own money (i.e. out of pocket)?

○ All the time (>95-100% of the patients)
○ Often (75-95% of the patients)
○ Moderately (25-74% of the patients)
○ Occasionally (5-24% of the patients)
○ Rarely (ranging from never to <5% of the patients)
○ I do not know I do not know
○ I do not want to answer

Q4-4. Regardless of who pays for the cost of blood culture, would you say that the benefits of blood culture outweigh the cost?

○ Very likely
○ Likely
○ Uncertain
○ Unlikely
○ Very unlikely
○ I do not know
○ I do not want to answer

(Page break)

Positive and negative consequences could encourage us to follow guidelines.

Q5-1. Are there **any positive consequences, incentives or rewards** (these can be social [e.g. praise] or material [e.g. a positive score]) if you or doctors in your hospital **order a blood culture when recommended**? (you can select more than one answer)

☐ No
☐ Yes- social
☐ Yes- material
☐ Yes- both social and material
☐ I do not know
☐ I do not want to answer

For peer review only

☐ Other: ……………

Q5-2. Are there **any negative consequences** to you or doctors (these can be social [e.g. verbal reprimand or that you/doctors are at risk of being scrutinized] or material [e.g. a negative score]) if you or doctors in your hospital **do not order a blood culture when recommended**? (you can select more than one answer)

☐ No
☐ Yes- social
☐ Yes- material
☐ Yes- both social and material
☐ I do not know
☐ I do not want to answer
☐ Other: ……………

Sometimes there are feedbacks that could discourage us to follow guidelines. This could be due to many reasons based on local context.

Q5-3. Are there **any negative consequences** to you or doctors (these can be social [e.g. verbal reprimand or any pressure from your supervisors/executives of your hospital as the hospital (may) have to pay for the (extra) cost of blood culture] or material [e.g. a negative score, that you/doctors are at risk of having to spend extra time and effort to reimburse the cost of blood culture from any health scheme or insurance, or that you/doctors are at risk of having to pay for the (extra) cost of blood culture yourselves]), if you or doctors in your hospital **order blood culture when recommended**? (you can select more than one answer)

☐ No
☐ Yes- social
☐ Yes- material
☐ Yes- both social and material
☐ I do not know
☐ I do not want to answer
☐ Other: ……………

Q5-4. Additional comments about feedbacks (including encouragement, punishments or any positive and negative consequences) on blood culture sampling in your hospital setting. Also, please provide more comments about whether any consequences you would recommend to implement in your hospital to support blood culture ordering.

………………………………………………………………………

(Page break)

Q5-5. In your hospital, are there **any training, lectures, classes or meetings** that provide you knowledge about local/national/international guidelines for blood culture sampling? (you can select more than one answers)

☐ No
☐ Yes, infrequently (less than once a year)
☐ Yes, occasionally (at least once a year)
☐ Yes, regularly (more than once a year)
☐ I do not know
☐ I do not want to answer

For peer review only - http://bmjopen.bmj.com/site/about/guidelines.xhtml

☐ Other: ....

Q5-6. In your hospital, are there **any procedures** that support you or doctors in your hospital to order or regulate ordering of blood culture per local/national/international guidelines? (you can select more than one answers)

☐ No
☐ Yes, there is a poster (and blood culture is mentioned)
☐ Yes, there is a standard order form for patients presenting with sepsis (and blood culture is already written in the order form)
☐ Yes, there is a computer system to remind ordering blood culture
☐ Yes, there is a case review (e.g. grand round; morning ward round, clinical meetings, etc and blood culture is often mentioned)
☐ Yes, there is a stewardship programme and reviewing blood culture is included in the programme (e.g. post-prescription review and stewardship round, etc.)
☐ Yes, there is a local hospital guideline (e.g. standard operating procedure [SOP])
☐ I do not know
☐ I do not want to answer
☐ Other: ....

(Page break)

Due to different personal beliefs, norms and limitations, blood culture sampling is encouraged or discouraged by peers and co-workers in different settings.

Q6-1. **To what extent do you or doctors in your hospital order blood culture sampling because you are following local norms? "**Norms" mean usual practice that are typical of or accepted within your hospital.

o All the time (>95-100% of the time)
o Often (75-95% of the time)
o Moderately (25-74% of the time)
o Occasionally (5-24% of the time)
o Rarely (ranging from never to <5% of the time)
o I do not know
o I do not want to answer

Please answer of all following question to the best of your ability. Please a check mark "V " in the appropriate answer for each question.

Q6-2. **Do following people have any positive or negative influence on you or doctors in your hospital to order blood culture?** Positive influence could mean facilitate, support or encourage blood culture sampling. Negative influence could mean hinder or discourage blood culture sampling.	Very positive influence	Positive influence	Neither positive nor negative influence	Negative influence	Very negative influence	I do not know	I do not want to answer
• Nurses							
• Final-year medical students							
• Interns							
• Residents (any postgraduate clinical training)							

For peer review only - http://bmjopen.bmj.com/site/about/guidelines.xhtml

• Doctors (defined as a doctor without a specialty/subspecialty degree and not under any postgraduate clinical training)							
• Consultants (defined as a doctor with a clinical specialty/subspecialty degree)							
• Head of the Department							
• Executives of the hospital							
• Patients							
• Family of patients							

Q6-3. Additional comments about social influence on blood culture sampling
…………………………………………………………………

Q6-4. Apart from your logical considerations, do you think that **any emotional factors** of anyone are involved in ordering and sampling for blood culture (including patients and family of patients) could influence whether blood culture is ordered or sampled? (for example: fear of blood, fear of needle, fear of blood transmitted diseases, etc)

o No
o Other: ……………

Q6-5. Additional comments about emotional factors (from anyone who are involved in ordering and sampling for blood culture; including patients and family of patients) on blood culture sampling
…………………………………………………………………

(Page break)

Finally, we have some questions about yourself

Q7-1. Which country do you currently work in?

o Thailand
o Vietnam
o Indonesia
o I do not want to answer

Province of your current hospital:…………………………………… (Dropdown list for each country)

Q7-2. Are you female or male?

o Female
o Male
o Other
o I do not want to answer

For peer review only

Q7-3. What is the number of beds in your hospital? (Please use the official number, and please estimate if you are uncertain.)<br><br>o < 200<br>o 201 - 400<br>o 401 - 600<br>o 601 - 1,000<br>o 1,001 - 2,000<br>o > 2,000<br>o I do not know<br>o I do not want to answer
Q7-4. In which department are you **currently working**? If your role (such as medical students) moves from one department to another department over time, please state the current department you are working in.<br>(you can select more than one answers; for example both internal medicine and infectious disease devision)<br><br>☐ Internal Medicine<br>☐ Pediatrics<br>☐ Infection disease division/department<br>☐ Surgery<br>☐ Orthopaedics<br>☐ Obstetrics / Gynaecology<br>☐ Emergency department<br>☐ Intensive care unit<br>☐ I do not want to answer<br>☐ Other: ……………
(Page break)
Q7-5. Do you want to be contacted for further studies?<br><br>o Yes<br>o No
Q7-6. Do you want to be informed the results of this study?<br><br>o Yes<br>o No
Q7-7. Your email address (If you want to be contacted via email address. Please leave it blank, if you do not want to be contact via email address)<br>……………………………………………………………….
Q7-8. Your phone number (if you want to be contacted via phone. Please leave it blank, if you do not want to be contact via phone)<br>……………………………………………………………….
Please note that a gift or cash (about $4 in value) for completing the survey is to be provided to you. Participants could receive the gift electronically if email account or telephone number is provided.

For peer review only

Please make sure that you click “submit” on the next page to complete the questionnaire. Otherwise, all answers that you made and your information for compensation will not be submitted to us via the system.
(Page break)
We are grateful for your participation. Thank you very much.

For peer review only

For peer review only - http://bmjopen.bmj.com/site/about/guidelines.xhtml

Appendix S4. Survey results

Questions	Indonesia (n=503)	Thailand (n=304)	Viet Nam (n=501)	P value
**Type of hospitals (Q1-1)**				
Government hospital	340 (67.6%)	209 (68.8%)	431 (86.0%)	<0.001
Private hospital	113 (22.5%)	15 (4.9%)	17 (3.4%)	
University hospital	26 (5.2%)	76 (25.0%)	29 (5.8%)	
Other[1]	19 (3.8%)	2 (0.7%)	22 (4.4%)	
I do not want to answer	5 (1.0%)	2 (0.7%)	2 (0.4%)	
**Case-study: Would you take BC sample from a hypothetical sepsis case? (Q1-3)**				
Definitely (>95-100% of the time)	157 (31.2%)	273 (89.8%)	252 (50.3%)	<0.001
Likely (75-95% of the time)	138 (27.4%)	23 (7.6%)	149 (29.7%)	
Maybe (25-74% of the time)	116 (23.1%)	5 (1.6%)	70 (14.0%)	
Unlikely (5-24% of the time)	44 (8.7%)	2 (0.7%)	19 (3.8%)	
Rarely (ranging from never <5% of the time)	46 (9.1%)	1 (0.3%)	9 (1.8%)	
I do not know	1 (0.2%)	0 (0%)	1 (0.2%)	
I do not want to answer	1 (0.2%)	0 (0%)	1 (0.2%)	
**Knowledge (TDF-1): Do you know of any guideline(s) or guideline(s) used in my hospital (Q1-4)?**				
Yes	240 (47.7%)	169 (55.6%)	347 (69.3%)	<0.001
No, my hospital does not have any	68 (13.5%)	33 (10.9%)	49 (9.8%)	
No, I do not know if my hospital uses any	183 (36.4%)	98 (32.2%)	95 (19.0%)	
I do not want to answer	12 (2.4%)	4 (1.3%)	10 (2.0%)	
**Knowledge (TDF-1): known local guideline among those who answered that they know of local guideline (Q1-5)**				
All patients presenting with SIRS	155/240 (64.6%)	147/169 (87.0%)	218/347 (62.8%)	<0.001
All patients presenting with sepsis	183/240 (76.2%)	138/169 (81.7%)	291/347 (83.9%)	0.07
All patients presenting with septic shock	147/240 (61.3%)	131/169 (77.5%)	270/347 (77.8%)	<0.001
All patients starting parenteral antibiotic treatment	92/240 (38.3%)	92/169 (54.4%)	73/347 (21.0%)	<0.001
All patients with no clinical improvement after receiving empirical antibiotics	141/240 (58.7%)	99/169 (58.6%)	160/347 (46.1%)	0.003
All patients presenting with infection and having underlying diseases	76/240 (31.7%)	61/169 (36.1%)	94/347 (27.1%)	0.10
All patients with chronic fever	97/240 (40.4%)	87/169 (51.5%)	208/347 (59.9%)	<0.001
All patients with fever of unknown origins	114/240 (47.5%)	100/169 (59.2%)	185/347 (53.3%)	0.06
All patients suspected of infections caused by atypical organisms	97/240 (40.4%)	74/169 (43.8%)	94/347 (27.1%)	<0.001
All patients suspected of infections caused by antimicrobial-resistant organisms	131/240 (54.6%)	96/169 (56.8%)	168/347 (48.4%)	0.14
All patients suspected of infections caused by multiple-drug-resistant organisms	136/240 (56.7%)	103/169 (60.9%)	194/347 (55.9%)	0.54
All patients suspected of hospital-acquired infections	116/240 (48.3%)	99/169 (58.6%)	184/347 (53.0%)	0.12
**Intention (TDF-8): How often do you plan to follow the local guideline among those who answered that they know of local guideline (Q1-6)?**				
All the time (>95-100% of the cases)	70/240 (29.2%)	76/169 (45.0%)	88/347 (25.4%)	<0.001

For peer review only - http://bmjopen.bmj.com/site/about/guidelines.xhtml

Questions	Indonesia (n=503)	Thailand (n=304)	Viet Nam (n=501)	P value
Often (75-95% of the cases)	102/240 (42.5%)	81/169 (47.9%)	195/347 (56.2%)	
Moderately (25-74% of the cases)	33/240 (13.8%)	11/169 (6.5%)	49/347 (14.1%)	
Occasionally (5-24% of the cases)	16/240 (6.7%)	0/169 (0%)	11/347 (3.2%)	
Rarely (ranging from never <5% of the cases)	11/240 (4.6%)	1/169 (0.6%)	2/347 (0.6%)	
I do not know	7/240 (2.9%)	0/169 (0%)	2/347 (0.6%)	
I do not want to answer	1/240 (0.4%)	0/169 (0%)	0/347 (0%)	
**Memory, attention and decision processes (TDF-10): any additional reasons for deciding to do BC among those who answered that they know of local guideline (Q1-7)?**				
No additional reasons	77/240 (32.1%)	35/169 (20.7%)	110/347 (31.7%)	0.02
Patients presenting with chills	15/240 (6.3%)	39/169 (23.1%)	23/347 (6.6%)	<0.001
Patients presenting with sepsis	102/240 (42.5%)	101/169 (59.8%)	113/347 (32.6%)	<0.001
Patients presenting with septic shock	86/240 (35.8%)	96/169 (56.8%)	139/347 (40.1%)	<0.001
Patients starting parenteral antibiotic treatment	48/240 (20.0%)	59/169 (34.9%)	35/347 (10.1%)	<0.001
Patient with no clinical improvement after receiving empirical antibiotics	102/240 (42.5%)	75/169 (44.4%)	97/347 (28.0%)	<0.001
Patients with infection and having underlying diseases	42/240 (17.5%)	36/169 (21.3%)	56/347 (16.1%)	0.35
Patients presenting with chronic fever	54/240 (22.5%)	55/169 (32.5%)	107/347 (30.8%)	0.04
Patients presenting with fever of unknown origin	72/240 (30.0%)	63/169 (37.3%)	96/347 (27.7%)	0.08
Patients suspected of infections caused by atypical organisms	52/240 (21.7%)	46/169 (27.2%)	48/347 (13.8%)	0.001
Patients suspected of infections caused by antimicrobial-resistant organisms	77/240 (32.1%)	53/169 (31.4%)	86/347 (24.8%)	0.10
Patients suspected of infections caused by multiple-drug-resistant organisms	82/240 (34.2%)	63/169 (37.3%)	92/347 (26.5%)	0.03
Patients suspected of hospital-acquired infections	77/240 (32.1%)	59/169 (34.9%)	97/347 (28.0%)	0.24
Laboratory results showing leukocytosis	29/240 (12.1%)	42/169 (24.9%)	25/347 (7.2%)	<0.001
Laboratory results showing neutropenia	36/240 (15.0%)	54/169 (32.0%)	28/347 (8.1%)	<0.001
Laboratory results showing left shift in blood count	31/240 (12.9%)	26/169 (15.4%)	14/347 (4.0%)	<0.001
Laboratory results showing CRP increase	37/240 (15.4%)	22/169 (13.0%)	42/347 (12.1%)	0.51
Laboratory results showing procalcitonin increase	55/240 (22.9%)	22/169 (13.0%)	94/347 (27.1%)	0.002
Patients can afford the cost of BC	25/240 (10.4%)	9/169 (5.3%)	32/347 (9.2%)	0.18
Patients have a health scheme or insurance that covers the cost of BC	24/240 (10.0%)	8/169 (4.7%)	26/347 (7.5%)	0.14
Patients are likely to have a final diagnosis that includes the cost of BC in the package of fee for service	18/240 (7.5%)	0/169 (0%)	25/347 (7.2%)	0.001
**Memory, attention and decision processes (TDF-10): any reasons for deciding to do BC among those who did not answer that they know of local guideline (Q1-8)?**				
Patients presenting with chills	20/263 (7.6%)	49/135 (36.3%)	29/154 (18.8%)	<0.001
Patients presenting with sepsis	188/263 (71.5%)	132/135 (97.8%)	109/154 (70.8%)	<0.001
Patients presenting with septic shock	165/263 (62.7%)	128/135 (94.8%)	135/154 (87.7%)	<0.001
Patients starting parenteral antibiotic treatment	48/263 (18.3%)	95/135 (70.4%)	26/154 (16.9%)	<0.001

For peer review only - http://bmjopen.bmj.com/site/about/guidelines.xhtml

Questions	Indonesia (n=503)	Thailand (n=304)	Viet Nam (n=501)	P value
Patient with no clinical improvement after receiving empirical antibiotics	188/263 (71.5%)	119/135 (88.1%)	84/154 (54.5%)	<0.001
Patients with infection and having underlying diseases	85/263 (32.3%)	79/135 (58.5%)	52/154 (33.8%)	<0.001
Patients presenting with chronic fever	91/263 (34.6%)	89/135 (65.9%)	108/154 (70.1%)	<0.001
Patients presenting with fever of unknown origin	138/263 (52.5%)	110/135 (81.5%)	100/154 (64.9%)	<0.001
Patients suspected of infections caused by atypical organisms	123/263 (46.8%)	81/135 (60.0%)	55/154 (35.7%)	<0.001
Patients suspected of infections caused by antimicrobial-resistant organisms	177/263 (67.3%)	108/135 (80.0%)	85/154 (55.2%)	<0.001
Patients suspected of infections caused by multiple-drug-resistant organisms	183/263 (69.6%)	113/135 (83.7%)	85/354 (24.0%)	<0.001
Patients suspected of hospital-acquired infections	136/263 (51.7%)	107/135 (79.3%)	78/154 (50.6%)	<0.001
Laboratory results showing leukocytosis	41/263 (15.6%)	52/135 (38.5%)	15/154 (9.7%)	<0.001
Laboratory results showing neutropenia	34/263 (12.9%)	59/135 (43.7%)	18/154 (11.7%)	<0.001
Laboratory results showing left shift in blood count	47/263 (17.9%)	47/135 (34.8%)	16/154 (10.4%)	<0.001
Laboratory results showing CRP increase	59/263 (22.4%)	23/135 (17.0%)	26/154 (16.9%)	0.27
Laboratory results showing procalcitonin increase	73/263 (27.8%)	28/135 (20.7%)	53/154 (34.4%)	0.04
Patients can afford the cost of BC	81/263 (30.8%)	18/135 (13.3%)	32/154 (20.8%)	<0.001
Patients have a health scheme or insurance that covers the cost of BC	88/263 (33.5%)	19/135 (14.1%)	31/154 (20.1%)	<0.001
Patients are likely to have a final diagnosis that includes the cost of BC in the package of fee for service	51/263 (19.4%)	0/135 (0%)	30/154 (19.5%)	<0.001
**Knowledge (TDF-1): Do you know of any international guideline(s) or guideline(s) (Q1-9)?**				
Yes	229 (45.5%)	142 (46.7%)	225 (44.9%)	<0.001
No	263 (52.3%)	156 (51.3%)	233 (46.5%)	
I do not want to answer	11 (2.2%)	6 (2.0%)	43 (8.6%)	
**Knowledge (TDF-1): known international guideline or guideline among those who answered that they know of any international guideline(s) or guideline(s) (Q1-10)**				
BC sampling in all patients presenting with sepsis	220/229 (96.1%)	138/142 (97.2%)	208/225 (92.4%)	0.08
BC sampling in all patients starting parenteral antibiotic treatment	125/229 (54.6%)	87/142 (61.3%)	147/225 (65.3%)	<0.001
**Professional role (Q2-1): Current job**				
Medical doctor – an executive level	13 (2.6%)	5 (1.6%)	17 (3.4%)	<0.001
Medical doctor – a consultant level	74 (14.7%)	75 (24.7%)	198 (39.5%)	
Medical doctor – a general physician level	124 (24.7%)	38 (12.5%)	112 (22.4%)	
Medical doctor – a resident/registra/fellow level	168 (33.4%)	63 (20.7%)	101 (20.2%)	
Intern – recent medical school graduate	33 (6.6%)	35 (11.5%)	14 (2.8%)	
Final-year medical student	91 (18.1%)	88 (28.9%)	59 (11.8%)	
**Professional role (Q2-2): Which types of professionals/staff can order or initiate an order for a BC?**				

For peer review only - http://bmjopen.bmj.com/site/about/guidelines.xhtml

Questions	Indonesia (n=503)	Thailand (n=304)	Viet Nam (n=501)	P value
Medical doctor – an executive level	61 (12.1%)	163 (53.6%)	59 (11.8%)	<0.001
Medical doctor – a consultant level	431 (85.7%)	250 (82.2%)	439 (87.6%)	0.11
Medical doctor – a general physician level	265 (52.7%)	240 (78.9%)	347 (69.3%)	<0.001
Medical doctor – a resident (postgrad training) level	268 (53.3%)	242 (79.6%)	317 (63.3%)	<0.001
Intern – a recent medical school graduate level	83 (16.5%)	231 (76.0%)	118 (23.6%)	<0.001
Final-year medical student	11 (2.2%)	87 (28.6%)	3 (0.6%)	<0.001
I do not want to answer	3 (0.6%)	1 (0.3%)	11 (2.2%)	0.03
Other	0 (0%)	0 (0%)	0 (0%)	>0.99
**Knowledge (TDF-1): Do you know when and which patients should receive an order for a BC in your hospital (Q2-3)?**				
Definitely (>95-100% of the case)	65 (12.9%)	106 (34.9%)	72 (14.4%)	<0.001
Likely (75-95% of the case)	200 (39.8%)	168 (55.3%)	245 (48.9%)	
Uncertain (25-74% of the case)	148 (29.4%)	28 (9.2%)	128 (25.5%)	
Unlikely (5-24% of the case)	59 (11.7%)	0 (0%)	31 (6.2%)	
Rarely (ranging from never <5% of the case)	19 (3.8%)	0 (0%)	6 (1.2%)	
I do not know	10 (2.0%)	1 (0.3%)	8 (1.6%)	
I do not want to answer	2 (0.4%)	1 (0.3%)	11 (2.2%)	
**Social professional role and identity (TDF-3): Is it an appropriate part of your current job to order BC (Q2-4)?**				
Very appropriate	119 (23.7%)	103 (33.9%)	110 (22.0%)	<0.001
Appropriate	232 (46.1%)	166 (54.6%)	290 (57.9%)	
Uncertain	62 (12.3%)	20 (6.6%)	48 (9.6%)	
Inappropriate	21 (4.2%)	2 (0.7%)	12 (2.4%)	
Very inappropriate	2 (0.4%)	0 (0%)	0 (0%)	
I do not know	10 (2.0%)	0 (0%)	0 (0%)	
I do not want to answer	2 (0.4%)	0 (0%)	19 (3.8%)	
I cannot order BC. It is not part of my job	55 (10.9%)	13 (4.3%)	22 (4.4%)	
**Social professional role and identity (TDF-3): Would it be an appropriate part of your current job to order BC among those who answered that they cannot order for a BC (Q2-5)?**				
Very appropriate	4/55 (7.3%)	0/13 (0%)	0/22 (0%)	0.009
Appropriate	19/55 (34.5%)	8/13 (61.5%)	4/22 (18.2%)	
Uncertain	10/55 (18.2%)	4/13 (30.8%)	2/22 (9.1%)	
Inappropriate	15/55 (27.3%)	1/13 (7.7%)	8/22 (36.4%)	
Very inappropriate	3/55 (5.5%)	0/13 (0%)	2/22 (9.1%)	
I do not know	4/55 (7.3%)	0/13 (0%)	2/22 (9.1%)	
I do not want to answer	0/55 (0%)	0/13 (0%)	4/22 (18.2%)	
**Professional role (Q2-6): Which types of professionals/staff are tasked to draw blood from patients for BC?**				
Medical doctor – executive level	12 (2.4%)	44 (14.5%)	23 (4.6%)	<0.001
Medical doctor – a consultant level	60 (11.9%)	90 (29.6%)	152 (30.3%)	0.11
Medical doctor – a general physician level	72 (14.3%)	105 (34.5%)	129 (25.7%)	<0.001
Medical doctor – a resident level	96 (19.1%)	122 (40.1%)	113 (22.6%)	<0.001
Intern – recent medical school graduate	39 (7.8%)	105 (34.5%)	85 (17.0%)	<0.001
Final-year medical student	27 (5.4%)	99 (32.6%)	25 (5.0%)	<0.001
Registered nurses	342 (68.0%)	215 (70.7%)	392 (78.2%)	0.001

For peer review only - http://bmjopen.bmj.com/site/about/guidelines.xhtml

Questions	Indonesia (n=503)	Thailand (n=304)	Viet Nam (n=501)	P value
Microbiology laboratory team	227 (45.1%)	91 (29.9%)	151 (30.1%)	<0.001
Specialized blood draw team	197 (39.2%)	91 (29.9%)	69 (13.8%)	<0.001
I do not want to answer	3 (0.6%)	0 (0%)	2 (0.4%)	0.41
**Social professional role and identity (TDF-3): Is it an appropriate part of your current job to draw blood (Q2-7)?**				
Very appropriate	34 (6.8%)	36 (11.8%)	49 (9.8%)	0.01
Appropriate	179 (35.6%)	102 (33.6%)	179 (35.7%)	
Uncertain	109 (21.7%)	52 (17.1%)	68 (13.6%)	
Inappropriate	89 (17.7%)	46 (15.1%)	85 (17.0%)	
Very inappropriate	7 (1.4%)	6 (2.0%)	3 (0.6%)	
I do not know	8 (1.6%)	4 (1.3%)	4 (0.8%)	
I do not want to answer	4 (0.8%)	1 (0.3%)	4 (0.8%)	
It is not part of my job to draw blood	73 (14.5%)	57 (18.8%)	109 (21.8%)	
**Skill (TDF-2): How skilled are you in drawing blood excluding those whose jobs did not include drawing blood (Q2-8)?**				
Very good skill	18/430 (4.2%)	12/247 (4.9%)	32/392 (8.2%)	<0.001
Good skill	138/430 (32.1%)	46/247 (18.6%)	112/392 (28.6%)	
Fair skill	202/430 (47.0%)	118/247 (47.8%)	196/392 (50.0%)	
Poor skill	20/430 (4.7%)	52/247 (21.1%)	33/392 (8.4%)	
Very poor skill	4/430 (0.9%)	16/247 (6.5%)	1/392 (0.3%)	
I do not know	39/430 (9.1%)	3/247 (1.2%)	11/392 (2.8%)	
I do not want to answer	9/430 (2.1%)	0/247 (0%)	7/392 (1.8%)	
**Beliefs about capabilities (TDF-4): How confident that you can draw blood successfully excluding those whose jobs did not include drawing blood (Q2-9)?**				
Strongly confident	32/430 (7.4%)	20/247 (8.1%)	42/392 (10.7%)	<0.001
Confident	271/430 (63.0%)	93/247 (37.7%)	231/392 (58.9%)	
Uncertain	74/430 (17.2%)	81/247 (32.8%)	90/392 (23.0%)	
Doubtful	42/430 (9.8%)	34/247 (13.8%)	22/392 (5.6%)	
Strongly doubtful	2/430 (0.5%)	19/247 (7.7%)	6/392 (1.5%)	
I do not know	4/430 (0.9%)	0/247 (0%)	0/392 (0%)	
I do not want to answer	5/430 (1.2%)	0/247 (0%)	1/392 (0.3%)	
**Beliefs about capabilities (TDF-4): How confident that you can draw blood appropriately excluding those whose jobs did not include drawing blood (Q2-10)?**				
Strongly confident	28/430 (6.5%)	30/247 (12.1%)	37/392 (9.4%)	<0.001
Confident	262/430 (60.9%)	109/247 (44.1%)	222/392 (56.6%)	
Uncertain	86/430 (20.0%)	61/247 (24.7%)	109/392 (27.8%)	
Doubtful	44/430 (10.2%)	33/247 (13.4%)	17/392 (4.3%)	
Strongly doubtful	3/430 (0.7%)	11/247 (4.5%)	2/392 (0.5%)	
I do not know	3/430 (0.7%)	1/247 (0.4%)	1/392 (0.3%)	
I do not want to answer	4/430 (0.9%)	2/247 (0.8%)	4/392 (1.0%)	
**Beliefs about capabilities (TDF-4): Are you confident that others can draw blood successfully (Q2-11)?**				
Strongly confident	99 (19.7%)	106 (34.9%)	71 (14.2%)	<0.001
Confident	366 (72.8%)	176 (57.9%)	333 (66.5%)	
Uncertain	17 (3.4%)	14 (4.6%)	88 (17.6%)	

For peer review only - http://bmjopen.bmj.com/site/about/guidelines.xhtml

Questions	Indonesia (n=503)	Thailand (n=304)	Viet Nam (n=501)	P value
Doubtful	16 (3.2%)	7 (2.3%)	6 (1.2%)	
Strongly doubtful	0 (0%)	0 (0%)	1 (0.2%)	
I do not know	2 (0.4%)	1 (0.3%)	1 (0.2%)	
I do not want to answer	3 (0.6%)	0 (0%)	1 (0.2%)	
**Beliefs about capabilities (TDF-4): Are you confident that others can draw blood appropriately (Q2-12)?**				
Strongly confident	86 (17.1%)	66 (21.7%)	45 (9.0%)	<0.001
Confident	342 (68.0%)	184 (60.5%)	273 (54.5%)	
Uncertain	42 (8.3%)	45 (14.8%)	170 (33.9%)	
Doubtful	26 (5.2%)	6 (2.0%)	8 (1.6%)	
Strongly doubtful	1 (0.2%)	2 (0.7%)	2 (0.4%)	
I do not know	4 (0.8%)	1 (0.3%)	1 (0.2%)	
I do not want to answer	2 (0.4%)	0 (0%)	2 (0.4%)	
**Optimism (TDF-5): how optimistic are you that a BC will be sampled and processed in the laboratory appropriately (Q2-13)?**				
Strongly optimistic	70 (13.9%)	38 (12.5%)	31 (6.2%)	<0.001
Optimistic	332 (66.0%)	225 (74.0%)	338 (67.5%)	
Neither optimistic nor pessimistic	74 (14.7%)	31 (10.2%)	124 (24.8%)	
Pessimistic	8 (1.6%)	4 (1.3%)	4 (0.8%)	
Strongly pessimistic	5 (1.0%)	0 (0%)	1 (0.2%)	
I do not know	10 (2.0%)	5 (1.6%)	2 (0.4%)	
I do not want to answer	4 (0.8%)	1 (0.3%)	1 (0.2%)	
**Beliefs about consequence (TDF-6): BC is helpful in clinical decisions (Q3-1-1).**				
Strongly agree	204 (40.6%)	153 (50.3%)	194 (38.7%)	<0.001
Agree	279 (55.5%)	144 (47.4%)	246 (49.1%)	
Uncertain	13 (2.6%)	6 (2.0%)	47 (9.4%)	
Disagree	4 (0.8%)	1 (0.3%)	11 (2.2%)	
Strongly disagree	0 (0%)	0 (0%)	1 (0.2%)	
I do not know	2 (0.4%)	0 (0%)	0 (0%)	
I do not want to answer	1 (0.2%)	0 (0%)	2 (0.4%)	
**Beliefs about consequence (TDF-6): BC is helpful to rule in an infection (Q3-1-2).**				
Strongly agree	192 (38.2%)	123 (40.5%)	162 (32.3%)	<0.001
Agree	276 (54.9%)	159 (52.3%)	260 (51.9%)	
Uncertain	14 (2.8%)	10 (3.3%)	51 (10.2%)	
Disagree	18 (3.6%)	7 (2.3%)	24 (4.8%)	
Strongly disagree	0 (0%)	1 (0.3%)	2 (0.4%)	
I do not know	2 (0.4%)	4 (1.3%)	0 (0%)	
I do not want to answer	1 (0.2%)	0 (0%)	2 (0.4%)	
**Beliefs about consequence (TDF-6): BC is helpful to rule out an infection (Q3-1-3).**				
Strongly agree	137 (27.2%)	72 (23.7%)	59 (11.8%)	<0.001
Agree	258 (51.3%)	97 (31.9%)	163 (32.5%)	
Uncertain	44 (8.7%)	32 (10.5%)	126 (25.1%)	
Disagree	56 (11.1%)	79 (26.0%)	127 (25.3%)	
Strongly disagree	5 (1.0%)	22 (7.2%)	23 (4.6%)	
I do not know	2 (0.4%)	2 (0.7%)	0 (0%)	
I do not want to answer	1 (0.2%)	0 (0%)	3 (0.6%)	

For peer review only

Questions	Indonesia (n=503)	Thailand (n=304)	Viet Nam (n=501)	P value
**Beliefs about consequence (TDF-6): BC is helpful in detecting AMR infections (Q3-1-4).**				
Strongly agree	267 (53.1%)	147 (48.4%)	154 (30.7%)	<0.001
Agree	219 (43.5%)	140 (46.1%)	272 (54.3%)	
Uncertain	10 (2.0%)	11 (3.6%)	51 (10.2%)	
Disagree	4 (0.8%)	4 (1.3%)	18 (3.6%)	
Strongly disagree	0 (0%)	1 (0.3%)	4 (0.8%)	
I do not know	2 (0.4%)	1 (0.3%)	1 (0.2%)	
I do not want to answer	1 (0.2%)	0 (0%)	1 (0.2%)	
**Beliefs about consequence (TDF-6): BC is helpful in adjusting antibiotics (Q3-1-5).**				
Strongly agree	285 (56.7%)	172 (56.6%)	177 (35.3%)	<0.001
Agree	206 (41.0%)	128 (42.1%)	256 (51.1%)	
Uncertain	9 (1.8%)	2 (0.7%)	40 (8.0%)	
Disagree	0 (0%)	1 (0.3%)	21 (4.2%)	
Strongly disagree	1 (0.2%)	1 (0.3%)	3 (0.6%)	
I do not know	1 (0.2%)	0 (0%)	0 (0%)	
I do not want to answer	1 (0.2%)	0 (0%)	4 (0.8%)	
**Beliefs about consequence (TDF-6): BC can reduce overuse of antibiotics (Q3-1-6).**				
Strongly agree	241 (47.9%)	142 (46.7%)	157 (31.3%)	<0.001
Agree	220 (43.7%)	131 (43.1%)	249 (49.7%)	
Uncertain	30 (6.0%)	19 (6.3%)	59 (11.8%)	
Disagree	9 (1.8%)	11 (3.6%)	30 (6.0%)	
Strongly disagree	1 (0.2%)	1 (0.3%)	4 (0.8%)	
I do not know	1 (0.2%)	0 (0%)	0 (0%)	
I do not want to answer	1 (0.2%)	0 (0%)	2 (0.4%)	
**Beliefs about consequence (TDF-6): BC can reduce length of hospital stay (Q3-1-7).**				
Strongly agree	167 (33.2%)	101 (33.2%)	106 (21.2%)	<0.001
Agree	215 (42.7%)	122 (40.1%)	227 (45.3%)	
Uncertain	97 (19.3%)	54 (17.8%)	124 (24.8%)	
Disagree	18 (3.6%)	23 (7.6%)	39 (7.8%)	
Strongly disagree	0 (0%)	2 (0.7%)	3 (0.6%)	
I do not know	4 (0.8%)	1 (0.3%)	0 (0%)	
I do not want to answer	2 (0.4%)	1 (0.3%)	2 (0.4%)	
**Beliefs about consequence (TDF-6): BC can reduce patient mortality (Q3-1-8).**				
Strongly agree	178 (35.4%)	120 (39.5%)	124 (24.8%)	<0.001
Agree	228 (45.3%)	135 (44.4%)	242 (48.3%)	
Uncertain	79 (15.7%)	38 (12.5%)	98 (19.6%)	
Disagree	12 (2.4%)	8 (2.6%)	31 (6.2%)	
Strongly disagree	1 (0.2%)	0 (0%)	3 (0.6%)	
I do not know	4 (0.8%)	3 (1.0%)	1 (0.2%)	
I do not want to answer	1 (0.2%)	0 (0%)	2 (0.4%)	
**Beliefs about consequence (TDF-6): Accumulative results of BC are helpful in understanding epidemiology of AMR bacterial infections (Q3-1-9).**				
Strongly agree	237 (47.1%)	144 (47.4%)	193 (38.5%)	0.003
Agree	247 (49.1%)	141 (46.4%)	266 (53.1%)	
Uncertain	13 (2.6%)	16 (5.3%)	32 (6.4%)	

For peer review only - http://bmjopen.bmj.com/site/about/guidelines.xhtml

Questions	Indonesia (n=503)	Thailand (n=304)	Viet Nam (n=501)	P value
Disagree	0 (0%)	1 (0.3%)	7 (1.4%)	
Strongly disagree	1 (0.2%)	0 (0%)	1 (0.2%)	
I do not know	4 (0.8%)	2 (0.7%)	0 (0%)	
I do not want to answer	1 (0.2%)	0 (0%)	2 (0.4%)	
**Beliefs about consequence (TDF-6): BC is unnecessary because antibiotic therapy can be determined based on clinical presentations (Q3-3-1).**				
Strongly agree	13 (2.6%)	7 (2.3%)	18 (3.6%)	<0.001
Agree	89 (17.7%)	48 (15.8%)	53 (10.6%)	
Uncertain	154 (30.6%)	48 (15.8%)	113 (22.6%)	
Disagree	199 (39.6%)	146 (48.0%)	264 (52.7%)	
Strongly disagree	42 (8.3%)	54 (17.8%)	53 (10.6%)	
I do not know	6 (1.2%)	1 (0.3%)	0 (0%)	
I do not want to answer	0 (0%)	0 (0%)	0 (0%)	
**Beliefs about consequence (TDF-6): The therapeutic consequence of BC sampling is questionable (Q3-3-2).**				
Strongly agree	12 (2.4%)	25 (8.2%)	16 (3.2%)	<0.001
Agree	82 (16.3%)	58 (19.1%)	45 (9.0%)	
Uncertain	167 (33.2%)	60 (19.7%)	123 (24.6%)	
Disagree	191 (38.0%)	116 (38.2%)	275 (54.9%)	
Strongly disagree	34 (6.8%)	39 (12.8%)	34 (6.8%)	
I do not know	17 (3.4%)	5 (1.6%)	2 (0.4%)	
I do not want to answer	0 (0%)	1 (0.3%)	6 (1.2%)	
**Beliefs about consequence (TDF-6): The scientific basis of the guideline on BC is questionable (Q3-3-3).**				
Strongly agree	9 (1.8%)	16 (5.3%)	15 (3.0%)	<0.001
Agree	45 (8.9%)	63 (20.7%)	43 (8.6%)	
Uncertain	106 (21.1%)	58 (19.1%)	141 (28.1%)	
Disagree	248 (49.3%)	120 (39.5%)	254 (50.7%)	
Strongly disagree	79 (15.7%)	39 (12.8%)	41 (8.2%)	
I do not know	15 (3.0%)	7 (2.3%)	4 (0.8%)	
I do not want to answer	1 (0.2%)	1 (0.3%)	3 (0.6%)	
**Beliefs about consequence (TDF-6): BC is unnecessary because results are often delayed (Q3-3-4).**				
Strongly agree	15 (3.0%)	8 (2.6%)	15 (3.0%)	<0.001
Agree	113 (22.5%)	31 (10.2%)	38 (7.6%)	
Uncertain	119 (23.7%)	23 (7.6%)	82 (16.4%)	
Disagree	212 (42.1%)	161 (53.0%)	303 (60.5%)	
Strongly disagree	36 (7.2%)	80 (26.3%)	62 (12.4%)	
I do not know	8 (1.6%)	0 (0%)	0 (0%)	
I do not want to answer	0 (0%)	1 (0.3%)	1 (0.2%)	
**Beliefs about consequence (TDF-6): BC is unnecessary because results are often not interpretable (Q3-3-5).**				
Strongly agree	7 (1.4%)	4 (1.3%)	11 (2.2%)	<0.001
Agree	46 (9.1%)	18 (5.9%)	26 (5.2%)	
Uncertain	120 (23.9%)	18 (5.9%)	70 (14.0%)	
Disagree	275 (54.7%)	166 (54.6%)	326 (65.1%)	

For peer review only

For peer review only - http://bmjopen.bmj.com/site/about/guidelines.xhtml

Questions	Indonesia (n=503)	Thailand (n=304)	Viet Nam (n=501)	P value
Strongly disagree	47 (9.3%)	97 (31.9%)	67 (13.4%)	
I do not know	7 (1.4%)	1 (0.3%)	0 (0%)	
I do not want to answer	1 (0.2%)	0 (0%)	1 (0.2%)	
**Beliefs about consequence (TDF-6): BC is unnecessary because results are often negative or no growth (Q3-3-6).**				
Strongly agree	9 (1.8%)	6 (2.0%)	11 (2.2%)	<0.001
Agree	57 (11.3%)	26 (8.6%)	39 (7.8%)	
Uncertain	114 (22.7%)	37 (12.2%)	83 (16.6%)	
Disagree	261 (51.9%)	149 (49.0%)	312 (62.3%)	
Strongly disagree	51 (10.1%)	85 (28.0%)	55 (11.0%)	
I do not know	10 (2.0%)	1 (0.3%)	0 (0%)	
I do not want to answer	1 (0.2%)	0 (0%)	1 (0.2%)	
**Beliefs about consequence (TDF-6): BC is unnecessary because cultures are often contaminated (Q3-3-7).**				
Strongly agree	8 (1.6%)	6 (2.0%)	10 (2.0%)	<0.001
Agree	65 (12.9%)	23 (7.6%)	31 (6.2%)	
Uncertain	166 (33.0%)	44 (14.5%)	105 (21.0%)	
Disagree	212 (42.1%)	153 (50.3%)	290 (57.9%)	
Strongly disagree	39 (7.8%)	77 (25.3%)	59 (11.8%)	
I do not know	12 (2.4%)	0 (0%)	1 (0.2%)	
I do not want to answer	1 (0.2%)	1 (0.3%)	5 (1.0%)	
**Beliefs about consequence (TDF-6): BC is unnecessary because results often do not agree with clinical signs (Q3-3-8).**				
Strongly agree	8 (1.6%)	5 (1.6%)	13 (2.6%)	<0.001
Agree	46 (9.1%)	22 (7.2%)	21 (4.2%)	
Uncertain	147 (29.2%)	36 (11.8%)	84 (16.8%)	
Disagree	249 (49.5%)	158 (52.0%)	325 (64.9%)	
Strongly disagree	43 (8.5%)	83 (27.3%)	49 (9.8%)	
I do not know	10 (2.0%)	0 (0%)	0 (0%)	
I do not want to answer	0 (0%)	0 (0%)	9 (1.8%)	
**Beliefs about consequence (TDF-6): BC is unnecessary because a contaminated result often leads to wrong therapeutic approaches (Q3-3-9).**				
Strongly agree	10 (2.0%)	7 (2.3%)	14 (2.8%)	<0.001
Agree	85 (16.9%)	23 (7.6%)	38 (7.6%)	
Uncertain	128 (25.4%)	42 (13.8%)	116 (23.2%)	
Disagree	229 (45.5%)	148 (48.7%)	277 (55.3%)	
Strongly disagree	41 (8.2%)	83 (27.3%)	42 (8.4%)	
I do not know	9 (1.8%)	1 (0.3%)	3 (0.6%)	
I do not want to answer	1 (0.2%)	0 (0%)	11 (2.2%)	
**Environmental context and resources (TDF-11): BC is unnecessary because it is too expensive (Q3-3-10).**				
Strongly agree	25 (5.0%)	6 (2.0%)	12 (2.4%)	<0.001
Agree	83 (16.5%)	19 (6.3%)	24 (4.8%)	
Uncertain	114 (22.7%)	37 (12.2%)	79 (15.8%)	
Disagree	227 (45.1%)	133 (43.8%)	310 (61.9%)	
Strongly disagree	39 (7.8%)	103 (33.9%)	64 (12.8%)	

For peer review only - http://bmjopen.bmj.com/site/about/guidelines.xhtml

Questions	Indonesia (n=503)	Thailand (n=304)	Viet Nam (n=501)	P value
I do not know	12 (2.4%)	5 (1.6%)	2 (0.4%)	
I do not want to answer	3 (0.6%)	1 (0.3%)	10 (2.0%)	
**Beliefs about consequence (TDF-6): BC is not benefiting the patients (Q3-3-11).**				
Strongly agree	5 (1.0%)	5 (1.6%)	10 (2.0%)	<0.001
Agree	19 (3.8%)	17 (5.6%)	20 (4.0%)	
Uncertain	88 (17.5%)	13 (4.3%)	46 (9.2%)	
Disagree	290 (57.7%)	139 (45.7%)	302 (60.3%)	
Strongly disagree	92 (18.3%)	130 (42.8%)	121 (24.2%)	
I do not know	8 (1.6%)	0 (0%)	0 (0%)	
I do not want to answer	1 (0.2%)	0 (0%)	2 (0.4%)	
**Beliefs about consequence (TDF-6): It is not too late to collect BC later, particularly if patients do not improve after receiving empirical antibiotic treatment (Q3-3-12).**				
Strongly agree	23 (4.6%)	48 (15.8%)	15 (3.0%)	<0.001
Agree	116 (23.1%)	114 (37.5%)	107 (21.4%)	
Uncertain	95 (18.9%)	32 (10.5%)	89 (17.8%)	
Disagree	208 (41.4%)	65 (21.4%)	226 (45.1%)	
Strongly disagree	49 (9.7%)	45 (14.8%)	61 (12.2%)	
I do not know	11 (2.2%)	0 (0%)	3 (0.6%)	
I do not want to answer	1 (0.2%)	0 (0%)	0 (0%)	
**Beliefs about consequence (TDF-6): Quality of laboratory is questionable (Q3-3-13).**				
Strongly agree	15 (3.0%)	11 (3.6%)	9 (1.8%)	<0.001
Agree	77 (15.3%)	27 (8.9%)	55 (11.0%)	
Uncertain	147 (29.2%)	81 (26.6%)	148 (29.5%)	
Disagree	196 (39.0%)	114 (37.5%)	239 (47.7%)	
Strongly disagree	48 (9.5%)	62 (20.4%)	40 (8.0%)	
I do not know	18 (3.6%)	8 (2.6%)	5 (1.0%)	
I do not want to answer	2 (0.4%)	1 (0.3%)	5 (1.0%)	
**Beliefs about consequence (TDF-6): Levels of local antibiotic resistance are low (Q3-3-14).**				
Strongly agree	5 (1.0%)	4 (1.3%)	8 (1.6%)	<0.001
Agree	45 (8.9%)	22 (7.2%)	42 (8.4%)	
Uncertain	120 (23.9%)	63 (20.7%)	111 (22.2%)	
Disagree	225 (44.7%)	130 (42.8%)	268 (53.5%)	
Strongly disagree	87 (17.3%)	77 (25.3%)	68 (13.6%)	
I do not know	21 (4.2%)	7 (2.3%)	3 (0.6%)	
I do not want to answer	0 (0%)	1 (0.3%)	1 (0.2%)	
**Goals (TDF-9): How often do you obtain BC prior to administration of empirical antibiotics in patients presenting with sepsis (Q3-5)?**				
All the time (>95-100% of the time)	95 (18.9%)	158 (52.0%)	150 (29.9%)	<0.001
Often (75-95% of the time)	156 (31.0%)	116 (38.2%)	230 (45.9%)	
Moderately (25-74% of the time)	85 (16.9%)	21 (6.9%)	64 (12.8%)	
Occasionally (5-24% of the time)	45 (8.9%)	5 (1.6%)	12 (2.4%)	
Rarely (ranging from never <5% of the time)	82 (16.3%)	0 (0%)	19 (3.8%)	
I do not know	34 (6.8%)	4 (1.3%)	11 (2.2%)	
I do not want to answer	6 (1.2%)	0 (0%)	15 (3.0%)	

For peer review only - http://bmjopen.bmj.com/site/about/guidelines.xhtml

Questions	Indonesia (n=503)	Thailand (n=304)	Viet Nam (n=501)	P value
**Goals (TDF-9): How often do you obtain BC prior to administration of empirical antibiotics in patients presenting with septic shock (Q3-6)?**				
All the time (>95-100% of the time)	90 (17.9%)	234 (77.0%)	218 (43.5%)	<0.001
Often (75-95% of the time)	160 (31.8%)	59 (19.4%)	175 (34.9%)	
Moderately (25-74% of the time)	76 (15.1%)	6 (2.0%)	48 (9.6%)	
Occasionally (5-24% of the time)	48 (9.5%)	0 (0%)	18 (3.6%)	
Rarely (ranging from never <5% of the time)	84 (16.7%)	0 (0%)	20 (4.0%)	
I do not know	40 (8.0%)	3 (1.0%)	9 (1.8%)	
I do not want to answer	5 (1.0%)	2 (0.7%)	13 (2.6%)	
**Memory, attention and decision processes (TDF-10): Would you still order BC if patients are already on antibiotics (Q3-7-1)?**				
Definitely not order	11 (2.2%)	14 (4.6%)	6 (1.2%)	<0.001
Likely not order	19 (3.8%)	53 (17.4%)	28 (5.6%)	
Maybe not order	295 (58.6%)	38 (12.5%)	85 (17.0%)	
Likely to still order	143 (28.4%)	116 (38.2%)	308 (61.5%)	
Very likely to still order	18 (3.6%)	81 (26.6%)	72 (14.4%)	
I do not know	16 (3.2%)	2 (0.7%)	1 (0.2%)	
I do not want to answer	1 (0.2%)	0 (0%)	1 (0.2%)	
**Memory, attention and decision processes (TDF-10): Would you still order BC if patients have anemia (Q3-7-2)?**				
Definitely not order	16 (3.2%)	84 (27.6%)	24 (4.8%)	<0.001
Likely not order	59 (11.7%)	64 (21.1%)	33 (6.6%)	
Maybe not order	255 (50.7%)	52 (17.1%)	58 (11.6%)	
Likely to still order	124 (24.7%)	52 (17.1%)	257 (51.3%)	
Very likely to still order	20 (4.0%)	45 (14.8%)	115 (23.0%)	
I do not know	28 (5.6%)	5 (1.6%)	2 (0.4%)	
I do not want to answer	1 (0.2%)	2 (0.7%)	12 (2.4%)	
**Memory, attention and decision processes (TDF-10): Would you still order BC if blood should be used for other laboratory tests (Q3-7-3)?**				
Definitely not order	7 (1.4%)	57 (18.8%)	59 (11.8%)	<0.001
Likely not order	43 (8.5%)	57 (18.8%)	64 (12.8%)	
Maybe not order	228 (45.3%)	75 (24.7%)	117 (23.4%)	
Likely to still order	158 (31.4%)	63 (20.7%)	172 (34.3%)	
Very likely to still order	20 (4.0%)	40 (13.2%)	60 (12.0%)	
I do not know	41 (8.2%)	12 (3.9%)	21 (4.2%)	
I do not want to answer	6 (1.2%)	0 (0%)	8 (1.6%)	
**Memory, attention and decision processes (TDF-10): Would you still order BC if there are no local guidelines/guidelines for BC sampling (Q3-7-4)?**				
Definitely not order	11 (2.2%)	42 (13.8%)	42 (8.4%)	<0.001
Likely not order	41 (8.2%)	43 (14.1%)	66 (13.2%)	
Maybe not order	241 (47.9%)	95 (31.3%)	136 (27.1%)	
Likely to still order	152 (30.2%)	66 (21.7%)	174 (34.7%)	
Very likely to still order	19 (3.8%)	33 (10.9%)	41 (8.2%)	
I do not know	32 (6.4%)	24 (7.9%)	35 (7.0%)	
I do not want to answer	7 (1.4%)	1 (0.3%)	7 (1.4%)	

For peer review only - http://bmjopen.bmj.com/site/about/guidelines.xhtml

Questions	Indonesia (n=503)	Thailand (n=304)	Viet Nam (n=501)	P value
**Memory, attention and decision processes (TDF-10): Would you still order BC if patients do not meet certain conditions for a BC following the local guidelines (Q3-7-5)?**				
Definitely not order	28 (5.6%)	39 (12.8%)	54 (10.8%)	<0.001
Likely not order	131 (26.0%)	80 (26.3%)	93 (18.6%)	
Maybe not order	250 (49.7%)	93 (30.6%)	177 (35.3%)	
Likely to still order	58 (11.5%)	54 (17.8%)	121 (24.2%)	
Very likely to still order	11 (2.2%)	22 (7.2%)	44 (8.8%)	
I do not know	23 (4.6%)	15 (4.9%)	8 (1.6%)	
I do not want to answer	2 (0.4%)	1 (0.3%)	4 (0.8%)	
**Memory, attention and decision processes (TDF-10): Would you still order BC if patients do not have a health scheme or insurance that covers the cost of BC (Q3-7-6)?**				
Definitely not order	39 (7.8%)	7 (2.3%)	21 (4.2%)	<0.001
Likely not order	56 (11.1%)	33 (10.9%)	43 (8.6%)	
Maybe not order	306 (60.8%)	95 (31.3%)	101 (20.2%)	
Likely to still order	68 (13.5%)	87 (28.6%)	265 (52.9%)	
Very likely to still order	6 (1.2%)	63 (20.7%)	61 (12.2%)	
I do not know	23 (4.6%)	14 (4.6%)	5 (1.0%)	
I do not want to answer	5 (1.0%)	5 (1.6%)	5 (1.0%)	
**Memory, attention and decision processes (TDF-10): Would you still order BC if microbiology laboratory in your hospital is not available (Q3-7-7)?**				
Definitely not order	53 (10.5%)	21 (6.9%)	97 (19.4%)	<0.001
Likely not order	114 (22.7%)	53 (17.4%)	101 (20.2%)	
Maybe not order	229 (45.5%)	77 (25.3%)	120 (24.0%)	
Likely to still order	74 (14.7%)	79 (26.0%)	109 (21.8%)	
Very likely to still order	10 (2.0%)	54 (17.8%)	36 (7.2%)	
I do not know	19 (3.8%)	12 (3.9%)	30 (6.0%)	
I do not want to answer	4 (0.8%)	8 (2.6%)	8 (1.6%)	
**Environmental context and resources (TDF-11): How often could you not order BC because consumables are not available (Q4-1)?**				
All the time (>95-100% of the time)	24 (4.8%)	12 (3.9%)	19 (3.8%)	<0.001
Often (75-95% of the time)	61 (12.1%)	15 (4.9%)	19 (3.8%)	
Moderately (25-74% of the time)	52 (10.3%)	11 (3.6%)	56 (11.2%)	
Occasionally (5-24% of the time)	86 (17.1%)	15 (4.9%)	51 (10.2%)	
Rarely (ranging from never <5% of the time)	219 (43.5%)	232 (76.3%)	309 (61.7%)	
I do not know	53 (10.5%)	18 (5.9%)	25 (5.0%)	
I do not want to answer	8 (1.6%)	1 (0.3%)	22 (4.4%)	
**Environmental context and resources (TDF-11): How often could you not order BC because the microbiology laboratory is not available or not functioning (Q4-2)?**				
All the time (>95-100% of the time)	34 (6.8%)	9 (3.0%)	15 (3.0%)	<0.001
Often (75-95% of the time)	58 (11.5%)	13 (4.3%)	28 (5.6%)	
Moderately (25-74% of the time)	48 (9.5%)	9 (3.0%)	37 (7.4%)	
Occasionally (5-24% of the time)	78 (15.5%)	14 (4.6%)	27 (5.4%)	
Rarely (ranging from never <5% of the time)	224 (44.5%)	238 (78.3%)	342 (68.3%)	

For peer review only - http://bmjopen.bmj.com/site/about/guidelines.xhtml

Questions	Indonesia (n=503)	Thailand (n=304)	Viet Nam (n=501)	P value
I do not know	56 (11.1%)	21 (6.9%)	28 (5.6%)	
I do not want to answer	5 (1.0%)	0 (0%)	24 (4.8%)	
**Environmental context and resources (TDF-11): How often do patients have to pay for BC using their own money (i.e. out of pocket) (Q4-3)?**				
All the time (>95-100% of the time)	26 (5.2%)	11 (3.6%)	6 (1.2%)	<0.001
Often (75-95% of the time)	52 (10.3%)	17 (5.6%)	28 (5.6%)	
Moderately (25-74% of the time)	50 (9.9%)	19 (6.3%)	67 (13.4%)	
Occasionally (5-24% of the time)	69 (13.7%)	48 (15.8%)	134 (26.7%)	
Rarely (ranging from never <5% of the time)	138 (27.4%)	135 (44.4%)	173 (34.5%)	
I do not know	163 (32.4%)	73 (24.0%)	72 (14.4%)	
I do not want to answer	5 (1.0%)	1 (0.3%)	21 (4.2%)	
**Environmental context and resources (TDF-11): Would you say that the benefits of BC outweigh the cost (Q4-4)?**				
Very likely	101 (20.1%)	135 (44.4%)	184 (36.7%)	<0.001
Likely	210 (41.7%)	97 (31.9%)	223 (44.5%)	
Uncertain	93 (18.5%)	37 (12.2%)	34 (6.8%)	
Unlikely	45 (8.9%)	10 (3.3%)	16 (3.2%)	
Very unlikely	3 (0.6%)	13 (4.3%)	17 (3.4%)	
I do not know	49 (9.7%)	12 (3.9%)	17 (3.4%)	
I do not want to answer	2 (0.4%)	0 (0%)	10 (2.0%)	
**Reinforcement (TDF-7): Are there any positive consequences if you order a BC when recommended (Q5-1)?**				
No	283 (56.3%)	187 (61.5%)	206 (41.1%)	<0.001
Yes, social	31 (6.2%)	37 (12.2%)	59 (11.8%)	
Yes, material	4 (0.8%)	2 (0.7%)	8 (1.6%)	
Yes, both social and material	33 (6.6%)	18 (5.9%)	103 (20.6%)	
I do not know	143 (28.4%)	58 (19.1%)	75 (15.0%)	
I do not want to answer	8 (1.6%)	1 (0.3%)	45 (9.0%)	
Other	1 (0.2%)	1 (0.3%)	5 (1.0%)	
**Reinforcement (TDF-7): Are there any negative consequences if you do not order a BC when recommended (Q5-2)?**				
No	248 (49.3%)	101 (33.2%)	134 (26.7%)	<0.001
Yes, social	65 (12.9%)	115 (37.8%)	100 (20.0%)	
Yes, material	8 (1.6%)	4 (1.3%)	13 (2.6%)	
Yes, both social and material	27 (5.4%)	22 (7.2%)	111 (22.2%)	
I do not know	142 (28.2%)	60 (19.7%)	83 (16.6%)	
I do not want to answer	12 (2.4%)	2 (0.7%)	55 (11.0%)	
Other	1 (0.2%)	0 (0%)	5 (1.0%)	
**Reinforcement (TDF-7): Are there any negative consequences if you order a BC when recommended (Q5-3)?**				
No	251 (49.9%)	162 (53.3%)	210 (41.9%)	<0.001
Yes, social	47 (9.3%)	43 (14.1%)	31 (6.2%)	
Yes, material	10 (2.0%)	3 (1.0%)	31 (6.2%)	
Yes, both social and material	30 (6.0%)	14 (4.6%)	91 (18.2%)	
I do not know	150 (29.8%)	78 (25.7%)	83 (16.6%)	
I do not want to answer	14 (2.8%)	4 (1.3%)	53 (10.6%)	

For peer review only

Questions	Indonesia (n=503)	Thailand (n=304)	Viet Nam (n=501)	P value
Other	1 (0.2%)	0 (0%)	2 (0.4%)	
**Behaviour regulation (TDF-14): Any training, lectures, classes or meetings that provide you knowledge about local/national/international guidelines for BC sampling (Q5-5)?**				
No	153 (30.4%)	64 (21.1%)	52 (10.4%)	<0.001
Yes, infrequent (less than once a year)	90 (17.9%)	87 (28.6%)	111 (22.2%)	
Yes, occasionally (at least once a year)	109 (21.7%)	84 (27.6%)	196 (39.1%)	
Yes, regularly	53 (10.5%)	22 (7.2%)	61 (12.2%)	
I do not know	91 (18.1%)	46 (15.1%)	74 (14.8%)	
I do not want to answer	5 (1.0%)	1 (0.3%)	6 (1.2%)	
Other	2 (0.4%)	0 (0%)	1 (0.2%)	
**Behaviour regulation (TDF-14): any procedures that support you or doctors to order or regulate ordering of BC per local/national/international guidelines (Q5-6)?**				
No	129 (25.7%)	71 (23.4%)	76 (15.2%)	<0.001
Poster	57 (11.3%)	40 (13.2%)	66 (13.2%)	0.62
Standard order form	120 (23.9%)	90 (29.6%)	107 (21.4%)	0.03
Computer system to remind ordering BC	25 (5.0%)	14 (4.6%)	74 (14.8%)	<0.001
case review (e.g. grand round; morning ward round, clinical meetings, and BC is often mentioned)	76 (15.1%)	86 (28.3%)	164 (32.7%)	<0.001
Stewardship programme and reviewing BC is included in the programme	61 (12.1%)	25 (8.2%)	121 (24.2%)	<0.001
Local hospital guideline (e.g. standard operating procedure [SOP])	113 (22.5%)	77 (25.3%)	162 (32.3%)	0.002
I do not know	107 (21.3%)	49 (16.1%)	66 (13.2%)	0.003
I do not want to answer	9 (1.8%)	2 (0.7%)	15 (3.0%)	0.07
**Social influence (TDF-12): To what extent do you order BC because you are following local norms (Q6-1)?**				
All the time (>95-100% of the time)	50 (9.9%)	67 (22.0%)	64 (12.8%)	<0.001
Often (75-95% of the time)	130 (25.8%)	166 (54.6%)	174 (34.7%)	
Moderately (25-74% of the time)	84 (16.7%)	41 (13.5%)	144 (28.7%)	
Occasionally (5-24% of the time)	67 (13.3%)	15 (4.9%)	40 (8.0%)	
Rarely (ranging from never <5% of the time)	80 (15.9%)	8 (2.6%)	40 (8.0%)	
I do not know	87 (17.3%)	7 (2.3%)	25 (5.0%)	
I do not want to answer	5 (1.0%)	0 (0%)	14 (2.8%)	
**Social influence (TDF-12): Influence from nurses (Q6-2-1)? Positive influence could mean facilitate, support or encourage BC sampling. Negative influence could mean hinder or discourage BC sampling.**				
Very positive influence	46 (9.1%)	29 (9.5%)	60 (12.0%)	<0.001
Positive influence	230 (45.7%)	103 (33.9%)	154 (30.7%)	
Neither positive nor negative influence	162 (32.2%)	122 (40.1%)	228 (45.5%)	
Negative influence	15 (3.0%)	26 (8.6%)	25 (5.0%)	
Very negative influence	1 (0.2%)	1 (0.3%)	0 (0%)	
I do not know	45 (8.9%)	19 (6.3%)	30 (6.0%)	
I do not want to answer	4 (0.8%)	4 (1.3%)	4 (0.8%)	

For peer review only - http://bmjopen.bmj.com/site/about/guidelines.xhtml

**Questions**	**Indonesia (n=503)**	**Thailand (n=304)**	**Viet Nam (n=501)**	**P value**
**Social influence (TDF-12): Influence from final-year medical students (Q6-2-2)?**				
Very positive influence	29 (5.8%)	22 (7.2%)	30 (6.0%)	0.004
Positive influence	155 (30.8%)	87 (28.6%)	104 (20.8%)	
Neither positive nor negative influence	249 (49.5%)	157 (51.6%)	315 (62.9%)	
Negative influence	4 (0.8%)	3 (1.0%)	6 (1.2%)	
Very negative influence	1 (0.2%)	1 (0.3%)	0 (0%)	
I do not know	60 (11.9%)	27 (8.9%)	42 (8.4%)	
I do not want to answer	5 (1.0%)	7 (2.3%)	4 (0.8%)	
**Social influence (TDF-12): Influence from Interns (Q6-2-3)?**				
Very positive influence	31 (6.2%)	41 (13.5%)	33 (6.6%)	<0.001
Positive influence	182 (36.2%)	134 (44.1%)	170 (33.9%)	
Neither positive nor negative influence	205 (40.8%)	96 (31.6%)	251 (50.1%)	
Negative influence	5 (1.0%)	4 (1.3%)	3 (0.6%)	
Very negative influence	1 (0.2%)	0 (0%)	1 (0.2%)	
I do not know	70 (13.9%)	24 (7.9%)	38 (7.6%)	
I do not want to answer	9 (1.8%)	5 (1.6%)	5 (1.0%)	
**Social influence (TDF-12): Influence from residents (Q6-2-4)?**				
Very positive influence	64 (12.7%)	73 (24.0%)	79 (15.8%)	<0.001
Positive influence	270 (53.7%)	138 (45.4%)	219 (43.7%)	
Neither positive nor negative influence	109 (21.7%)	63 (20.7%)	161 (32.1%)	
Negative influence	2 (0.4%)	3 (1.0%)	1 (0.2%)	
Very negative influence	0 (0%)	0 (0%)	1 (0.2%)	
I do not know	51 (10.1%)	23 (7.6%)	37 (7.4%)	
I do not want to answer	7 (1.4%)	4 (1.3%)	3 (0.6%)	
**Social influence (TDF-12): Influence from doctors (Q6-2-5)?**				
Very positive influence	82 (16.3%)	62 (20.4%)	67 (13.4%)	<0.001
Positive influence	293 (58.3%)	125 (41.1%)	216 (43.1%)	
Neither positive nor negative influence	90 (17.9%)	85 (28.0%)	188 (37.5%)	
Negative influence	6 (1.2%)	3 (1.0%)	3 (0.6%)	
Very negative influence	0 (0%)	3 (1.0%)	1 (0.2%)	
I do not know	29 (5.8%)	23 (7.6%)	15 (3.0%)	
I do not want to answer	3 (0.6%)	3 (1.0%)	11 (2.2%)	
**Social influence (TDF-12): Influence from consultants (Q6-2-6)?**				
Very positive influence	172 (34.2%)	117 (38.5%)	109 (21.8%)	<0.001
Positive influence	255 (50.7%)	125 (41.1%)	261 (52.1%)	
Neither positive nor negative influence	38 (7.6%)	41 (13.5%)	113 (22.6%)	
Negative influence	5 (1.0%)	4 (1.3%)	4 (0.8%)	
Very negative influence	1 (0.2%)	2 (0.7%)	0 (0%)	
I do not know	26 (5.2%)	11 (3.6%)	13 (2.6%)	
I do not want to answer	6 (1.2%)	4 (1.3%)	1 (0.2%)	
**Social influence (TDF-12): Influence from head of department (Q6-2-7)?**				
Very positive influence	81 (16.1%)	51 (16.8%)	135 (26.9%)	<0.001
Positive influence	254 (50.5%)	89 (29.3%)	252 (50.3%)	
Neither positive nor negative influence	104 (20.7%)	119 (39.1%)	95 (19.0%)	
Negative influence	10 (2.0%)	6 (2.0%)	6 (1.2%)	
Very negative influence	0 (0%)	1 (0.3%)	0 (0%)	

For peer review only - http://bmjopen.bmj.com/site/about/guidelines.xhtml

Questions	Indonesia (n=503)	Thailand (n=304)	Viet Nam (n=501)	P value
I do not know	48 (9.5%)	34 (11.2%)	11 (2.2%)	
I do not want to answer	6 (1.2%)	4 (1.3%)	2 (0.4%)	
**Social influence (TDF-12): Influence from executive or administrative level of the hospital (Q6-2-8)?**				
Very positive influence	55 (10.9%)	35 (11.5%)	101 (20.2%)	<0.001
Positive influence	188 (37.4%)	67 (22.0%)	216 (43.1%)	
Neither positive nor negative influence	169 (33.6%)	145 (47.7%)	154 (30.7%)	
Negative influence	21 (4.2%)	8 (2.6%)	7 (1.4%)	
Very negative influence	8 (1.6%)	2 (0.7%)	1 (0.2%)	
I do not know	57 (11.3%)	42 (13.8%)	19 (3.8%)	
I do not want to answer	5 (1.0%)	5 (1.6%)	3 (0.6%)	
**Social influence (TDF-12): Influence from patients (Q6-2-9)?**				
Very positive influence	43 (8.5%)	44 (14.5%)	57 (11.4%)	<0.001
Positive influence	197 (39.2%)	74 (24.3%)	148 (29.5%)	
Neither positive nor negative influence	197 (39.2%)	141 (46.4%)	250 (49.9%)	
Negative influence	18 (3.6%)	14 (4.6%)	21 (4.2%)	
Very negative influence	1 (0.2%)	1 (0.3%)	1 (0.2%)	
I do not know	44 (8.7%)	26 (8.6%)	20 (4.0%)	
I do not want to answer	3 (0.6%)	4 (1.3%)	4 (0.8%)	
**Social influence (TDF-12): Influence from family of patients (Q6-2-10)?**				
Very positive influence	32 (6.4%)	21 (6.9%)	34 (6.8%)	<0.001
Positive influence	171 (34.0%)	40 (13.2%)	119 (23.8%)	
Neither positive nor negative influence	221 (43.9%)	186 (61.2%)	282 (56.3%)	
Negative influence	23 (4.6%)	20 (6.6%)	39 (7.8%)	
Very negative influence	3 (0.6%)	2 (0.7%)	2 (0.4%)	
I do not know	50 (9.9%)	30 (9.9%)	19 (3.8%)	
I do not want to answer	3 (0.6%)	5 (1.6%)	6 (1.2%)	
**Emotions (TDF-13): Any emotional factors (Q6-4)?**				
Yes	51 (10.1%)	10 (3.3%)	32 (6.4%)	0.001
**Gender (Q7-2)**				
Female	263 (52.3%)	195 (64.1%)	222 (44.3%)	<0.001
Male	236 (46.9%)	106 (34.9%)	263 (52.5%)	
Other	1 (0.2%)	0 (0%)	0 (0%)	
I do not want to answer	3 (0.6%)	3 (1.0%)	16 (3.2%)	
**Hospital bed size (Q7-3)**				
<200	99 (19.7%)	35 (11.5%)	24 (4.8%)	<0.001
201-400	107 (21.3%)	46 (15.1%)	29 (5.8%)	
401-600	72 (14.3%)	39 (12.8%)	62 (12.4%)	
601-1,000	66 (13.1%)	45 (14.8%)	144 (28.7%)	
1,001-2,000	39 (7.8%)	82 (27.0%)	125 (25.0%)	
> 2,000	27 (5.4%)	30 (9.9%)	74 (14.8%)	
I do not know	89 (17.7%)	27 (8.9%)	35 (7.0%)	
I do not want to answer	4 (0.8%)	0 (0%)	8 (1.6%)	
**Department (Q7-4)**				
Internal medicine	149 (29.6%)	155 (51.0%)	146 (29.1%)	<0.001
Pediatrics	65 (12.9%)	43 (14.1%)	45 (9.0%)	0.05
Infection disease division/department	12 (2.4%)	5 (1.6%)	56 (11.2%)	<0.001
Surgery	21 (4.2%)	45 (14.8%)	81 (16.2%)	<0.001

For peer review only

Questions	Indonesia (n=503)	Thailand (n=304)	Viet Nam (n=501)	P value
Orthopaedics	6 (1.2%)	18 (5.9%)	14 (2.8%)	0.001
Obstetrics / Gynaecology	20 (4.0%)	29 (9.5%)	7 (1.4%)	<0.001
Emergency department	112 (22.3%)	34 (11.2%)	29 (5.8%)	<0.001
Intensive care unit	45 (8.9%)	13 (4.3%)	51 (10.2%)	0.01
I do not want to answer	24 (4.8%)	25 (8.2%)	52 (10.4%)	0.004
Other	137 (27.2%)	29 (9.5%)	58 (11.6%)	<0.001

Gray color represents questions that were asked to subsets of participants. [1] Included primary health care, clinic, retired and answers as role of doctors (including residents, interns and medical students).

For peer review only

Appendix S5: Sample quotes

TDF domains	Themes	Sample quotes
Goal	Priority of BC	• "If other urgent examinations are to be required, BC could be delayed." (Vietnamese respondent [barrier]).<br>• "Early blood cultures should be encouraged for patients presenting with infection before antibiotics are given" (Vietnamese respondent [enabler])<br>• "BC should be performed, although the results are often negative. We can't wait for patients not responding to empirical antibiotics before starting BC. It could lead to a prolonged hospital stay" (Indonesian respondent [enabler])
Social professional role and identity	Level of doctors who can order or initiate an order for BC	• "Medical students can order BC; however, medical students must have a signature of a supervising medical doctor together all the time." (Thai respondent [enabler])<br>• "Medical doctors in charge hold the decisions of ordering BC. However, residents (medical doctors who are currently under postgraduate clinical doctors) could report (to medical doctors in charge) which patients need BC." (Indonesian respondent [barrier])
	Perception about their role to order or initiate an order for BC.	
	Perception about their role to draw blood for BC	
Belief about consequences	Perception that BC is helpful	• "(BC is helpful because) immediate use of BC and prior to giving antibiotics can inform whether a patient has bacteraemia or not, what organism is the cause, and which antibiotic would be appropriate." (Thai respondent [enabler])<br>• "(BC is helpful because) BC shortens the time to find the agent and shortens the treatment time for the patient" (Vietnamese respondent)<br>• "(BC is helpful because) BC can reduce irrational antibiotic prescriptions." (Indonesian respondent)
	Perception that BC is unnecessary	• "(BC is unnecessary because) BC often requires a long time to generate the results. Hence, the patient's condition has improved with empirical antibiotics when BC results are generated." (Indonesian respondent [barrier])<br>• "(BC is unnecessary because) laboratory often causes contamination, making the result irrelevant to clinical signs." (Thai respondent [barrier])<br>• "(BC is unnecessary because) most patients have self-medication with antibiotics at home, so BC often yields undesirable results." (Vietnamese respondent [barrier])<br>• "(BC is unnecessary because) time to return results is slow and most of them do not find pathogenic bacteria." (Vietnamese respondent [barrier])

For peer review only - http://bmjopen.bmj.com/site/about/guidelines.xhtml

TDF domains	Themes	Sample quotes
		• "BCs are not useful when the focal point of the infection is clear and the patient responds well to treatment." (Vietnamese respondent [barrier])
Intention	Intention to follow guidelines	• A guideline on BC examination should be written in detail, reviewed multiple times, monitored and followed with the appropriate rewards and punishment. (Vietnamese respondent [enabler]
Knowledge	Awareness of guidelines	
	Training	• "I have not learnt about the local recommendation for BC sampling in my university hospital." (Indonesian respondent [barrier]).<br>• "BC has not been highlighted in the clinics when I have Bed Side Teaching, Case Review, Tutorials, etc. It is recommended to do as ideal as is written in the literature." (Indonesian respondent [barrier])
Social influence	Norms of BC sampling	• "Social factors could influence diagnosis and therapy." (Indonesian respondent [barrier/enabler])
	Influences from healthcare workers, patients and family of patients	• "The patient's families often have a strong influence on patients. They often decide not to provide consent to BC." (Indonesian respondent [barrier])<br>• "Negative influence in the order of BC is cost. Supervisor or the executives (of the hospitals) gave an order to control the cost." (Thai respondent [barrier])<br>• "The patient's relatives are not satisfied with the cost of (BC) testing." (Vietnamese respondent [barrier]).<br>• "Because people do not understand, when ordering BC, they often complain." (Vietnamese respondent [barrier])<br>• "Some patients think that physicians and other healthcare workers only perform BC examinations for money." (Indonesian respondent [barrier]).<br>• "Sometimes, when the blood puncture fails on the first try, patients and their families refuse to have more blood drawn." (Indonesian respondent [barrier]).
Reinforcement	Consequences that discourage BC sampling	• "Warnings are given due to the costly examination, especially for patients insured with the Healthcare and Social Security Agency." (Indonesian respondent [barrier])<br>• "Sometimes, the cost of BC cannot be reimbursed, and the doctor has to pay." (Vietnamese respondent [barrier])<br>• "Occasionally, the insurance assessment agency often asks questions, requires explanations and can make it difficult to limit the order of BC for patients." (Vietnamese respondent [barrier])
	Consequences that encourage BC sampling	• "The consequences are usually minimal. The hospital prioritizes the clinical improvement and satisfaction of the patients and their families instead of conducting according to the guidelines or minimizing antibiotic resistance." (Vietnamese respondent [barrier])<br>• "If the patient dies without BC testing, it will be questioned in the death case report." (Indonesian respondent [enabler])<br>• "If (we) do not follow the recommendation for (BC) diagnostic tests, there will be a verbal reprimand in order

For peer review only - http://bmjopen.bmj.com/site/about/guidelines.xhtml

TDF domains	Themes	Sample quotes
		to make sure that the care is up to the standard." (Thai respondent [enabler])<br>• "There are no incentives, rewards or penalties." (Vietnamese respondent [lack of enabler])<br>• "The case of septic shock without a BC will be reprimanded." (Vietnamese respondent [enabler])
Behavioural regulation	Regulations on cost reimbursement	• "National insurance coverage and hospital regulation could inhibit BC examination." (Indonesian respondent [barrier])<br>• "The insurance often disapproves of BC examination. It is only approved when patients are admitted to the ICU or HCU [High Care Unit]." (Indonesian respondent [barrier])<br>• "It is affected by the insurance. Healthcare and Social Security Agency in Indonesia only covers septic patients around two million rupiahs/patient [about 138 US$], it is not sufficient to cover the resources required, including BC examinations." (Indonesian respondent [barrier].<br>• "Some hospitals allow only three laboratory tests; therefore, (doctors) must select laboratory tests for patients." (Thai respondent [barrier])<br>• "When the final diagnosis does not match, (the cost of BC) will not be paid by Health Insurance." (Vietnamese respondent [barrier])<br>• "Medical professionals often object to BC due to tiredness [disheartened feeling] and the consequence of reduced reimbursement." (Vietnamese respondent [barrier])<br>• "It is difficult (to order BC) because there are restrictions from the financial coverage on the Healthcare and Social Security Agency." (Indonesian respondent)
	Procedures to support or regulate doctors to order BC	•
Environmental context and resources	Perceived cost-effectiveness of BC	• "BC is still not cost-effective for my hospital" (Indonesian respondent [barrier]).<br>• "BC is not cost-effective" (Vietnamese respondent [barrier])
	Availability of microbiology laboratories, transport modalities, resources and consumables	• "Hospitals that do not have a microbiology laboratory cannot obtain culture results. If you still want to take BC, you have to send it to another hospital, it will cost the patient more" (Vietnamese respondent [barrier])
	Out-of-pocket	• "BC is essential, but it costs a lot (Indonesia Rp 750.000,00 [about 52US$]), and many patients could not afford it." (Indonesian respondent [barrier])<br>• "Patients usually refuse BC due to the cost." (Indonesian respondent [barrier])
Emotion	Fear or anxiety of healthcare providers	• "In some patients with blood-borne infectious diseases, doctors are afraid to draw blood." (Vietnamese respondent [barrier])<br>• "Nurses are afraid to draw a lot of blood." (Vietnamese respondent [barrier])

For peer review only - http://bmjopen.bmj.com/site/about/guidelines.xhtml

TDF domains	Themes	Sample quotes
	Fear or anxiety of patients or families of patients	• "Patient and their families are afraid of contracting blood-transmitted diseases." (Indonesian respondent [barrier])<br>• "Patient are afraid to be drawn a lot of blood." (Vietnamese respondent [barrier])<br>• "Fear of pain. Fear of needle" (Thai respondent [barrier]<br>• "Anxiety, panic or uncooperative attitude." (Vietnamese respondent [barrier])<br>• "Patients are afraid that taking a lot of blood will cause anemia." (Vietnamese respondent [barrier])
Optimism	Confidence that BC will be appropriately sampled and processed in the laboratory	
Skill	Skill in drawing blood for BC	
Memory, attention and decision processes	Patients who are already on antibiotics or have anemia	"In patients who have already received antibiotics, BC is not meaningful." (Vietnamese respondent [barrier]
	Clinical presentations for deciding to order BC	"Patients who are receiving palliative-care may not be tested for BC, even though there are criteria for it" (Thai respondent [barrier])
Beliefs about capabilities	Belief in their own capability to draw blood	
	Belief in capability of those who are tasked to draw blood	

For peer review only - http://bmjopen.bmj.com/site/about/guidelines.xhtml

Appendix S6. Associations between barriers/enablers and the responses that they would definitely take BC in the case scenario

Barriers or enablers	Indonesia[1] (n=503)	Thailand[1] (n=304)	Viet Nam[1] (n=501)	Odds ratio[2]	P value
**TDF domain: Goals**					
**How often do you obtain BC prior to receiving empirical antibiotic in patients presenting with sepsis?**					
All the time / Often (>75-100% of the time)	45.4% (113/249)	91.6% (251/274)	58.6% (222/379)	4.25 (3.04-5.94)	<0.001
Moderately / Occasionally / Rarely (0-74% of the time)	15.6% (33/212)	73.1% (19/26)	22.1% (21/95)	1.0	
**How often do you obtain BC prior to receiving empirical antibiotic in patients presenting with septic shock?**					
All the time / Often (>75-100% of the time)	44.8% (111/248)	90.1% (264/293)	56.4% (221/392)	3.71 (2.61-5.27)	<0.001
Moderately / Occasionally / Rarely (0-74% of the time)	15.4% (32/208)	83.3% (5/6)	25.6% (22/86)	1.0	
**TDF domain: Social professional role and identity**					
**Current job**					
Medical doctor – an executive level	15.4% (2/13)	60.0% (2/3)	35.3% (6/17)	0.20 (0.09-0.47)	<0.001
Medical doctor – a consultant level	34.4% (25/73)	90.7% (68/75)	49.2% (97/197)	0.48 (0.33-0.69)	
Medical doctor – a general physician level	10.5% (13/124)	81.6% (31/38)	46.0% (51/111)	0.27 (0.18-0.40)	
Medical doctor – a resident/registra/fellow level	48.8% (82/168)	93.7% (59/63)	68.3% (69/101)	1.0	
Intern – recent medical school graduate	12.1% (4/33)	88.6% (31/35)	35.7% (5/14)	0.26 (0.14-0.49)	
Final-year medical student	34.4% (31/90)	92.1% (81/88)	40.7% (24/59)	0.50 (0.33-0.76)	
**Perception about their role to order or initiate an order for BC**					
Very appropriate / Appropriate	45.5% (120/264)	91.2% (250/274)	61.2% (195/317)	3.36 (2.50-4.51)	<0.001
Uncertain / Inappropriate / Very inappropriate	16% (36/225)	78.6% (22/28)	33.3% (55/165)	1.0	
**Perception about their role to draw blood for BC[3]**					
Very appropriate / Appropriate	38.0% (27/71)	87.8% (65/74)	52.4% (54/103)	1.94 (1.04-3.64)	0.04
Uncertain / Inappropriate / Very inappropriate	28.6% (4/14)	94.8% (55/58)	25.6% (10/39)	1.0	
**TDF domain: Belief about consequences**					
**BC is helpful in clinical decision**					
Strongly agree / Agree	31.5% (152/482)	89.9% (267/297)	54.1% (237/438)	2.96 (1.71-5.12)	<0.001
Uncertain / Disagree / Strongly disagree	23.5% (4/17)	85.7% (6/7)	23.7% (14/59)	1.0	
**BC is helpful to rule in an infection**					
Strongly agree / Agree	31.9% (149/467)	90.1% (254/282)	52.4% (220/420)	1.58 (1.04-2.39)	0.03
Uncertain / Disagree / Strongly disagree	21.9% (7/32)	100% (18/18)	40.3% (31/77)	1.0	
**BC is helpful to rule out an infection**					
Strongly agree / Agree	31.2% (123/394)	88.2% (149/169)	47.7% (105/220)	0.91 (0.69-1.19)	0.49
Uncertain / Disagree / Strongly disagree	31.4% (33/105)	91.7% (122/133)	52.9% (146/276)	1.0	

For peer review only - http://bmjopen.bmj.com/site/about/guidelines.xhtml

Barriers or enablers	Indonesia[1] (n=503)	Thailand[1] (n=304)	Viet Nam[1] (n=501)	Odds ratio[2]	P value
**BC is helpful to detecting AMR bacterial infections**					
Strongly agree / Agree	31.3% (152/485)	89.2% (256/287)	51.2% (217/424)	1.26 (0.80-1.98)	0.32
Uncertain / Disagree / Strongly disagree	28.6% (4/14)	100% (16/16)	45.2% (33/73)	1.0	
**BC is helpful in adjusting antibiotics**					
Strongly agree / Agree	31.0% (152/490)	89.7% (269/300)	52.2% (225/431)	1.50 (0.90-2.50)	0.12
Uncertain / Disagree / Strongly disagree	44.4% (4/9)	100% (4/4)	39.1% (25/64)	1.0	
**BC can reduce overuse of antibiotics**					
Strongly agree / Agree	30.7% (141/460)	89.0% (243/273)	52.2% (211/404)	1.08 (0.74-1.58)	0.68
Uncertain / Disagree / Strongly disagree	38.5% (15/39)	97% (30/31)	42.0% (40/93)	1.0	
**BC can reduce length of hospital stay**					
Strongly agree / Agree	31.5% (120/381)	91.5% (204/223)	55.3% (183/331)	1.53 (1.14-2.04)	0.004
Uncertain / Disagree / Strongly disagree	29.6% (34/115)	86.1% (68/79)	41.0% (68/166)	1.0	
**BC can reduce patient mortality**					
Strongly agree / Agree	32.8% (133/405)	89.0% (227/255)	55.0% (200/364)	1.61 (1.18-2.20)	0.003
Uncertain / Disagree / Strongly disagree	23.9% (22/92)	95.7% (44/46)	38.6% (51/132)	1.0	
**Accumulative results of BC are helpful in understanding epidemiology of AMR bacterial infections**					
Strongly agree / Agree	31.5% (152/483)	90.5% (258/285)	52.5% (240/457)	2.89 (1.60-5.19)	<0.001
Uncertain / Disagree / Strongly disagree	21.4% (3/14)	76.5% (13/17)	25% (10/40)	1.0	
**BC is unnecessary because antibiotic therapy can be determined based on clinical presentation**					
Strongly agree / Agree	20.8% (21/101)	83.6% (46/44)	33.8% (24/71)	0.51 (0.36-0.73)	<0.001
Uncertain / Disagree / Strongly disagree	33.9% (134/395)	91.1% (226/248)	53.3% (228/428)	1.0	
**The therapeutic consequence of BC is questionable**					
Strongly agree / Agree	32.3% (30/93)	88.0% (73/83)	41.0% (25/61)	0.84 (0.59-1.19)	0.32
Uncertain / Disagree / Strongly disagree	30.6% (120/392)	91.2% (196/215)	51.9% (223/430)	1.0	
**The scientific basis of the guideline on BC is questionable**					
Strongly agree / Agree	32.0% (17/53)	87.3% (69/79)	32.8% (19/58)	0.66 (0.45-0.98)	0.04
Uncertain / Disagree / Strongly disagree	30.4% (132/433)	91.2% (198/217)	53.2% (231/434)	1.0	
**BC is unnecessary because results are often delayed**					
Strongly agree / Agree	18.9% (24/127)	82.1% (32/39)	30.2% (16/53)	0.48 (0.33-0.69)	<0.001
Uncertain / Disagree / Strongly disagree	35.2% (129/367)	90.9% (240/264)	53.0% (236/445)	1.0	
**BC is unnecessary because results are often not interpretable**					
Strongly agree / Agree	25.0% (13/52)	77.3% (17/22)	29.7% (11/37)	0.54 (0.34-0.87)	0.01
Uncertain / Disagree / Strongly disagree	31.7% (140/442)	90.8% (255/281)	52.3% (241/461)	1.0	
**BC is unnecessary because results are often negative or no growth**					

For peer review only - http://bmjopen.bmj.com/site/about/guidelines.xhtml

Barriers or enablers	Indonesia[1] (n=503)	Thailand[1] (n=304)	Viet Nam[1] (n=501)	Odds ratio[2]	P value
Strongly agree / Agree	30.8% (20/65)	81.3% (26/32)	28.0% (14/50)	0.58 (0.39-0.88)	0.01
Uncertain / Disagree / Strongly disagree	30.8% (131/426)	91.1% (247/271)	53.1% (238/448)	1.0	
**BC is unnecessary because cultures are often contaminated**					
Strongly agree / Agree	26.3% (19/72)	79.3% (23/29)	34.2% (14/41)	0.64 (0.42-0.98)	0.04
Uncertain / Disagree / Strongly disagree	31.9% (133/417)	90.9% (249/274)	52.2% (236/452)	1.0	
**BC is unnecessary because results often do not agree with clinical signs**					
Strongly agree / Agree	34.0% (18/53)	88.9% (24/27)	23.5% (8/34)	0.77 (0.48-1.22)	0.27
Uncertain / Disagree / Strongly disagree	30.8% (135/439)	89.9% (249/277)	52.9% (241/456)	1.0	
**BC is unnecessary because it is too expensive**					
Strongly agree / Agree	25.5% (24/94)	80.0% (24/30)	32.7% (17/52)	0.62 (0.42-0.92)	0.02
Uncertain / Disagree / Strongly disagree	32.4% (129/398)	91.2% (249/273)	52.9% (229/443)	1.0	
**BC is not benefiting the patients**					
Strongly agree / Agree	14.0% (15/107)	84.0% (21/25)	19.4% (7/36)	0.37 (0.24-0.57)	<0.001
Uncertain / Disagree / Strongly disagree	35.8% (136/380)	90.1% (246/273)	53.0% (239/451)	1.0	
**BC is unnecessary because a contaminated result often leads to wrong therapeutic approaches**					
Strongly agree / Agree	30.4% (7/23)	86.4% (19/22)	20.0% (6/30)	0.53 (0.30-0.95)	0.03
Uncertain / Disagree / Strongly disagree	31.5% (148/470)	90.1% (254/282)	52.5% (245/467)	1.0	
**It is not too late to collect BC later, particularly if patients do not improve after receiving empirical antibiotic treatment**					
Strongly agree / Agree	13.8% (19/138)	88.3% (143/162)	31.2% (38/122)	0.37 (0.27-0.51)	<0.001
Uncertain / Disagree / Strongly disagree	38.1% (134/352)	91.6% (130/142)	57.2% (214/373)	1.0	
**Quality of laboratory is questionable**					
Strongly agree / Agree	24.2% (22/91)	84.2% (32/38)	26.6% (17/64)	0.48 (0.33-0.70)	<0.001
Uncertain / Disagree / Strongly disagree	32.7% (128/391)	90.3% (232/257)	54.1% (230/435)	1.0	
**Levels of local antibiotic resistance are low**					
Strongly agree / Agree	34.7% (17/49)	76.9% (20/26)	32.0% (16/50)	0.64 (0.41-0.98)	0.04
Uncertain / Disagree / Strongly disagree	31.3% (135/432)	91.1% (246/270)	52.8% (235/445)	1.0	
**TDF domain: Intention**					
**Intention to follow local guidelines[3]**					
All the time / Often (>75-100% of the cases)	51.7% (89/172)	90.5% (142/157)	64.7% (183/283)	2.92 (1.88-4.53)	<0.001
Moderately / Occasionally / Rarely (0-74% of the cases)	18.6% (11/59)	100% (12/12)	37.7% (23/61)	1.0	
**TDF domain: Knowledge**					
**Awareness of local guidelines**					
Yes	42.7% (102/239)	91.1% (154/169)	59.5% (206/346)	2.55 (1.93-3.38)	<0.001
No	21.1% (53/251)	89.3% (117/131)	29.4% (42/143)	1.0	

For peer review only - http://bmjopen.bmj.com/site/about/guidelines.xhtml

Barriers or enablers	Indonesia[1] (n=503)	Thailand[1] (n=304)	Viet Nam[1] (n=501)	Odds ratio[2]	P value
**Awareness of international guidelines**					
Yes	38.9% (138/226)	90.8% (128/141)	65.9% (147/223)	1.97 (1.50-2.57)	<0.001
No	25.4% (67/264)	89.9% (143/159)	38.0% (101/266)	1.0	
**Any training, lectures, classes or meetings that provide knowledge about guidelines for BC sampling**					
Available	36.2% (92/254)	92.2% (178/193)	53.5% (197/368)	1.68 (1.18-2.38)	0.004
Not available	21.7% (33/152)	82.8% (53/64)	46.2% (24/52)	1.0	
**TDF domain: Social influences**					
**To what extent do you order BC in your hospital because you are following local norms? [4]**					
All the time / Often (>75-100% of the time)	45.3% (81/179)	90.1% (210/233)	61.3% (146/238)	2.20 (1.67-2.90)	<0.001
Moderately / Occasionally / Rarely (0-74% of the time)	22.2% (51/230)	90.6% (58/64)	41.3% (92/223)	1.0	
**TDF domain: Reinforcement**					
**Positive consequences if doctors order a BC when it is recommended**					
Yes	29.9% (20/67)	86.0% (49/57)	42.4% (72/170)	0.53 (0.37-0.74)	<0.001
No	32.0% (136/425)	90.6% (222/245)	57.4% (160/279)	1.0	
**Negative consequences if doctors do not order a BC when it is recommended**					
Yes	39.4% (39/99)	90.1% (127/141)	50.0% (112/224)	0.87 (0.63-1.21)	0.42
No	30.1% (117/389)	89.4% (144/161)	55.6% (120/216)	1.0	
**Negative consequences if doctors order a BC when it is recommended**					
Yes	29.2% (19/65)	86.0% (49/57)	41.4% (67/162)	0.48 (0.34-0.67)	<0.001
No	32.3% (136/421)	90.5% (220/243)	60.1% (170/283)	1.0	
**TDF domain: Behavioural regulation**					
**Considering whether "patients have a health scheme or insurance that covers the cost of BC" as a reason for deciding to do BC sampling [5]**					
Yes	27.7% (31/112)	92.6% (25/27)	38.6% (22/57)	0.82 (0.57-1.18)	0.29
No	32.4% (126/389)	89.5% (248/277)	52.0% (230/442)	1.0	
**Considering whether "Patients are likely to have a final diagnosis that includes the cost of BC in the package of fee for service" as a reason for deciding to do BC sampling [5]**					
Yes	33.8% (24/69)	96.4% (27/28)	41.8% (23/55)	1.04 (0.70-1.54)	0.85
No	30.8% (133/432)	89.1% (246/276)	51.6% (229/444)	1.0	

Barriers or enablers	Indonesia[1] (n=503)	Thailand[1] (n=304)	Viet Nam[1] (n=501)	Odds ratio[2]	P value
**Procedures that support doctors to order or regulate ordering of BC**					
No	44.7% (34/76)	88.7% (63/71)	24.2% (31/128)	1.0	0.006
Poster (and BC is mentioned)	36.8% 921/57)	92.5% (37/40)	51.5% (34/66)	1.13 (0.76-1.69)	
Standard order form for patients with sepsis (with BC written)	32.5% (39/120)	92.2% (83/90)	46.7% (50/107)	0.82 (0.59-1.14)	
Computer system to remind ordering BC	36.0% (9/25)	92.9% (13/14)	45% (33/73)	0.72 (0.48-1.15)	
case reviews (e.g. grand round; with BC often mentioned)	44.7% (34/76)	90.7% (78/86)	57.3% (94/164)	1.38 (0.94-2.00)	
Stewardship programmes (including BC)	49.2% (30/61)	92.0% (23/25)	58.7% (71/121)	1.33 (0.87-2.03)	
Local hospital guideline (e.g. standard operating procedure)	37.2% (42/113)	94.8% (73/77)	58.6% (95/162)	1.45 (1.06-1.99)	
**TDF domain: Environmental context and resources**					
**Do the benefits of BC outweigh the cost?**					
Very likely / likely	35.3% (109/309)	91.0% (211/232)	53.1% (216/407)	1.63 (1.17-2.26)	0.004
Uncertain / Unlikely / Very unlikely	22.0% (31/141)	86.7% (52/60)	42.3% (29/67)	1.0	
**How often are consumables for BC not available?**					
All the time / Often (>75-100% of the time)	31.3% (26/83)	88.9% (24/27)	34.2% (13/38)	0.81 (0.53-1.22)	0.32
Moderately / Occasionally / Rarely (0-74% of the time)	31.9% (114/357)	89.5% (231/258)	53.5% (222/415)	1.0	
**How often are laboratories not available or not functioning?**					
All the time / Often (>75-100% of the time)	28.9% (26/90)	90.9% (2/22)	48.8% (21/43)	0.94 (0.63-1.41)	0.78
Moderately / Occasionally / Rarely (0-74% of the time)	32.6% (114/350)	89.3% (233/261)	53.3% (216/405)	1.0	
**How often do patients have to pay for BC using their own money?**					
All the time / Often (>75-100% of the time)	22.4% (17/76)	92.7% (26/28)	47.1% (16/34)	0.79 (0.51-1.22)	0.29
Moderately / Occasionally / Rarely (0-74% of the time)	36.2% (93/257)	88.1% (178/202)	55.8% (208/373)	1.0	
**Considering whether "patients can afford the cost of BC" as a reason for deciding to do BC sampling**					
Yes	31.1% (33/106)	92.6% (25/27)	46.9% (30/64)	1.12 (0.79-1.61)	0.53
No	31.4% (124/395)	89.5% (248/277)	51.0% (222/435)	1.0	
**TDF domain: Emotion**					
**Any emotional factors**					
Yes	25.5% (13/51)	80% (8/10)	65.6% (21/32)	1.06 (0.65-1.71)	0.82
No	32.0% (144/450)	90.1% (265/294)	49.5% (231/467)	1.0	
**TDF domain: Optimism**					
**Optimistic that a BC will be sampled and processed in the laboratory appropriately**					
Strongly optimistic / Optimistic	33.3% (133/400)	90.5% (238/263)	54.4% (200/368)	1.78 (1.29-2.46)	<0.001
Neither / Pessimistic / Strongly pessimistic	20.7% (18/87)	88.6% (31/35)	39.8% (51/128)	1.0	
**TDF domain: Skills**					

For peer review only - http://bmjopen.bmj.com/site/about/guidelines.xhtml

Barriers or enablers	Indonesia[1] (n=503)	Thailand[1] (n=304)	Viet Nam[1] (n=501)	Odds ratio[2]	P value
**How skilled are you in drawing blood? [6]**					
Very good / Good	38.5% (15/39)	88.2% (30/34)	57.1% (40/70)	1.74 (1.02-2.97)	0.04
Fair / Poor / Very poor	31.8% (14/44)	93.1% (81/87)	35.1% (20/57)	1.0	
**TDF domain: Memory, attention and decision processes**					
**Even when BC is recommended, would you still order BC if patients are already on antibiotics**					
Definitely not order / likely not order	20.0% (6/30)	86.6% (58/67)	41.2% (14/34)	0.69 (0.42-1.11)	0.13
Maybe not order/ likely to still order / very likely to still order	31.2% (142/455)	90.6% (213/235)	51.3% (238/464)	1.0	
**Even when BC is recommended, would you still order BC if patients have anemia**					
Definitely not order / likely not order	21.3% (16/75)	91.9% (136/148)	47.4% (27/57)	0.89 (0.62-1.28)	0.55
Maybe not order/ likely to still order / very likely to still order	32.2% (128/398)	87.3% (130/149)	51.3% (220/429)	1.0	
**TDF domain: Beliefs about capabilities**					
**Are you confident that you can draw blood successfully? [6,7]**					
Strongly confident / Confident	34.7% (25/72)	89.1% (57/64)	51.9% (56/108)	1.39 (0.69-2.79)	0.36
Uncertain / Doubtful / Strongly doubtful	36.4% (4/11)	94.7% (54/57)	22.2% (4/18)	1.0	
**Are you confident that you can draw blood appropriately? [6,7]**					
Strongly confident / Confident	34.8% (24/69)	89.7% (70/78)	54.6% (54/99)	1.67 (0.88-3.17)	0.11
Uncertain / Doubtful / Strongly doubtful	35.7% (5/14)	95.2% (40/42)	22.2% (6/27)	1.0	
**Are you confident that others (who are tasked to draw blood in your hospital) can draw blood successfully? [7]**					
Strongly confident / Confident	30.7% (142/463)	90.1% (254/282)	52.5% (212/404)	1.35 (0.91-2.00)	0.13
Uncertain / Doubtful / Strongly doubtful	33.3% (11/33)	85.7% (18/21)	43.0% (40/93)	1.0	
**Are you confident that others (who are tasked to draw blood in your hospital) can draw blood appropriately? [7]**					
Strongly confident / Confident	31.0% (132/426)	89.6% (224/250)	52.8% (168/318)	1.20 (0.89-1.62)	0.23
Uncertain / Doubtful / Strongly doubtful	31.9% (22/69)	90.6% (48/53)	46.6% (83/178)	1.0	

[1] Percentage of participants who answered with "definitely take BC" in the case scenario are presented. For each question, participants who answered 'I do not know' or 'I do not want to answer' were excluded. [2] Estimated by using logistic regression models with random effects for countries, for types of hospital nested in the same country, and for professional roles nested in the same types of hospital. [3] Among those who answered that they know of local guidelines. [4] "Norms" means usual practice that are typical of or accepted within your hospital. [5] Included answers in Q1-7 (which were asked to those who answered that they knew of local guideline) and Q1-8 (which were asked to those who answered that they did not know of local guideline) (Appendix S3). [6] Among those who answered that their professional roles are tasked of drawing blood for BC. [7] "Successfully" means obtaining blood; "Appropriately" means that general recommendations for BC specimen collection such as aseptic technique are followed.

For peer review only - http://bmjopen.bmj.com/site/about/guidelines.xhtml

Appendix S7. Links between COM-B components (Capability, Opportunity, motivation and behaviour components), and suggested intervention types and policy options.

Links between COM-B components and intervention types*

Intervention types	COM-B components					
	Capability		Opportunity		Motivation	
	Psychological	Physical	Social	Physical	Reflective	Automatic
**Education**		X			X	
**Persuasion**					X	X
**Incentivisation**					X	X
**Coercion**					X	X
**Training**	X	X				
**Restriction**			X	X		
**Environmental restructuring**			X	X		X
**Modelling**						X
**Enablement**	X	X	X	X		X

* as previously published.[8]

Links between intervention types and policy categories*

Intervention types	Policy categories						
	Communi-cation/ Marketing	Guidelines	Fiscal	Regulation	Legislation	Environ-mental/ Social planning	Service provision
**Education**	X	X		X	X		X
**Persuasion**	X	X		X	X		X
**Incentivisation**	X	X	X	X	X		X
**Coercion**	X	X	X	X	X		X
**Training**		X	X	X	X		X
**Restriction**		X		X	X		
**Environmental restructuring**		X	X	X	X	X	
**Modelling**	X						X
**Enablement**		X	X	X	X	X	X

* as previously published.[8]

For peer review only

References

1. Chongsuvivatwong V, Phua KH, Yap MT, Pocock NS, Hashim JH, Chhem R, et al. Health and health-care systems in southeast Asia: diversity and transitions. Lancet. 2011;377(9763):429-37.
2. World Bank. GDP (current US$). Available from: https://data.worldbank.org/indicator/NY.GDP.MKTP.CD
3. Tangcharoensathien V, Limwattananon S, Patcharanarumol W, Thammatacharee J. Monitoring and evaluating progress towards Universal Health Coverage in Thailand. PLoS Med. 2014;11(9):e1001726.
4. Sumriddetchkajorn K, Shimazaki K, Ono T, Kusaba T, Sato K, Kobayashi N. Universal health coverage and primary care, Thailand. Bull World Health Organ. 2019;97(6):415-22.
5. Le QN, Blizzard L, Si L, Giang LT, Neil AL. The evolution of social health insurance in Vietnam and its role towards achieving universal health coverage. Health Policy OPEN. 2020;1:100011.
6. Thuan NT, Lofgren C, Lindholm L, Chuc NT. Choice of healthcare provider following reform in Vietnam. BMC Health Serv Res. 2008;8:162.
7. WHO. Out-of-pocket expenditure (OOP) per capita in US$. Available from: https://www.who.int/data/gho/data/indicators/indicator-details/GHO/out-of-pocket-expenditure-(oop)-per-capita-in-us
8. Michie S, van Stralen MM, West R. The behaviour change wheel: A new method for characterising and designing behaviour change interventions. Implementation Science. 2011;6(1):42.

For peer review only

Checklist for Reporting Of Survey Studies (CROSS)

Section/topic	Item	Item description	Reported on page #
**Title and abstract**			
Title and abstract	1a	State the word "survey" along with a commonly used term in title or abstract to introduce the study's design.	1, 3
	1b	Provide an informative summary in the abstract, covering background, objectives, methods, findings/results, interpretation/discussion, and conclusions.	3, 4
**Introduction**			
Background	2	Provide a background about the rationale of study, what has been previously done, and why this survey is needed.	6, 7
Purpose/aim	3	Identify specific purposes, aims, goals, or objectives of the study.	7
**Methods**			
Study design	4	Specify the study design in the methods section with a commonly used term (e.g., cross-sectional or longitudinal).	10
Data collection methods	5a	Describe the questionnaire (e.g., number of sections, number of questions, number and names of instruments used).	7 - 9
	5b	Describe all questionnaire instruments that were used in the survey to measure particular concepts. Report target population, reported validity and reliability information, scoring/classification procedure, and reference links (if any).	7 - 11
	5c	Provide information on pretesting of the questionnaire, if performed (in the article or in an online supplement). Report the method of pretesting, number of times questionnaire was pre-tested, number and demographics of participants used for pretesting, and the level of similarity of demographics between pre-testing participants and sample population.	9
	5d	Questionnaire if possible, should be fully provided (in the article, or as appendices or as an online supplement).	S3
Sample characteristics	6a	Describe the study population (i.e., background, locations, eligibility criteria for participant inclusion in survey, exclusion criteria).	7-10, S1
	6b	Describe the sampling techniques used (e.g., single stage or multistage sampling, simple random sampling, stratified sampling, cluster sampling, convenience sampling). Specify the locations of sample participants whenever clustered sampling was applied.	9-10
	6c	Provide information on sample size, along with details of sample size calculation.	9-10
	6d	Describe how representative the sample is of the study population (or target population if possible), particularly for population-based surveys.	9-10
Survey	7a	Provide information on modes of questionnaire administration, including the type and number of contacts, the location where the survey was conducted (e.g., outpatient	9-10

For peer review only

administration		room or by use of online tools, such as SurveyMonkey).	
	7b	Provide information of survey's time frame, such as periods of recruitment, exposure, and follow-up days.	9-10
	7c	Provide information on the entry process:<br>–>For non-web-based surveys, provide approaches to minimize human error in data entry.<br>–>For web-based surveys, provide approaches to prevent "multiple participation" of participants.	10
Study preparation	8	Describe any preparation process before conducting the survey (e.g., interviewers' training process, advertising the survey).	9
Ethical considerations	9a	Provide information on ethical approval for the survey if obtained, including informed consent, institutional review board [IRB] approval, Helsinki declaration, and good clinical practice [GCP] declaration (as appropriate).	33
	9b	Provide information about survey anonymity and confidentiality and describe what mechanisms were used to protect unauthorized access.	S3
Statistical analysis	10a	Describe statistical methods and analytical approach. Report the statistical software that was used for data analysis.	10-11
	10b	Report any modification of variables used in the analysis, along with reference (if available).	10-11, S1
	10c	Report details about how missing data was handled. Include rate of missing items, missing data mechanism (i.e., missing completely at random [MCAR], missing at random [MAR] or missing not at random [MNAR]) and methods used to deal with missing data (e.g., multiple imputation).	10-11, S1
	10d	State how non-response error was addressed.	31
	10e	For longitudinal surveys, state how loss to follow-up was addressed.	Not applicable
	10f	Indicate whether any methods such as weighting of items or propensity scores have been used to adjust for non-representativeness of the sample.	Not applicable
	10g	Describe any sensitivity analysis conducted.	S1
**Results**			
Respondent characteristics	11a	Report numbers of individuals at each stage of the study. Consider using a flow diagram, if possible.	13, S4, S5
	11b	Provide reasons for non-participation at each stage, if possible.	13, S4, S5
	11c	Report response rate, present the definition of response rate or the formula used to calculate response rate.	Not applicable

For peer review only

	11d	Provide information to define how unique visitors are determined. Report number of unique visitors along with relevant proportions (e.g., view proportion, participation proportion, completion proportion).	13, S4, S5
Descriptive results	12	Provide characteristics of study participants, as well as information on potential confounders and assessed outcomes.	17-25, S1, S5
Main findings	13a	Give unadjusted estimates and, if applicable, confounder-adjusted estimates along with 95% confidence intervals and p-values.	17-25, S1, S5
	13b	For multivariable analysis, provide information on the model building process, model fit statistics, and model assumptions (as appropriate).	Not applicable
	13c	Provide details about any sensitivity analysis performed. If there are considerable amount of missing data, report sensitivity analyses comparing the results of complete cases with that of the imputed dataset (if possible).	S1
**Discussion**			
Limitations	14	Discuss the limitations of the study, considering sources of potential biases and imprecisions, such as non-representativeness of sample, study design, important uncontrolled confounders.	31
Interpretations	15	Give a cautious overall interpretation of results, based on potential biases and imprecisions and suggest areas for future research.	31
Generalizability	16	Discuss the external validity of the results.	28-31
**Other sections**			
Role of funding source	17	State whether any funding organization has had any roles in the survey's design, implementation, and analysis.	31
Conflict of interest	18	Declare any potential conflict of interest.	32
Acknowledgements	19	Provide names of organizations/persons that are acknowledged along with their contribution to the research.	32

For peer review only
